# Indirect Formation of Peptide Bonds as a Prelude to Ribosomal Transpeptidation

**DOI:** 10.1021/jacs.4c10326

**Published:** 2024-12-18

**Authors:** Harvey J. A. Dale, John D. Sutherland

**Affiliations:** MRC Laboratory of Molecular Biology, Francis Crick Avenue, Cambridge CB2 0QH, U.K.

## Abstract

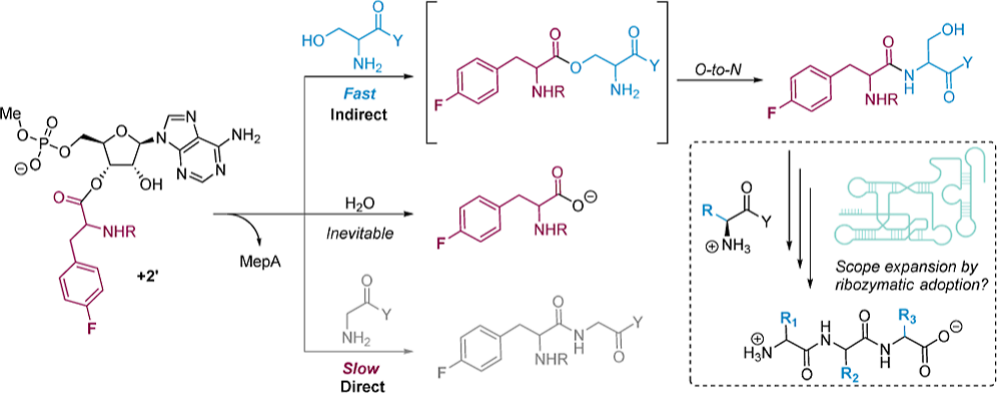

The catalytic competency of the ribosome in extant protein biosynthesis is thought to arise primarily from two sources: an ability to precisely juxtapose the termini of two key substrates—3′-aminoacyl and *N*-acyl-aminoacyl tRNAs—and an ability to ease direct transpeptidation by their desolvation and encapsulation. In the absence of ribosomal, or enzymatic, protection, however, these activated alkyl esters undergo efficient hydrolysis, while significant entropic barriers serve to hamper their intermolecular cross-aminolysis in bulk water. Given that the spontaneous emergence of a catalyst of comparable size and sophistication to the ribosome in a prebiotic RNA world would appear implausible, it is thus natural to ask how appreciable peptide formation could have occurred with such substrates in bulk water without the aid of advanced ribozymatic catalysis. Using a combination of fluorine-tagged aminoacyl adenylate esters, in situ monitoring by ^19^F{^1^H} NMR spectroscopy, analytical deconvolution of kinetics, pH–rate profile analysis, and temperature-dependence studies, we here explore the mechanistic landscape of indirect amidation, via transesterification and O-to-N rearrangement, as a highly efficient, alternative manifold for transpeptidation that may have served as a prelude to ribosomal peptide synthesis. Our results suggest a potentially overlooked role for those amino acids implicated by the cyanosulfidic reaction network with hydroxyl side chains (Ser and Thr), and they also help to resolve some outstanding ambiguities in the broader literature regarding studies of similar systems (e.g., aminolyzes with Tris buffer). The evolutionary implications of this mode of peptide synthesis and the involvement of a very specific subset of amino acids are discussed.

## Introduction

**Figure 1:**
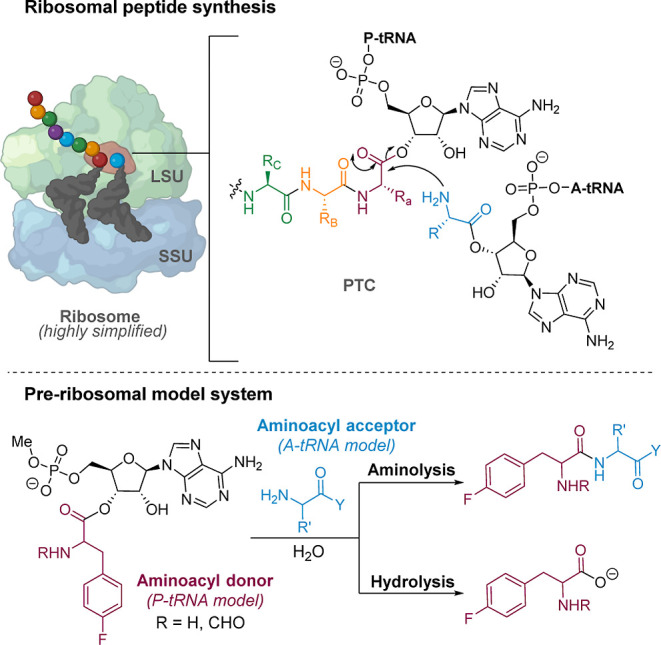
Underlying chemistry of ribosomal peptide synthesis.

The coded biosynthesis of proteins—the translation of genetic information into function—is a defining pillar of molecular biology and an apparent prerequisite for life. At the heart of the biochemical apparatus responsible for coordinating this synthesis in extant biology is the ribosome^1^—a macromolecular ensemble composed of both ribonucleic acid (RNA) and protein—and within the ribosome, the key site of synthetic chemical interest is the peptidyl transferase center (PTC).^2^ It is here that peptide bonds are forged, via the sequential cross-aminolysis of nucleophilic 3′-aminoacyl-tRNA esters and electrophilic 3′-*N*-acyl-aminoacyl-tRNA (peptidyl-tRNA) esters,^3^ and here where peptides are eventually released by the hydrolysis of the final peptidyl-tRNA ester (Figure 1). The secondary structure and primary sequence of the RNA surrounding the PTC appear to be highly conserved across every kingdom of life, and this, combined with suggestive symmetry and the absence of ribosomal protein in the immediate vicinity of the PTC, has led to the suggestion that the RNA surrounding the PTC may constitute a vestige of a primordial transpeptidation ribozyme. The recent work of Yonath and co-workers,^4^ demonstrating the possibility of peptide bond formation—albeit in trace amounts—mediated solely by fragments of this putative protoribosome,^4−6^ provides notable support for this hypothesis.

Unlike typical protein-based enzymes, the catalytic competency of the ribosome is predicated on its ability to serve as an “entropy trap”.^7,8^ It catalyzes peptide synthesis not by enthalpic transition state stabilization but by using the RNA surrounding the PTC to precisely align the two key substrates in a manner conducive to aminolysis and by providing a desolvated environment in which passage through charge-separated transition states (i.e., tetrahedral zwitterions) is not hampered by the need to reorganize solvent (water). Given that parsimony supports the principle of continuity, it would seem reasonable to expect a similar modus operandi of a protoribosome.^10^

The remarkable efficiency of ribosomal peptide synthesis, however, belies a significant problem for uncatalyzed or otherwise unassisted peptide synthesis under the same manifold: in water, it simply is not very good. For example, even at highly elevated concentrations (400 mM), the autoaminolysis of 2′(3′)-*O*-(glycyl)-adenosine-5′-(*O*-methylphosphate) (**MepA-Gly**) in water, Orgel’s chemical model for ribosomal peptide synthesis, is decidedly inefficient,^11a^ with the combined yield of all peptide-derived products not surpassing 17% even under optimal conditions. **MepA-Gly**, an alkyl ester activated by virtue of aminoacyl N*-*protonation and by a competent diol leaving group, undergoes highly efficient hydrolysis around neutral pH;^9^ it is this hydrolytic instability, combined with the significant entropic barriers associated with direct aminolysis in water, that conspires to suppress the yield of peptide. Other chemical models of ribosomal peptide synthesis have encountered the same problem time and again:^11−13^ for all but the most favorable or biased systems, outcompeting hydrolysis is hard, even at “the highest possible concentrations”.^13^

Given the physical basis of ribosomal activity, the woeful productivity of this chemistry in bulk aqueous solution would appear to pose an evolutionary dichotomy. On the one hand, one might conclude that the incipient stages of ribosomal peptide synthesis, based on the aminolysis of aminoacyl RNA esters, would have been too inefficient to convey any evolutionary advantage. In such a case, ribosomal peptide synthesis could only have emerged, somehow, after the independent development, for other reasons, of a ribozyme that fortuitously permitted the use of such substrates. An alternative conclusion might be that preribosomal peptide synthesis relied on fundamentally similar chemistry—and similar substrates—to that now used in biology but exploited some auxiliary mechanism, beyond direct amidation, to outcompete hydrolysis in the absence of ribozymatic assistance.

We were drawn to the second of these possibilities by the observations of Weber and Orgel^11b^ and the independent work of Jencks and co-workers.^14^ While the cross-aminolysis of **MepA-Gly** (400 mM) with equimolar glycine (Gly) was shown to be just as inefficient as autoaminolysis, Orgel reported that a reaction with l-serine (Ser) exhibited a drastically different profile: not only did Gly–Ser, the cross-aminolysis product, form efficiently, but its formation even outpaced **MepA-Gly** hydrolysis under the particular conditions studied. This finding, combined with Jencks’ observation of the enhanced nucleophilicity of serinyl hydroxyl groups,^14^ points to the existence of a more efficient, indirect mechanism for peptide formation proceeding via transesterification to the serine hydroxyl group and O-to-N rearrangement (cf. native chemical ligation),^15^ and thereby an opportunity for outcompeting hydrolysis in a preribosomal world. The proven capacity of the extant ribosome to facilitate transesterification with α-hydroxy analogues of aminoacyl tRNA esters,^8,16^ our finding that serine and threonine (but not cysteine) precursors emerge naturally, as part of a limited subset of amino acids, from the cyanosulfidic reaction network,^17^ and the reality that even the most fruitful demonstrations of protoribosomal activity have furnished little more than trace peptidation with nonhydroxylated aminoacyl acceptors,^4^ all suggest this mechanism could have evolutionary significance hitherto underappreciated.

On this basis and in light of the conflicting mechanisms proposed in the literature for such processes, we have sought to investigate, systematically and in quantitative detail, a chemical model system for ribosomal peptide formation. Our biomimetic system, designed to be amenable to in situ monitoring by ^1^H-decoupled ^19^F NMR spectroscopy (^19^F{^1^H}_IG_ NMR), encompasses a range of ^19^F-tagged aminoacyl adenylate esters as aminoacyl donors (P-tRNA analogues), differing in their degree of acylation, N-functionalization, and stereochemistry, as well as a range of hydroxyl-bearing amino acid derivatives, plus nonhydroxylated controls—as aminoacyl acceptors (A-tRNA analogues). Herein, we report the results of our investigation and discuss the possible evolutionary implications of a preribosomal dependence on indirect amidation.

Our study places earlier observations into an overarching framework; offers direct spectroscopic evidence and kinetic characterization of the elusive serinyl ester intermediate; resolves apparent incongruities between the related conclusions of Pinck and Schuber,^18,19^ Orgel and Weber,^11^ Wolfenden and co-workers,^7^ and others;^20,21^ and offers subtle mechanistic insights from studies of concentration, pH, and temperature dependence. We also discuss possible functions for the hydroxyl-functionalized peptides, or lack thereof, that one would presumably expect to emerge from a dependence on indirect amidation in a preribosomal world.

## Results

### Preliminary Experiments

Preliminary work focused on the design of a chemical model system for ribosomal peptide formation that was readily amenable to in situ monitoring under a wide range of temperatures and conditions, and which represented a reasonable compromise between synthetic accessibility and fidelity to the conserved CCA-3′ terminus of extant aminoacyl-tRNAs. Drawing on the substrates used in previous studies,^4,7,12,13,22,23^ we thus prepared 2′(3′)-*O*-(4-fluoro-l-phenylalanyl)-adenosine-5′-(*O*-methylphosphate) (**MepA-****l****-PheF**) by conventional synthesis, with an eye to deploying ^19^F{^1^H}_IG_ NMR for in situ monitoring.^24^ Initial experiments focused on **MepA-****l****-PheF** rather than any N-acylated derivatives, out of both practical expediency and a desire to establish a benchmark for assessing the perturbation brought about by *N*-acylation. The pure monoester **MepA-****l****-PheF** was isolated as a mixture of the 2′- and 3′-regioisomers by preparative reverse-phase HPLC (formic acid/acetonitrile; pH = 2.4) and lyophilization, and its identity was confirmed by standard NMR characterization and LC/ESI(+)-MS (*m*/*z* = 527.0 g mol^–1^; calcd 527.1 g mol^–1^). Preliminary ^19^F{^1^H}_IG_ NMR monitoring of **MepA-****l****-PheF** (4 mM) in D_2_O (pH* = 7.0; 20 °C) over time revealed two well-resolved, decaying singlets for the 2′- and 3′-regioisomers (Δδ_F_ ≈ 200 Hz), as well as a third, growing singlet attributed to, and subsequently confirmed as, 4-fluoro-l-phenylalanine (**l****-PheF-OH**). As expected, the ratio of the two regioisomers remained constant at 65:35 (3′:2′; *f*_{Em,3′}_ = 65%)^12,25,26^ throughout the course of hydrolysis, indicating that 2′,3′-aminoacyl exchange is rapid with respect to substrate hydrolysis but slow relative to Δδ_F_.^25^

To gauge the coarse efficiency of peptide formation in water with **MepA-****l****-PheF** as an aminoacyl donor, a range of independent reactions of **MepA-****l****-PheF** (2 mM)^27^ and various aminoacyl acceptors were run under buffered conditions in D_2_O (20 °C). The aminoacyl acceptors, either achiral or single enantiomers, were used in large excess (600 mM), and their nucleophilic forms were liberated with an appropriate amount of KOD to establish a buffer near the relevant p*K*_a_*. Under these conditions, and as per Orgel’s findings with **MepA-Gly**,^11b^ quantitative end-point ^19^F{^1^H}_IG_ NMR analysis revealed minimal cross-aminolysis (yield <5%) with even the least hindered aminoacyl acceptors, including glycine (Gly-OH; pH*(20 °C) = 10.3), glycinamide (Gly-NH_2_; pH*(20 °C) = 8.5), and l-alaninamide (l-Ala-NH_2_; pH*(20 °C) = 8.5). This was in stark contrast to the reactions with l-serine (l-Ser-OH; pH*(20 °C) = 9.7; 42%), with l-serinamide (l-Ser-NH_2_; pH*(20 °C) = 7.7; 55%), and with l-serine *tert*-butyl ester (l-Ser-O^*t*^Bu; pH*(20 °C) = 7.7; 55%), for which hydrolysis and aminolysis, strikingly, were almost equally competitive in each case. Significant, albeit lower, yields of amide were also observed following incubation with l-threoninamide (l-Thr-NH_2_; pH*(20 °C) = 7.4; 16%), but only one product—that of hydrolysis—was observed in solutions buffered with *O*-methyl-l-serine (pH*(20 °C) = 8.8) and *O*-phospho-l-serine (pH*(20 °C) = 9.8) (both O-blocked). Notably, incubation of **MepA-****l****-PheF** with a mixture of *N*-acetyl-l-serine (600 mM; N-blocked) and buffering glycinamide (100 mM; pH*(20 °C) = 7.6) also yielded significant quantities (27%) of a second product beyond **l****-PheF-OH**, which on the basis of its hydrolytic lability at higher pH (pH*(20 °C) > 9.5) was assigned as the transesterification product, *O*-(4-fluoro-l-phenylalanyl)-*N*-acetyl-l-serine.

Subtle modulations in these selectivities were observed in the reactions of various derivatives of **MepA-****l****-PheF**, all synthesized and incubated in an analogous manner, with a subset of aminoacyl acceptors (see Supporting Information). Compared to **MepA-****l****-PheF**, modest increases in the apparent predisposition to amidation, all other things being equal, were observed in the case of 2′(3′)-*O*-(*N*-formyl-4-fluoro-l-phenylalanyl)-adenosine-5′-(*O*-methylphosphate) (**MepA-***N*-f-l**-PheF**) and 2′,3′-bis-*O*-(4-fluoro-l-phenylalanyl)-adenosine-5′-(*O*-methylphosphate) (**MepA-L-(PheF)**_**2**_) and with d-serine versus l-serine, as well as at lower temperatures; minor reductions in selectivity for aminolysis were observed with the diastereomeric aminoacyl donor **MepA-****d****-PheF**.

### Kinetic Studies

Clearly, the presence or absence of an α-hydroxymethyl substituent in the aminoacyl acceptor exerts a profound effect on the efficiency of amide bond formation, and thereby the ability of such acceptors to compete as nucleophiles with water. This observation would appear to implicate a distinct, indirect mechanism for aminolysis, comprising O-aminoacylation followed by intramolecular O-to-N rearrangement, yet various studies of similar systems^18,21^ have discounted such a pathway.

With this ambiguity in mind, we thus decided to set out on a detailed kinetic study of the aminolysis of **MepA-****l****-PheF** (hereafter “**E**_**m**_”; [**E**_**m**_]_T,0_ = 4.0 mM)—and some key derivatives—using l-serinamide (^L^**S**) as a hydrolytically stable aminoacyl acceptor analogue.^28^ All reactions were monitored in situ by ^19^F{^1^H}_IG_ NMR under pseudo-first-order conditions, using a minimum of a 150-fold excess of total l-serinamide, [^**L**^**S**]_T_, and the ionic strength of all reactions was standardized to 2.0 M using KCl as an inert salt.^29a^ Free base l-serinamide, ^**L**^**S**^**0**^, was liberated from its hydrochloride salt via the addition of variable amounts of either KOH or KOD, depending upon whether aminolysis was monitored in H_2_O or D_2_O, and in all cases, sufficient base was used to ensure that the pH (H_2_O) or apparent pH* (D_2_O) varied by <0.1 units over the course of the reaction.^29b^^19^F{^1^H}_IG_ NMR spectra were acquired identically in all cases, using parameters that allowed the relative quantitation of all key species to within <5%, and all reactions were monitored to a minimum substrate conversion of 75% (i.e., two half-lives).

**Figure 2:**
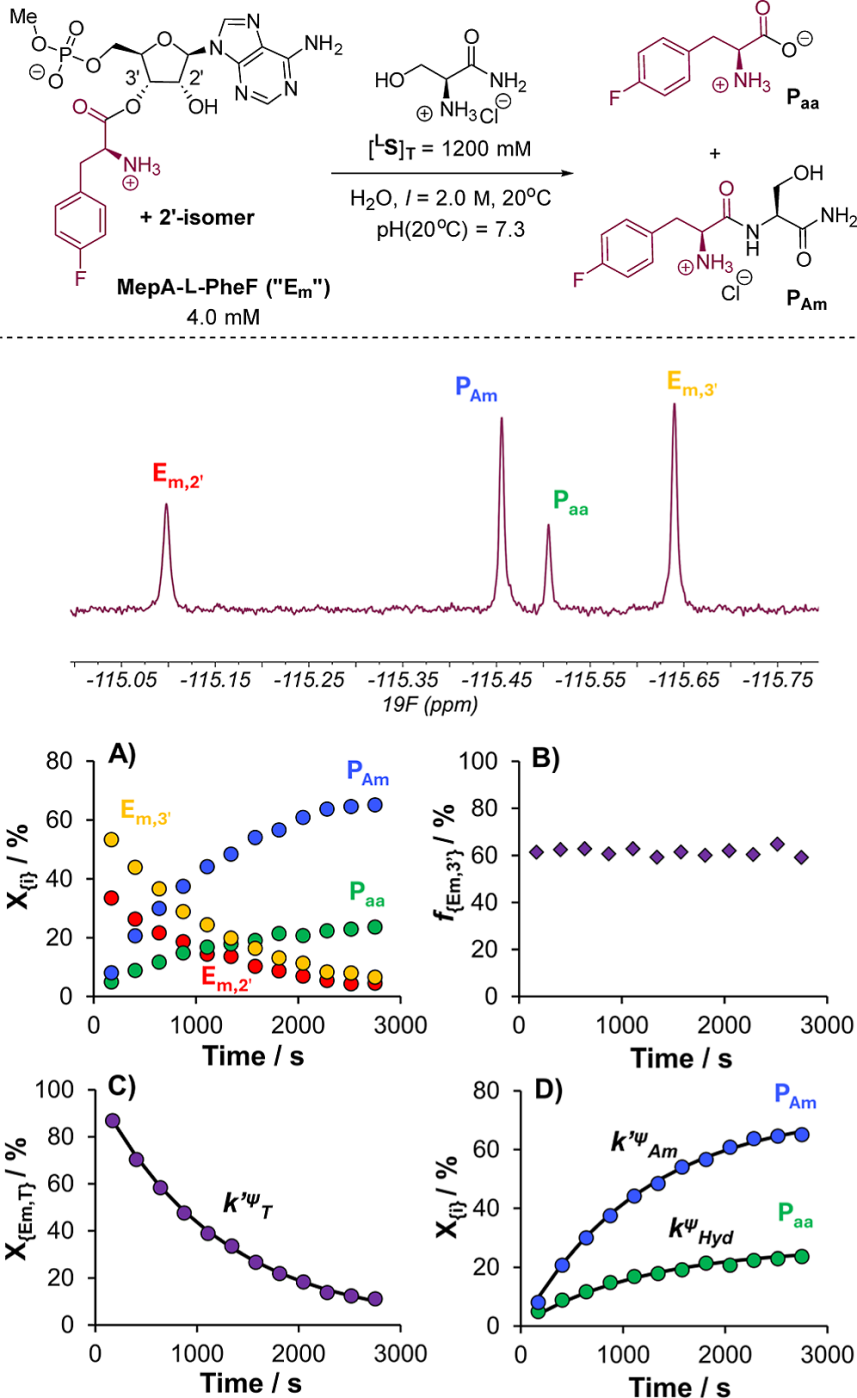
Chemical scheme, example ^19^F{^1^H}_IG_ NMR spectrum (376 MHz), and key temporal evolution profiles from the monitoring of a typical aminolysis of **MepA-****l****-PheF** (**E**_**m**_) with l-serinamide ^**L**^**S** in aqueous solution (this example: H_2_O, 20 °C, pH = 7.3, *I* = 2.0 M). (A) Temporal evolution of experimental mole fractions of **E**_**m,2′**_, **E**_**m,3′**_, **P**_**Am**_, and **P**_**aa**_. (B) Temporal evolution of experimentally observed 2′/3′ speciation of **E**_**m**_, with *f*_{Em,3′}_ = [**E**_**m,3**′_]/[**E**_**m**_]_T_ = fractional population of the 3′-isomer of **E**_**m**_ over all charge states. (C) Temporal evolution of the mole fraction of **E**_**m,T**_ with fit to the kinetic model: **E**_**m,T**_ → **P**_**Am**_ (*k*_Am_^ψ^), **E**_**m,T**_ → **P**_**aa**_ (*k*_Hyd_^ψ^). (D) Temporal evolution of the mole fractions of **P**_**Am**_ and **P**_**aa**_ with fit to the kinetic model above. [**E**_**m**_]_T_ = [**E**_**m,2**′_] + [**E**_**m,3**′_] = total concentration of **E**_**m**_ in all charge states. *k*_T_^ψ^ = total pseudo-first-order rate constant for the decay of **E**_**m,T**_. *k*_Am_^ψ^ = *f*_{Am}_.*k*_T_^ψ^ = *k*_Am_′_._[^**L**^**S**]_T_ = pseudo-first-order rate constant for the aminolysis of **E**_**m,T**_. *k*_Am_′ = total second-order rate constant for the aminolysis of **E**_**m,T**_. *k*_Hyd_^ψ^ = *f*_{Hyd}_. *k*_T_^ψ^ = pseudo-first-order rate constant for the hydrolysis of **E**_**m,T**_. [**E**_**m,j′**_] = [**E**_**m,j′**_^**(0)**^] + [**E**_**m,j′**_^**(+)**^], where [**E**_**m,j′**_^**(i)**^] = concentration of j′-regioisomer with a charge of i on the α-amino group. X_{*i*}_ = mole fraction of species *i*.

Under near neutral and moderately alkaline conditions (pH*(20 °C) ≈ 6.5–9.2), we found that the monoexponential decay of the aminoacyl-monoester **E**_**m**_ was accompanied by the parallel, monoexponential formation of two stable products (Figure 2): l**-PheF-OH** (“**P**_**aa**_”), the result of substrate hydrolysis, and *N*-(4-fluoro-l-phenylalanyl)-l-serinamide (**P**_**Am**_), the amide product. This is consistent with the observation of Wolfenden and others in related systems.^7,13^ A typical set of reaction profiles is shown in Figure 2, alongside an example ^19^F{^1^H}_IG_ NMR spectrum acquired during in situ monitoring. No systematic drift was observed in the 2′/3′ speciation of the monoester, *f*_{Em,3′}_, in this example or within any other run, and therefore, pseudo first-order rate constants (generally, *k*^ψ^) for hydrolysis (*k*_Hyd_^ψ^) and aminolysis (*k*_Am_^ψ^) were determined by fitting the experimental mole fractions^29c^ of the total monoester (2′ + 3′), **E**_**m,T**_, as well as **P**_**aa**_ and **P**_**Am**_ to the equations
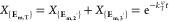
1
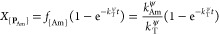
2
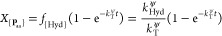
3
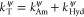
4

**Figure 3:**
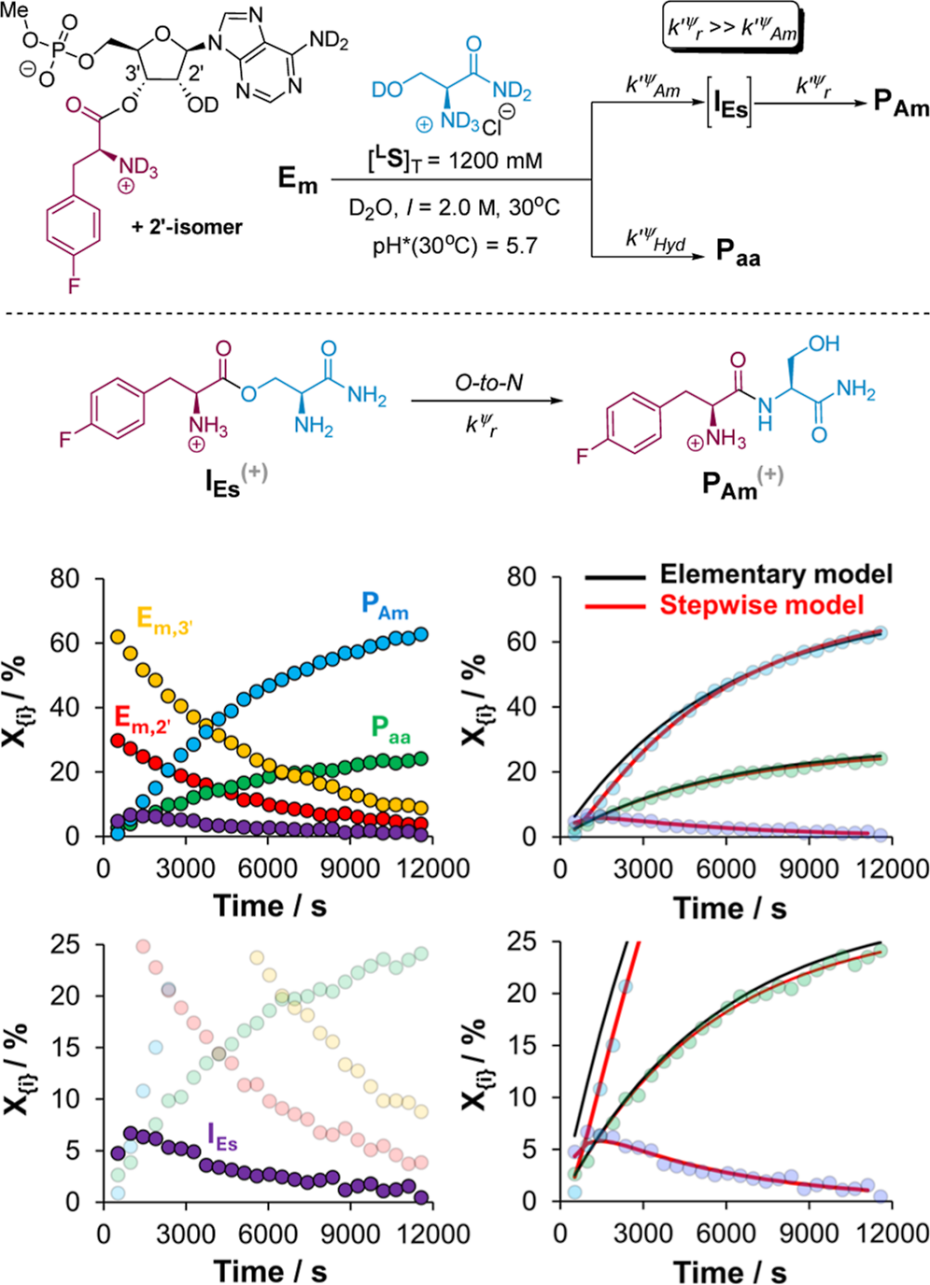
Kinetic scheme, temporal evolution profiles, and comparative fits to two distinct kinetic models for key species in a typical aminolysis of **MepA-****l****-PheF** (**E**_**m**_) with l-serinamide under a low pH regime (this example: D_2_O, 30 °C, pH*(30 °C) = 5.7, *I* = 2.0 M). Elementary model = **E**_**m,T**_ → **P**_**Am**_ (*k*_Am_^ψ^), **E**_**m,T**_ → **P**_**aa**_ (*k*_Hyd_^ψ^). Stepwise model: = **E**_**m,T**_ → **I**_**Es**_ (*k*_Am_^ψ^), **I**_**Es**_ → **P**_**Am**_ (*k*_r_^ψ^), **E**_**m,T**_ → **P**_**aa**_ (*k*_Hyd_^ψ^).

More complex reaction profiles were observed under mildly acidic conditions (pH* < 6.5), with the emergence of an intermediate species and subtly sinusoidal behavior in the temporal evolution of the aminolysis product **P**_**Am**_ (Figure 3). The detection of the intermediate under such conditions was reproducible across different batches of **E**_**m**_, in H_2_O and D_2_O, and at different temperatures, vide infra, and global fitting of experimental populations to an analytical kinetic model (eqs 2, 3, 5, and 6) in which the intermediate derives from **E**_**m**_ and decomposes to **P**_**Am**_ gave materially improved kinetic fits relative to a one-step model.
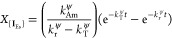
5
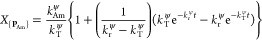
6

The kinetically implied provenance and fate of the intermediate, its greater accumulation at lower pH, and the relative stability of the transesterification product of **E**_**m**_ and *N*-acetyl-l-serine at pH*(20 °C) ≈ 7.5, vide supra, lead us to assign this species as the previously elusive serinyl ester intermediate, in this case *O*-(4-fluoro-l-phenylalanyl)-l-serinamide (**I**_**Es**_).

Under the assumption of this assignment, the rate-determining step in the aminolysis **E**_**m**_ with l-serinamide must be interpreted as being O*-*acylation, such that the pseudo-first-order rate constant *k*_Am_^ψ^ actually reflects the rate of the initial transesterification; *k*_r_^ψ^, measurable only at sufficiently low pH, then reflects the rate of the relatively rapid, intramolecular O-to-N rearrangement. This assignment is consistent with many,^20,30^ though not all,^18,21^ studies of the reactions of tris(hydroxymethyl)aminomethane (**Tris**) with various activated esters; it is also strongly indicated by the studies of Hansen,^31^ and Porter and co-workers,^32^ on the intramolecular O-to-N rearrangement of *O*-acetyl-ethanolamine in aqueous solution and that of Phillips and Baltzly on the rearrangement of analogous esters of ethanolamine.^33^

**Figure 4:**
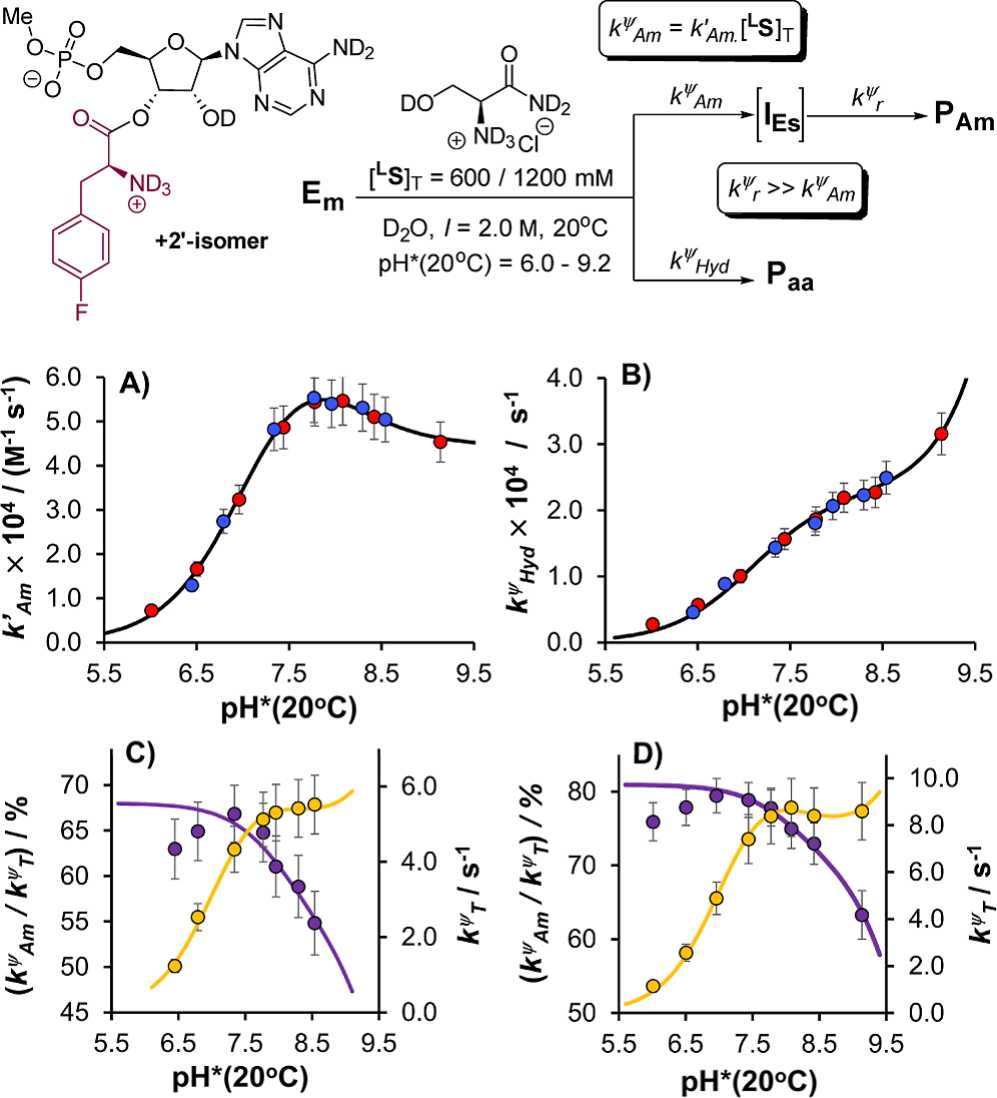
Kinetic analysis of the aminolysis of **E**_**m**_ with l-serinamide ^**L**^**S**, and concurrent hydrolysis, over the range pH* = 5.7–9.3 (20 °C, D_2_O, *I* = 2.0 M, KCl; [**E**_**m**_]_T,0_ = 4.0 mM). (A) pH*–*k*′_Am_ profile for [^**L**^**S**]_T_ = 600 mM (blue) and 1200 mM (red), with fit to experimental data. (B) pH*–*k*_Hyd_^ψ^ profile, with same color coding as (A), and fit to experimental data. (C) Empirical selectivity for aminolysis, *k*_Am_^ψ^/*k*_T_^ψ^, and total pseudo-first-order rate of decay *k*_T_^ψ^ as a function of pH*(20 °C), with fits to experimental data, for [^**L**^**S**]_T_ = 600 mM. (D) as for (C) but with [^**L**^**S**]_T_ = 1200 mM. Fitted parameters (*k*_Am,+_, *k*_Am,0_, *k*_OH^–^,+_, *k*_OH^–^,0_; p*K*_a_*(**E**_**m**_^**+**^)) and their standard errors (Table S1, Supporting Information) were determined by global, unweighted nonlinear fitting of the pH*–*k*′_Am_ and pH*–*k*_Hyd_^ψ^ profiles (OriginPro 2024b). Relative uncertainties in *k*_Hyd_^ψ^ and *k*′_Am_ were conservatively estimated to be ±10% (see Supporting Information). Uncertainties in *k*_T_^ψ^ and *k*_Am_^ψ^/*k*_T_^ψ^ (not fitted directly) were calculated by propagation. See Supporting Information for further discussion.

With a means of monitoring and analytical kinetic deconvolution in place, the aminolysis—and concurrent hydrolysis—of **E**_**m**_ with l-serinamide in D_2_O (20 °C) was next studied systematically across the range pH*(20 °C) = 6.0–9.3 (*I* = 2.0 M) (Figure 4). Two series of reactions, with different total l-serinamide concentrations [^L^**S**]_T_ = 600 or 1200 mM, were conducted, with the pH* of each reaction adjusted by the addition of variable quantities of KOD to l-serinamide deuterochloride. The resultant pH*–rate profiles (pH*–*k*_Am_′, pH*–*k*_Hyd_^ψ^; *k*_Am_′ = *k*_Am_^ψ^/[^**L**^**S**]_T_) are shown in Figure 4. In accordance with Wolfenden’s study with **Tris**(7a)—but in contrast to a second study with glycinamide^7b^—the fact that data from both series of experiments fall on the same profiles suggests that (i) the rate of transesterification, and therefore overall aminolysis, is first-order with respect to [^**L**^**S**]_T_ and (ii) the rate of hydrolysis is independent of [^**L**^**S**]_T_. This appears to rule out significant general-base catalysis by l-serinamide, of either transesterification or hydrolysis, under these conditions.^34^

A satisfactory fit of the pH*–*k*′_Am_ profile was obtained by assuming that *k*′_Am_ comprises only two terms, including rates for (i) the transesterification (formal aminolysis) of the aminoacyl ester (**E**_**m**_) in its N-protonated state, **E**_**m**_^**+**^ (*k*_Am,+_), and (ii) the transesterification (formal aminolysis) of the free base aminoacyl ester, **E**_**m**_^**0**^ (*k*_Am,0_). It did not seem necessary to include any additional terms for, e.g., the specific-base-catalyzed reaction of l-serinamide with either **E**_**m**_^**0**^ or **E**_**m**_^**+**^.^35^ The second-order rate constants *k*_Am,+_ and *k*_Am,0_ are weighted averages of the corresponding constants for the 2′ - and 3′-regioisomers since [**E**_**m**_^**i**^] = [**E**_**m,2′**_^**i**^] + [**E**_**m,3′**_^**i**^] (*i* = 0 or +).

Assuming that only the free base state of l-serinamide, ^**L**^**S**^**0**^, can undergo amidation, this means that
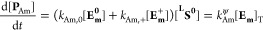
7
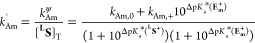
8
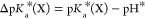
9

The underlying second-order kinetic parameters, *k*_Am,+_ and *k*_Am,0_, and the apparent p*K*_a_* of **E**_**m**_^**+**^ were determined^36^ by nonlinear fitting (unweighted)^37a,37b^ of *k*_Am_′ to eq 8, with the apparent p*K*_aH_* of l-serinamide, ^**L**^**S**, measured independently by ^13^C{^1^H} NMR titration under equivalent conditions (D_2_O, 20 °C, *I* = 2.0 M), constrained during fitting (p*K*_aH_*(^**L**^**S**; 20 °C) = 7.94).

**Figure 5:**
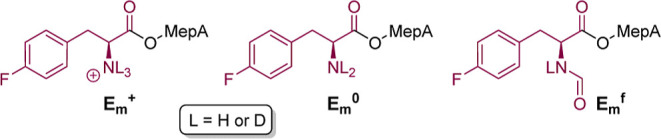
Simplified structural depictions of **E**_**m**_^**+**^ and **E**_**m**_^**0**^ and the N-formylated derivative **E**_**m**_^**f**^. Note that 2′–3′ isomerization (not shown) appears to be limitingly rapid for both **E**_**m**_ and **E**_**m**_^**f**^, relative to hydrolysis and aminolysis (pH* > 5.5), and so, all rate constants and isotope effects in Tables 1 and 2 are weighted averages. See Supporting Information for details.

An analogous process was followed for the pH*–*k*_Hyd_^ψ^ profile, with two terms required to obtain a satisfactory fit to the experimental data, including rates for (*L* = *H*/*D*) (i) the saponification of **E**_**m**_^+^ (*k*_OL^–^,+_) and (ii) the saponification of **E**_**m**_^**0**^ (*k*_OL^–^,0_).^36,37^ The underlying kinetic parameters were again determined by nonlinear fitting (unweighted)^37a,37b^ to the appropriate equations, with the p*K*_a_* of **E**_**m**_^**+**^ obtained by global fitting of a common value to both the pH*–*k*′_Am_ and pH*–*k*_Hyd_^ψ^ profiles.^38^ The apparent self-ionization constants of H_2_O and D_2_O, on the molarity scale, p*K*_w_*, were approximated from the thermodynamic values reported by Covington and co-workers,^39^ with the activity coefficient for the hydroxide/deuteroxide anion γ_OH^–^_ = γ_OD–_ = 0.7 approximated from Harned.^40^
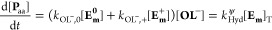
10
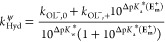
11
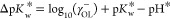
12In coarse terms, the shape of the pH*–*k*_Hyd_^ψ^ profile (Figure 4b) is consistent with that reported by Wolfenden for single-stranded leucyl-RNA^9^ and reflects the combined influence of pH* upon the concentration of hydroxide (with [OH^–^] ∝ 10^pH*^) and the speciation of **E**_**m**_ (Figure 5), as well as the different reactivities of **E**_**m**_^**0**^ (i.e., *k*_OL^–^,0_) and **E**_**m**_^**+**^ (i.e., *k*_OL^–^,+_). The factors governing the pH*–*k*′_Am_ profile (Figure 4a) are ultimately very similar, but the shape, featuring a maximum around pH*(20 °C) ≈ 8, is clearly distinct. This arises from the fact that the concentration of free base l-serinamide (^**L**^**S**^**0**^)—the reactive nucleophile in aminolysis—saturates (i.e., [^**L**^**S**^**0**^] → [^**L**^**S**]_T_) over the pH* range in Figure 4 in a way that the hydroxide concentration does not. While increasing the pH* leads to higher concentrations of the active nucleophile in both reactions, it also shifts the speciation of the monoester toward its less electrophilic form, **E**_**m**_^**0**^. The compensation of these two factors means that the changes in the observed rates of hydrolysis and aminolysis (Figure 4; *k*_Hyd_^ψ^, *k*′_Am_) do not fully reflect, at least visually, the intrinsic reactivity differences of **E**_**m**_^**0**^ and **E**_**m**_^**+**^, thus requiring mathematical deconvolution.

The empirical selectivity for aminolysis, *k*_Am_^ψ^/*k*_T_^ψ^, is dependent on [^**L**^**S**]_T_, but for both [^**L**^**S**]_T_ = 600 and 1200 mM, it appears to peak around pH*(20 °C) ≈ 7.0–7.5. The significant drop in selectivity at higher pH* is a direct consequence of the saturation in [^**L**^**S**^**0**^], and this is captured fully by our analysis; the more subtle drop in selectivity at lower pH*, however, is not captured. We tentatively ascribe this to the (necessary)^37a^ exclusion of the hydrolysis of **I**_**Es**_ to **P**_**aa**_ as a pathway from the underlying kinetic model (Figure 4, top). Such a pathway will be negligible above pH*(20 °C) ≈ 7.5 but may become significant at lower pH* on account of the increasing degree of protonation of the α-amino group in **I**_**Es**_, which will simultaneously preclude O-to-N rearrangement and activate the intermediate to hydrolysis.^37b^

The intrinsic reactivities of **E**_**m**_^**0**^ and **E**_**m**_^**+**^ toward both aminolysis and hydrolysis, in the form of second-order rate constants, are summarized in Tables 1 and 2. A simple but important observation from this data pertains to the relative reactivities of water, hydroxide, and free base l-serinamide toward **E**_**m**_^**+**^: l-serinamide is 6 orders of magnitude less reactive than hydroxide but at least 4 orders of magnitude more reactive than water itself (*k*_D_2_O,+_ < 2 × 10^–7^ M^–1^ s^–1^; [D_2_O] = 55 M).^37c^ Given that no kinetic term for the reaction of the l-serinamide alkoxide anion was required to reproduce the experimental pH*–*k*′_Am_ profile, this trend suggests that transesterification with neutral l-serinamide ought to involve intramolecular general base catalysis by its α-amino group,^20^ though it cannot in itself give any indication as to the extent of proton transfer in the transition state. Some kind of qualitative constraint on this front may, though, be placed by noting that the protonation of the α-amino group in the aminoacyl ester accelerates saponification (e.g., *k*_OD^−^,+_/*k*_OD^−^,0_ = 274; D_2_O, 20 °C) to a far more significant degree than transesterification (e.g., *k*_Am,+_/*k*_Am,0_ = 13; D_2_O, 20 °C).^41^ This in turn results in a precipitous drop in the intrinsic selectivity (*k*_OH^–^,+_/*k*_Am,+_ = 9.9 × 10^5^; *k*_OH^–^,0_/*k*_Am,0_ = 4.7 × 10^4^) for transesterification over saponification.^42^ In the hypothetical limit of full intramolecular proton transfer in the transition state—i.e., if one imagines the reaction of the zwitterionic tautomer of l-serinamide—it might be expected that protonation of the aminoacyl ester would accelerate this reaction similarly to its reaction with hydroxide. That this is not observed suggests, in turn, that the charge distribution in the transition state for transesterification is quite distinct from saponification for either one, or both, of **E**_**m**_^**0**^ or **E**_**m**_^**+**^. Quantum chemical computations and hypothetical kinetic analysis appear to support this line of argument,^43,43a^ but we cannot definitively rule out the direct reaction of the l-serinamide zwitterion in all contexts—especially in light of the dynamic subtleties that ought to be considered in distinguishing specific- and intramolecular general-base-catalyzed processes.^43b^

**Table 1:** Kinetic Solvent Isotope Effects (*k*_H_/*k*_D_)_*i*_ for the Saponification and Aminolysis of **E**_**m**_ with l-Serinamide^a^

*i*	*k*_i_/(M^–1^ s^–1^) (H_2_O)	*k*_i_/(M^–1^ s^–1^) (D_2_O)	(*k*_H_*/k*_D_)_*i*_
Am, +	4.6(3) × 10^–3^	5.8(2) × 10^–3^	0.80(6)
Am, 0	4.9(1) × 10^–4^	4.5(1) × 10^–4^	1.10(3)
OL^–^, +	3.2(3) × 10^3^	5.7(2) × 10^3^	0.56(6)
OL^–^, 0	10(3)	21(2)	0.5(1)
Am, f	4.1(2)×10^–4^	3.3(2) × 10^–4^	1.2(1)
OL^–^, f	1.9(1) × 10^2^	2.3(1) × 10^2^	0.83(4)

a (*k*_H_/*k*_D_)_i_ corresponds to the solvent (H_2_O/D_2_O) kinetic isotope effect for the second-order rate constant *k*_*i*_. Uncertainties in (*k*_H_/*k*_D_)_i_ determined by propagation of standard errors in the individual rate constants *k*_i_. All uncertainties and quoted in brackets (e.g., 1.0(2) = 1.0 ± 0.2). All kinetic isotope effects measured at 20 °C with *I* = 2.0 M (KCl).

**Table 2:** Kinetic and Acidity Parameters for the Aminolysis and Hydrolysis of **E**_**m**_ with l-Serinamide^a^

	10 °C	20 °C	30 °C
p*K*_a_*(^**L**^**S**^+^)	8.17(5)	7.94(5)	7.69(5)
*(p*K*_a_^D^)*	*(8.58(5))*	*(8.35(5))*	*(8.10(5))*
p*K*_a_*(**E**_**m**_^**+**^)	7.40(5)	7.08(2)	6.79(3)
*(p*K*_a_^D^)*	*(7.81(5))*	*(7.49(2))*	*(7.20(3))*
p*K*_w_*	15.03	14.64	14.29
*(p*K*_w_^D^)*	*(15.44)*	*(15.05)*	*(14.70)*
			
*k*_Am,+_	2.6(2) × 10^–3^	5.8(2) × 10^–3^	1.0(1) × 10^–2^
*k*_Am,0_	2.5(1) × 10^–4^	4.5(1) × 10^–4^	7.8(1) × 10^–4^
*k*_OL^–^,+_	2.9(3) × 10^3^	5.7(2) × 10^3^	1.0(6) × 10^4^
*k*_OL^–^,0_	11(3)	21(2)	35(3)
*k*_Am,f_		3.3(2) × 10^–4^	5.8(3) × 10^–4^
*k*_OH^–^,f_		2.3(1) × 10^2^	4.2(2) × 10^2^

a Second-order rate constants (*k*; M^–1^ s^–1^) and p*K*_a_*(**E**_**m**_^**+**^) values were obtained by global nonlinear fitting (unweighted)^37b^ of experimental pH*–*k*′_Am_ and pH*–*k*_Hyd_^ψ^ profiles (D_2_O, *I* = 2.0 M) to eqs 8 and 11; standard errors of fitted parameters were determined with OriginPro 2024b unless otherwise stated and quoted in brackets (e.g., 1.0(2) = 1.0 ± 0.2). p*K*_a_*(^**L**^**S**^**+**^) was determined independently by ^13^C{^1^H} NMR titrations with estimated uncertainties of ±0.05 units at all temperatures. All p*K*_a_* values are apparent/empirical and not true thermodynamic values, with pD = pH* + 0.408.^7b^ Apparent autoprotolysis constants in D_2_O, p*K*_w_*, were taken from Covington et al.^39^ See Supporting Information for details.

N-Formylation of the aminoacyl ester^44^ has an interesting effect: it accelerates saponification significantly, relative to **E**_**m**_^**0**^, but modestly suppresses transesterification, with the upshot that the intrinsic selectivity for amide bond formation (*k*_OH^–^,f_/*k*_Am,f_ = 7.0 × 10^5^) is very similar, though still superior, to the protonated ester, **E**_**m**_^**+**^. That the intrinsic selectivity and hydrolytic lability of **E**_**m**_^**f**^ is intermediate to **E**_**m**_^**0**^ and **E**_**m**_^**+**^ would appear to discount 5(4*H*)-oxazolones as intermediates in the transesterification and hydrolysis of the former, so the influence of N-formylation must represent a substituent perturbation on otherwise analogous transition states.^46^

**Figure 6:**
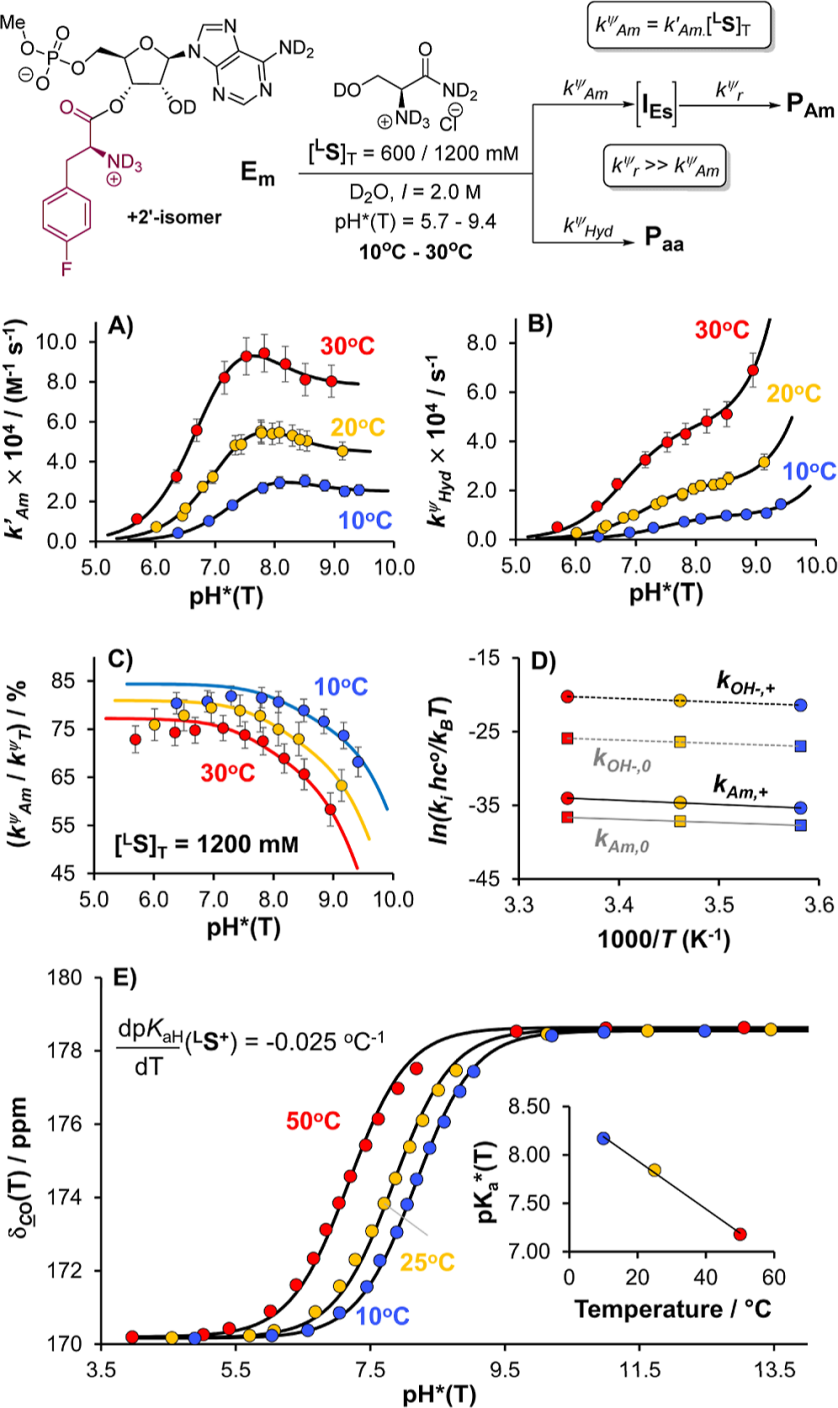
Temperature dependence (10–30 °C) of the hydrolysis and aminolysis of **E**_**m**_ with l-serinamide ^**L**^**S**, as a function of pH* (D_2_O, *I* = 2.0 M, KCl, [**E**_**m**_]_T,0_ ≈ 4 mM). (A) Temperature dependence of the pH*–*k*′_Am_ profile (aminolysis), with (unweighted) fits to experimental data. (B) Temperature dependence of the pH*–*k*_Hyd_^ψ^ profile (hydrolysis), with (unweighted) fits to experimental data. (C) Temperature and pH* dependence of (i) the empirical selectivity for aminolysis *k*_Am_^ψ^/*k*_T_^ψ^ and (ii) the total pseudo-first-order rate of decay *k*_T_^ψ^, with fits to experimental data, for [^**L**^**S**]_T_ = 1200 mM. (D) Linearized Eyring plots of the second-order rate constants *k*_Am,+_, *k*_Am,0_, *k*_OH^–^,+_, and *k*_OH^–^,0_. (E) ^13^C{^1^H} NMR titrations (δ_13CO_) of l-serinamide (100 mM; D_2_O, *I* = 2.0 M, KCl) at different temperatures ([dp*K*_aH_/d*T*] = −0.025 °C^–1^; cf. glycinamide^45^). pH*(10 °C) = 6.38–9.42 (p*K*_a_*(^**L**^**S**^**+**^) = 8.17). pH*(20 °C) = 6.01–9.14 (p*K*_a_*(^**L**^**S**^**+**^) = 7.94). pH*(30 °C) = 5.69–8.95 (p*K*_a_*(^**L**^**S**^**+**^) = 7.69). [^**L**^**S**]_T_ = 600–2000 mM. Fitted parameters (*k*_Am,+_, *k*_Am,0_, *k*_OH^–^,+_, *k*_OH^–^,0_; p*K*_a_*(**E**_**m**_^**+**^)) and their standard errors (Table 1, Supporting Information) were determined by global, unweighted^37b^ nonlinear fitting of pH*–*k*′_Am_ and pH*–*k*_Hyd_^ψ^ (OriginPro 2024b). See the Figure 4 caption and Supporting Information for details of error analysis.

Further pH*–*k*′_Am_ and pH*–*k*_Hyd_^ψ^ profiles were subsequently measured in H_2_O (20 °C) and at two additional temperatures in D_2_O (10 °C and 30 °C). These profiles (Figure 6) were analogous to those acquired in D_2_O at 20 °C, with all pH*–*k*_Hyd_^ψ^ profiles exhibiting inflections around p*K*_a_(**E**_**m**_^**+**^), all pH*–*k*′_Am_ profiles exhibiting maxima around p*K*_a_(^**L**^**S**^**+**^), and the aminolysis selectivities deteriorating subtly at lower pH* in a manner not fully captured by our deconvolution.^37^

As before, the intrinsic reactivities of **E**_**m**_^**0**^ and **E**_**m**_^**+**^ toward aminolysis and hydrolysis, in the form of second-order rate constants, were extracted by nonlinear fitting of the appropriate pH*–*k*^ψ^ profiles (Tables 1 and 2).

For a given temperature and total loading of l-serinamide, the maximal yield of amidation, generally around pH^(^*^)^ = p*K*_a_^(^*^)^(**E**_**m**_^**+**^), was found to increase slightly in D_2_O (e.g., 20 °C, [^**L**^**S**]_T_ = 1.2 M: 80%) compared to H_2_O (75%). The data in Tables 1 and 2 suggest that this is the result of various underlying solvent isotope effects. Significant inverse solvent isotope effects were observed for the saponification of both the protonated ((*k*_H_*/k*_D_)_OL^–^,+_ = 0.56(6)) and free base ((*k*_H_*/k*_D_)_OL^–^,0_ = 0.5(1)) aminoacyl monoesters; an attenuated, but nevertheless still inverse, effect was also observed for the saponification of the N-formylated derivative ((*k*_H_*/k*_D_)_OH^–^,f_ = 0.83(4)).^47^ The value for the *N*-formyl-aminoacyl ester is highly typical of effects seen in the saponification of classic carboxylic esters^48,49^ and may be the result of either rate-determining direct hydroxide attack or attack by water with general-base assistance by hydroxide.^50^ The more inverse effects for the free aminoacyl ester, in either ionization state, must reflect an even more pronounced tightening of hydrogenic sites in the transition state;^51^ though the basis for this is unclear, it is noteworthy that these values match closely the solvent effect observed for the specific-base-catalyzed reaction of pentaerythritol with *p*-nitrophenyl acetate (*k*_H_*/k*_D_ = 0.55).^21^

More interesting are the disparate solvent kinetic isotope effects observed for the transesterification of the free base, protonated, and *N*-formylated esters, with small normal effects observed for **E**_**m**_^**0**^ and **E**_**m**_^**f**^ but an inverse effect found for **E**_**m**_^**+**^.^52^ That the effects for **E**_**m**_^**+**^ and **E**_**m**_^**0**^ are so similar for saponification, but in opposite directions for transesterification, would appear to be diagnostic of some significant change in the nature of the transition state for transesterification in **E**_**m**_^**+**^. One interpretation would be that the inverse effect for **E**_**m**_^**+**^ reflects material alkoxide character in the transition state for transesterification, while the small normal effects for **E**_**m**_^**0**^ and **E**_**m**_^**f**^ reflect a transition state with less extensive O–H cleavage; this is understandable on the basis that greater alkoxide character in the l-serinamide hydroxyl group would benefit from both electrostatic and hydrogen-bond stabilization by an NH_3_^+^ substituent.

The temperature dependence of the various kinetic parameters offers further insight. Higher temperatures, for example, appear to enhance the activating influence brought about by the protonation of the α-amino group in the aminoacyl ester—both *k*_Am,+_/*k*_Am,0_ and *k*_OH^–^,+_/*k*_OH^–^,0_ increase with temperature—but they tend to leave the intrinsic selectivity for transesterification (*k*_OH^–^_/*k*_Am_) unaffected for all esters (**E**_**m**_^**0**^, **E**_**m**_^**+**^, and **E**_**m**_^**f**^). Another key observation is that the activation parameters (Table 3) for the aminolysis of both **E**_**m**_^**0**^ and **E**_**m**_^**f**^ match almost quantitatively, in both their enthalpic–entropic partition and absolute values, those measured by Wolfenden for the reaction of **Tris** with the ethylene glycol ester of *N*-formyl glycine (**f-Gly-Glycol**).^7a^ The correspondence of these activation parameters, the equivalent first-order dependence on the nucleophile, and our in situ detection and kinetic characterization of **I**_**Es**_ with l-serinamide, all point to a unifying mechanism of rate-determining transesterification and rapid O-to-N rearrangement. This supports Orgel’s hypothesis,^11b^ and the findings of Jencks and Carriuolo^20^ and de Jersey et al.^30^ in related systems, but differs from the conclusions of Schuber and Pinck^18^ and those of Bruice.^21^ For both saponification and transesterification, protonation of the aminoacyl ester leads to significant enthalpic destabilization and a compensatory entropic stabilization of the transition state with a net stabilization under ambient conditions.

**Table 3:** Transition State Activation Parameters for the Aminolysis and Hydrolysis of **E**_**m**_ and **E**_**m**_^**f**^ with l-Serinamide (D_2_O, *I* = 2.0 M, 10–40 °C)^a^

*k*_i_/(M^–1^ s^–1^)	Δ^‡^*H* (kJ mol^–1^)	Δ^‡^*S* (J K^–1^ mol^–1^)	–(*T*Δ^‡^*S*)_293K_ (kJ mol^–1^)	Δ^‡^*G*_293K_ (kJ mol^–1^)
*k*_Am,+_	47 ± 4	–127 ± 12	37 ± 4	84 ± 5
*k*_Am,0_	38 ± 1	–179 ± 2	52 ± 1	91 ± 1
*k*_OD^–^,+_	42 ± 1	–31 ± 3	9 ± 1	51 ± 1
*k*_OD^–^,0_	38 ± 1	–91 ± 4	27 ± 1	64 ± 2
*k*_Am,f_	36 ± 2	–188 ± 6	55 ± 2	91 ± 3
*k*_OL^–^,f_	38 ± 1	–67 ± 4	20 ± 1	58 ± 2
*k*_Tris_^‡^	38	–184	54	92
*k*_Gly-ND2_^†^	33	–220	65	97

a All activation parameters obtained by standard Eyring analysis, i.e., linear regression of ln(*k*_i_*hc*°/k_B_T) vs 1000/T, with *c*° = 1 M. Standard errors in Δ^‡^*H* and Δ^‡^*S* (shown) were propagated to estimate the uncertainty in Δ^‡^*G*_293K_. ^‡^Wolfenden’s study on the reaction of Tris with *N*-formyl-glycine glycol ester (**f-Gly-Glycol**). ^†^A more recent study on the reaction of glycinamide (Gly-ND_2_) with *N*-formyl-phenylalanine trifluoroethyl ester (f-Phe-TFE).

Inspired by the observations of Hecht on tandemly activated tRNAs^23,53^—their ability to serve as substrates in ribosomal synthesis, and their purportedly unusual stability to hydrolysis and aminolysis—we also studied the reaction of the bis-aminoacylated ester **MepA-(****l****-PheF)**_**2**_ (**E**_**bis**_) with l-serinamide over the range pH* = 5.6–8.6 (D_2_O, 20 °C, *I* = 2.0 M). This species was invariably formed during our attempts to prepare **E**_**m**_, by conventional synthesis, from **MepA** and equimolar **l****-PheF-OH** (activated with CDI), suggesting that its formation is predisposed and thus is of possible significance in the context of protoribosomal transpeptidation. All reactions, as before, were monitored over time by quantitative in situ ^19^F{^1^H}_IG_ NMR, and the kinetics were deconvoluted by analytical means (see Supporting Information).

**Figure 7:**
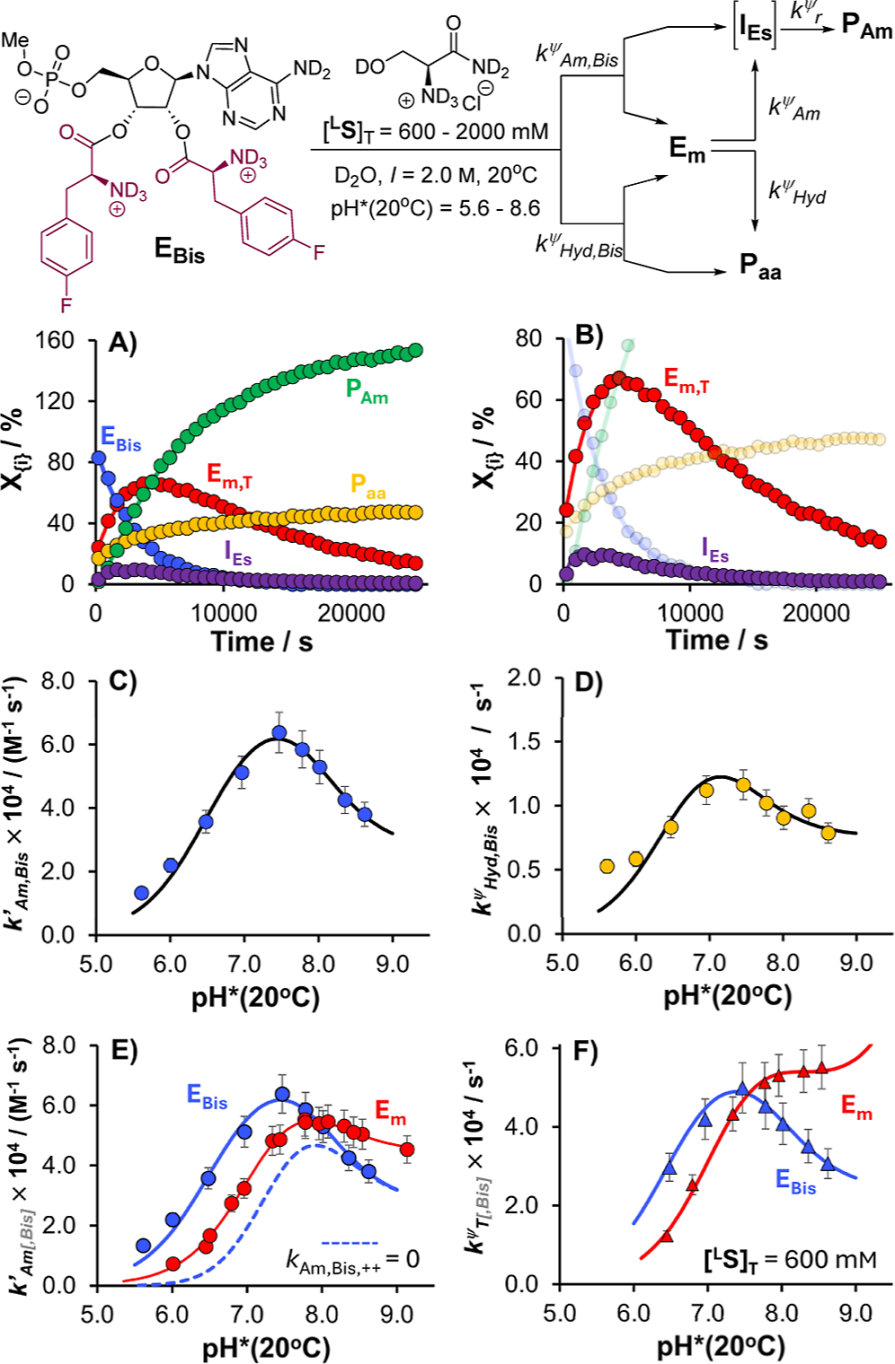
Example ^19^F{^1^H}_IG_ NMR monitoring data and kinetic analysis for the aminolysis, and concurrent hydrolysis, of **MepA-(****l****-PheF)**_**2**_ (**E**_**Bis**_) with l-serinamide in D_2_O (20 °C, pH* = 5.6–8.6, *I* = 2.0 M; [^**L**^**S**]_T_ = 600–2000 mM; [**E**_**Bis**_]_T,0_ ≈ 4 mM). (A) Temporal evolution profiles, with kinetic fits shown, for all key species under a low pH* regime (this example: pH* = 5.6, [^**L**^**S**]_T_ = 2000 mM). (B) Expanded view of (A), with a focus on the intermediate kinetics of **E**_**m,T**_ and **I**_**Es**_. (C) pH*–*k*′_Am,Bis_ profile with (unweighted) fit to experimental data (*k*′_Am,Bis_ = *k*_Am,Bis_^ψ^/[^**L**^**S**]_T_). (D) pH*–*k*_Hyd,Bis_^ψ^ profile with (unweighted) fit to experimental data. (E) Co-plot of the pH*–*k*′_Am_ (**E**_**m**_) and pH*–*k*′_Am,Bis_ (**E**_**Bis**_) profiles; dotted line reflects the pH*–*k*′_Am,Bis_ profile without any contribution from the aminolysis of the doubly protonated state, **E**_**Bis**_^**++**^ (i.e., *k*_Am,Bis_,_++_ = 0). (F) Co-plot of the pH*–*k*_T_^ψ^ (**E**_**m**_) and pH*–*k*_T,Bis_^ψ^ (**E**_**Bis**_) profiles ([^**L**^**S**]_T_ = 600 mM). Kinetic model for pH* > 6.5 = **E**_**Bis**_ → **E**_**m,T**_ + **P**_**Am**_ (*k*_Am,Bis_^ψ^), **E**_**Bis**_ → **E**_**m,T**_ + **P**_**aa**_ (*k*_Hyd,Bis_^ψ^), **E**_**m,T**_ → **P**_**Am**_ (*k*_Am_^ψ^), **E**_**m,T**_ → **P**_**aa**_ (*k*_Hyd_^ψ^). Kinetic model for pH* < 6.5: = **E**_**Bis**_ → **E**_**m,T**_ + **I**_**Es**_ (*k*_Am,Bis_^ψ^), **E**_**Bis**_ → **E**_**m,T**_ + **P**_**aa**_ (*k*_Hyd,Bis_^ψ^), **E**_**m,T**_ → **I**_**Es**_ (*k*_Am_^ψ^), **E**_**m,T**_ → **P**_**aa**_ (*k*_Hyd_^ψ^), **I**_**Es**_ → **P**_**Am**_ (*k*_r_^ψ^). All steps are (pseudo; ψ) first-order. Fitted parameters (*k*_Am,Bis,++_, *k*_Am,Bis,+_, *k*_Am,Bis,0_, *k*_OD^–^,Bis,++_, *k*_OD^–^,Bis,+_) and their standard errors in Table 4. p*K*_a_*(**E**_**Bis**_^**++**^) and p*K*_a_*(**E**_**Bis**_^**+**^) constrained during fitting; values taken from ^19^F{^1^H} NMR chemical shift analysis. See Supporting Information for details.

As anticipated, at sufficiently high pH* (>6.5), the reaction of **E**_**Bis**_ with l-serinamide in aqueous solution affords **E**_**m**_ as an intermediate—with the expected equilibrium ratio of 2′- and 3′-isomers at any given pH*—and **P**_**Am**_ and **P**_**aa**_ as terminal products. These species were found to evolve, as expected, according to a simple kinetic scheme in which **E**_**Bis**_ and **E**_**m**_ undergo irreversible, (pseudo)first-order transformations to either **P**_**Am**_ (**E**_**Bis**_: *k*_Am,Bis_^ψ^; **E**_**m**_: *k*_Am_^ψ^) or **P**_**aa**_ (**E**_**Bis**_: *k*_Hyd,Bis_^ψ^; **E**_**m**_: *k*_Hyd_^ψ^). Reactions monitored at pH* < 6.5 led, again as anticipated, to the minor accumulation of the intermediate species **I**_**Es**_ (Figure 5), thus requiring the use of a more complex analytical model for kinetic deconvolution (Figure 7). The dipeptide **(****l****-PheF)**_**2**_, which in principle could form from an intramolecular aminolysis within **E**_**Bis**_,^54^ was not detected under any conditions, in agreement with the findings of Hecht.^53^

**Figure 8:**
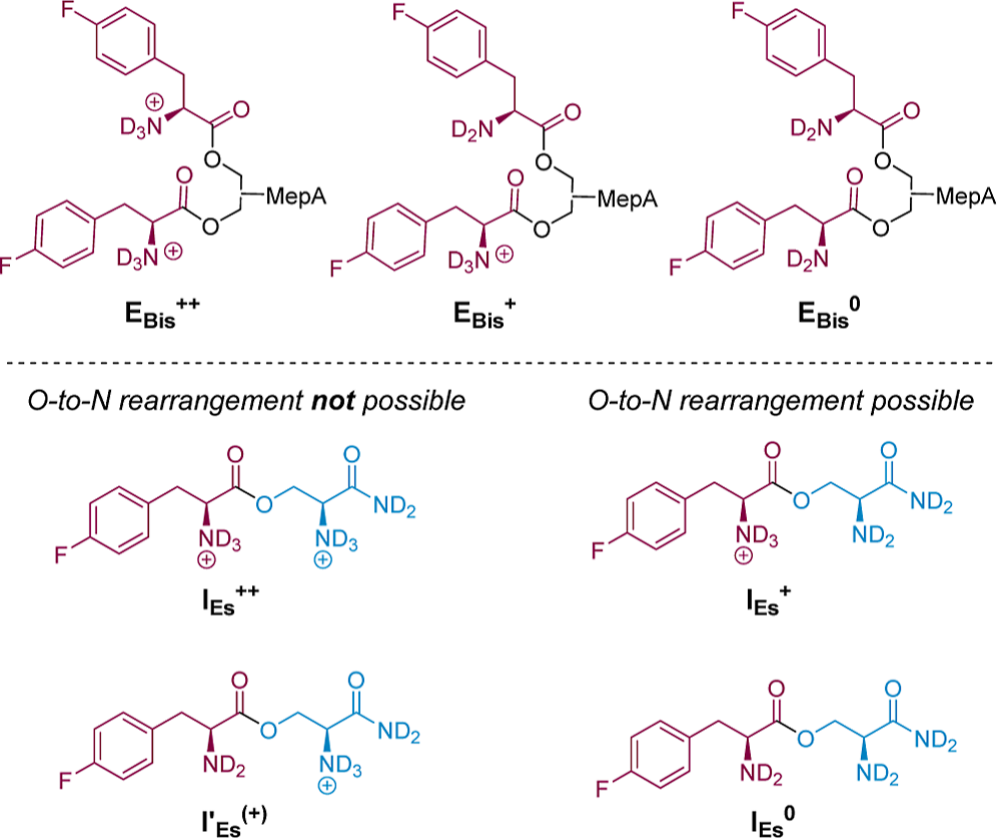
Structural depictions of **E**_**Bis**_ (simplified) and **I**_**Es**_ in their various charge states. Note that [**E**_**Bis**_^**+**^] = [**E**_**Bis,2′+**_^**+**^] + [**E**_**Bis,3′+**_^**+**^]. For simplicity, we assume that **I**_**Es**_^**+**^ and **I′**_**Es**_^**+**^ have identical microscopic acidities.

A satisfactory fit of the pH*–*k*′_Am,Bis_ profile was obtained by assuming that *k*′_Am,Bis_ comprises three terms, all featuring l-serinamide in its unionized state (^**L**^**S**^**0**^), including rates for the (formal) aminolysis of the bis-aminoacyl ester **E**_**Bis**_ in its (i) *N*,*N*′-bis-protonated state, **E**_**Bis**_^**++**^ (*k*_Am,Bis,++_), (ii) N-monoprotonated state, **E**_**Bis**_^**+**^ (*k*_Am,Bis,+_), and (iii) doubly free base state, **E**_**Bis**_^**0**^ (*k*_Am,Bis,0_).^55^ The pH*–*k*_Hyd,Bis_^ψ^ profile was deconvoluted in an analogous manner, by invoking separate terms for the saponification of **E**_**Bis**_^**++**^ (*k*_OD^–^,Bis,++_), **E**_**Bis**_^**+**^ (*k*_OD^–^,Bis,+_), and **E**_**Bis**_^**0**^ (*k*_OD^–^,Bis,0_).^56^ In each case, the intrinsic reactivities (second-order rate constants) were obtained by nonlinear fitting (unweighted) of the pH*–*k*′_Am,Bis_ and pH*–*k*_Hyd,Bis_^ψ^ profiles (see Supporting Information for full analysis).^56^ Given the more complex kinetic deconvolution (Figure 7, top) compared to reactions initiated with **E**_**m**_ (Figure 4), and given the more complex speciation of **E**_**Bis**_ (Figure 8), values for p*K*_a_*(**E**_**Bis**_^**++**^) and p*K*_a_*(**E**_**Bis**_^**+**^) were determined by ^19^F{^1^H} NMR chemical shift analysis (see Supporting Information) and constrained during fitting (Table 4).^57^ The fits to both profiles are generally very good, with the expected outlier at pH*(20 °C) = 5.6 likely the result of material partitioning of **I**_**Es**_ to **P**_**aa**_ (vide supra).^37^

**Table 4:** Kinetic and Acidity Parameters for the Aminolysis and Hydrolysis of **E**_**Bis**_ with l-Serinamide (20 °C, D_2_O, *I* = 2.0 M)^a^

species (X)	p*K*_a_*(X)	*k*_Am_/(M^–1^ s^–1^)	*k*_OD^–^_/(M^–1^ s^–1^)
**E**_**Bis**_^**++**^	6.43	0.011(1)^†^	1.0(1) × 10^4†^
**E**_**Bis**_^**+**^	7.47	2.9(2) × 10^–3^	8(2) × 10^2^
**E**_**Bis**_^**0**^		1.3(2) × 10^–4†^	n.d.
**E**_**m**_^**+**^	7.08(2)	5.8(2) × 10^–3^	5.7(2) × 10^3^
**E**_**m**_^**0**^		4.5(1) × 10^–4^	21(2)

a Second-order rate constants (*k*; M^–1^ s^–1^) obtained by nonlinear fitting (unweighted)^37b^ of experimental pH*–*k*′_Am,Bis_ and pH*–*k*_Hyd,Bis_^ψ^ profiles (D_2_O, *I* = 2.0 M) (see Supporting Information for equations); values for **E**_**m**_ taken directly from Table 2. Standard errors of second-order rate constants were determined with OriginPro 2024b unless otherwise stated and quoted in parentheses (e.g., 1.0(2) = 1.0 ± 0.2). p*K*_a_*(**E**_**Bis**_^**++**^) and p*K*_a_*(**E**_**Bis**_^**+**^) were constrained during fitting; values were taken from ^19^F{^1^H} NMR chemical shift analysis (see Supporting Information). ^†^Rate constants “statistically” corrected for two (nearly) degenerate sites. See Supporting Information for details.

On a broad level, our second-order rate constants appear to be consistent with the empirical observations of Hecht and co-workers,^23^ insomuch as the reactivity of the neutral bis*-*aminoacyl ester, **E**_**Bis**_^**0**^, is notably suppressed relative to the neutral mono-aminoacyl ester **E**_**m**_^**0**^. No saponification term for **E**_**Bis**_^**0**^ could be established with sufficient confidence in the pH* range of our experiments, and the term for transesterification is, after statistical correction, roughly a quarter of that for **E**_**m**_^**0**^. The hydrolytic stability of **E**_**m**_^**0**^ is particularly striking and the main factor behind the divergence of the pH*–*k*_T_^ψ^ and pH*–*k*_T,Bis_^ψ^ profiles (Figure 7F) at high pH*; though not measured directly,^58^ the similarity between **E**_**m**_^**0**^ and **E**_**m**_^**f**^ (Table 2) would suggest that comparable stability ought to characterize the N-formylated derivative **E**_**Bis**_^**f**^.

A more nuanced trend emerges for the monocationic state of the bis*-*aminoacyl ester, **E**_**Bis**_^**+**^: it is much less reactive than **E**_**m**_^**+**^ toward saponification but only modestly less susceptible to transesterification with l-serinamide. A reasonable interpretation of this disparity may be that neighboring group assistance from the vicinal 2′–OH group plays a more significant role in saponification^49^ than in transesterification;^7a^ given the insensitivity of classical transition state probes^49^ to such assistance, however, this conclusion must remain tentative. The statistically corrected reactivity of the doubly protonated bis-aminoacyl ester **E**_**Bis**_^**++**^, meanwhile, is enhanced relative to **E**_**m**_^**+**^, resulting in a crossover of the pH*–*k*′_Am_ and pH*–*k*′_Am,Bis_ profiles and in the total pseudo-first-order decays of **E**_**m**_ and **E**_**Bis**_, around pH* = 7.5 ((20 °C, D_2_O), *I* = 2.0 M). Thus, the bis-aminoacylated ester will, as previously observed, appear significantly more stable than the monoaminoacyl ester—but only at relatively high pH*.^59^ This property has been correlated to the use of such bis-aminoacyl esters as ribosomal substrates in extreme thermophiles,^60^ and in purely chemical terms, it would appear to recommend their use as aminoacyl acceptors (Figure 8).

## Evolutionary Implications and Conclusions

Ribosomal peptide synthesis in extant biology is remarkably efficient, and the underlying chemistry is shared by all known living systems. It is widely presumed to have underpinned the functioning of the Last Universal Common Ancestor (LUCA), but we know neither how this machinery emerged from chemistry nor what selective pressure drove its emergence. The chemistry at the heart of ribosomal peptide formation, direct ester aminolysis, works well in the protective, desolvated environment of the PTC of the ribosome, but in bulk aqueous solution, it is very inefficient. If, as imagined, ribosomal peptide synthesis is the consequence of the fortuitous emergence, and then extensive evolution, of some primordial transpeptidation ribozyme, the innate inefficiency of direct aminolysis in water would appear to pose a dichotomy for the origin of life.

Inspired by the previous observations of Orgel,^11^ and of others,^7,14,18^ we have therefore investigated an alternative, indirect manifold for preribosomal peptide formation that instead proceeds via the formation of a serinyl intermediate ester and then intramolecular aminolysis via O-to-N rearrangement. We have explored this manifold by way of studies on a chemical model system, in which various 4-fluorophenylalanyl esters of adenosine-5′-(*O*-methylphosphate) serve as aminoacyl donor (P-tRNA) analogues and hydroxyl-functionalized amino acid derivatives serve as corresponding aminoacyl acceptor (A-tRNA) analogues. By the application of structure–reactivity relationships, in situ reaction monitoring by ^19^F{^1^H}_IG_ NMR, pH–rate profile analysis, and various transition state probes, we show, inter alia, that (i) the competitive advantage provided by a hydroxymethyl/hydroxyethyl substituent in the aminoacyl acceptor is not only profound but also robust, both with respect to structural modulations of the aminoacyl acceptor itself (e.g., serine esterification and amidation) and of the donor (e.g., N-formylation, bis-aminoacylation, and 2′-deoxygenation); (ii) these substrates do, after all,^18,7a^ react to afford serinyl esters as critical intermediates, but relatively rapid O-to-N rearrangement will generally preclude the discernible accumulation of such esters at or above neutral pH; (iii) at the concentrations needed to afford significant peptide bond formation, hydroxyl-functionalized aminoacyl acceptor analogues undergo rate-determining transesterification on a bimolecular basis, in contrast to direct aminolysis with nonhydroxylated acceptors, whose rates are typically dominated by formally termolecular terms, arising from the requirement for a distinct general base;^7b^ (iv) the aminolysis of unionized, free aminoacyl esters appears to be kinetically similar and microscopically analogous to that of *N*-acyl-aminoacyl esters, allowing the use of free aminoacyl esters as P-tRNA analogues at high pH*; (v) the α-hydroxymethyl group of l-serine, or analogues thereof, is boosted by intramolecular general base catalysis from the vicinal amino group, but it still retains significant nucleophilicity—albeit for different reasons^14^—if this amino group is acylated; and (vi) bis-aminoacyl esters appear to be considerably more stable to the corresponding monoaminoacyl esters, with the exception of free bis-aminoacyl esters in their doubly protonated state.

Extant biology exploits various facets of simple chemistry to make ribosomal translation work—the initiation of translation with *N*-formyl methionine, for example, prevents the self-destruction of dipeptidyl-tRNAs by cyclization to form diketopiperazines—and it occurs to us that several of the mechanistic features of indirect peptidation could have been useful in the evolution of (pre)ribosomal peptide synthesis. At the broadest level, the ability of indirect peptidation to compete with hydrolysis at relatively modest concentrations of the aminoacyl acceptor (10–100 mM, rather than >1 M), without any catalysis, encapsulation, or templating, would clearly provide an “easier” starting point from which to discover a nascent transpeptidation ribozyme. Furthermore, the bimolecular kinetics of the indirect mechanism—apparently the result of intramolecular general base catalysis—also means that the uncatalyzed process will be less inhibited at higher dilution than direct aminolysis, typically dominated by termolecular kinetics.^7b^ That hydroxymethyl-bearing amino acids retain significant nucleophilicity when N-acylated^15^ is also suggestive to us, as it means that the incorporation of such amino acids into peptides or peptidyl-RNAs, in contrast to the alkyl amino acids (e.g., Gly, Ala, Leu, and Val), does not eliminate their essential character.

Given that substrate promiscuity is a hallmark of ribozymatic catalysis,^61^ it thus seems credible to envision a scenario in which a nascent transpeptidation ribozyme (protoribosome) would first emerge to catalyze indirect amidation with specific, α-hydroxymethyl-functionalized substrates, only to evolve, eventually, to accommodate—and encapsulate—otherwise analogous substrates devoid of these specific substituents. This evolutionary trajectory appeals to us as (i) it does not require evolution to supplant some completely different form of chemistry altogether in developing ribosomal translation; (ii) the initial, uncatalyzed chemistry relies on amino acids that emerge naturally from the cyanosulfidic reaction network of prebiotic chemistry;^17^ and (iii) the ribosome in extant biology remains perfectly capable of effecting transesterification^16^ as well as transpeptidation, consistent with the idea that evolution simply enabled the expansion of substrate scope.

It occurs to us that one might imagine two broadly distinguishable possibilities for the selective pressure that could have driven such an evolutionary trajectory for the ribosome. On the one hand, it may be imagined that the X-Ser peptides that ought to have initially predominated possessed some function that was directly selectable; in such a case, ribosomal evolution could have been driven by continuous selection for improvement of that function. It is not clear to us exactly what this function could have been, though an ability to furnish RNA, by complexation, with nucleophilic functionality might have been significant.^6,62^ Alternatively, it may have been that these X-Ser peptides had no function per se, in which case the selective pressure for ribosome evolution might have arisen from the process of their formation. In this respect, we are intrigued by the idea that (proto)ribosomal peptide synthesis evolved not to make peptides but to deblock aminoacyl RNA in preparation for replication/ligation, i.e., that the product of real evolutionary value in preribosomal peptide synthesis transpeptidation was the free oligoribonucleotide rather than the peptide. Again, the reactions of hydroxymethyl-bearing amino acids (e.g., serine) are particularly useful in this respect, as a significant degree of their nucleophilicity is preserved upon peptidation.^14^

## Supporting Information Available

Synthetic procedures, compound characterization data (NMR and ESI-MS), methods for kinetic studies, mathematical derivations of kinetic models, details of pH–rate profile deconvolution, raw kinetic data, raw pH–rate profile data, and summaries of all key rate coefficients (PDF)

## Abbreviations

P-tRNA: peptidyl transfer ribonucleic acid
A-tRNA: aminoacyl (acceptor) transfer ribonucleic acid
PTC: peptidyl transferase center
