# Supplementary material for: Indirect Formation of Peptide Bonds as a Prelude to Ribosomal Transpeptidation
Source: J Am Chem Soc. 2024 Dec 18;147(1):305–17. doi: 10.1021/jacs.4c10326 (PMC11726440; doi:10.1021/jacs.4c10326)
Supplement: Supplementary file 1 — ja4c10326_si_001.pdf [file ja4c10326_si_001.pdf]

Supplementary Information

Indirect Formation of Peptide Bonds as a Prelude to Ribosomal Transpeptidation

Harvey J. A. Dale* and John D. Sutherland*

Correspondence to: hdale@mrc-lmb.cam.ac.uk; johns@mrc-lmb.cam.ac.uk

Table of Contents

Experimental 6
Materials and Methods 6
Key Reagents 6
pH measurements 6
NMR spectroscopy 6
Chromatography 7
Kinetic studies 8
General 8
Typical reaction assembly 8
Longitudinal relaxation time constants 10
Kinetic analysis 13
Determination of pseudo first-order rate constants 13
Kinetic model 1: MepA-L-PheF (No Intermediate) 14
Kinetic model 2: MepA-L-PheF (With Intermediate) 15
Kinetic model 3: MepA-$(PheF)_2$ (No Intermediate) 16
Kinetic model 4: MepA-$(PheF)_2$ (With Intermediate) 18
pH-k^{Ψ} profile deconvolution 20
General considerations 20
k'_{Am} 21
k^{Ψ}_{Hyd} 24
$k'_{Am,Bis}$ 25
$k^{\Psi}_{Hyd,Bis}$ 27
pK_a* determination by NMR chemical shift analysis 29
$^{1}H/^{13}C\{^{1}H\}$ NMR titration: L-serinamide 29
pK_a computations for $^{L}S_{OH^+}$ 32
In situ pK_a determination by δ_F analysis 36
Kinetic data 40
Supplementary figures 40
Fitted parameters: MepA-L-PheF (E_m) + L-serinamide (^{L}S) 44
Unweighted fitting: data 44

Unweighted fitting: plots 46
Variance-weighted fitting: data 47
Variance-weighted fitting: plots 48
MepA-L-PheF (**E$_m$**) + L-serinamide (^{L}**S**); D_2O, 20 °C 49
Summary of pseudo first-order rate constants 49
Raw reaction profiles 50
MepA-L-PheF (**E$_m$**) + L-serinamide (^{L}**S**); H_2O, 20 °C 65
Summary of pseudo first-order rate constants 65
Raw reaction profiles 66
MepA-L-PheF (**E$_m$**) + L-serinamide (^{L}**S**); D_2O, 10 °C 75
Summary of pseudo first-order rate constants 75
Raw reaction profiles 76
MepA-L-PheF (**E$_m$**) + L-serinamide (^{L}**S**); D_2O, 30 °C 85
Summary of pseudo first-order rate constants 85
Raw reaction profiles 86
MepA-N-f-PheF (**E$_m^f$**) + L-serinamide (^{L}**S**); H_2O/D_2O, 20 °C 95
Summary of rate constants 95
Raw reaction profiles 96
MepA-(PheF)$_2$ (**E$_{Bis}$**) + L-serinamide (^{L}**S**); D_2O, 20 °C 100
Summary of pseudo first-order rate constants 100
Raw reaction profiles 101
Aminoacyl ester synthesis 110
Adenosine-5‘-O-methylphosphate (**MepA**) 110
2'(3')-O-(4-fluoro-L-phenylalanyl)-adenosine-5'-(O-methylphosphate) (**E$_m$**) 110
2',3'-bis-O-(4-fluoro-L-phenylalanyl)-adenosine-5'-(O-methylphosphate) (**E$_{Bis}$**) 112
2'(3')-O-(4-fluoro-D-phenylalanyl)-adenosine-5'-(O-methylphosphate) (**E$_m^D$**) 113
N-formyl-4-fluorophenylalanine (f-PheF-OH) 114
2'(3')-O-(N-formyl-4-fluoro-L/D-phenylalanyl)-adenosine-5'-(O-methylphosphate) (**E$_m^f$**) 114
Raw characterisation data: NMR 117
MepA 117
^{1}H (400 MHz, D_2O) 117
^{13}C (101 MHz, D_2O) 118

^{31}P (162 MHz, D_2O) 119
MepA-L-PheF (E_m) 120
^{1}H (400 MHz, D_2O) 120
^{13}C (101 MHz, D_2O) 121
^{31}P (162 MHz, D_2O) 122
^{19}F (377 MHz, D_2O) 123
MepA-(L-PheF)$_2$ (E_{bis}) 124
^{1}H (400 MHz, D_2O) 124
^{13}C (101 MHz, D_2O) 125
^{31}P (162 MHz, D_2O) 126
^{19}F (377 MHz, D_2O) 127
MepA-D-PheF (E_m^D) 128
^{1}H (400 MHz, D_2O) 128
^{13}C (101 MHz, D_2O) 129
^{31}P (162 MHz, D_2O) 130
^{19}F (377 MHz, D_2O) 131
MepA-N-f-L-PheF (E_m^f) 132
^{1}H (400 MHz, D_2O) 132
^{31}P (162 MHz, D_2O) 133
^{19}F (377 MHz, D_2O) 134
N-formyl-4-fluoro-L-phenylalanine (N-f-L-PheF-OH) 135
^{1}H (400 MHz, CD_3CN) 135
^{13}C (101 MHz, CD_3CN) 136
^{19}F (377 MHz, CD_3CN) 137
Raw characterisation data: ESI(+)-MS 138
MepA 138
MepA-L-PheF (E_m) 139
MepA-(L-PheF)$_2$ (E_{bis}) 140
MepA-D-PheF (E_m^D) 141
MepA-N-f-L-PheF (E_m^f) 142
N-formyl-4-fluoro-L-phenylalanine (N-f-L-PheF-OH) 143
References 144

Experimental

Materials and Methods

Key Reagents

Key reagents were obtained from *Thermo Scientific* (4-fluoro-L-phenylalanine, L-serinamide hydrochloride, N,N-dicyclohexylcarbodiimide), *Sigma Aldrich* (adenosine-5'-monophosphate monohydrate, L-serine, O-phospho-L-serine, L-serine tert-butyl ester hydrochloride, glycinamide hydrochloride, glycine, N,N-diisopropylethylamine, formic acid), *Santa Cruz Biotechnology* (L-threoninamide, boc-4-fluoro-L-phenylalanine, boc-4-fluoro-D-phenylalanine), *Honeywell* (trifluoroacetic acid), *Key Organics* (N-acetyl-L-serine), *Alfa Aesar* (L-alaninamide hydrochloride), *Acros Organics* (D-serine), or *Ambeed* (O-methyl-L-serine) and used without further purification. D_2O (Magnisolv, >99.9 %) was obtained from *Sigma Aldrich*, whilst ultrapure H_2O was obtained in-house from a *Millipore* Milli-Q Advantage A10 water purification system fitted with a Quantum TIX cartridge and Biopak polisher.

pH measurements

Measurements of pH were made using a *Mettler Toledo* SevenMulti pH Meter (MTC) combined with a *Thermo Fisher Scientific* Orion 8103BN Ross semi-micro pH electrode. The pH of all solutions was measured in Eppendorf tubes (1.5 mL), using an *Eppendorf* Thermomixer C fitted with a SmartBlock (1.5 mL) to maintain temperature control; for non-ambient temperatures, all solutions were equilibrated for five minutes prior to pH measurement. Each set of pH measurements was preceded by a manual calibration, at 20 °C, using phalate (pH(20°C) = 3.980; *Fisher Chemicals*), phosphate (pH(20°C) = 7.010; *Fisher Chemicals*), and borate (pH(20°C) = 9.190; Acros Organics) buffers.

NMR spectroscopy

All ^{1}H (400 MHz), $^{13}C\{^{1}H\}$ (128 MHz), $^{31}P/^{31}P\{^{1}H\}$ (162 MHz), $^{19}F/^{19}F\{^{1}H\}$ (376 MHz) and 2D (^{1}H-^{13}C HSQC, ^{1}H-^{13}C HMBC, ^{1}H-^{1}H COSY, ^{1}H-^{1}H NOESY, ^{1}H-^{1}H-ROESY) NMR spectra were acquired, without spinning, on a *Bruker* Ultrashield 400 Plus/Avance III spectrometer fitted with a room-temperature double resonance broadband probe (BBO 400S1 BBF-H-D with Z-Gradient; 5 mm) and a BCU05 cooling system. All samples were analysed in 5 mm borosilicate glass tubes (Norell Select Series; 400 MHz). The temperature of the probe was calibrated over the range 15 – 60 °C using 80% ethylene glycol in DMSO-d_6, and unless otherwise stated (e.g., for kinetics measurements), all NMR spectra were acquired at 300 K with a default VT gas (N_2) flow rate of 400 L hr^{-1}.

All spectra were processed using standard MestReNova software (Version 14.1.2), with phasing (zeroth- and first-order) optimised manually and baseline corrections (Whittaker Smoother

($^{19}F/^{19}F\{^1H\}$ or third-order Bernstein polynomial (^1H, ^{13}C, $^{31}P/^{31}P\{^1H\}$) applied automatically. All coupling constants, J, are quoted in Hz; coupling constants that did not match as a result of digitisation are reported as rounded averages. Multiplicities arising from coupling to spin ½ nuclei are indicated as s (singlet), brs (broad singlet), d (doublet, 1:1), brd (broad doublet, 1:1), t (triplet, 1:2:1), q (quartet, 1:3:3:1), quint. (quintet, 1:4:6:4:1), sext. (sextet, 1:5:10:10:5:1), sept. (septet, 1:6:15:20:15:6:1), m (multiplet), or combinations thereof.

Quantitative $^{19}F/^{19}F\{^1H\}$ NMR spectra (non-kinetics) for the determination of end-point selectivities were acquired using a hard π/6 excitation pulse (zg30/zgig30; p1 = 15 µs), an acquisition time of AQ = 1.5 s, a relaxation time of D1 = 10 s (recycle delay t_D = 11.5 s), an offset of -100.0 ppm and a spectral width of 100.0 ppm. Raw free induction decays (FIDs) were zero-filled to 512k points and subjected to exponential weighting (manually optimised, 0.3 – 0.6 Hz) prior to Fourier transform; transformed $^{19}F/^{19}F\{^1H\}$ spectra were manually phased (zeroth- and first-order) and their baselines corrected using the Whittaker smoother algorithm, with the filter and smooth factor optimised automatically.

Chromatography

Reverse-phase high-pressure liquid chromatography (RP-HPLC) at the analytical scale was typically used to assess the stability of stock solutions of **MepA-L-PheF**, or derivatives thereof, prior to their use in endpoint or kinetics experiments. Stock solutions for which >15% of the initial material had hydrolysed were not used. Such measurements were made using a *Thermo Scientific Dionex* Ultimate 3000 RS (U)HPLC system equipped with a variable wavelength UV-visible detector and a *Waters* Atlantis dC18 column (3 µm, 4.6 × 150 mm) (Eluent system: A = 20 mM TEAA in MQ-H_2O (pH 7.0), B = Acetonitrile (>99.9%, gradient grade); typical conditions = 1 mL min^{-1}, 25 °C (Column), 260 nm, 5-10-85% A; linear ramps: 1– 4 min (5 – 10% A), 4 – 11 min (10 – 85% A)). It was assumed that the molar extinction coefficient of **MepA** ε_{H2O}(260 nm) ≈ 1.5 × 10^4 M^{-1} cm^{-1} (~ pH 5), and that (mono- or bis-) aminoacylation has a negligible effect on this coefficient.

All preparative-scale RP-HPLC runs were conducted with an *Agilent* 1260 Infinity II LC equipped with a diode-array detector (wide-range) and either a *Waters* Atlantis T3 Prep OBD column (5 µm, 19 × 250 mm) or a *Phenomenex* Luna C18(2) semi-prep column (5 µm, 10 × 250 mm, 100 Å). (Eluent system: A = 20 mM formic acid in MQ-H_2O (pH 2.3), B = Acetonitrile (>99.9%, gradient grade). For each compound purified by preparative scale HPLC, exact gradients and conditions given in the full synthetic procedure.

Mass spectra were obtained using an *Agilent* 1260 Infinity LC equipped with an electrospray ionization (ESI) source, a 6130 single-quadrupole mass spectrometer and an *Agilent* InfinityLab Poroshell 120 EC-C18 column (2.7 µm, 3.0 × 50 mm). (Eluent system: A = 20 mM formic acid in MQ-H_2O; B = 20 mM formic acid in acetonitrile (>99.9%, LC-MS grade); typical conditions = 0.7 mL min^{-1}, 40 °C (Column), 260 nm, 5-10-85% A; linear ramps: 1– 4 min (5 – 10% A), 4 – 11 min (10 – 85% A)).

Kinetic studies

General

All kinetic data reported in the main text was obtained by *in situ* $^{19}F\{^{1}H\}$ NMR monitoring, with the sample remaining in the thermally-regulated probehead throughout the course of each reaction. All reactions were initiated by manual assembly, and the composition of the reaction mixture in each run was analysed periodically using the multi_zgvd program in TopSpin 3.2, with each time point characterized by a $^{19}F\{^{1}H\}$ spectrum acquired using inverse-gated ^{1}H-decoupling and: (i) between 32 and 256 transients (ns = 32 – 256); (ii) a hard π/6 excitation pulse (zgig30; p1 = 15 μs); (iii) a relaxation delay of t_{D1} = 2.0 s; (iv) an acquisition time of t_{AQ} = 1.50 s; (v) an inter-spectrum delay of at least t_{MD} > 5.0 s; (vi) a transmitter offset of -100 ppm; and (vii) a spectral width of 100.0 ppm. The total recycle time during the acquisition of each spectrum was thus t_D = 3.50 s. All spectra acquired in a kinetic run were processed identically as a stack in MestReNova software (Version 14.1.2), with FIDs zero-filled to 512k points and subjected to exponential weighting (0.5 Hz) prior to phasing (zeroth-order) and baseline corrections (Whittaker smoother, auto). Integrals were calculated according to the default sum algorithm.

Stock solutions of concentrated L-serinamide deuterochloride (2.5 – 3.0 M) were prepared by dissolving a known quantity of L-serinamide hydrochloride in D_2O, incubating at room temperature for 12 h, lyophilising this solution, and then re-dissolving the lyophilizate in D_2O to make up an identical total volume. Stock solutions of all other reagents (phosphonoacetic acid, potassium hydroxide) in D_2O were simply prepared by dissolution of the protiated material in D_2O. The concentrations of stock solutions of **MepA-L-PheF**, and derivatives thereof, were determined from their absorbance at 260 nm (A_{260}) as measured using a *NanoDrop*® ND-1000 spectrophotometer, assuming $\varepsilon_{260} \approx 1.5 \times 10^{4}$ M^{-1} cm^{-1}; these measurements were corroborated by quantitative $^{1}H/^{31}P$ NMR analysis at the end-point of each reaction, with phosphonoacetic acid (4 mM), present in all reactions, serving as an internal integration standard.

Typical reaction assembly

In a typical kinetics experiment, all mutually unreactive components – typically L-serinamide (**^{L}S**), KOD/KOH, KCl and phosphonoacetic acid, *vide supra* – were first assembled together in the 5mm NMR tube to afford a stable sample with a volume of > 600 uL (Solution 1); this sample was typically prepared < 4 h prior to reaction initiation. In preparation for monitoring, this pre-reactive sample was first loaded into the NMR probe and equilibrated at the appropriate temperature for 5 min, after which time the probe was automatically tuned to ^{1}H and matched, locked (^{2}H; D_2O), and subjected to gradient shimming; the ^{2}H lock level was then further optimised by refining the Z-X-Y-XZ-YZ-Z shims using the automated routine in Topshim. The receiver gain was optimised automatically, and a quantitative 1D ^{1}H spectrum acquired (zg30, t_{aq} = 4.0 s, t_{d1} = 10 s, ns = 4) to determine the quality of the shimming/lineshape, which was refined by further shimming if necessary. In preparation for $^{19}F\{^{1}H\}$ monitoring, the probe was subsequently re-

tuned and matched to $^{19}F/^{1}H$, the receiver gain set to its maximum value, and the appropriate acquisition parameters loaded. In all cases the pre-reactive sample was equilibrated at the appropriate temperature in for at least 10 min in the probehead prior to reaction initiation.

In short order, the pre-reactive sample was ejected from the spectrometer and moved to a proximate bench, whereupon it was uncapped and the reaction initiated by injection of an appropriate volume (typically 15 – 60 μL) of an aqueous stock solution of **MepA-L-PheF**, or derivative thereof (typically 40 – 160 mM), via a *Gilson* pipette. For reactions monitored at 10 °C, the stock solution of **MepA-L-PheF**, or derivative thereof, was added directly from a solution chilled on ice; for reactions monitored at > 20 °C, the stock solution was warmed to room temperature for 5 min prior to its addition to the NMR tube. The fully assembled reaction was rapidly mixed by inverting the capped sample (five times), and then swiftly transferred back to the spectrometer and loaded into the probe, after which monitoring was commenced immediately. Typically the sample was returned to the probehead within < 1 minute of its initial ejection. The total time elapsed between reaction initiation and acquisition of the first spectrum – the dead time, t_{Dead} – was recorded manually; the time point t_i corresponding to the ith spectrum was calculated according to: $t_i = t_{Dead} + (i-1).t_R$.

The concentrations reported in the schemes in the main manuscript pertain to the *final* concentrations of all species at t = 0 in the fully assembled reaction mixtures; pH*(T) values pertain to measurements taken at the *end-point* of the respective reactions. The final pH*(T) value was always within < 0.1 units of the pH*(T) of the corresponding *pre-reactive* solution (i.e., that prior to the addition of the aminoacyl ester).

Longitudinal relaxation time constants

To guide the optimisation of acquisition parameters for $^{19}F\{^1H\}$ NMR reaction monitoring (*vide supra*), longitudinal relaxation time constants (T1) were measured explicitly for ^{19}F nuclei in a range of key reaction components under conditions directly comparable to those used for the acquisition of kinetic data (D_2O, 20 °C, I = 2.0 M, KCl; **Table S1**). Systematic measurements of T1 constants across a (non-exhaustive) range of conditions were made for the ^{19}F nuclei in 4-fluorophenylalanine (L-PheF-OH; $\mathbf{P_{aa}}$) and N-(4-fluoro-L-phenylalanyl)-L-serinamide ($\mathbf{P_{Am}}$), and their N-formyl derivatives (pH*(20°C) = 5.6 – 9.2, $[^{L}\mathbf{S}]_T$ = 600 – 2000 mM); these measurements were made at the *end-points* of reactions between **MepA-(PheF)$_2$** and L-serinamide (**Scheme S1**). To suppress the rate of their decomposition by transesterification, approximate T1 constants for the ^{19}F nucleus in **MepA-L-PheF,** and the two ^{19}F nuclei in **MepA-(PheF)$_2$**, were only measured at neutral pH and using L-alaninamide (^{L}**A**), rather than L-serinamide (^{L}S), as a buffer (pH*(20°C) = 7.1, [^{L}**A**] = 600 Mm; **Figure S2**).

T1 constants were measured by conventional, non-selective $^{19}F\{^1H\}$ inversion-recovery experiments using the t1irig pulse sequence from Bruker. In each experiment, the recovery of longitudinal magnetisation was monitored by repeating the pulse sequence $\{t_{D1}$--π--τ--π/2--$t_{AQ}\}$ for a standardised list of 13 recovery delays (τ = 0.05 s, 0.1 s, 0.2 s, 0.5 s, 1.0 s, 1.5 s, 2.0 s, 2.5 s, 3.0 s, 5.0 s, 7.0 s, 10 s, 15 s), acquiring eight transients per spectrum (ns = 8) and imposing an extended relaxation delay of t_{D1} = 15 s between successive transients. Four dummy transients were used prior to the acquisition of each spectrum, and the standard π/2 pulse width (p1 = 15 μs) was used in all cases. The acquisition time (t_{aq} = 1.5 s), offset (-100.0 ppm) and spectral width (100.0 ppm) were the same as for all *in situ* monitoring experiments. The 13 $^{19}F\{^1H\}$ spectra obtained in each experiment were processed as a stack, with identical zero-filling (512k), exponential weighting (0.5 Hz), phasing (zeroth-order) and baseline corrections (Whittaker smoother) applied to all spectra.

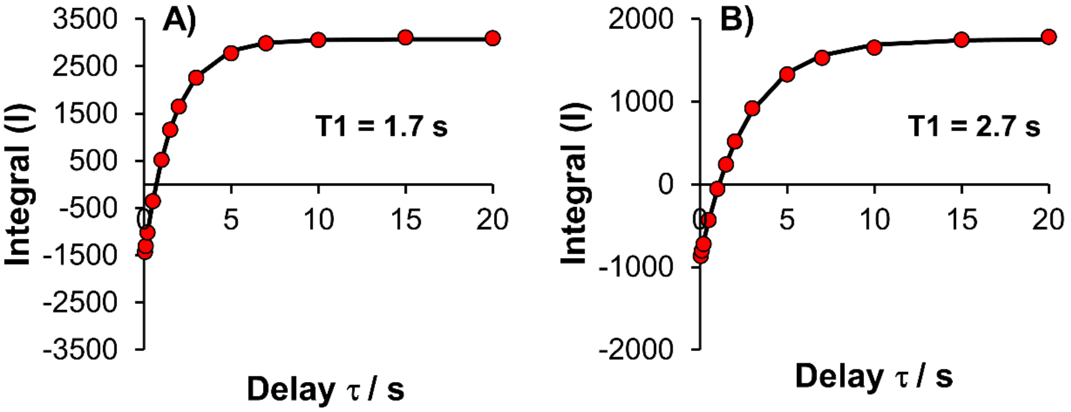

Figure S1: Typical $^{19}F\{^1H\}$ inversion recovery data from the measurement of T1 constants in this work (D_2O, pH*(20°C) = 7.8, *I* = 2.0 M, KCl). (A) Inversion recovery data for N-(4-fluoro-L-phenylalanyl)-L-serinamide ($\mathbf{P_{Am}}$) (T1 = 1.7 s, γ = 1.5). (B) Inversion recovery data for 4-fluorophenylalanine ($\mathbf{P_{aa}}$) (T1 = 2.7 s, γ = 1.5).

Relaxation time constants were extracted from plots of resonance intensities (I) vs τ by non-linear regression (**Figure S1**); to account for imperfect inversions from the initial π pulse, a three parameter model (I_∞, ζ, T1) was used in all cases (**Equation S1**).

$$I = I_\infty \left(1 - \zeta e^{-\frac{\tau}{T_1}}\right) \tag{S1}$$

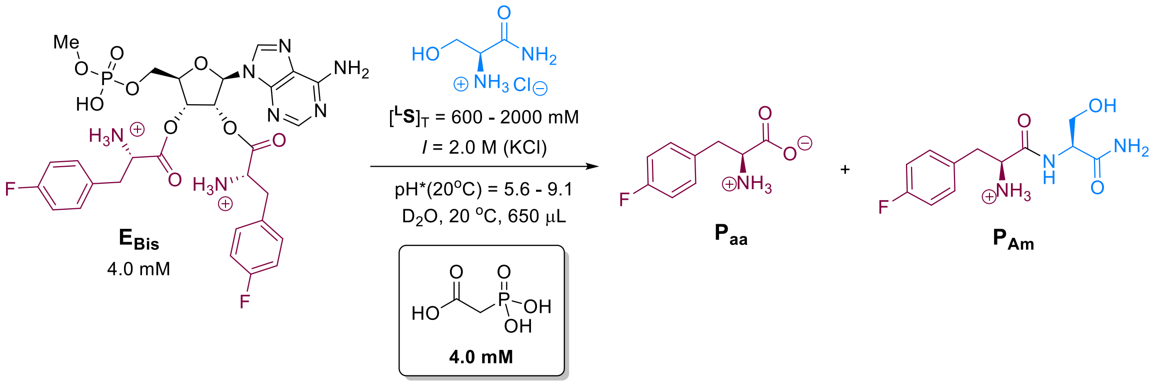

Scheme S1: Reaction conditions used to form $\mathbf{P_{aa}}$ and $\mathbf{P_{Am}}$ *in situ* and then to determine T1 constants for the ^{19}F nuclei thereof. $\mathbf{E_{Bis}}$ and $\mathbf{E_m}$, formed as an intermediate, were too short-lived under these conditions to measure the T1 constants of their ^{19}F nuclei directly.

	pH*(20°C)				
	**9.1**	**8.6**	**7.8**	**6.5**	**5.6**
[^{L}**S**]$_T$ / mM	600	600	600	600	2000
δ_F($\mathbf{P_{Am}}$) / ppm	-116.47	-116.41	-115.87	-115.19	-115.04
T_1 ($\mathbf{P_{Am}}$) / s	1.6	1.8	1.7	1.6	1.3
δ_F($\mathbf{P_{aa}}$) / ppm	-115.94	-115.82	-115.74	-115.73	-115.63
T_1 ($\mathbf{P_{aa}}$) / s	2.4	2.7	2.7	2.7	2.1
γ	1.5	1.5	1.5	1.5	1.5

Table S1: Longitudinal relaxation time constants (T1) for N-(4-fluoro-L-phenylalanyl)-L-serinamide ($\mathbf{P_{Am}}$) and 4-fluorophenylalanine ($\mathbf{P_{aa}}$) as a function of pH* (D_2O, 20 °C, *I* = 2.0 M, KCl) obtained via conventional inversion recovery experiments. Chemical shifts δ_F shifts referenced to trifluoroacetate (-75.15 ppm). T1 constants measured at the end-point of the reaction given in the scheme between **MepA-(PheF)$_2$** and L-serinamide (**Scheme S1**).

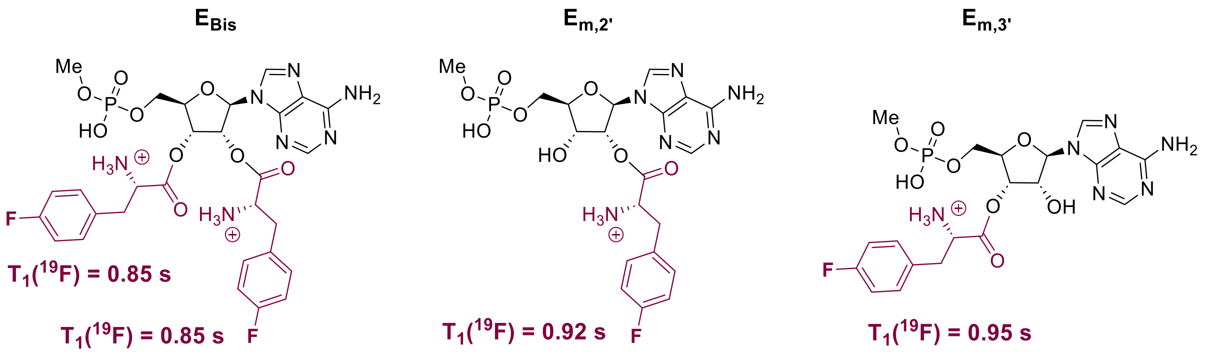

Figure S2: Longitudinal relaxation time constants (T1) for $\mathbf{E_{Bis}}$, $\mathbf{E_{m,3'}}$ and $\mathbf{E_{m,2'}}$ in near-neutral aqueous solution (pH* = 7.1, D_2O, 20 °C, *I* = 2.0 M, KCl; [$^{\mathbf{L}}\mathbf{A}$] = 600 mM) obtained via conventional inversion recovery experiments. T1 constants are approximate values, obtained by non-linear regression to **equation S1** without any correction to account for hydrolytic decay. To minimise hydrolysis, L-alaninamide, $^{\mathbf{L}}\mathbf{A}$, was used as a buffer rather than L-serinamide. Chemical shifts δ_F shifts referenced to trifluoroacetate (-75.15 ppm).

For all reaction courses monitored by $^{19}F\{^1H\}$ NMR in this work, each timepoint was characterised by a $^{19}F\{^1H\}$ spectrum acquired using a standard pulse-acquire sequence with multiple transients (ns > 32). The integral of a given resonance in each such spectrum acquired in this way will depend on the concentration of the corresponding species *i*, the longitudinal relaxation constant of the detected nucleus in that species ($T_{1,i}$), and the overall recycle delay (t_R) in the pulse sequence. If a hard excitation pulse of angle θ is used, the measured (steady-state) integral $I_{ss,i}$ – reflecting the incomplete recovery of longitudinal magnetisation during the recycle delay – will depend on θ, t_R and $T_{1,i}$ in accordance with:

$$I_{SS,i} = I_{0,i} \sin\theta \left(\frac{1 - e^{\frac{t_R}{T_{1,i}}}}{\cos\theta - e^{\frac{t_R}{T_{1,i}}}} \right) \tag{S2}$$

where $I_{0,i}$ is the integral one would observe for species *i* if the detected nucleus was allowed to reach its equilibrium magnetisation between successive hard π/2 pulses. The inherent accuracy of the underlying quantitation, χ, for *any* pulse angle θ is then:

$$\chi = \frac{\left(\frac{I_{SS}}{I_0}\right)}{\lim_{t_R \to \infty} \left(\frac{I_{SS}}{I_0}\right)} = \left(\frac{1 - e^{\frac{t_R}{T_1}}}{\cos\theta - e^{\frac{t_R}{T_1}}} \right) \tag{S3}$$

All $^{19}F\{^1H\}$ spectra acquired during *in situ* monitoring experiments in this work were obtained using a recycle delay of t_R = 3.5 s and hard π/6 excitation pulses, and the longest T1 constant measured for any species under ambient conditions (20°C) was T1 = 2.7 s (4-fluorophenylalanine; see **Table S1**). Accordingly, the *maximum* quantitation error in this work ought to be < 5% (i.e., χ > 95 %).

Kinetic analysis

Determination of pseudo first-order rate constants

Pseudo first-order rate constants (generally k^{ψ}) were determined by fitting experimental mole fraction data to equations derived analytically from one of a number of kinetic models (vide infra). In all cases, experimental data, obtained by *in situ* $^{19}F\{^{1}H\}$ NMR monitoring, was fitted by standard non-linear least-squares regression without any weighting. Standard errors in the pseudo first-order rate constants obtained by non-linear fitting were generally < 5%; on this basis, and on the basis that uncertainties in the $^{19}F\{^{1}H\}$ NMR integrals themselves are also subject to error of < 5% (vide supra), uncertainties in k^{ψ} were approximated conservatively to be $\pm$ 10%.

Experimental mole fractions at each time point during a reaction course were calculated by dividing the integral of the corresponding ^{19}F resonance for each species by the sum of the integrals of *all* resonances (including, when present, *both* ^{19}F resonances for the bis-aminoacyl ester, $\mathbf{E_{Bis}}$). The sum of integrals calculated in this manner was found to be constant in all kinetic runs, i.e. it did not show any systematic drift during any reaction course, indicating no lost mass balance. For reactions involving the bis-aminoacyl ester, $\mathbf{E_{Bis}}$, the mole fractions were arbitrarily normalized to 100% at t = 0.

It should be noted that the kinetic models below assume, either implicitly or explicitly, that the intermediate species $\boldsymbol{I_{Es}}$ *undergoes quantitative conversion to* $\boldsymbol{P_{Am}}$ *across all pH*, and that its hydrolysis to* $\boldsymbol{P_{aa}}$ *is negligible. We judge this to be a very reasonable assumption at pH*(20°C) > 6.5, as well as a practically necessary one in the context of kinetic deconvolution with minor/trace intermediates, but note that* k^{ψ} *values at lower pH* (i.e., pH*(20°C) < 6), where hydrolysis will better compete with intramolecular O-to-N rearrangement, are likely to be subject to greater error than those at higher pH*. We did not seek to measure the partition of* $\boldsymbol{I_{Es}}$ *directly on account of experimental limitations, but note that such measurements would be of great value.*

Kinetic model 1: MepA-L-PheF (No Intermediate)

For the reaction of **MepA-L-PheF** ($\mathbf{E_m}$) with L-serinamide ($^{\mathbf{L}}\mathbf{S}$) in aqueous solution, the rate of decay of the total concentration $[\mathbf{E_m}]_T$ may be expressed as:

$$\frac{d[\mathbf{E_m}]_T}{dt} = -(v_{Am} + v_{Hyd}) = -(k^{\psi}_{Am} + k^{\psi}_{Hyd})[\mathbf{E_m}]_T = -k^{\psi}_T\,[\mathbf{E_m}]_T \quad \text{(S4)}$$

where k^{ψ}_{Am} is the pseudo first-order rate constant for (formal) aminolysis and k^{ψ}_{Hyd} is the pseudo first-order rate constant for hydrolysis. The temporal evolution of $[\mathbf{E_m}]_T$ is a simple monoexponential decay, and the mole fraction of total monoester, $\mathbf{X}_{\{Em,T\}}$, may be expressed as:

$$\boldsymbol{X}_{\{\boldsymbol{E_m},\boldsymbol{T}\}} = \frac{[\mathbf{E_m}]_T}{[\mathbf{E_m}]_{T,0}} = e^{-k^{\psi}_T t} \quad \text{(S5)}$$

where $[\mathbf{E_m}]_{T,0}$ is the *initial* concentration of $\mathbf{E_{m,T}}$ and k^{ψ}_T is the total pseudo first-order rate constant for the decomposition of $\mathbf{E_{m,T}}$.

In the limit of negligible accumulation of the ester intermediate, the rate of total amide, $\mathbf{P_{Am,T}}$, formation will be:

$$\frac{d[\mathbf{P_{Am}}]_T}{dt} = v_{Am} = k^{\psi}_{Am}[\mathbf{E_m}]_T = k^{\psi}_{Am}[\mathbf{E_m}]_{T,0}e^{-k^{\psi}_T \cdot t} \quad \text{(S6)}$$

$$k^{\psi}_{Am} = k'_{Am}[^{\mathbf{L}}\mathbf{S}]_{\mathbf{T}} \quad \text{(S7)}$$

where k'_{Am}, distinct from the pseudo first-order constant k^{ψ}_{Am}, is the observed/empirical *second-order* rate constant for (formal) aminolysis by L-serinamide. In general, this is not a microscopic second-order rate constant, but a weighted sum of microscopic rate constants reflecting the aminolysis of the various ionisation states/regioisomers of $\mathbf{E_m}$ at a given pH(*) and temperature.

The temporal evolution of the total amide mole fraction, $\mathbf{X}_{\{PAm,T\}}$, under this regime is:

$$\boldsymbol{X}_{\{\boldsymbol{P_{Am}},\boldsymbol{T}\}} = \frac{[\mathbf{P_{Am}}]_{\mathbf{T}}}{[\mathbf{E_m}]_{\mathbf{T,0}}} = \left(\frac{k^{\psi}_{Am}}{k^{\psi}_T}\right)\left(1 - e^{-k^{\psi}_T t}\right) = f_{Am}\left(1 - e^{-k^{\psi}_T t}\right) \quad \text{(S8)}$$

Similarly, the rate of hydrolysis, and thereby amino acid formation, will be:

$$\frac{d[\mathbf{P_{aa}}]_{\mathbf{T}}}{dt} = v_{Hyd} = k^{\psi}_{Hyd}[\mathbf{E_m}]_T = k^{\psi}_{Hyd}[\mathbf{E_m}]_{T,0}e^{-k^{\psi}_T \cdot t} \quad \text{(S9)}$$

and the mole fraction of the total amino acid – the product of hydrolysis – $\mathbf{X}_{\{\mathbf{Paa,T}\}}$ will vary with time according to:

$$X_{\{P_{aa},T\}} = \frac{[\mathbf{P_{aa}}]_{\mathbf{T}}}{[\mathbf{E_m}]_{\mathbf{T,0}}} = \left(\frac{k^{\psi}_{Hyd}}{k^{\psi}_T}\right)\left(1 - e^{-k^{\psi}_T t}\right) = f_{Hyd}\left(1 - e^{-k^{\psi}_T t}\right) \quad \text{(S10)}$$

Kinetic model 2: MepA-L-PheF (With Intermediate)

In the case of appreciable accumulation of ester intermediate $\mathbf{I_{Es}}$ in the reaction of $\mathbf{E_m}$ with L-serinamide, the equations for $\mathbf{X_{\{Em,T\}}}$ and $\mathbf{X_{\{Paa,T\}}}$ remain unaffected relative to kinetic model 1; however, the equations describing the total mole fractions of the intermediate, $\mathbf{X_{\{IEs,T\}}}$, and the amide, $\mathbf{X_{\{PAm,T\}}}$, are more complex. The rate of change of $[\mathbf{I_{Es}}]_T$ is given by:

$$\frac{d[\mathbf{I_{Es}}]_\mathrm{T}}{dt} = k_{Am}^{\psi}[\mathbf{E_m}]_T - k_r^{\psi}[\mathbf{I_{Es}}]_T = v_{Am} - v_r \tag{S11}$$

where $k^{\Psi}{}_{r}$ is the (pseudo) first-order rate constant for the *O*-to-*N* rearrangement of $\mathbf{I_{Es}}$. Using integrating factors, this first-order differential equation can be solved analytically to afford the solution for $\mathbf{X_{\{IEs,T\}}}$ below:

$$\frac{d[\mathbf{I_{Es}}]_\mathrm{T}}{dt} + k_r^{\psi}[\mathbf{I_{Es}}]_\mathrm{T} = k_{Am}^{\psi}[\mathbf{E_m}]_{\mathrm{T},0}\, e^{-k_T^{\psi}t} \tag{S12}$$

$$\frac{d}{dt}\left([\mathbf{I_{Es}}]_\mathrm{T} e^{k_r^{\psi}t}\right) = k_{Am}^{\psi}[\mathbf{E_m}]_{\mathrm{T},0}\, e^{(k_r^{\psi}-k_T^{\psi})t} \tag{S13}$$

$$\mathbf{X}_{\{\mathbf{I_{Es}},\mathrm{T}\}} = \frac{[\mathbf{I_{Es}}]_\mathrm{T}}{[\mathbf{E_m}]_{\mathrm{T},0}} = \left(\frac{k_{Am}^{\psi}}{k_r^{\psi}-k_T^{\psi}}\right)\left(e^{-k_T^{\psi}t} - e^{-k_r^{\psi}.t}\right) = f_{Am}\left(\frac{k_T^{\psi}}{k_r^{\psi}-k_T^{\psi}}\right)\left(e^{-k_T^{\psi}t} - e^{-k_r^{\psi}t}\right) \tag{S14}$$

The mole fraction of the amide product, $\mathbf{X_{\{PAm,T\}}}$, can be derived from this in a standard manner from, i.e.:

$$\frac{d\mathbf{X}_{\{\mathrm{P_{Am,T}}\}}}{dt} = k_r^{\psi}\mathbf{X}_{\{\mathrm{I_{Es,T}}\}} = k_r^{\psi} f_{Am}\left(\frac{k_T^{\psi}}{k_r^{\psi}-k_T^{\psi}}\right)\left(e^{-k_T^{\psi}t} - e^{-k_r^{\psi}t}\right) \tag{S15}$$

$$\boldsymbol{X}_{\{P_{Am,T}\}} = k_r^{\psi} f_{Am}\left(\frac{k_T^{\psi}}{k_r^{\psi}-k_T^{\psi}}\right)\left\{\left(\frac{1}{k_r^{\psi}}e^{-k_r^{\psi}t} - \frac{1}{k_T^{\psi}}e^{-k_T^{\psi}t}\right) - \left(\frac{1}{k_r^{\psi}} - \frac{1}{k_T^{\psi}}\right)\right\} \tag{S16}$$

$$\boldsymbol{X}_{\{P_{Am,T}\}} = f_{Am}\left\{1 + \left(\frac{1}{k_r^{\psi}-k_T^{\psi}}\right)\left(k_T^{\psi}e^{-k_r^{\psi}t} - k_r^{\psi}e^{-k_T^{\psi}t}\right)\right\} \tag{S17}$$

Kinetic model 3: MepA-(PheF)$_2$ (No Intermediate)

For the reaction of **MepA-(PheF)$_2$** (**E_{Bis}**) with L-serinamide (^{L}**S**) in aqueous solution, the rate of decay of the total concentration [**E_{Bis}**]$_T$ may be expressed as:

$$\frac{d[\mathbf{E_{Bis}}]_\mathrm{T}}{dt} = -\left(v_{Am,Bis} + v_{Hyd,Bis}\right) = -\left(k^{\psi}_{Am,Bis} + k^{\psi}_{Hyd,Bis}\right)[\mathbf{E_{Bis}}]_\mathrm{T} = -k^{\psi}_{T,Bis}[\mathbf{E_{Bis}}]_\mathrm{T} \qquad \text{(S18)}$$

where $k^{\psi}_{\mathrm{Am,Bis}}$ is the pseudo first-order rate constant for (formal) aminolysis and $k^{\psi}_{\mathrm{Hyd,Bis}}$ is the pseudo first-order rate constant for hydrolysis of **E_{Bis}**. These constants are distinct from k^{ψ}_{Am} and k^{ψ}_{Hyd}, which pertain to the (formal) aminolysis and hydrolysis of **E_m**, the mono-aminoacyl ester, respectively. The temporal evolution of [**E_{Bis}**]$_T$ is a simple monoexponential decay, i.e.

$$[\mathbf{E_{Bis}}]_\mathrm{T} = [\mathbf{E_{Bis}}]_\mathrm{T,0}\; e^{-k^{\psi}_{T,Bis} t} \qquad \text{(S19)}$$

where [**E_{Bis}**]$_{T,0}$ is the *initial* concentration of **$E_{Bis,T}$** and $k^{\psi}_{\mathrm{T,Bis}}$ is the total pseudo first-order rate constant for the decomposition of **$E_{Bis,T}$**. On account of the fact that stock solutions of **E_{Bis}** invariably exhibited some background hydrolysis (5 – 15 %), in practice **$X^*_{\{EBis,T\}}$** was calculated from the modified equation below, where $c_{\mathrm{Bis,0}} < 1$ was treated as a variable parameter during non-linear fitting.

$$\mathbf{X}^*_{\{\mathbf{E_{Bis,T}}\}} = c_{Bis,0}\; e^{-k^{\psi}_{T,Bis} t} \qquad \text{(S20)}$$

In the limit of negligible accumulation of the ester intermediate, the rate of change of [**E_m**]$_T$ – now also an intermediate itself – is given by:

$$\frac{d[\mathbf{E_m}]_\mathbf{T}}{dt} = k^{\psi}_{T,Bis}[\mathbf{E_{Bis}}]_\mathbf{T} - k^{\psi}_{T}[\mathbf{E_m}]_\mathbf{T} = \left(v_{Am,Bis} + v_{Hyd,Bis}\right) - \left(v_{Am} + v_{Hyd}\right) \qquad \text{(S21)}$$

which can in turn be solved analytically, by the method of integrating factors, to give the equation below. Note that a distinct term is retained for [**E_m**]$_{T,0}$, vide supra.

$$[\mathbf{E_m}]_\mathbf{T} = [\mathbf{E_{Bis}}]_\mathbf{T,0}\left(\frac{k^{\psi}_{T,Bis}}{k^{\psi}_{T} - k^{\psi}_{T,Bis}}\right)\left(e^{-k^{\psi}_{T,Bis} t} - e^{-k^{\psi}_{T} t}\right) + [\mathbf{E_m}]_\mathbf{T,0}\; e^{-k^{\psi}_{T} t} \qquad \text{(S22)}$$

In practice, the calculated mole fraction of **$E_{m,T}$** was determined using **$X^*_{\{Em,T\}}$**, in which $c_{\mathrm{m,0}}$ was treated as a variable parameter during non-linear fitting.

$$\mathbf{X}^*_{\{\mathbf{E_{m,T}}\}} = c_{Bis,0}\left(\frac{k^{\psi}_{T,Bis}}{k^{\psi}_{T} - k^{\psi}_{T,Bis}}\right)\left(e^{-k^{\psi}_{T,Bis} t} - e^{-k^{\psi}_{T} t}\right) + c_{m,0}\; e^{-k^{\psi}_{T} t} \qquad \text{(S23)}$$

When both $\mathbf{E_{Bis}}$ and $\mathbf{E_m}$ are present in L-serinamide buffer, both can undergo aminolysis and hydrolysis, affording $\mathbf{P_{Am}}$ and $\mathbf{P_{aa}}$, respectively. The rate of formation in the case of, e.g., $\mathbf{P_{Am}}$, is thus:

$$\frac{d[\mathbf{P_{Am}}]_\mathrm{T}}{dt} = k^{\psi}_{Am,Bis}[\mathbf{E_{Bis}}]_\mathrm{T} + k^{\psi}_{Am}[\mathbf{E_m}]_\mathbf{T} = v_{Am,Bis} + v_{Am} \quad \text{(S24)}$$

$$\frac{d[\mathbf{P_{Am}}]_T}{dt} = k^{\psi}_{Am,Bis}[\mathbf{E_{Bis}}]_{\mathrm{T},0}\, e^{-k^{\psi}_{T,Bis}t} + [\mathbf{E_{Bis}}]_{\mathbf{T,0}} \left(\frac{k^{\psi}_{Am}\, k^{\psi}_{T,Bis}}{k^{\psi}_{T} - k^{\psi}_{T,Bis}}\right)\left(e^{-k^{\psi}_{T,Bis}\,t} - e^{-k^{\psi}_{T}\,t}\right) + k^{\psi}_{Am}[\mathbf{E_m}]_{\mathbf{T,0}}\, e^{-k^{\psi}_{T}\,t} \quad \text{(S25)}$$

This differential equation can be solved analytically, assuming $[\mathbf{P_{Am}}]_{\mathrm{T},0} = 0$, giving

$$[\mathbf{P_{Am}}]_\mathrm{T} = f_{Am,Bis}[\mathbf{E_{Bis}}]_{\mathrm{T},0}\left(1 - e^{-k^{\psi}_{T,Bis}t}\right) + f_{Am}[\mathbf{E_{Bis}}]_{\mathbf{T,0}}\left\{1 + \left(\frac{1}{k^{\psi}_{T} - k^{\psi}_{T,Bis}}\right)\left(k^{\psi}_{T,Bis}e^{-k^{\psi}_{T}t} - k^{\psi}_{T}e^{-k^{\psi}_{T,Bis}t}\right)\right\} + f_{Am}[\mathbf{E_m}]_{\mathbf{T,0}}\left(1 - e^{-k^{\psi}_{T}t}\right) \quad \text{(S26)}$$

where the constants $f_{Am,Bis}$ and f_{Am} reflect the empirical selectivity for aminolysis of $\mathbf{E_{Bis,T}}$ and $\mathbf{E_{m,T}}$, respectively:

$$f_{Am,Bis} = \frac{k^{\psi}_{Am,Bis}}{k^{\psi}_{T,Bis}} \qquad f_{Am} = \frac{k^{\psi}_{Am}}{k^{\psi}_{T}} \quad \text{(S27)}$$

In practice, the mole fraction of $\mathbf{P_{Am,T}}$, $\mathbf{X_{\{PAm,T\}}}$, was calculated according to:

$$\mathbf{X}^{*}_{\{\mathbf{P_{Am}},\mathbf{T}\}} = f_{Am,Bis}\cdot c_{Bis,0}\left(1 - e^{-k^{\psi}_{T,Bis}t}\right) + f_{Am}\cdot c_{Bis,0}\left\{1 + \left(\frac{1}{k^{\psi}_{T} - k^{\psi}_{T,Bis}}\right)\left(k^{\psi}_{T,Bis}e^{-k^{\psi}_{T}t} - k^{\psi}_{T}e^{-k^{\psi}_{T,Bis}t}\right)\right\} + f_{Am}\cdot c_{m,0}\left(1 - e^{-k^{\psi}_{T}t}\right) \quad \text{(S28)}$$

The total rate of formation of $\mathbf{P_{aa}}$, the hydrolysis product, is similarly:

$$\frac{d[\mathbf{P_{aa}}]_\mathrm{T}}{dt} = k^{\psi}_{Hyd,Bis}[\mathbf{E_{Bis}}]_\mathrm{T} + k^{\psi}_{Hyd}[\mathbf{E_m}]_\mathbf{T} = v_{Hyd,Bis} + v_{Hyd} \quad \text{(S29)}$$

The mole fraction of the amino acid, $\mathbf{X_{\{Paa,T\}}}$, was calculated analogously to $\mathbf{X_{\{PAm,T\}}}$, with an additional term, $c_{aa,0}$, reflecting the fractional hydrolysis of the stock solution of $\mathbf{E_{Bis}}$. The constants $c_{aa,0}$ and $c_{m,0}$ were both floated independently during non-linear fitting.

$$\mathbf{X}^{*}_{\{\mathbf{P_{aa,T}}\}} = f_{Hyd,Bis}\cdot c_{Bis,0}\left(1 - e^{-k^{\psi}_{T,Bis}\cdot t}\right) + f_{Hyd}\cdot c_{Bis,0}\left\{1 + \left(\frac{1}{k^{\psi}_{T} - k^{\psi}_{T,Bis}}\right)\left(k^{\psi}_{T,Bis}e^{-k^{\psi}_{T}t} - k^{\psi}_{T}e^{-k^{\psi}_{T,Bis}t}\right)\right\} + f_{Hyd}\cdot c_{m,0}\left(1 - e^{-k^{\psi}_{T}\cdot t}\right) + c_{aa,0} \quad \text{(S30)}$$

Kinetic model 4: MepA-(PheF)$_2$ (With Intermediate)

In the case of appreciable accumulation of ester intermediate $\mathbf{I_{Es}}$ in the reaction of $\mathbf{E_{Bis}}$ with L-serinamide, the equations for $\mathbf{X_{\{EBis,T\}}}$, $\mathbf{X_{\{Em,T\}}}$ and $\mathbf{X_{\{Paa,T\}}}$ remain unaffected relative to kinetic model 3; however, the equations describing the mole fractions of the intermediate, $\mathbf{X_{\{IEs,T}}$, and the amide, $\mathbf{X_{\{PAm,T\}}}$, are more complex. The rate of change of $[\mathbf{I_{Es}}]_\mathrm{T}$ is given by:

$$\frac{d[\mathbf{I_{Es}}]_\mathrm{T}}{dt} = k_{Am,Bis}^{\psi}[\mathbf{E_{Bis}}]_T + k_{Am}^{\psi}[\mathbf{E_m}]_T - k_r^{\psi}[\mathbf{I_{Es}}]_T = v_{Am,Bis} + v_{Am} - v_r \qquad \text{(S31)}$$

$$\frac{d[\mathbf{I_{Es}}]_\mathrm{T}}{dt} + k_r^{\psi}[\mathbf{I_{Es}}]_T = f_{Am,Bis}k_{T,Bis}^{\psi}[\mathbf{E_{Bis}}]_{\mathrm{T,0}}\, e^{-k_{T,Bis}^{\psi}t} + f_{Am}[\mathbf{E_{Bis}}]_{\mathbf{T,0}}\left(\frac{k_T^{\psi}k_{T,Bis}^{\psi}}{k_T^{\psi}-k_{T,Bis}^{\psi}}\right)\left(e^{-k_{T,Bis}^{\psi}t} - e^{-k_T^{\psi}t}\right)$$
$$+ f_{Am}k_T^{\psi}[\mathbf{E_m}]_{\mathbf{T,0}}\, e^{-k_T^{\psi}t} \qquad (S32)$$

This can be solved analytically using the method of integrating factors, via:

$$\frac{d}{dt}\left([\mathbf{I_{Es}}]_T\, e^{k_r^{\psi}t}\right) = f_{Am,Bis}k_{T,Bis}^{\psi}[\mathbf{E_{Bis}}]_{\mathrm{T,0}}\, e^{\left(k_r^{\psi}-k_{T,Bis}^{\psi}\right)t} + f_{Am}[\mathbf{E_{Bis}}]_{\mathbf{T,0}}\left(\frac{k_T^{\psi}k_{T,Bis}^{\psi}}{k_T^{\psi}-k_{T,Bis}^{\psi}}\right)\left(e^{\left(k_r^{\psi}-k_{T,Bis}^{\psi}\right)t} - e^{\left(k_r^{\psi}-k_T^{\psi}\right)t}\right)$$
$$+ f_{Am}k_T^{\psi}[\mathbf{E_m}]_{\mathbf{T,0}}\, e^{\left(k_r^{\psi}-k_T^{\psi}\right)t} \qquad \text{(S33)}$$

Given that no intermediate ester ought to be present at the outset of the reaction, the temporal evolution of $\mathbf{I_{Es}}$ is given by:

$$[\mathbf{I_{Es}}]_T = f_{Am,Bis}[\mathbf{E_{Bis}}]_{\mathrm{T,0}}\left(\frac{k_{T,Bis}^{\psi}}{k_r^{\psi}-k_{T,Bis}^{\psi}}\right)\left(e^{-k_{T,Bis}^{\psi}.t} - e^{-k_r^{\psi}.t}\right)$$
$$+ f_{Am}[\mathbf{E_{Bis}}]_{\mathbf{T,0}}\left(\frac{k_T^{\psi}k_{T,Bis}^{\psi}}{k_T^{\psi}-k_{T,Bis}^{\psi}}\right)\left(\left(\frac{\mathbf{1}}{k_r^{\psi}-k_{T,Bis}^{\psi}}\right)\left(e^{-k_{T,Bis}^{\psi}.t} - e^{-k_r^{\psi}.t}\right)\right.$$
$$\left. - \left(\frac{1}{k_r^{\psi}-k_T^{\psi}}\right)\left(e^{-k_T^{\psi}.t} - e^{-k_r^{\psi}.t}\right)\right) + f_{Am}[\mathbf{E_m}]_{\mathbf{T,0}}\left(\frac{k_T^{\psi}}{k_r^{\psi}-k_T^{\psi}}\right)\left(e^{-k_T^{\psi}.t} - e^{-k_r^{\psi}.t}\right) \qquad \text{(S34)}$$

where the constants $f_{Am,Bis}$ and f_{Am} reflect the empirical selectivity for aminolysis of $\mathbf{E_{Bis}}$ and $\mathbf{E_m}$, respectively:

$$f_{Am,Bis} = \frac{k_{Am,Bis}^{\psi}}{k_{T,Bis}^{\psi}} \qquad f_{Am} = \frac{k_{Am}^{\psi}}{k_T^{\psi}} \qquad \text{(S35)}$$

In practice, the mole fraction of the intermediate ester, $\mathbf{X_{\{IEs,T\}}}$, was calculated according to:

$$\mathbf{X}_{\{\mathbf{I_{Es}},\mathbf{T}\}}^{*} = f_{Am,Bis}\cdot c_{Bis,0}\left(\frac{k_{T,Bis}^{\psi}}{k_r^{\psi}-\mathrm{k}_{T,Bis}^{\psi}}\right)\left(e^{-k_{T,Bis}^{\psi}.t} - e^{-k_r^{\psi}.t}\right)$$
$$+ f_{Am}\cdot c_{Bis,0}\left(\frac{k_T^{\psi}k_{T,Bis}^{\psi}}{k_T^{\psi}-k_{T,Bis}^{\psi}}\right)\left(\left(\frac{1}{k_r^{\psi}-k_{T,Bis}^{\psi}}\right)\left(e^{-k_{T,Bis}^{\psi}.t} - e^{-k_r^{\psi}.t}\right)\right.$$
$$\left. - \left(\frac{1}{k_r^{\psi}-k_T^{\psi}}\right)\left(e^{-k_T^{\psi}.t} - e^{-k_r^{\psi}.t}\right)\right) + f_{Am}\cdot c_{m,0}\left(\frac{k_T^{\psi}}{k_r^{\psi}-k_T^{\psi}}\right)\left(e^{-k_T^{\psi}.t} - e^{-k_r^{\psi}.t}\right) \qquad \text{(S36)}$$

The mole fraction of the amide product, $\mathbf{X}_{\{\mathrm{Pam,T}\}}$, can be derived from this in a standard manner from, i.e.:

$$
\begin{aligned}
\frac{d\mathbf{X}^{*}_{\{\mathrm{P_{Am},T}\}}}{dt} &= k_r^{\psi}\,\mathbf{X}^{*}_{\{\mathrm{I_{Es},T}\}} \\
&= f_{Am,Bis}\cdot c_{Bis,0}k_r^{\psi}\left(\frac{k^{\Psi}_{T,Bis}}{k_r^{\psi}-k^{\Psi}_{T,Bis}}\right)\left(e^{-k^{\Psi}_{T,Bis}.t}-e^{-k_r^{\psi}.t}\right) \\
&+ f_{Am}\cdot c_{Bis,0}k_r^{\psi}\left(\frac{k_T^{\Psi}k^{\Psi}_{T,Bis}}{k_T^{\Psi}-k^{\Psi}_{T,Bis}}\right)\left(\left(\frac{1}{k_r^{\psi}-k^{\Psi}_{T,Bis}}\right)\left(e^{-k^{\Psi}_{T,Bis}.t}-e^{-k_r^{\psi}.t}\right)\right. \\
&\left.-\left(\frac{1}{k_r^{\psi}-k_T^{\Psi}}\right)\left(e^{-k_T^{\Psi}.t}-e^{-k_r^{\psi}.t}\right)\right)+f_{Am}\cdot c_{m,0}k_r^{\psi}\left(\frac{k_T^{\Psi}}{k_r^{\psi}-k_T^{\Psi}}\right)\left(e^{-k_T^{\Psi}.t}-e^{-k_r^{\psi}.t}\right) \quad \text{(S37)}
\end{aligned}
$$

Integration and rearrangement affords the following equation, which was used in practice to calculate the mole fraction of the amide product.

$$
\begin{aligned}
\mathbf{X}^{*}_{\{\mathrm{P_{Am}},T\}} &= f_{Am,Bis}\cdot c_{Bis,0}\left(\frac{1}{k_r^{\psi}-k^{\Psi}_{T,Bis}}\right)\left(k^{\Psi}_{T,Bis}\left(e^{-k_r^{\psi}.t}-1\right)-k_r^{\psi}\left(e^{-k^{\Psi}_{T,Bis}.t}-1\right)\right) \\
&+ f_{Am}\cdot c_{Bis,0}\left(\frac{1}{k_T^{\Psi}-k^{\Psi}_{T,Bis}}\right)\left(\left(\frac{k_T^{\Psi}}{k_r^{\psi}-k^{\Psi}_{T,Bis}}\right)\left(k^{\Psi}_{T,Bis}\left(e^{-k_r^{\psi}.t}-1\right)-k_r^{\psi}\left(e^{-k^{\Psi}_{T,Bis}.t}-1\right)\right)\right. \\
&\left.-\left(\frac{k^{\Psi}_{T,Bis}}{k_r^{\psi}-k_T^{\Psi}}\right)\left(k_T^{\Psi}\left(e^{-k_r^{\psi}.t}-1\right)-k_r^{\psi}\left(e^{-k_T^{\Psi}.t}-1\right)\right)\right) \\
&+ f_{Am}\cdot c_{m,0}\left(\frac{1}{k_r^{\psi}-k_T^{\Psi}}\right)\left(k_T^{\Psi}\left(e^{-k_r^{\psi}.t}-1\right)-k_r^{\psi}\left(e^{-k_T^{\Psi}.t}-1\right)\right) \quad \text{(S38)}
\end{aligned}
$$

pH-k^{Ψ} profile deconvolution

General considerations

The various second-order rate constants reported in the main text, corresponding to *specific bimolecular processes*, were estimated by deconvoluting experimentally obtained pH*-k^{Ψ} profiles. The pseudo first-order rate constants k^{Ψ} used in the construction of these profiles were themselves obtained by analytical kinetic analysis, *vide supra*, and deconvolution was realised by *non-linear* fitting of experimental values of k^{Ψ} to an appropriate kinetic model (using *OriginPro 2024b*); the nature of these models are summarized below. Raw values of k^{Ψ} were fit *directly,* with all parameters reported in the main text arising from *unweighted* fittings. Variance-weighted fits of k^{Ψ} as a function of pH* were also explored – see below for alternative sets of parameters obtained from such fits – but this fitting routine was deemed to be less appropriate than the unweighted routine on the basis that the *relative* uncertainties in k^{Ψ} are unknown and not likely to be constant at different pH* (see page 12).

All pH*-k^{Ψ} profiles are based on apparent (i.e., directly measured) pH values, and throughout the main text and SI, the notation ΔpK_a*(**X**) pertains to the difference between the (apparent) pK_a* of species **X** and the apparent pH*, i.e.:

$$\Delta pK_a^*(\mathbf{X}) = pK_a^*(\mathbf{X}) - \mathrm{pH}^* \quad \text{(S39)}$$

The apparent pK_a* of the conjugate acid of L-serinamide, pK_a*($^{\mathbf{L}}\mathbf{S}^+$), and its temperature dependence (dpK_a*/dT), were determined by NMR titration in both D_2O and H_2O using *apparent* pH* values, *vide infra*, and under conditions identical to those imposed during kinetic studies (*I* = 2.0 M, KCl). Measurements of ΔpK_a*($^{\mathbf{L}}\mathbf{S}^+$) = p$K_a$*($^{\mathbf{L}}\mathbf{S}^+$) – pH* therefore ought to predict the speciation of L-serinamide accurately for any given pH* and [$^{\mathbf{L}}\mathbf{S}$]$_T$. Similarly, all pK_a* values determined via the deconvolution of pH-k^{Ψ} profiles in this work are apparent; they do not represent strict thermodynamic values, but they are self-consistent with the apparent pK_a* of the conjugate acid of L-serinamide and the apparent pH* measurements used in this work.

The *apparent* autoprotolysis constants of H_2O (K_w*(H_2O)) and D_2O (K_w*(D_2O)) were not measured directly, and so there is a greater degree of uncertainty in the absolute values in the second-order rate constants for *saponification* determined by the fitting of pH*-k^{Ψ}_{Hyd} profiles*.* For simplicity, values for pK_w*(H_2O) over the range 10 – 40 °C were taken to be *equal* to the thermodynamic values at the corresponding temperature on the *molarity* scale, p$K_{w,c}$(H_2O), as reported by Covington and co-workers;[1] values for pK_w*(D_2O) were derived from Covington's p$K_{w,c}$(D_2O) values via:

$$pK_w^*(\mathrm{D_2O}) = pK_{w,c}(\mathrm{D_2O}) - 0.408 \quad \text{(S40)}$$

where the constant -0.408 reflects the correction required to account for the fact that pH* measurements made for D_2O solutions were done so using a pH meter calibrated with protiated buffers and reference filling solutions.[2]

Hydroxide concentrations ([OL⁻]; L = H or D) were then calculated according to:

$$[\text{OL}^-] = 10^{-\Delta pK_w^*(L_2O)} \tag{S41}$$

$$\Delta pK_w^*(L_2O) = \log_{10}\gamma_{OL^-} + pK_w^*(\text{L}_2\text{O}) - pH^* \tag{S42}$$

where the activity coefficient of hydroxide $\gamma_{OH^-} = \gamma_{OD^-} \approx 0.7$ was approximated from Harned assuming I = 2.0 M (KCl).[3] Similar values have been used in recent studies.[4]

k'$_{Am}$

The pH*-k'_{Am} profiles ($k'_{\text{Am}} = k^{\psi}_{\text{Am}}/[^{\text{L}}\mathbf{S}]_{\text{T}}$) in the main text were deconvoluted by assuming that k^{ψ}_{Am} comprises contributions from only two terms, including rates for the reaction of L-serinamide in its neutral state ($^{\text{L}}\mathbf{S}^0$) with: (i) $\mathbf{E_m}$ in its N-protonated (ammonium) state, $\mathbf{E_m}^+$ ($k_{\text{Am},+}$); and (ii) $\mathbf{E_m}$ in its unprotonated state, $\mathbf{E_m}^0$ ($k_{\text{Am},0}$). Note that the "+" and "0" superscripts for $\mathbf{E_m}^+$ and $\mathbf{E_m}^0$ do not pertain to the *overall charge* of the molecule, but to the formal charge on the α-amino substituent. Thus, the pH*-k'_{Am} profiles were deconvoluted by assuming that:

$$v_{Am} = \left(k_{Am,0}[\mathbf{E_m^0}] + k_{Am,+}[\mathbf{E_m^+}]\right)[^{\mathbf{L}}\mathbf{S^0}] \tag{S43}$$

The concentration of L-serinamide in its neutral state, $[^{\text{L}}\mathbf{S}^0]$, may be expressed in terms of the observed/phenomenological pK_a* of its conjugate acid, $^{\text{L}}\mathbf{S}^+$, and its analytical (total) concentration, $[^{\text{L}}\mathbf{S}]_{\text{T}}$, via:

$$K_a^*(^{\mathbf{L}}\mathbf{S}^+) = \frac{[^{\mathbf{L}}\mathbf{S^0}][\text{H}^+]}{[^{\mathbf{L}}\mathbf{S}^+]} \tag{S44}$$

$$\frac{[^{\mathbf{L}}\mathbf{S^0}]}{[^{\mathbf{L}}\mathbf{S}]_{\mathbf{T}}} = \frac{[^{\mathbf{L}}\mathbf{S^0}]}{[^{\mathbf{L}}\mathbf{S^0}] + [^{\mathbf{L}}\mathbf{S}^+]} = \frac{1}{1 + \frac{[\text{H}^+]}{K_a^*(^{\mathbf{L}}\mathbf{S}^+)}} = \frac{1}{1 + 10^{[pK_a^*(^{\mathbf{L}}\mathbf{S}^+) - pH^*]}} = \frac{1}{1 + 10^{\Delta pK_a^*(^{\mathbf{L}}\mathbf{S}^+)}} \tag{S45}$$

Similar expressions may be deduced for $\mathbf{E_m}^+$ and $\mathbf{E_m}^0$, such that

$$[\mathbf{E_m^0}] = \frac{[\mathbf{E_m}]_{\mathbf{T}}}{1 + 10^{[\Delta pK_a^*(\mathbf{E_m^+})]}} \tag{S46}$$

$$[\mathbf{E_m^+}] = [\mathbf{E_m}]_{\mathbf{T}} - [\mathbf{E_m^0}] = \frac{[\mathbf{E_m}]_{\mathbf{T}}.10^{[\Delta pK_a^*(\mathbf{E_m^+})]}}{1 + 10^{[\Delta pK_a^*(\mathbf{E_m^+})]}} \tag{S47}$$

The rate of amide formation from $\mathbf{E_m}$ is then:

$$v_{Am} = \frac{k_{Am,0} + k_{Am,+}10^{[\Delta pK_a^*(\mathbf{E_m^+})]}}{\left(1 + 10^{[\Delta pK_a^*(^{\mathbf{L}}\mathbf{S}^+)]}\right)\left(1 + 10^{[\Delta pK_a^*(\mathbf{E_m^+})]}\right)}[\mathbf{E_m}]_{\mathbf{T}}[^{\mathbf{L}}\mathbf{S}]_{\mathbf{T}} = k^{\psi}_{Am}[\mathbf{E_m}]_{\mathbf{T}} = k'_{Am}[\mathbf{E_m}]_{\mathbf{T}}[^{\mathbf{L}}\mathbf{S}]_{\mathbf{T}} \tag{S48}$$

By analogy to equation S4, the *overall second-orde*r rate constant, k'_{Am}, is:

$$k'_{Am} = \frac{k^{\psi}_{Am}}{[^{\mathbf{L}}\mathbf{S}]_{\mathbf{T}}} = \frac{k_{Am,0} + k_{Am,+}10^{[\Delta pK_a^*(\mathbf{E_m^+})]}}{\left(1 + 10^{[\Delta pK_a^*(^{\mathbf{L}}\mathbf{S}^+)]}\right)\left(1 + 10^{[\Delta pK_a^*(\mathbf{E_m^+})]}\right)} \tag{S49}$$

The second-order rate constants $k_{\mathrm{Am},+}$ and $k_{\mathrm{Am},0}$ are themselves composite rate constants reflecting weighted averages of the corresponding rate constants for the aminolysis of the 2'- and 3'-regioisomers of $\mathbf{E_m^0}$ ($\mathbf{E_{m,2'}^0}$; $\mathbf{E_{m,3'}^0}$) and $\mathbf{E_m^+}$ ($\mathbf{E_{m,2'}^+}$; $\mathbf{E_{m,3'}^+}$). Written explicitly in terms of the four possible rate terms, the overall rate of formation can be expressed as

$$v_{Am} = \left(k_{Am,3',0}\left[\mathbf{E_{m,3'}^0}\right] + k_{Am,3',+}\left[\mathbf{E_{m,3'}^+}\right] + k_{Am,2',0}\left[\mathbf{E_{m,2'}^0}\right] + k_{Am,2',+}\left[\mathbf{E_{m,2'}^+}\right]\right)[{}^{\mathbf{L}}\mathbf{S^0}] \quad \text{(S50)}$$

where the concentrations of $[\mathbf{E_m^0}]$ and $[\mathbf{E_m^+}]$ reflect the sum of the two regioisomers, i.e.

$$[\mathbf{E_m^0}] = \left[\mathbf{E_{m,2'}^0}\right] + \left[\mathbf{E_{m,3'}^0}\right] \quad \text{(S51)}$$

$$[\mathbf{E_m^+}] = \left[\mathbf{E_{m,2'}^+}\right] + \left[\mathbf{E_{m,3'}^+}\right] \quad \text{(S52)}$$

The relative stabilities of the 2' and 3' regioisomers of $\mathbf{E_m}$, in each ionization state, may be expressed in terms of equilibrium constants $K_{\mathrm{iso},+}$ and $K_{\mathrm{iso},0}$:

$$K_{iso,+} = \frac{\left[\mathbf{E_{m,3'}^+}\right]}{\left[\mathbf{E_{m,2'}^+}\right]} \qquad K_{iso,0} = \frac{\left[\mathbf{E_{m,3'}^0}\right]}{\left[\mathbf{E_{m,2'}^0}\right]} \quad \text{(S53)}$$

such that:

$$\left[\mathbf{E_{m,2'}^0}\right] = [\mathbf{E_m^0}] - \left[\mathbf{E_{m,3'}^0}\right] = \frac{[\mathbf{E_m^0}]}{1 + K_{iso,0}} \quad \text{(S54)}$$

$$\left[\mathbf{E_{m,2'}^+}\right] = [\mathbf{E_m^+}] - \left[\mathbf{E_{m,3'}^+}\right] = \frac{[\mathbf{E_m^+}]}{1 + K_{iso,+}} \quad \text{(S55)}$$

This, in turn, gives rise to the following equations for the composite second-order rate constants $k_{\mathrm{Am},0}$ and $k_{\mathrm{Am},+}$:

$$v_{Am} = \left\{\left(k_{Am,3',0}\left(\frac{K_{iso,0}}{1 + K_{iso,0}}\right) + k_{Am,2',0}\left(\frac{1}{1 + K_{iso,0}}\right)\right)[\mathbf{E_m^0}] + \left(k_{Am,3',+}\left(\frac{K_{iso,+}}{1 + K_{iso,+}}\right) + k_{Am,2',+}\left(\frac{1}{1 + K_{iso,+}}\right)\right)[\mathbf{E_m^+}]\right\}[{}^{\mathbf{L}}\mathbf{S^0}] \quad \text{(S56)}$$

$$k_{Am,0} = \frac{k_{Am,2',0} + k_{Am,3',0}K_{iso,0}}{1 + K_{iso,0}} \quad \text{(S57)}$$

$$k_{Am,+} = \frac{k_{Am,2',+} + k_{Am,3',+}K_{iso,+}}{1 + K_{iso,+}} \quad \text{(S58)}$$

Similar manipulations can be used to demonstrate the relationship between the observed/phenomenological acidity constant of $\mathbf{E_m^+}$, $K_a^*(\mathbf{E_m^+})$, and the microscopic constants for the 2'- and 3'-regioisomers ($K_a^*(\mathbf{E_{m,2'}^+})$, $K_a^*(\mathbf{E_{m,2'}^+})$).

$$K_a^*(\mathbf{E_m^+}) = \frac{[\mathbf{E_m^0}][\mathrm{H^+}]}{[\mathbf{E_m^+}]} = \frac{\left(\left[\mathbf{E_{m,2'}^0}\right] + \left[\mathbf{E_{m,3'}^0}\right]\right)[\mathrm{H^+}]}{\left[\mathbf{E_{m,2'}^+}\right] + \left[\mathbf{E_{m,3'}^+}\right]} = \frac{(1 + K_{iso,0})K_a^*\left(\mathbf{E_{m,2'}^+}\right)K_a^*\left(\mathbf{E_{m,3'}^+}\right)}{K_a\left(\mathbf{E_{m,3'}^+}\right) + K_{iso,0}K_a^*\left(\mathbf{E_{m,2'}^+}\right)} \quad \text{(S59)}$$

The observed/phenomenological acidity constant of $\mathbf{E_m}^+$, pK_a*($\mathbf{E_m}^+$) = 7.1 (20°C, D_2O, I = 2.0 M) was determined by non-linear fitting of the experimental pH*-k'_{Am} profile to equation S49.

To determine pK_a*($\mathbf{E_{m,2'}}^+$) and pK_a*($\mathbf{E_{m,3'}}^+$)) independently, the 2'/3' speciation of $\mathbf{E_m}$ was analysed as a function of pH*(20°C) using experimental speciation data from all kinetic runs (> 20) under pertinent conditions (20°C, D_2O, I = 2.0 M; **Figure S3**). Qualitatively, it was found that the preponderance of the 3'-regioisomer was more significant at lower pH*(20°C), with the fractional 3' speciation, $f_{\{Em,3'\}}$, decreasing from around 70 % at pH*(20°C) < 5.5 to ca 55 % at pH*(20°C) > 8.5. This suggests that the 2'-regioisomer of $\mathbf{E_m}^+$ is more acidic than the 3'-regioisomer.

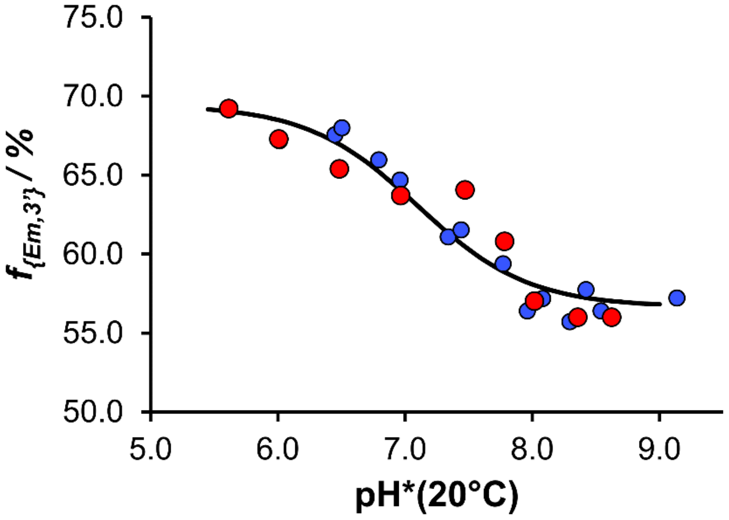

Figure S3: Experimental fractional 3'-speciation of the aminoacyl monoester **MepA-L-PheF** ($\mathbf{E_m}$), $f_{\{Em,3'\}}$, as a function of pH*(20°C) in D_2O ($[\mathbf{^LS}]_T$ = 600 – 2000 mM; I = 2.0 M, KCl). Red data points: experimental speciation data obtained from kinetic runs initiated with **MepA-(PheF)$_2$** ($\mathbf{E_{bis}}$), where **MepA-L-PheF** ($\mathbf{E_m}$) was observed as an intermediate. Blue data points: experimental speciation data obtained from kinetic runs initiated with **MepA-L-PheF** ($\mathbf{E_m}$). Black line is calculated fractional 3'-speciation with parameters determined by non-linear regression (see text). pK_a*($\mathbf{E_{m,2'}}^+$) = 6.95, pK_a*($\mathbf{E_{m,3'}}^+$) = 7.19, pK_a*($\mathbf{E_m}^+$) = 7.1, $K_{iso,0}$ = 1.3 and $K_{iso,+}$ = 2.3.

Quantitative deconvolution was achieved by noting that the fractional 3'-speciation observed by NMR is:

$$f_{\{E_{m,3'}\}} = \frac{[\mathbf{E_{m,3'}}]_T}{[\mathbf{E_{m,3'}}]_T + [\mathbf{E_{m,2'}}]_T} = \frac{[\mathbf{E^0_{m,3'}}] + [\mathbf{E^+_{m,3'}}]}{[\mathbf{E^0_{m,3'}}] + [\mathbf{E^+_{m,3'}}] + [\mathbf{E^0_{m,2'}}] + [\mathbf{E^+_{m,2'}}]} = \frac{1 + \frac{[\mathbf{E^+_{m,3'}}]}{[\mathbf{E^0_{m,3'}}]}}{1 + \frac{[\mathbf{E^+_{m,3'}}]}{[\mathbf{E^0_{m,3'}}]} + \frac{[\mathbf{E^0_{m,2'}}]}{[\mathbf{E^0_{m,3'}}]} + \frac{[\mathbf{E^+_{m,2'}}]}{[\mathbf{E^0_{m,3'}}]}} \quad (S60)$$

$$f_{\{E_{m,3'}\}} = \frac{1 + \frac{[H^+]}{K_a(\mathbf{E^+_{m,3'}})}}{1 + \frac{[H^+]}{K_a(\mathbf{E^+_{m,3'}})} + \frac{1}{K_{iso,0}} + \frac{[H^+]}{K_{iso,0}.K_a(\mathbf{E^+_{m,2'}})}} = \frac{1 + 10^{[\Delta pK_a^*(\mathbf{E^+_{m,3'}})]}}{1 + 10^{[\Delta pK_a^*(\mathbf{E^+_{m,3'}})]} + 10^{[pK_{iso,0}]} + 10^{[\Delta pK_a^*(\mathbf{E^+_{m,2'}}) + pK_{iso,0}]}} \quad (S61)$$

and then by fitting experimental speciation data as a function of pH* to equation S61, with pK_a*($\mathbf{E_{m,2'}}^+$) and pK_{iso} floated as variable parameters and pK_a*($\mathbf{E_{m,3'}}^+$) constrained according to the equation below, where pK_a*($\mathbf{E_m}^+$) = 7.1 was obtained from kinetic data, vide supra.

$$pK_a^*(\mathbf{E}_{\mathbf{m},3'}^+) = -\log_{10} K_a^*(\mathbf{E}_{\mathbf{m},3'}^+) = -\log_{10}\left(\frac{K_{iso,0}K_a^*(\mathbf{E}_{\mathbf{m},2'}^+)K_a^*(\mathbf{E}_{\mathbf{m}}^+)}{(1+K_{iso,0})K_a^*(\mathbf{E}_{\mathbf{m},2'}^+) - K_a^*(\mathbf{E}_{\mathbf{m}}^+)}\right) \quad \text{(S62)}$$

The result of the non-linear regression is shown in **Figure S3**, with pK_a*($\mathbf{E_{m,2'}}^+$) = 6.95, pK_a*($\mathbf{E_{m,3'}}^+$) = 7.19, $K_{iso,0}$ = 1.3 and $K_{iso,+}$ = 2.3. The 2'-regioisomer of $\mathbf{E_m}$ thus appears to be 0.24 pK units more acidic than the 3'-regioisomer (20°C, D_2O, I = 2.0 M).

Though not determined explicitly, a similar relationship would be expected in H_2O.

k^{Ψ}_{Hyd}

The pH*-k^{Ψ}_{Hyd} profiles in the main text was deconvoluted by assuming that k^{Ψ}_{Hyd} comprises contributions from two terms, including terms for the *saponification* – reaction with hydroxide (OL^-; L = H or D) – of $\mathbf{E_m}$ in its N-protonated (ammonium) state, $\mathbf{E_m}^+$ ($k_{OL^-,+}$), and in its unprotonated state, $\mathbf{E_m}^0$ ($k_{OL^-,0}$). Note that the "+" and "0" superscripts for $\mathbf{E_m}^+$ and $\mathbf{E_m}^0$ do not pertain to the *overall charge* of the molecule, but to the formal charge on the α-amino substituent. Thus, the pH*-k^{Ψ}_{Hyd} profiles were deconvoluted by assuming that:

$$v_{Hyd} = \left(k_{OL^-,0}[\mathbf{E}_\mathbf{m}^\mathbf{0}] + k_{OL^-,+}[\mathbf{E}_\mathbf{m}^+]\right)[\mathrm{OL}^-] \quad \text{(S63)}$$

Given expressions for [$\mathbf{E_m}^0$] and [$\mathbf{E_m}^+$] (vide supra) and [OL^-] (L = H or D):

$$[\mathbf{E}_\mathbf{m}^\mathbf{0}] = \frac{[\mathbf{E}_\mathbf{m}]_\mathbf{T}}{1 + 10^{[\Delta pK_a^*(\mathbf{E}_\mathbf{m}^+)]}} \quad \text{(S64)}$$

$$[\mathbf{E}_\mathbf{m}^+] = \mathbf{E}_\mathbf{m}]_\mathbf{T} - [\mathbf{E}_\mathbf{m}^\mathbf{0}] = \frac{[\mathbf{E}_\mathbf{m}]_\mathbf{T}.\,10^{[\Delta pK_a^*(\mathbf{E}_\mathbf{m}^+)]}}{1 + 10^{[\Delta pK_a^*(\mathbf{E}_\mathbf{m}^+)]}} \quad \text{(S65)}$$

$$[\mathrm{OL}^-] = 10^{-\Delta pK_w^*(L_2O)} \quad \text{(S66)}$$

$$\Delta pK_w^*(L_2O) = \log_{10}\gamma_{OL^-} + pK_w^*(L_2O) - pH^* \quad \text{(S67)}$$

The rate of amino acid formation from $\mathbf{E_m}$ can then be expressed as:

$$v_{Hyd} = k_{Hyd}^{\psi}[\mathbf{E}_\mathbf{m}]_\mathbf{T} \quad \text{(S68)}$$

where the pseudo first-order rate constant for hydrolysis, k^{Ψ}_{Hyd}, is:

$$k_{Hyd}^{\psi} = \frac{10^{-\Delta pK_w^*(L_2O)}\left(k_{OL^-,0} + k_{OL^-,+}10^{[\Delta pK_a^*(\mathbf{E}_\mathbf{m}^+)]}\right)}{1 + 10^{[\Delta pK_a^*(\mathbf{E}_\mathbf{m}^+)]}} = \frac{k_{OL^-,0} + k_{OL^-,+}10^{[\Delta pK_a^*(\mathbf{E}_\mathbf{m}^+)]}}{10^{\Delta pK_w^*(L_2O)}\left(1 + 10^{[\Delta pK_a^*(\mathbf{E}_\mathbf{m}^+)]}\right)} \quad \text{(S69)}$$

The second-order rate constants $k_{OL\text{-},0}$, $k_{OL\text{-},+}$ and $k_{L2O\text{-},+}$ are weighted averages of the individual rate constants for the saponification/water hydrolysis of the 2'- and 3'-regioisomers of $\mathbf{E_m}$; see section above for detailed discussion.

Though not described in the main text explicitly, a more complex model, including a term for the spontaneous hydrolysis (*neutral water reaction*) of $\mathbf{E_m}^+$ ($k_{L2O,+}$), was also explored. Such a model would, in theory, provide a possible explanation for the apparent deterioration in the selectivity for aminolysis at lower pH*, since with such a pathway the pseudo first-order rate constant for hydrolysis would evolve with pH* according to

$$k_{Hyd}^{\psi} = \frac{10^{-\Delta pK_w^*(L_2O)}\left(k_{OL^-,0} + k_{OL^-,+}10^{[\Delta pK_a^*(\mathbf{E_m^+})]}\right) + k_{L_2O,+}10^{[\Delta pK_a^*(\mathbf{E_m^+})]}}{1 + 10^{[\Delta pK_a^*(\mathbf{E_m^+})]}} \qquad (S70)$$

where the second-order rate constant for neutral water hydrolysis of $\mathbf{E_m}^+$, $k'_{L2O\text{-},+}$, would be

$$k'_{L_2O,+} = \frac{k_{L_2O,+}}{[L_2O]}\ ; \qquad [L_2O] \approx 55\ \mathrm{M} \qquad (S71)$$

k'Am,Bis

The pH*-$k^{\Psi}_{Am,Bis}$ profile in the main text was deconvoluted by assuming that $k^{\Psi}_{Am,Bis}$ comprises contributions from three terms, including rates for the reaction of L-serinamide in its neutral state ($^{\mathbf{L}}\mathbf{S}^0$) with: (i) $\mathbf{E_{Bis}}$ in its bis-N-protonated state, $\mathbf{E_{bis}}^{++}$ ($k_{Am,Bis,++}$); (ii) with $\mathbf{E_{Bis}}$ in its mono-N-protonated state, $\mathbf{E_{bis}}^+$ ($k_{Am,Bis,+}$); and (iii) $\mathbf{E_{Bis}}$ in its unprotonated state, $\mathbf{E_{Bis}}^0$ ($k_{Am,Bis,0}$). Note that the "++", "+" and "0" superscripts for $\mathbf{E_{Bis}}^{++}$, $\mathbf{E_{Bis}}^+$ and $\mathbf{E_{Bis}}^0$ do not pertain to the *overall charge* of the molecule, but to the combined formal charge of the two α-amino substituents. Thus, the pH*-$k^{\Psi}_{Am,Bis}$ profiles were deconvoluted by assuming that:

$$v_{Am,Bis} = \left(k_{Am,Bis,0}[\mathbf{E^0_{Bis}}] + k_{Am,Bis,+}[\mathbf{E^+_{Bis}}] + k_{Am,Bis,++}[\mathbf{E^{++}_{Bis}}]\right)[^{\mathbf{L}}\mathbf{S^0}] \qquad (S72)$$

Given observed/phenomenological pK_a* values of $\mathbf{E_{Bis}}^{++}$ and $\mathbf{E_{Bis}}^+$, and pK_a*($^{\mathbf{L}}\mathbf{S}^+$), as defined by:

$$K_a^*(\mathbf{E^{++}_{Bis}}) = \frac{[\mathbf{E^+_{Bis}}][H^+]}{[\mathbf{E^{++}_{Bis}}]} \qquad K_a^*(\mathbf{E^+_{Bis}}) = \frac{[\mathbf{E^0_{Bis}}][H^+]}{[\mathbf{E^+_{Bis}}]} \qquad (S73)$$

$$K_a^*(\mathbf{E^+_{Bis}})K_a^*(\mathbf{E^{++}_{Bis}}) = \frac{[\mathbf{E^0_{Bis}}][H^+]^2}{[\mathbf{E^{++}_{Bis}}]} \qquad (S74)$$

it can be shown that:

$$[\mathbf{E^0_{Bis}}] = \frac{[\mathbf{E_{Bis}}]_\mathbf{T}}{1 + \dfrac{[H^+]}{K_a^*(\mathbf{E^+_{Bis}})} + \dfrac{[H^+]^2}{K_a^*(\mathbf{E^+_{Bis}})K_a^*(\mathbf{E^{++}_{Bis}})}} = \frac{[\mathbf{E_{Bis}}]_\mathbf{T}}{1 + 10^{[\Delta pK_a^*(\mathbf{E^+_{Bis}})]} + 10^{[\Delta pK_a^*(\mathbf{E^+_{Bis}}) + \Delta pK_a^*(\mathbf{E^{++}_{Bis}})]}} \qquad (S75)$$

and in turn that:

$$[\mathbf{E}_{\mathbf{Bis}}^{+}] = \frac{[\mathbf{E}_{\mathbf{Bis}}]_{\mathbf{T}}.\,10^{[\Delta pK_a^*(\mathbf{E}_{\mathbf{Bis}}^{+})]}}{1 + 10^{[\Delta pK_a^*(\mathbf{E}_{\mathbf{Bis}}^{+})]} + 10^{[\Delta pK_a^*(\mathbf{E}_{\mathbf{Bis}}^{+}) + \Delta pK_a^*(\mathbf{E}_{\mathbf{Bis}}^{++})]}} \tag{S76}$$

$$[\mathbf{E}_{\mathbf{Bis}}^{++}] = \frac{[\mathbf{E}_{\mathbf{Bis}}]_{\mathbf{T}}.\,10^{[\Delta pK_a^*(\mathbf{E}_{\mathbf{Bis}}^{+}) + \Delta pK_a^*(\mathbf{E}_{\mathbf{Bis}}^{++})]}}{1 + 10^{[\Delta pK_a^*(\mathbf{E}_{\mathbf{Bis}}^{+})]} + 10^{[\Delta pK_a^*(\mathbf{E}_{\mathbf{Bis}}^{+}) + \Delta pK_a^*(\mathbf{E}_{\mathbf{Bis}}^{++})]}} \tag{S77}$$

The total rate of (formal) aminolysis of $\mathbf{E}_{\mathbf{Bis}}$, $v_{Am,Bis}$, can then be expressed as:

$$v_{Am,Bis} = k_{Am,Bis}^{\psi}[\mathbf{E}_{\mathbf{Bis}}]_{\mathbf{T}} = k'_{Am,Bis}[\mathbf{E}_{\mathbf{Bis}}]_{\mathbf{T}}[{}^{\mathbf{L}}\mathbf{S}]_{\mathbf{T}} \tag{S78}$$

$$k'_{Am,Bis} = \frac{k_{Am,Bis,0} + k_{Am,Bis,+}10^{[\Delta pK_a^*(\mathbf{E}_{\mathbf{Bis}}^{+})]} + k_{Am,Bis,++}10^{[\Delta pK_a^*(\mathbf{E}_{\mathbf{Bis}}^{+}) + \Delta pK_a^*(\mathbf{E}_{\mathbf{Bis}}^{++})]}}{\left(1 + 10^{[\Delta pK_a^*(\mathbf{E}_{\mathbf{Bis}}^{+})]} + 10^{[\Delta pK_a^*(\mathbf{E}_{\mathbf{Bis}}^{+}) + \Delta pK_a^*(\mathbf{E}_{\mathbf{Bis}}^{++})]}\right)\left(1 + 10^{\Delta pK_a^*({}^{\mathbf{L}}\mathbf{S}^{+})}\right)} \tag{S79}$$

Several subtleties regarding the constants in this equation should be noted. The second-order rate constants $k_{\mathrm{Am,Bis,0}}$ and $k_{\mathrm{Am,Bis,++}}$, for example, are necessarily equal to *sums* of the respective rate constants for reaction at the 2'- and 3'-aminoacyl fragments, i.e.,

$$k_{Am,Bis,0} = k_{Am,Bis,0,2'} + k_{Am,Bis,0,3'} \tag{S80}$$

$$k_{Am,Bis,++} = k_{Am,Bis,++,2'} + k_{Am,Bis,++,3'} \tag{S81}$$

where $k_{\mathrm{Am,Bis,0,x'}}$ denotes the second-order rate constant for the reaction of neutral L-serinamide at the x'-aminoacyl fragment in $\mathbf{E}_{\mathbf{Bis}}^{\mathbf{0}}$, and $k_{\mathrm{Am,Bis,++,x'}}$ is defined equivalent for $\mathbf{E}_{\mathbf{Bis}}^{++}$. The rate constant $k_{\mathrm{Am,Bis,+}}$, meanwhile, is well approximated as a *weighted average* – not a sum – of $k_{\mathrm{Am,Bis,2+}}$ and $k_{\mathrm{Am,Bis,3+}}$, with the weighting dictated by the relative acidities of the two tautomers of $\mathbf{E}_{\mathbf{Bis}}^{+}$. These tautomers are denoted: (i) $\mathbf{E}_{\mathbf{Bis,2'+}}^{+}$, corresponding to the tautomer in which the 2'-aminoacyl fragment is protonated and the 3'-aminoacyl fragment is not; and (ii) $\mathbf{E}_{\mathbf{Bis,3'+}}^{+}$, corresponding to the tautomer in which the 3'-aminoacyl fragment protonated and the 2'-aminoacyl fragment is not.

$$[\mathbf{E}_{\mathbf{Bis}}^{+}] = [\mathbf{E}_{\mathbf{Bis,2'+}}^{+}] + [\mathbf{E}_{\mathbf{Bis,3'+}}^{+}] \tag{S82}$$

If the reactivity of the unprotonated aminoacyl fragment each tautomer of $\mathbf{E}_{\mathbf{Bis}}^{+}$ is neglected, one can express the rate of aminolysis of $\mathbf{E}_{\mathbf{Bis}}$, $v_{\mathrm{Am,Bis}}$, more precisely as:

$$v_{Am,Bis} = \left(k_{Am,Bis,0}[\mathbf{E}_{\mathbf{Bis}}^{\mathbf{0}}] + k_{Am,Bis,2'+}[\mathbf{E}_{\mathbf{Bis,2'+}}^{+}] + k_{Am,Bis,3'+}[\mathbf{E}_{\mathbf{Bis,3'+}}^{+}] + k_{Am,Bis,++}[\mathbf{E}_{\mathbf{Bis}}^{++}]\right)[{}^{\mathbf{L}}\mathbf{S}^{\mathbf{0}}] \tag{S83}$$

Defining the relative stabilities of $\mathbf{E}_{\mathbf{Bis,3'+}}^{+}$ and $\mathbf{E}_{\mathbf{Bis,2'+}}^{+}$ as K_{taut} then leads to:

$$K_{taut} = \frac{[\mathbf{E}_{\mathbf{Bis,3'+}}^{+}]}{[\mathbf{E}_{\mathbf{Bis,2'+}}^{+}]} \tag{S84}$$

$$[\mathbf{E}_{\mathbf{Bis,2'+}}^{+}] = \frac{[\mathbf{E}_{\mathbf{Bis}}^{+}]}{1 + K_{taut}} \qquad [\mathbf{E}_{\mathbf{Bis,3'+}}^{+}] = \frac{K_{taut}[\mathbf{E}_{\mathbf{Bis}}^{+}]}{1 + K_{taut}} \tag{S85}$$

Such that the $k_{\mathrm{Am,Bis,+}}$ is:

$$k_{Am,Bis+} = \frac{k_{Am,Bis,2'+} + k_{Am,Bis,3'+}K_{taut}}{1 + K_{taut}} \tag{S86}$$

The observed/phenomenological acidity constants K_a*($\mathbf{E_{Bis}}^{++}$) and K_a*($\mathbf{E_{Bis}}^{+}$) – as obtained from kinetic fitting – can be expressed in terms of the microscopic acidity constants of these two tautomers, K_a*($\mathbf{E_{Bis,3'+}}^{+}$) and K_a*($\mathbf{E_{Bis,2'+}}^{+}$), via:

$$K_a^*(\mathbf{E_{Bis}^+}) = \frac{[\mathbf{E_{Bis}^0}][\mathrm{H^+}]}{[\mathbf{E_{Bis}^+}]} = \frac{[\mathbf{E_{Bis}^0}][\mathrm{H^+}]}{[\mathbf{E_{Bis,2'+}^+}] + [\mathbf{E_{Bis,3'+}^+}]} \tag{S87}$$

$$\frac{1}{K_a^*(\mathbf{E_{Bis}^+})} = \frac{1}{K_a^*(\mathbf{E_{Bis,2'+}^+})} + \frac{1}{K_a^*(\mathbf{E_{Bis,3'+}^+})} \tag{S88}$$

$$10^{pK_a^*(\mathbf{E_{Bis}^+})} = 10^{pK_a^*(\mathbf{E_{Bis,2'+}^+})} + 10^{pK_a^*(\mathbf{E_{Bis,3'+}^+})} \tag{S89}$$

Similarly, one can define two distinct microscopic acidity constants for $\mathbf{E_{Bis}}^{++}$: (i) K_a*($\mathbf{E_{Bis,3'}}^{++}$), corresponding to the *loss* of a proton from the *3'-aminoacyl* fragment to form $\mathbf{E_{Bis,2'+}}^{+}$; and (ii) K_a*($\mathbf{E_{Bis,2'}}^{++}$), corresponding to the *loss* of a proton from the *2'-aminoacyl* fragment to form $\mathbf{E_{Bis,3'+}}^{+}$. Accordingly,

$$K_a^*(\mathbf{E_{Bis,3'}^{++}}) = \frac{[\mathbf{E_{Bis,2'+}^+}][\mathrm{H^+}]}{[\mathbf{E_{Bis}^{++}}]} \qquad K_a^*(\mathbf{E_{Bis,2'}^{++}}) = \frac{[\mathbf{E_{Bis,3'+}^+}][\mathrm{H^+}]}{[\mathbf{E_{Bis}^{++}}]} \tag{S90}$$

$$K_a^*(\mathbf{E_{Bis}^{++}}) = \frac{([\mathbf{E_{Bis,2'+}^+}] + [\mathbf{E_{Bis,3'+}^+}])[\mathrm{H^+}]}{[\mathbf{E_{Bis}^{++}}]} = K_a^*(\mathbf{E_{Bis,3'}^{++}}) + K_a^*(\mathbf{E_{Bis,2'}^{++}}) \tag{S91}$$

$$10^{-pK_a^*(\mathbf{E_{Bis}^{++}})} = 10^{-pK_a^*(\mathbf{E_{Bis,3'}^{++}})} + 10^{-pK_a^*(\mathbf{E_{Bis,2'}^{++}})} \tag{S92}$$

These expressions were used (see later section) to calculate the microscopic acidity constants pK_a*($\mathbf{E_{Bis,3'}}^{++}$) and pK_a*($\mathbf{E_{Bis,2'}}^{++}$) via NMR chemical shift analysis as a function of pH*.

$k^{\Psi}_{Hyd,Bis}$

The pH*-$k^{\Psi}_{\mathrm{Hyd,Bis}}$ profile in the main text was deconvoluted by assuming that $k^{\Psi}_{\mathrm{Hyd,Bis}}$ comprises contributions from three terms, including rates for the *saponification* – reaction with hydroxide (OL^-; L = H or D) – of $\mathbf{E_{Bis}}$ in its: (i) bis-N-protonated state $\mathbf{E_{bis}}^{++}$ ($k_{\mathrm{OL^-,Bis,++}}$); (ii) its mono-N-protonated state $\mathbf{E_{bis}}^{+}$ ($k_{\mathrm{OL^-,Bis,+}}$); and (iii) its unprotonated state $\mathbf{E_{Bis}}^{0}$ ($k_{\mathrm{OH^-,Bis,0}}$). Note that the "++", "+" and "0" superscripts for $\mathbf{E_{Bis}}^{++}$, $\mathbf{E_{Bis}}^{+}$ and $\mathbf{E_{Bis}}^{0}$ do not pertain to the *overall charge* of the molecule, but to the combined formal charge of the two α-amino substituents. Thus, the pH*-$k^{\Psi}_{\mathrm{Hyd,Bis}}$ profiles were deconvoluted by assuming that:

$$v_{Hyd,Bis} = \left(k_{OL^-,Bis,0}[\mathbf{E_{Bis}^0}] + k_{OL^-,Bis,+}[\mathbf{E_{Bis}^+}] + k_{OL^-,Bis,++}[\mathbf{E_{Bis}^{++}}]\right)[\mathbf{OL^-}] \tag{S93}$$

Given standard equations for the phenomenological speciation of $\mathbf{E_{Bis}}$ and the hydroxide concentration as a function of pH*:

$$[\mathbf{E^0_{Bis}}] = \frac{[\mathbf{E_{Bis}}]_\mathbf{T}}{1 + 10^{[\Delta pK_a^*(\mathbf{E^+_{Bis}})]} + 10^{[\Delta pK_a^*(\mathbf{E^+_{Bis}}) + \Delta pK_a^*(\mathbf{E^{++}_{Bis}})]}} \tag{S94}$$

$$[\mathbf{E^+_{Bis}}] = \frac{[\mathbf{E_{Bis}}]_\mathbf{T}\ 10^{[\Delta pK_a^*(\mathbf{E^+_{Bis}})]}}{1 + 10^{[\Delta pK_a^*(\mathbf{E^+_{Bis}})]} + 10^{[\Delta pK_a^*(\mathbf{E^+_{Bis}}) + \Delta pK_a^*(\mathbf{E^{++}_{Bis}})]}} \tag{S95}$$

$$[\mathbf{E^{++}_{Bis}}] = \frac{[\mathbf{E_{Bis}}]_\mathbf{T}\ 10^{[\Delta pK_a^*(\mathbf{E^+_{Bis}}) + \Delta pK_a^*(\mathbf{E^{++}_{Bis}})]}}{1 + 10^{[\Delta pK_a^*(\mathbf{E^+_{Bis}})]} + 10^{[\Delta pK_a^*(\mathbf{E^+_{Bis}}) + \Delta pK_a^*(\mathbf{E^{++}_{Bis}})]}} \tag{S96}$$

$$[\mathrm{OL}^-] = 10^{-\Delta pK_w^*(L_2O)} \tag{S97}$$

$$\Delta pK_w^*(L_2O) = \log_{10}\gamma_{OL^-} + pK_w^*(L_2O) - pH^* \tag{S98}$$

the total rate of hydrolysis of $\mathbf{E_{Bis}}$ can be expressed in the form:

$$v_{Hyd,Bis} = k^{\psi}_{Hyd,Bis}[\mathbf{E_{Bis}}]_\mathbf{T} \tag{S99}$$

where the pseudo first-order rate constant $k^{\Psi}_{\mathrm{Hyd,Bis}}$ is:

$$k^{\psi}_{Hyd,Bis} = \frac{k_{OL^-,Bis,0} + k_{OL^-,Bis,+}\ 10^{[\Delta pK_a^*(\mathbf{E^+_{Bis}})]} + k_{OL^-,Bis,++}\ 10^{[\Delta pK_a^*(\mathbf{E^+_{Bis}}) + \Delta pK_a^*(\mathbf{E^{++}_{Bis}})]}}{10^{\Delta pK_w^*(L_2O)}\left(1 + 10^{[\Delta pK_a^*(\mathbf{E^+_{Bis}})]} + 10^{[\Delta pK_a^*(\mathbf{E^+_{Bis}}) + \Delta pK_a^*(\mathbf{E^{++}_{Bis}})]}\right)} \tag{S100}$$

The second-order rate constant $k_{\mathrm{OL-,Bis,++}}$ is equal to the sums of the rate constants for saponification/water hydrolysis at the 2'- and 3'-aminoacyl fragments in $\mathbf{E_{Bis}}^{++}$; $k_{\mathrm{OH-,Bis,0}}$ is equal to the sum of the rate constants for saponification of the 2'- and 3'-aminoacyl fragments in $\mathbf{E_{Bis}}^{\mathbf{0}}$. $k_{\mathrm{OL-,Bis,+}}$ is approximately equal to a *weighted average* of the rate constants for the saponification of the two tautomers of $\mathbf{E_{Bis}}^+$ ($\mathbf{E_{Bis,3'+}}^+$ and $\mathbf{E_{Bis,2'+}}^+$); see previous section for details.

If a spontaneous hydrolysis (neutral water reaction) term is included for the most reactive species $\mathbf{E_{Bis}}^{++}$, $k_{\mathrm{L2O,Bis,++}}$, then $k^{\Psi}_{\mathrm{Hyd,Bis}}$ will instead evolve with pH* according to:

$$k^{\psi}_{Hyd} = \frac{N_{OL^-}10^{-\Delta pK_w^*(L_2O)} + N_{L_2O}}{1 + 10^{[\Delta pK_a^*(\mathbf{E^+_{Bis}})]} + 10^{[\Delta pK_a^*(\mathbf{E^+_{Bis}}) + \Delta pK_a^*(\mathbf{E^{++}_{Bis}})]}} \tag{S100}$$

$$N_{OL^-} = k_{OL^-,Bis,0} + k_{OL^-,Bis,+}\ 10^{[\Delta pK_a^*(\mathbf{E^+_{Bis}})]} + k_{OL^-,Bis,++}\ 10^{[\Delta pK_a^*(\mathbf{E^+_{Bis}}) + \Delta pK_a^*(\mathbf{E^{++}_{Bis}})]} \tag{$S101$}$$

$$N_{L_2O} = k_{L_2O,Bis,++}\ 10^{[\Delta pK_a^*(\mathbf{E^+_{Bis}}) + \Delta pK_a^*(\mathbf{E^{++}_{Bis}})]} \tag{$S102$}$$

pK_a determination by NMR chemical shift analysis*

$^1H/^{13}C\{^1H\}$ NMR titration: L-serinamide

The apparent pK_a* of the conjugate acid of L-serinamide, pK_a*(^{L}**S**$^+$) and its temperature dependence (dpK_a*/dT; 10°C, 25°C, 50°C) were determined in D_2O by standard $^1H/^{13}C\{^1H\}$ NMR titrations, using conditions that were self-consistent with all kinetics experiments (i.e., I = 2.0 M, KCl). The apparent pK_a*(^{L}**S**$^+$) was also determined in H_2O, with 5% D_2O, at one temperature (25 °C) by $^{13}C\{^1H\}$ NMR; it was assumed that the temperature-dependence, dpK_a*/dT, would be approximately equal in H_2O and D_2O. Prior to NMR measurements, the spectrometer temperature was calibrated using ethylene glycol (80%) in DMSO-d_6, as per the recommendations of Bruker, using a VT gas flow rate of 400 L hr^{-1}. All final pH* measurements were made in batch, after calibration of the pH meter at 20 °C using phalate (pH(20°C) = 3.980; *Fisher Chemicals*), phosphate (pH(20°C) = 7.010; *Fisher Chemicals*), and borate (pH(20°C) = 9.190; Acros Organics) buffers.

For the titrations in D_2O, a parent stock solution of L-serinamide deuterochloride ([^{L}**S**]$_T$ = 100 mM), tetra-*n*-butylammonium chloride (TBAC; 20 mM) and potassium chloride (1880 mM) was prepared from standard D_2O stocks and aliquoted (700 µL) into 16 separate Eppendorf tubes. Variable amounts of concentrated KOD (4.0 M), nominally spanning 0 – 1.1 equivalents (i.e., 0 – 77 µM) with respect to [^{L}**S**]$_T$, were dispensed in regular increments to each tube, and rough pH* values were measured under ambient conditions to ensure appropriate sampling of the pH* range, including limiting values. Small adjustments were made with further KOD (4.0 M) or DCl (4.0 M) where necessary to achieve good sampling.

Each sample was next transferred to a separate 5mm borosilicate NMR tube (Norell Select Series, 400 MHz), and then analysed by ^1H (zg30, t_{aq} = 4.0 s, t_{d1} = 10 s) and $^{13}C\{^1H\}$ (zgpg30, t_{aq} = 2.0 s) NMR spectroscopy. All samples were first analysed at 25.0 °C, with each sample equilibrated in the NMR probehead for at least 10 minutes prior to FID acquisition (^1H followed by $^{13}C\{^1H\}$). The NMR probehead was then cooled to 10.0 °C, and all samples were analyzed in batch, this time using a total pre-acquisition equilibration period of 15 min. Finally, the NMR probehead was warmed, slowly, to 50.0 °C, after which all samples were analysed for a final time, again using a pre-acquisition equilibration period of 15 min. All ^1H and $^{13}C\{^1H\}$ spectra were processed in a standard manner, with referencing to the residual HDO signal (δ_H = 4.790 ppm) or the most upfield signal of TBAC (δ_C = 13.25 ppm).

After all NMR measurements had been completed, all 16 samples were transferred back to separate, clean Eppendorf tubes for final pH* analysis. No changes to the composition of any solution were made at this point. All samples were first equilibrated at 25 °C in an *Eppendorf* Thermomixer C for 10 min (600 rpm), and final pH*(25 °C) values were then measured directly. All solutions were then cooled to 10°C *in situ*, and the pH*(10°C) of each sample was measured, without any automatic temperature corrections, after an equilibration period of 15 min. Several readings were taken for each solution, and a final reading for pH*(10°C) was only taken once the value of pH* had stabilized. All solutions were then warmed to 50°C *in situ*, and the pH*(50°C) of each sample was measured in the same way as at 10°C.

The pK_{aH}* of L-serinamide at a given temperature, pK_a*(^{L}**S**$^{+}$; T), was determined by non-linear least-squares fitting of observed ($\delta_{obs}^{(i)}$) chemical shifts to calculated values ($\delta_{calc}^{(i)}$) (**Figure S4**). In the case of ^{13}C NMR data, a single value for pK_a*(^{L}**S**$^{+}$; T) was determined by *globally* fitting the chemical shifts of all three ^{13}C resonances; for ^{1}H NMR, only the chemical shifts of the α-proton were used. At all temperatures, identical values of pK_a*(^{L}**S**$^{+}$; T) (within 0.01 pK units) were obtained by ^{1}H and ^{13}C analysis.

The theoretical chemical shift for each resonance i, $\delta_{calc}^{(i)}$, was calculated according to:

$$\delta_{calc}^{(i)} = X_{\{^{L}S^{0}\}}\,\delta_{\{^{L}S^{0}\}}^{(i)} + \left(1 - X_{\{^{L}S^{0}\}}\right)\delta_{\{^{L}S^{+}\}}^{(i)} \qquad (S103)$$

where $X_{\{LS0\}}$ denotes the mole fraction of unprotonated L-serinamide (overall charge = 0), $\delta_{\{LS0\}}^{(i)}$ is the chemical shift of resonance *i* in this state, and $\delta_{\{LS+\}}^{(i)}$ is the chemical shift of resonance *i* in protonated L-serinamide (overall charge = 1). Making no distinction between the un-ionised and zwitterionic tautomers of neutral L-serinamide, $X_{\{LS0\}}$ was calculated according to:

$$X_{\{^{L}S^{0}\}} = \frac{1}{1 + 10^{\Delta pK_a^{*}(^{L}S^{+})}} \qquad (S104)$$

The limiting shifts $\delta_{\{LS0\}}^{(i)}$ and $\delta_{\{LS+\}}^{(i)}$ were constrained to the values of $\delta_{obs}^{(i)}$ measured at limitingly high (i.e., pH* > 10) and low pH* (i.e., pH* < 5); only pK_a*(^{L}**S**$^{+}$) was floated as a variable parameter.

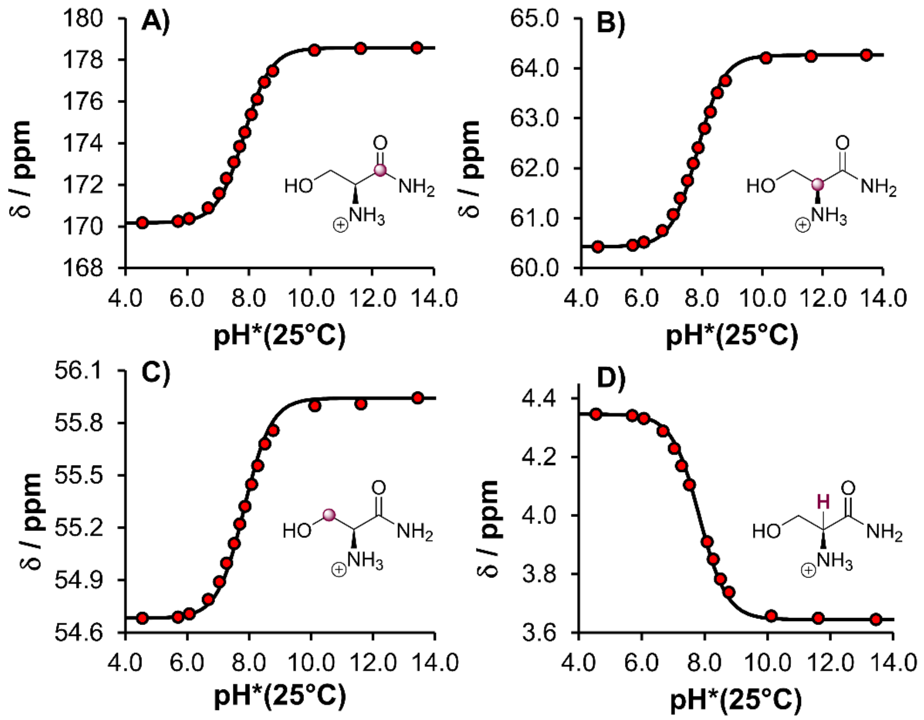

Figure S4: Typical ^{13}C{^{1}H} (A-C) and ^{1}H (D) NMR titration data for L-serinamide in (D_2O, 25 °C, I = 2.0 M, KCl), with fitted isotherms. pK_a*(^{L}**S**$^{+}$; 25°C) = 7.84. Temperature-dependence data shown in the main text; dpK_a*(^{L}**S**$^{+}$)/dT = -0.025 °C^{-1} (10 – 50 °C). ^{1}H NMR data around pH*(25°C) = 7.7 – 7.9 compromised by highly second-order spectra.

Strictly, the observed/phenomenological acidity constant K_a*(^{L}**S**$^+$) is equal to the sum of the microscopic acidity constants N-H and O-H protons in ^{L}**S**$^+$: loss of a N-H proton gives the un-ionised species (^{L}**S**$_0^0$), with a microscopic acidity constant K_a*(^{L}**S**$_{NH3}^+$); loss of the O-H proton gives the zwitterion of L-serinamide (^{L}**S**$_{+-}^0$), with a microscopic acidity constant K_a*(^{L}**S**$_{OH}^+$):

$$K_a^*(^L\mathbf{S}^+) = \frac{[^L\mathbf{S^0}][\mathbf{H}^+]}{[^L\mathbf{S}^+]} = \frac{\left([^L\mathbf{S}_0^0] + [^L\mathbf{S}_\pm^0]\right)[\mathbf{H}^+]}{[^L\mathbf{S}^+]} = K_a^*(^L\mathbf{S}_{\mathbf{NH_3}}^+) + K_a^*(^L\mathbf{S}_{\mathbf{OH}}^+) \tag{S105}$$

The relative proportion of neutral L-serinamide in its zwitterionic state (i.e., [^{L}**S**$_{+-}^0$]/[^{L}**S**^{0}]) cannot be deduced from the NMR titration data because tautomerization of the un-ionised species to the zwitterion does not involve a net loss or gain of protons. Though the fractional population of the zwitterion is expected to be very small, in principle the alkoxide fragment would be expected to be potently nucleophilic, so the possibility arises that the reaction of neutral L-serinamide with aminoacyl esters proceeds via direct reaction of a small reservoir of highly reactive zwitterion. This possibility cannot be distinguished kinetically from the reaction of un-ionised serinamide with intramolecular general base catalysis.

The kinetic plausibility of the direct zwitterion reaction can, however, be probed *indirectly* by *assuming* that this pathway predominates in the reactions of neutral L-serinamide, and then calculating the *hypothetical* second-order rate constants for its reaction with, e.g., **E**$_m^+$ ($k_{Am,+\{+-\}}$) (and/or **E**$_m^0$ ($k_{Am,0\{+-\}}$)). I.e., if it is assumed that

$$k_{Am,+\{\pm\}}[^L\mathbf{S}_\pm^0] = k_{Am,+}[^L\mathbf{S^0}] \tag{S106}$$

$$k_{Am,0\{\pm\}}[^L\mathbf{S}_\pm^0] = k_{Am,0}[^L\mathbf{S^0}] \tag{S107}$$

Then the hypothetical second-order rate constants $k_{Am,+\{+-\}}$ and $k_{Am,0\{+-\}}$ can in principle be calculated from: (i) $k_{Am,+}$ and $k_{Am,0}$, deduced from experiment and pH*-k^{Ψ}_{Am} profile deconvolution, vide supra; and (ii) the microscopic acidity constants K_a*(^{L}**S**$_{NH3}^+$) and K_a*(^{L}**S**$_{OH}^+$). Given that K_a*(^{L}**S**$_{NH3}^+$) >> K_a*(^{L}**S**$_{OH}^+$), and therefore that K_a*(^{L}**S**$_{NH3}^+$) ≈ K_a*(^{L}**S**$^+$), it is clear that

$$\frac{[^L\mathbf{S}_\pm^0]}{[^L\mathbf{S^0}]} = \frac{[^L\mathbf{S}_\pm^0]}{[^L\mathbf{S}_0^0] + [^L\mathbf{S}_\pm^0]} \approx \frac{K_a^*(^L\mathbf{S}_{\mathbf{OH}}^+)}{K_a^*(^L\mathbf{S}_{\mathbf{NH_3}}^+)} = 10^{\left[pK_a^*(^L\mathbf{S}_{\mathbf{NH_3}}^+) - pK_a^*(^L\mathbf{S}_{\mathbf{OH}}^+)\right]} = 10^{\left[pK_a^*(^L\mathbf{S}^+) - pK_a^*(^L\mathbf{S}_{\mathbf{OH}}^+)\right]} \tag{S108}$$

$$k_{Am,+\{\pm\}} = k_{Am,+}.\,10^{\left[pK_a^*(^L\mathbf{S}_{\mathbf{OH}}^+) - pK_a^*(^L\mathbf{S}^+)\right]} \tag{S109}$$

$$k_{Am,+\{\pm\}} = k_{Am,+}.\,10^{\left[pK_a^*(^L\mathbf{S}_{\mathbf{OH}}^+) - pK_a^*(^L\mathbf{S}^+)\right]} \tag{S110}$$

pK_a computations for $^{L}S_{OH}^{+}$

The microscopic acidity constant of the O-H proton in $^{L}\mathbf{S}^{+}$, K_a*($^{L}\mathbf{S}_{OH}^{+}$), is not directly measurable, but it may be estimated in reasonably accurate manner by comparison to the experimentally observed pK_a of choline (pK_a*($\mathbf{C}^{+}$) = 13.9)[5] and *ab initio* computation of the free energy of reaction $\Delta G_{PT}°$, corresponding to the isodesmic reaction below.

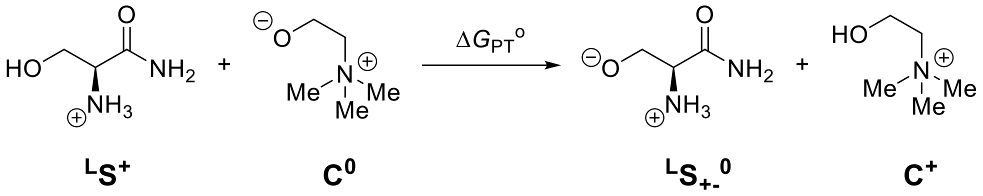

Scheme S2: Isodesmic reaction used to compute the microscopic pK_a of the neutral zwitterion of L-serinamide.

$$K_{PT} = e^{\frac{-\Delta G^{\circ}_{PT}}{RT}} = \frac{K_a^*(^{\mathbf{L}}\mathbf{S}^{+}_{\mathbf{OH}})}{K_a^*(\mathbf{C}^{+})} = 10^{[pK_a^*(\mathbf{C}^{+}) - pK_a^*(^{\mathbf{L}}\mathbf{S}^{+}_{\mathbf{OH}})]} \quad \text{(S111)}$$

$$pK_a^*(\mathbf{C}^{+}) - pK_a^*(^{\mathbf{L}}\mathbf{S}^{+}_{\mathbf{OH}}) = \log_{10}\left(e^{\frac{-\Delta G^{\circ}_{PT}}{RT}}\right) = \frac{-\Delta G^{\circ}_{PT}}{RT\ln 10} \quad \text{(S112)}$$

$$pK_a^*(^{\mathbf{L}}\mathbf{S}^{+}_{\mathbf{OH}}) = pK_a^*(\mathbf{C}^{+}) + \frac{\Delta G^{\circ}_{PT}}{RT\ln 10} \quad \text{(S113)}$$

The free energy of reaction $\Delta G_{PT}°$ was computed using a combination of the xTB (v. 6.5.1),[6] CREST (v. 2.1.2),[7] QCG, [8]and CENSO (v. 1.2.0)[9] programs of the Grimme group, underpinned by electronic structure calculations performed with ORCA (v. 5.0.3)[10] from the Neese group. Initial geometries of $^{L}\mathbf{S}^{+}$, $\mathbf{C}^{0}$, $^{L}\mathbf{S}_{+-}^{0}$ and $\mathbf{C}^{+}$ were constructed in Avogadro (v. 1.2.0) and then optimized crudely at the GFN-FF level of theory, using the analytical linearized Poisson-Boltzmann (ALPB) model of implicit solvation (H_2O), to afford input geometries for all subsequent calculations.

The standard Gibbs free energy of each species *i* (={$^{L}\mathbf{S}^{+}$, $\mathbf{C}^{0}$, $^{L}\mathbf{S}_{+-}^{0}$,**C**}) was taken to be that of the corresponding *conformer ensemble*, $G_{av}°${i}, at T = 298.15 K (25 °C) and c° = 1M. $G_{av}°${i} was approximated as a Boltzmann-weighted average of the free energies of the individual conformers, $G_j°${i}, in the ensemble of *i;* the individual conformers *j*, up to a total of n_{conf}, were generated as described below. No attempt was made to rigorously calculate the conformational entropy of any ensemble, i.e.,

$$G^{\circ}_{av}\{i\} = \sum_{j=1}^{n_{conf}} p_j\{i\}.G^{\circ}_j\{i\} \qquad p_j\{i\} = \frac{e^{-\frac{\left(G^{\circ}_j\{i\} - G^{\circ}_{ref}\{i\}\right)}{RT}}}{\sum_k^{n_{conf}} e^{-\frac{\left(G^{\circ}_k\{i\} - G^{\circ}_{ref}\{i\}\right)}{RT}}} \quad \text{(S114)}$$

where p_j{i} is the Boltzmann population of conformer *j* in the ensemble of species *i*. $\Delta G_{PT}°$ was then calculated according to:

$$\Delta G^{\circ}_{PT} = \left(G^{\circ}_{av}\{\mathbf{C}^{+}\} + G^{\circ}_{av}\{^{\mathbf{L}}\mathbf{S}^{\mathbf{0}}_{\pm}\}\right) - \left(G^{\circ}_{av}\{\mathbf{C}^{\mathbf{0}}\} + G^{\circ}_{av}\{^{\mathbf{L}}\mathbf{S}^{+}\}\right) \quad \text{(S115)}$$

The conformer ensemble of a given species was calculated in two different ways: (i) as an ensemble of *implicitly solvated* conformers; and (ii) as an ensemble of explicitly micro-solvated conformers, with each conformer consisting of the solute surrounded by a cluster of *n* water molecules (*n* = 2 – 20). In the latter case, $\Delta G_{PT}°$ was calculated for each given *n* with *all species* strictly solvated by an *equal number* of water molecules, i.e.,

$$\Delta G^{\circ}_{PT} = \left(G^{\circ}_{av}\{\mathbf{C}^{+}_{\mathbf{n-H_2O}}\} + G^{\circ}_{av}\{{}^{\mathbf{L}}\mathbf{S}^{\mathbf{0}}_{\pm,\mathbf{n-H_2O}}\}\right) - \left(G^{\circ}_{av}\{\mathbf{C}^{\mathbf{0}}_{\mathbf{n-H_2O}}\} + G^{\circ}_{av}\{{}^{\mathbf{L}}\mathbf{S}^{+}_{\mathbf{n-H_2O}}\}\right) \quad \text{(S116)}$$

Implicitly solvated conformer ensembles were generated directly using the iMTD-GC algorithm of CREST, at the GFN2-xTB level of theory, in conjunction with: (i) the analytical linearized Poisson-Boltzmann (ALPB) model of implicit solvation (ALPB[H_2O]); (ii) an energy window of 7.0 kcal mol^{-1}; (iii) automatically calculated simulation lengths and timesteps for the metadynamics simulations; (iv) very tight optimization thresholds for the final stage ANCOPT optimizations; and (v) otherwise default options for conformational searching (with genetic z-matrix crossing) and ensemble sorting (RMSD threshold = 0.125 Ang; conformer energy threshold = 0.05 kcal mol^{-1}; minimum rotational constant threshold = 1.0%). *Explicitly solvated* conformer ensembles were generated with the Quantum Cluster Growth (QCG) algorithm implemented in CREST , using: (i) GFN-FF for both cluster growth and ensemble generation; (ii) xTB-IFF to for initial docking, in conjunction with an initial wall potential scaling factor of 0.7; (iii) the NCI-MTD algorithm for ensemble generation, with the default MTD simulation time extended from 10 ps to 500 ps and the timestep kept at 1.5 fs; (iv) an energy window of 8.0 kcal mol^{-1}; and (v) final cluster optimization at the GFN2-xTB/ALPB[H_2O] level of theory. For QCG runs with the zwitterion of L-serinamide (${}^{\mathbf{L}}\mathbf{S}_{+-}^{\mathbf{0}}$), significant harmonic constraints (2.0 Ha Bohr^{-2}) were placed on all N-H bonds and on the C_{β}-H bonds to prevent inadvertent tautomerization/covalent hydration (this was observed repeatedly in the absence of such constraints).

For the implicitly solvated ensembles, all conformers with potential energies lying within an energy window of 7.0 kcal^{-1} from the most stable conformer were taken forward for further refinement; for explicitly solvated ensembles, the *ten* lowest energy clusters/conformers were used.

The refined conformer ensembles from CREST/QCG, both implicitly and explicitly solvated, were next subjected to a higher-level filtering process, as implemented in CENSO and underpinned by ORCA. At each stage of filtering, any conformers with energies outside a given window from the most stable conformer in the ensemble were eliminated; the exact nature of the "energy" calculated for each conformer was dependent on the stage of filtering. For each ensemble, a total of five stages of filtering were conducted, consisting of the following: (i) stage 1 screening, according to single-point potential energy calculations at the TPSS-d3/def2-SV(P)/ALPB[H_2O] level, using the initial GFN2-xTB-optimised geometries supplied by CREST/QCG (window = 7.0 kcal mol^{-1}); (ii) stage 2 screening, according to single-point potential energy calculations on the same geometries at the r2SCAN-3c/def2-mTZVPP/SMD[H_2O] level (window = 6.0 kcal mol^{-1}); (iii) stage 3 screening, according to single-point *free energies*, again on the same geometries, calculated from the sum of the potential energies in step (ii) and single-point (SPH) thermostatistical

corrections G_{mRRHO} calculated at the GFN2-xTB/ALPB[H_2O] level (window = 6.0 kcal mol^{-1} + σ{G_{mRRHO}}); (iv) stage 4 screening, according to free energies calculated at the r2SCAN-3c/def2-mTZVPP/SMD[H_2O]+G_{mRRHO}{GFN2-xTB/ALPB[H_2O]} level using *coarsely optimized* geometries at this level (window = 5.0 kcal mol^{-1}); and (v) final stage filtering, according to free energies calculated at the same level as step (iv) but using *fully optimized* geometries at the r2SCAN-3c/def2-mTZVPP/SMD[H_2O] level. The final conformational ensemble for each species was assembled from the lowest-energy conformers emerging from stage (v), up to a cumulative Boltzmann population of 95%. The final individual conformer energies $G_j°${i} were taken directly from the output of stage (v).

***n* H_2O**	**$\Delta G°_{PT}$ / (kJ mol^{-1})**	**pK_a(^{L}S$_{OH}^+$)**
0 (Implicit)	-14.3	11.4
2	-9.5	12.2
3	-17.7	10.8
4	6.8	15.1
5	-13.1	11.6
6	-17.3	10.9
7	-9.0	12.3
8	0.9	14.1
10	8.8	15.5
12	0.2	13.9
14	-5.4	13.0
16	-12.0	11.8
18	3.3	14.5
20	8.4	15.4
	Average	**13.03**
	SEM	**0.45**
	Error bound (90%)	**0.74**

Table S2: Computed standard free energies of reaction (298 K, 1 M) for the isodesmic proton transfer reaction in Scheme S2, for a given number n of explicit water solvent molecules. See main text for details.

The computed free energies of reaction $\Delta G_{PT}°$ for implicit (n = 0) and explicit (n = 2 – 20) solvation are given in **Table S2,** alongside the corresponding values for pK_a*(^{L}**S**$_{OH}^+$) calculated under the assumption that pK_a*(**C**$^+$) = 13.90. It is clear that there remains a material degree of uncertainty in pK_a*(^{L}**S**$_{OH}^+$), arising from the inherently non-deterministic behaviour of the QCG algorithm; nevertheless, a reasonable estimate of pK_a*(^{L}**S**$_{OH}^+$) may be taken as the mean value pK_a*(^{L}**S**$_{OH}^+$) = 13.03 ± 0.74 (90% confidence).

In conjunction with the experimentally observed value of pK_a*($^L\mathbf{S}^+$) = 7.59 (H_2O, 25 °C, I = 2.0 M, KCl), this suggests that pK_a*($^L\mathbf{S_{OH}}^+$) – pK_a*($^L\mathbf{S}^+$) = 5.44 and in turn that:

$$k_{Am,+\{\pm\}} \approx k_{Am,+} \times 10^{5.44} = k_{Am,+} \times (2.7 \times 10^5) \quad \text{(S117)}$$

$$k_{Am,0\{\pm\}} \approx k_{Am,0} \times 10^{5.44} = k_{Am,0} \times (2.7 \times 10^5) \quad \text{(S118)}$$

The value of the hypothetical rate constant $k_{Am,0\{+-\}}$ is, notably, roughly an order of magnitude greater than the experimental second-order rate constant for the saponification of $\mathbf{E_m^0}$ ($k_{Am,0}$) in H_2O (20°C). The hypothetical constant $k_{Am,+\{+-\}}$ would, by a similar calculation, be estimated to be roughly the same value as $k_{OH-,+}$. Thus, in order for the direct reaction of the zwitterion of L-serinamide to predominate as the major pathway for aminolysis, the zwitterion would have to be at least as *nucleophilic* as hydroxide – despite being an order of magnitude *less basic* and considerably *more sterically hindered*. It would appear reasonable to conclude, in turn, that intramolecular general base catalysis is operative in this system, though we cannot exclude the direct zwitterion reaction definitively or in all contexts.

In situ pK_a determination by δ_F analysis

The observed/phenomenological pK_a* values of $\mathbf{E_m}^+$, $\mathbf{E_{Bis}}^+$ and $\mathbf{E_{Bis}}^{++}$ reported in the main text were obtained *indirectly,* via the deconvolution and fitting of experimental pH*-k^Ψ profiles (*vide supra*), rather than by direct measurement. Conventional NMR titrations, as performed for L-serinamide, were precluded by the hydrolytic lability of these species. Fortunately, however, *direct* corroboration of these values was made possible by analysis of the *in situ*-observed ^{19}F shifts of $\mathbf{E_{m,2'}}$, $\mathbf{E_{m,3'}}$, and $\mathbf{E_{Bis}}$ as a function of pH*. This analysis, which was undertaken using ^{19}F shift and pH* data acquired in D_2O (20°C, I = 2.0 M) over the range pH* = 5.6 – 8.6, is outlined below. All data was extracted from kinetic runs initiated with $\mathbf{E_{Bis}}$, during which $\mathbf{E_{m,2'}}$ and $\mathbf{E_{m,3'}}$, are formed as intermediates.

Given that intramolecular acyl exchange in $\mathbf{E_m}$ was found to be rapid with respect to hydrolysis/aminolysis, but slow relative to the "NMR timescale", distinct, well-resolved ^{19}F resonances were observed for both $\mathbf{E_{m,2'}}$ ($\delta^{F,obs}_{\{Em,2'\}}$) and $\mathbf{E_{m,3'}}$ ($\delta^{F,obs}_{\{Em,3'\}}$) during *in situ* monitoring by ^{19}F{^{1}H} NMR. These shifts, around neutral pH* (i.e., 6 – 9), will be speciation-weighted averages of the individual ^{19}F chemical shifts of $\mathbf{E_{m,2'}}^{\mathbf{0}}$ ($\delta^F_{\{Em0,2'\}}$) and $\mathbf{E_{m,2'}}^+$ ($\delta^F_{\{Em+,2'\}}$), and $\mathbf{E_{m,3'}}^{\mathbf{0}}$ ($\delta^F_{\{Em0,3'\}}$) and $\mathbf{E_{m,3'}}^+$ ($\delta^F_{\{Em+,3'\}}$), respectively, and so may be calculated according to:

$$\delta^{F,calc}_{\{\mathbf{E_{m,2'}}\}} = X_{\{\mathbf{E^0_{m,2'}}\}}\,\delta^{F}_{\{\mathbf{E^0_{m,2'}}\}} + \left(1 - X_{\{\mathbf{E^0_{m,2'}}\}}\right)\delta^{F}_{\{\mathbf{E^+_{m,2'}}\}} \tag{S119}$$

$$\delta^{F,calc}_{\{\mathbf{E_{m,3'}}\}} = X_{\{\mathbf{E^0_{m,3'}}\}}\,\delta^{F}_{\{\mathbf{E^0_{m,3'}}\}} + \left(1 - X_{\{\mathbf{E^0_{m,3'}}\}}\right)\delta^{F}_{\{\mathbf{E^+_{m,3'}}\}} \tag{S120}$$

where $X_{\{\mathbf{Em,2'^0}\}}$ and $X_{\{\mathbf{Em,3'^0}\}}$, denoting the mole fractions of the 2'- and 3'- regioisomers of the aminoacyl monoester $\mathbf{E_m}$ in its unprotonated state, $\mathbf{E_m}^{\mathbf{0}}$, respectively, may be calculated from:

$$X_{\{\mathbf{E^0_{m,i'}}\}} = \frac{[\mathbf{E^0_{m,i'}}]}{[\mathbf{E^0_{m,i'}}] + [\mathbf{E^+_{m,i'}}]} = \frac{1}{1 + \dfrac{[\mathbf{E^+_{m,i'}}]}{[\mathbf{E^0_{m,i'}}]}} = \frac{1}{1 + \dfrac{[\mathrm{H^+}]}{K^*_a(\mathbf{E^+_{m,i'}})}} = \frac{1}{1 + 10^{[\Delta pK^*_a(\mathbf{E^+_{m,i'}})]}} \tag{S121}$$

The microscopic pK_a* values for $\mathbf{E_{m,i'}}^+$ were therefore determined *directly* by fitting the calculated shifts, $\delta^{F,calc}_{\{Em,i'\}}$, to the observed values, $\delta^{F,obs}_{\{Em,i'\}}$, as a function of pH*, with pK_a*($\mathbf{E_{m,i'}}^+$) and both limiting shifts ($\delta^F_{\{Em0,i'\}}$ and $\delta^F_{\{Em+,i'\}}$) floated as variable parameters. For comparison to the kinetically-derived value, the weighted pK_a* of $\mathbf{E_m}^+$ was in turn calculated from the equation below ($K_{iso,0}$ = 1.3, 20°C, D_2O):

$$pK^*_a(\mathbf{E^+_m}) = -\log 10\left\{\frac{(1 + K_{iso,0})K^*_a(\mathbf{E^+_{m,2'}})K^*_a(\mathbf{E^+_{m,3'}})}{K_a(\mathbf{E^+_{m,3'}}) + K_{iso,0}K^*_a(\mathbf{E^+_{m,2'}})}\right\} \tag{S122}$$

An analogous analysis was also undertaken for the amide product, $\mathbf{P_{Am}}$, according to:

$$\delta^{F,calc}_{\{\mathbf{P_{Am}}\}} = X_{\{\mathbf{P^0_{Am}}\}}\,\delta^{F}_{\{\mathbf{P^0_{Am}}\}} + \left(1 - X_{\{\mathbf{P^0_{Am}}\}}\right)\delta^{F}_{\{\mathbf{P^+_{Am}}\}} \tag{S123}$$

$$X_{\{\mathbf{P^0_{Am}}\}} = \frac{1}{1 + 10^{[\Delta pK^*_a(\mathbf{P^+_{Am}})]}} \tag{S124}$$

More nuanced analysis is required in the case of $\mathbf{E_{Bis}}$, for which, again, two distinct, well-resolved resonances were observed by *in situ* $^{19}F\{^1H\}$ NMR: $\delta^{F,2',obs}{}_{\{EBis\}}$, denoting the chemical shift of the ^{19}F nucleus in the 2' aminoacyl fragment; and $\delta^{F,3',obs}{}_{\{EBis\}}$, denoting the corresponding shift for the 3'-aminoacyl fragment. Compared to $\mathbf{E_m}$, the bis-aminoacyl ester poses more complications because there are *four*, rather than *two*, intrinsic ^{19}F chemical shifts that will contribute to *each observed* shift. In the case of the ^{19}F nucleus in the 2'-aminoacyl fragment, for example, the observed shift $\delta^{F,2',obs}{}_{\{EBis\}}$ will strictly be a speciation-weighted average of: (i) $\delta^{F,2'}{}_{\{EBis0\}}$, corresponding to the intrinsic chemical shift of the ^{19}F nucleus of the 2'-aminoacyl fragment (2'-δ_F) in $\mathbf{E_{Bis}^0}$; $\delta^{F,2'}{}_{\{EBis+,2'+\}}$, for the 2'-$\delta_F$ in $\mathbf{E_{Bis,2'+}^+}$; $\delta^{F,2'}{}_{\{EBis+,3'+\}}$, for the 2'-$\delta_F$ in $\mathbf{E_{Bis,3'+}^+}$; and $\delta^{F,2'}{}_{\{EBis++\}}$, for the 2'-$\delta_F$ in $\mathbf{E_{Bis}^{++}}$. An analogous set of four intrinsic ^{19}F chemical shifts would, in principle, contribute to the observed chemical shift of the ^{19}F nucleus in the 3'-aminoacyl fragment $\delta^{F,obs}{}_{\{EBis,3'\}}$.

$$\delta^{F,2',calc}_{\{\mathbf{E_{Bis}}\}} = X_{\{\mathbf{E^0_{Bis}}\}}\,\delta^{F,2'}_{\{\mathbf{E^0_{Bis}}\}} + X_{\{\mathbf{E^+_{Bis,2'+}}\}}\,\delta^{F,2'}_{\{\mathbf{E^+_{Bis,2'+}}\}} + X_{\{\mathbf{E^+_{Bis,3'+}}\}}\,\delta^{F,2'}_{\{\mathbf{E^+_{Bis,3'+}}\}} + X_{\{\mathbf{E^{++}_{Bis}}\}}\,\delta^{F,2'}_{\{\mathbf{E^{++}_{Bis}}\}} \quad (S125)$$

$$\delta^{F,3',calc}_{\{\mathbf{E_{Bis}}\}} = X_{\{\mathbf{E^0_{Bis}}\}}\,\delta^{F,3'}_{\{\mathbf{E^0_{Bis}}\}} + X_{\{\mathbf{E^+_{Bis,2'+}}\}}\,\delta^{F,3'}_{\{\mathbf{E^+_{Bis,2'+}}\}} + X_{\{\mathbf{E^+_{Bis,3'+}}\}}\,\delta^{F,3'}_{\{\mathbf{E^+_{Bis,3'+}}\}} + X_{\{\mathbf{E^{++}_{Bis}}\}}\,\delta^{F,3'}_{\{\mathbf{E^{++}_{Bis}}\}} \quad (S126)$$

As a necessary simplification, it was assumed that the shift of the ^{19}F nucleus in the 2'-aminoacyl fragment is approximately independent of the charge state in the 3'-aminoacyl fragment, and *vice versa*, in all species. Under such an assumption, one has $\delta^{F,2'}{}_{\{EBis0\}} \approx \delta^{F,2'}{}_{\{EBis+,3'+\}}$ and $\delta^{F,2'}{}_{\{EBis+,2'+\}} \approx \delta^{F,2'}{}_{\{EBis++\}}$, and equivalent approximations for the 3'-δ_F, so that:

$$\delta^{F,2',calc}_{\{\mathbf{E_{Bis}}\}} = \left(X_{\{\mathbf{E^0_{Bis}}\}} + X_{\{\mathbf{E^+_{Bis,3'+}}\}}\right)\delta^{F,2'}_{\{\mathbf{E^0_{Bis}}\}} + \left(X_{\{\mathbf{E^+_{Bis,2'+}}\}} + X_{\{\mathbf{E^{++}_{Bis}}\}}\right)\delta^{F,2'}_{\{\mathbf{E^{++}_{Bis}}\}} \quad (S127)$$

$$\delta^{F,3',calc}_{\{\mathbf{E_{Bis}}\}} = \left(X_{\{\mathbf{E^0_{Bis}}\}} + X_{\{\mathbf{E^+_{Bis,2'+}}\}}\right)\delta^{F,3'}_{\{\mathbf{E^0_{Bis}}\}} + \left(X_{\{\mathbf{E^+_{Bis,3'+}}\}} + X_{\{\mathbf{E^{++}_{Bis}}\}}\right)\delta^{F,3'}_{\{\mathbf{E^{++}_{Bis}}\}} \quad (S128)$$

The mole fractions of $\mathbf{E_{Bis}^0}$, $\mathbf{E_{Bis,2'+}^+}$, $\mathbf{E_{Bis,3'+}^+}$ and $\mathbf{E_{Bis}^{++}}$ are:

$$X_{\{\mathbf{E^0_{Bis}}\}} = \frac{[\mathbf{E^0_{Bis}}]}{[\mathbf{E_{Bis}}]_\mathbf{T}} = \frac{1}{1 + 10^{\left[\Delta pK_a^*\left(\mathbf{E^+_{Bis,3'+}}\right)\right]} + 10^{\left[\Delta pK_a^*\left(\mathbf{E^+_{Bis,2'+}}\right)\right]} + 10^{\left[\Delta pK_a^*(\mathbf{E^+_{Bis}})+\Delta pK_a^*(\mathbf{E^{++}_{Bis}})\right]}} \quad (S129)$$

$$X_{\{\mathbf{E^+_{Bis,2'+}}\}} = \frac{[\mathbf{E^+_{Bis,2'+}}]}{[\mathbf{E_{Bis}}]_\mathbf{T}} = \frac{10^{\left[\Delta pK_a^*\left(\mathbf{E^+_{Bis,2'+}}\right)\right]}}{1 + 10^{\left[\Delta pK_a^*\left(\mathbf{E^+_{Bis,3'+}}\right)\right]} + 10^{\left[\Delta pK_a^*\left(\mathbf{E^+_{Bis,2'+}}\right)\right]} + 10^{\left[\Delta pK_a^*(\mathbf{E^+_{Bis}})+\Delta pK_a^*(\mathbf{E^{++}_{Bis}})\right]}} \quad (S130)$$

$$X_{\{\mathbf{E^+_{Bis,3'+}}\}} = \frac{[\mathbf{E^+_{Bis,3'+}}]}{[\mathbf{E_{Bis}}]_\mathbf{T}} = \frac{10^{\left[\Delta pK_a^*\left(\mathbf{E^+_{Bis,3'+}}\right)\right]}}{1 + 10^{\left[\Delta pK_a^*\left(\mathbf{E^+_{Bis,3'+}}\right)\right]} + 10^{\left[\Delta pK_a^*\left(\mathbf{E^+_{Bis,2'+}}\right)\right]} + 10^{\left[\Delta pK_a^*(\mathbf{E^+_{Bis}})+\Delta pK_a^*(\mathbf{E^{++}_{Bis}})\right]}} \quad (S131)$$

$$X_{\{\mathbf{E^{++}_{Bis}}\}} = \frac{[\mathbf{E^{++}_{Bis}}]}{[\mathbf{E_{Bis}}]_\mathbf{T}} = \frac{10^{\left[\Delta pK_a^*(\mathbf{E^+_{Bis}})+\Delta pK_a^*(\mathbf{E^{++}_{Bis}})\right]}}{1 + 10^{\left[\Delta pK_a^*\left(\mathbf{E^+_{Bis,3'+}}\right)\right]} + 10^{\left[\Delta pK_a^*\left(\mathbf{E^+_{Bis,2'+}}\right)\right]} + 10^{\left[\Delta pK_a^*(\mathbf{E^+_{Bis}})+\Delta pK_a^*(\mathbf{E^{++}_{Bis}})\right]}} \quad (S132)$$

The three *microscopic* acidity constants pK_a*($\mathbf{E}_{\mathbf{Bis,2'}}$$^{++}$), p$K_a$*($\mathbf{E}_{\mathbf{Bis,2'+}}$$^{+}$) and p$K_a$*($\mathbf{E}_{\mathbf{Bis,3'+}}$$^{+}$), and the four limiting ^{19}F chemical shifts ($\delta^{F,2'}{}_{\{EBis0\}}$; $\delta^{F,2'}{}_{\{EBis++\}}$; $\delta^{F,3'}{}_{\{EBis0\}}$; $\delta^{F,3'}{}_{\{EBis++\}}$), were then determined by global fitting of $\delta^{F,2',calc}{}_{\{EBis\}}$ and $\delta^{F,3',calc}{}_{\{EBis\}}$ to the corresponding observed ^{19}F shifts for the 2'- and 3'-aminoacyl fragments in $\mathbf{E}_{\mathbf{Bis}}$, respectively, with the constraint that:

$$pK_a^*(\mathbf{E}_{\mathbf{Bis,3'}}^{++}) \approx pK_a^*(\mathbf{E}_{\mathbf{Bis,2'}}^{++}) + [pK_a^*(\mathbf{E}_{\mathbf{Bis,3'+}}^{+}) - pK_a^*(\mathbf{E}_{\mathbf{Bis,2'+}}^{+})] \tag{S133}$$

The *observed/phenomenological* acidity constants pK_a*($\mathbf{E}_{\mathbf{Bis}}$$^{++}$) and p$K_a$*($\mathbf{E}_{\mathbf{Bis}}$$^{+}$) were then calculated according to the equations below (see equations S82 – S92); it is these values that must be compared with those deduced from kinetic data.

$$pK_a^*(\mathbf{E}_{\mathbf{Bis}}^{+}) = pK_a^*(\mathbf{E}_{\mathbf{Bis,2'+}}^{+}) + \log_{10}\left(1 + 10^{pK_a^*(\mathbf{E}_{\mathbf{Bis,3'+}}^{+}) - pK_a^*(\mathbf{E}_{\mathbf{Bis,2'+}}^{+})}\right) \tag{S134}$$

$$pK_a^*(\mathbf{E}_{\mathbf{Bis}}^{++}) = pK_a^*(\mathbf{E}_{\mathbf{Bis,3'}}^{++}) - \log_{10}\left(1 + 10^{pK_a^*(\mathbf{E}_{\mathbf{Bis,3'}}^{++}) - pK_a^*(\mathbf{E}_{\mathbf{Bis,2'}}^{++})}\right) \tag{S135}$$

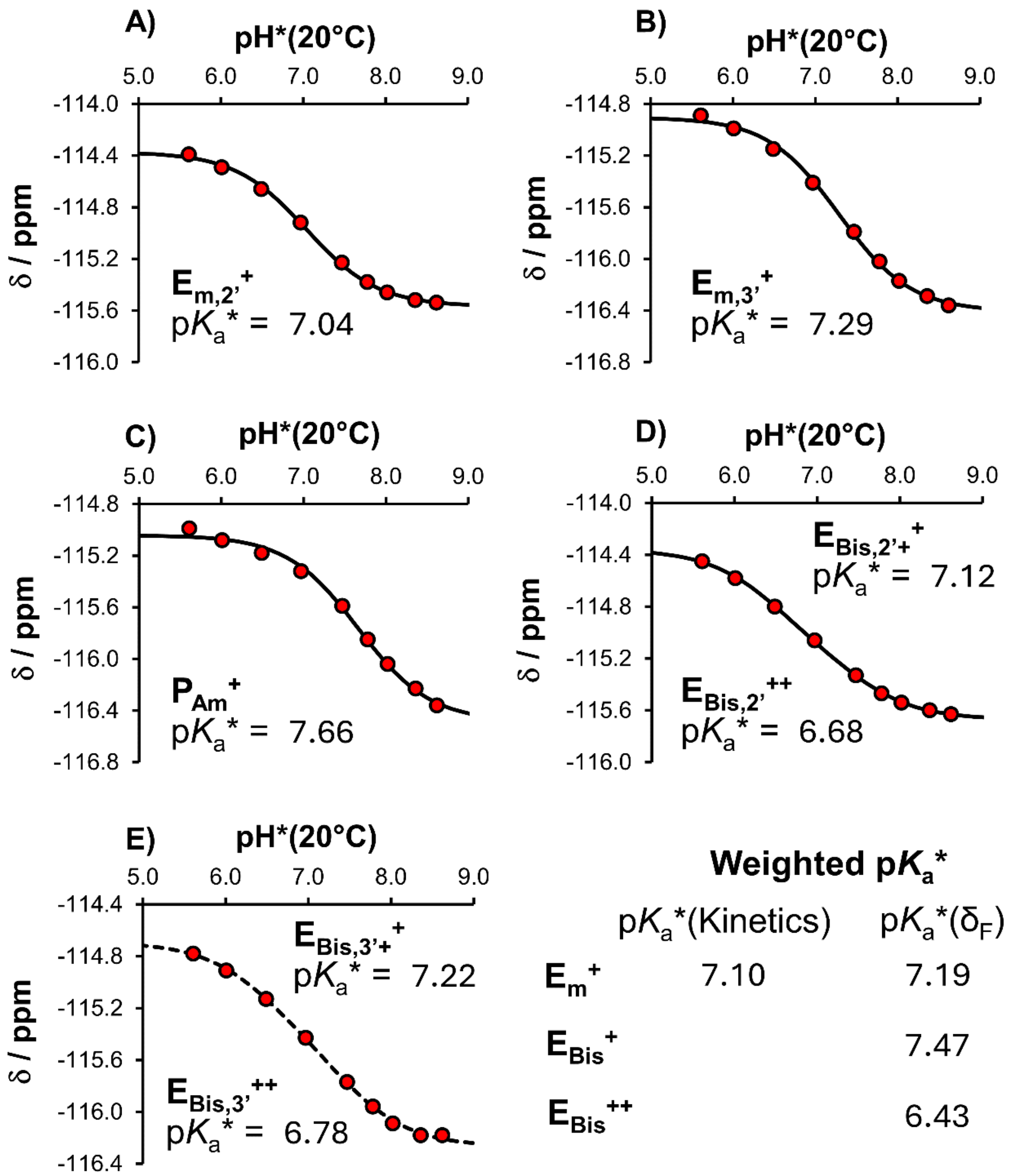

Figure S5: In situ $^{19}F\{^{1}H\}$ NMR titration data for $\mathbf{E_m}$ [(A), (B)], $\mathbf{P_{Am}}$ (C) and $\mathbf{E_{Bis}}$ [(D), (E)] in D_2O (20 °C, I = 2.0 M, KCl) over the range pH*(20°C) = 5.6 – 8.6. All ^{19}F chemical shifts, δ_F, were measured during the course of reactions of $\mathbf{E_{Bis}}$ (4 mM) with L-serinamide ($[^{L}\mathbf{S}]_T$ = 600 – 2000 mM) and referenced against trifluoroacetate (δ_F = 75.15 ppm). Microscopic pK_a* values and limiting shifts were determined by non-linear regression. Limiting shifts (ppm): $\delta^{F}_{\{Em0,3'\}}$ = -116.41, $\delta^{F}_{\{Em+,3'\}}$ = -114.90; $\delta^{F}_{\{Em0,2'\}}$ = -115.57, $\delta^{F}_{\{Em+,2'\}}$ = -114.37; $\delta^{F,2'}_{\{EBis0\}}$ = -115.67, $\delta^{F,2'}_{\{EBis++\}}$ = -114.36; $\delta^{F,3'}_{\{EBis0\}}$ = -116.2, $\delta^{F,3'}_{\{EBis++\}}$ = -114.69; $\delta^{F}_{\{PAm0\}}$ = -116.48, $\delta^{F}_{\{PAm+\}}$ = -115.04. Table shows *observed/phenomenological* pK_a* values derived from the *microscopic* pK_a* values in (A) – (E), and their comparison to the observed/phenomenological values determined by kinetic deconvolution (*vide supra*). See text for further details.

Kinetic data

Supplementary figures

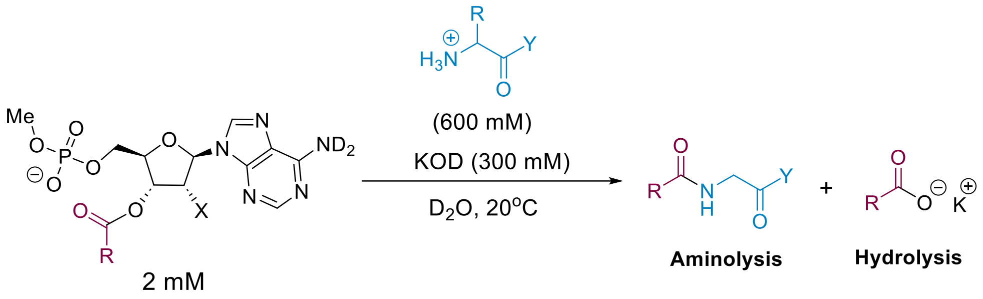

Nucleophile	pH*(20°C)	E_m	E_m^D	E_m^f	E_{Bis}	dE_m^f
Gly-OH	10.3	**< 5**	< 5	< 5	< 5	< 5
Gly-NH_2	8.5	**< 5**	< 5	7	9	10
L-Ser-OH	9.7	**42**	32	42	50	43
D-Ser-OH	9.7	**52**	34	56	55	45
L-Ser-NH_2	7.7	**55**	43	61	67	61
O-P-L-Ser-OH	9.8	**< 5**	< 5	< 5	< 5	< 5
O-Me-L-Ser-OH	8.8	**< 5**	< 5	< 5	< 5	< 5
L-Ser-O^{t}Bu	7.7	**55**	-	-	-	-
L-Thr-NH_2	7.4	**16**	-	-	-	-

Table S3: Yields of amide (fractional aminolysis; %) in the reactions of given pairs of aminoacyl ester (**E_m**, **E_m^D**, **E_m^f**, **E_{bis}**, **dE_m^f**) and nucleophile/buffer in D_2O (20 °C), as determined by quantitative end-point $^{19}F\{^1H\}$ NMR analysis. Ionic strength was not strictly maintained in these experiments. pH*(20°C) corresponds to the apparent, directly-measured pH in D_2O, and reflects the approximate apparent pK_a* of the corresponding nucleophile(/buffer). For O-P-L-Ser-OH, sufficient KOD was added to the phosphoric acid form to achieve a pH* similar to the previous reported pK_{aH}* of the α-amino group.[11]

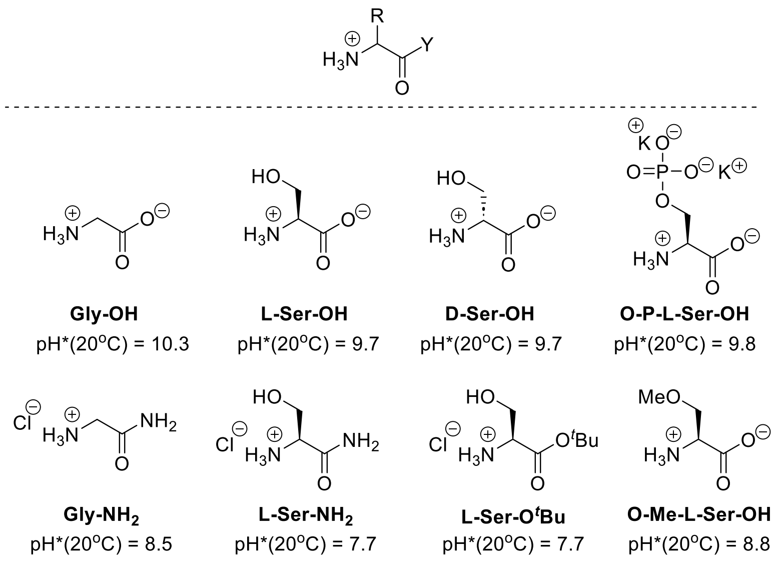

Figure S6: Summary of structures and abbreviated names of the various nucleophiles mentioned in this work. pH*(20°C) value reflects approximate pK_a* (ca. ± 0.5 units).

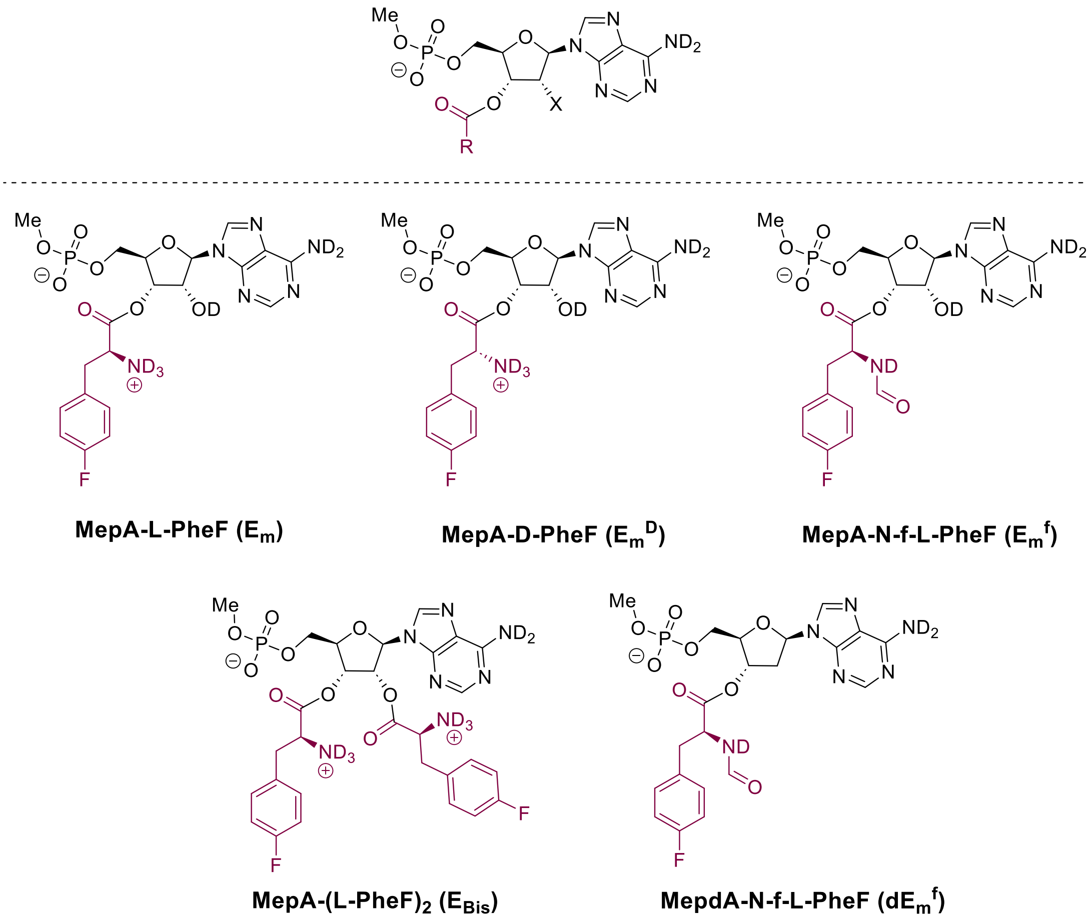

Figure S7: Summary of structures and abbreviated names of the various aminoacyl esters mentioned in this work.

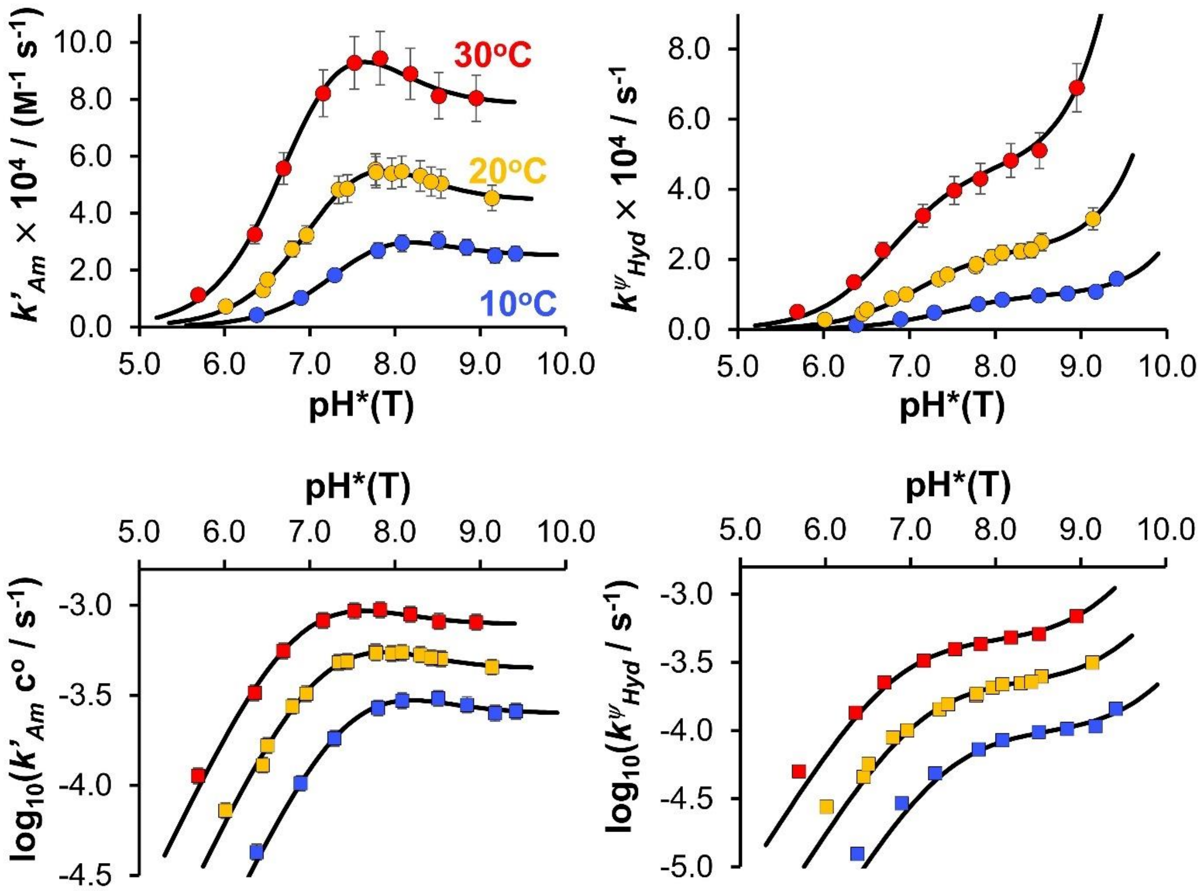

Figure S8: Comparison of pH*-k'_{Am} and pH*-k^{ψ}_{Hyd} profiles (10 - 30°) on logarithmic and non-logarithmic scales, with rate constants measured for the reaction of **MepA-L-PheF** (**E$_m$**) with L-serinamide (**^{L}S**) in D_2O ([**^{L}S**]$_T$ = 600 – 1200 mM; I = 2.0 M, KCl). Simulated profiles (black) calculated using fitted values for second-order rate constants and pK_a*(**E$_m^+$**) values in the main text (*unweighted*). See text for further details. Standard concentration c° = 1 M.

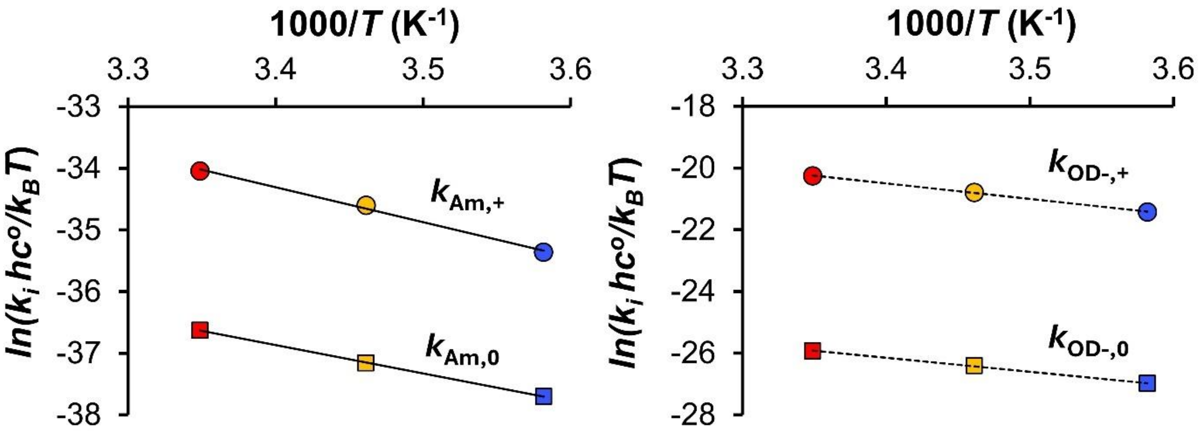

Figure S9: Linearised Eyring plots of the second-order rate constants $k_{Am,+}$, $k_{Am,0}$, $k_{OD-,+}$, and $k_{OD-,0}$ in D_2O (I = 2.0 M). Second-order rate constants obtained by pH*-k'_{Am} and pH*-k^{Ψ}_{Hyd} rate profile deconvolution, as described in the main text (unweighted non-linear fitting). Standard concentration c° = 1 M.

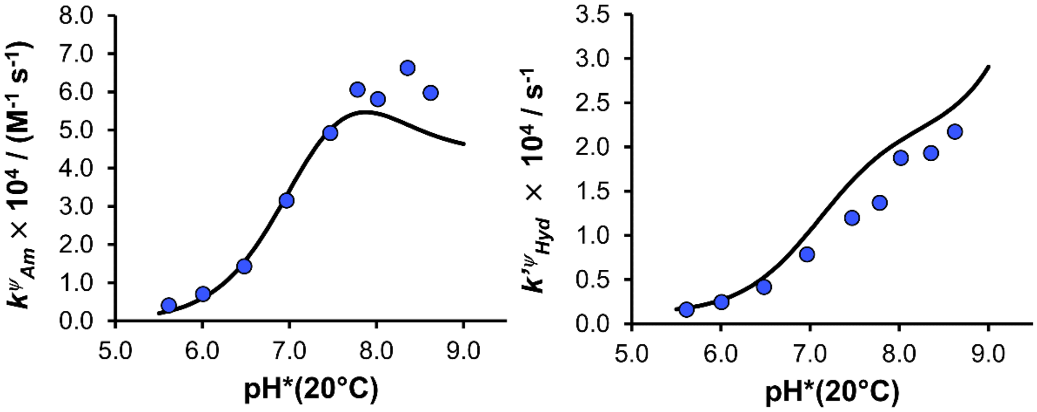

Figure S10: pH*-k'_{Am} and pH*-k^{Ψ}_{Hyd} profiles constructed from pseudo-first order rate constants extracted from kinetic data on the reactions of the *bis*-aminoacyl ester **MepA-(PheF)$_2$** (**E_{Bis}**) with L-serinamide (**^{L}S**) in D_2O (20°C; pH*(20°C) = 5.6 – 8.6; [**^{L}S**]$_T$ = 600 – 2000 mM; I = 2.0 M, KCl). Simulated profiles (black) were calculated using second-order rate constants and pK_a*(**E_m^+**) values *obtained independently, from the deconvolution of* pH*-k'_{Am} and pH*-k^{Ψ}_{Hyd} profiles obtained from data on the reactions of *the mono-aminoacyl ester* ***MepA-L-PheF*** *(**E_m**)* under otherwise identical conditions, i.e., the simulated profiles are calculated from non-fitted parameters.

Fitted parameters: MepA-L-PheF (E_m) + L-serinamide (^{L}S)

Unweighted fitting: data

	**H_2O (20 °C)**	**D_2O (10 °C)**	**D_2O (20 °C)**	**D_2O (30 °C)**
pK_{aH}*(^{L}**S**)	7.72	8.17(5)	7.94(5)	7.69(5)
(pK_{aH}^{D})	-	*(8.58(5))*	*(8.35(5))*	*(8.10(5))*
pK_{aH}*(**E_m**)	6.98(5)	7.40(5)	7.08(2)	6.79(3)
(pK_{aH}^{D})	-	*(7.81(5))*	*(7.49(2))*	*(7.20(3))*
pK_w*	14.16	15.03	14.64	14.29
(pK_w^{D})	-	*(15.44)*	*(15.05)*	*(14.70)*
$k_{Am,+}$ / (M^{-1} s^{-1})	4.6(3) × 10^{-3}	2.6(2) × 10^{-3}	5.8(2) × 10^{-3}	1.0(1) × 10^{-2}
$k_{Am,0}$ / (M^{-1} s^{-1})	4.9(1) × 10^{-4}	2.5(1) × 10^{-4}	4.5(1) × 10^{-4}	7.8(1) × 10^{-4}
$k_{OH^-,+}$ / (M^{-1} s^{-1})	3.2(3) × 10^{3}	2.9(3) × 10^{3}	5.7(2) × 10^{3}	1.0(6) × 10^{4}
$k_{OH^-,0}$ / (M^{-1} s^{-1})	10(3)	11(3)	21(2)	35(3)
$(k_H/k_D)_{Am,+}$	0.80(6)	-	-	-
$(k_H/k_D)_{Am,0}$	1.10(3)	-	-	-
$(k_H/k_D)_{OH^-,+}$	0.56(6)	-	-	-
$(k_H/k_D)_{OH^-,0}$	0.5(1)	-	-	-

Table S4: Full summary of second-order rate-constants, pK_a* and pK_w* data for the reaction of MepA-L-PheF (**E_m**) with L-serinamide (^{L}**S**) in aqueous solution (L_2O; L = H or D; I = 2.0 M, KCl). Values for pK_{aH}(^{L}**S**) obtained by independent $^{1}H/^{13}C\{^{1}H\}$ NMR titrations; values for pK_w* taken from the data of Covington (pK_w^{H}, pK_w^{D}),[1] and values in D_2O calculated from pK_w* = pK_w^{D} – 0.408. All second-order rate constants and pK_{aH}(**E_m**) data obtained vi*a global, unweighted, non-linear fitting of experimental pH*-k'_{Am} and pH*-k^{ψ}_{Hyd} profiles*, in turn obtained by *in situ* $^{19}F\{^{1}H\}$ NMR and analytical kinetic deconvolution, to the appropriate equations (see previous sections). pH* and pK_a* values are apparent/empirical and not true thermodynamic values. pK_{aH}*(^{L}**S**) and pK_w* determined independently (i.e., not by fitting pH*-k profiles). *These data are shown in the main text.*

k_i / (M^{-1} s^{-1})	**Δ‡*H*** (kJ mol^{-1})	**Δ‡*S*** (J K^{-1} mol^{-1})	-(***T*.Δ‡*S***)$_{293K}$ (kJ mol^{-1})	**Δ‡*G*$_{293K}$** (kJ mol^{-1})
$k_{Am,+}$	47 ± 4	-127 ± 12	37 ± 4	84 ± 5
$k_{Am,0}$	38 ± 1	-179 ± 2	52 ± 1	91 ± 1
$k_{OD^-,+}$	42 ± 1	-31 ± 3	9 ± 1	51 ± 1
$k_{OD^-,0}$	38 ± 1	-91 ± 4	27 ± 1	64 ± 2

Table S5: Summary of activation parameters obtained by standard Eyring analysis, i.e., linear regression of $\ln(k_i hc°/k_B T)$ vs 1000/T, with c° = 1 M, for the second-order rate constants in **Table S4**. Standard errors in $\Delta^‡H$ and $\Delta^‡S$ were determined by linear regression, and these were then propagated to estimate the uncertainties in $\Delta^‡G_{293K}$.

Unweighted fitting: plots

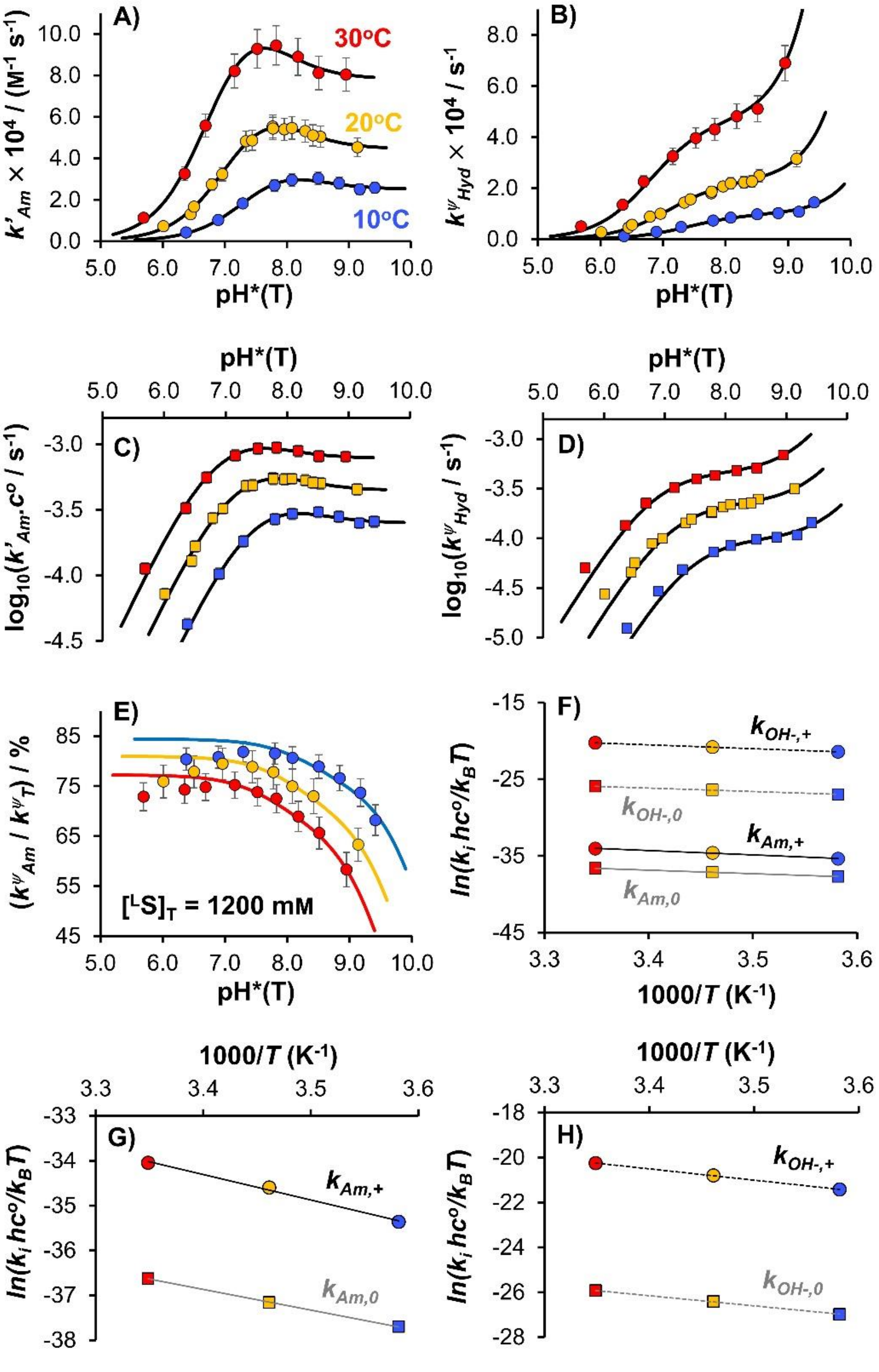

Figure S11: Key plots of experimental data and overlaid model fits, using parameters in **Table S4**, for the reaction of MepA-L-PheF (**E$_m$**) with L-serinamide (^{L}**S**) in aqueous solution (L_2O; L = H or D; I = 2.0 M, KCl; 10 – 30 °C). (A) pH*-k'_{Am} profiles; (B) pH*-k^{ψ}_{Hyd} profiles; (C) Logarithmic form of (A). (D) Logarithmic form of (C). (E) Empirical aminolysis selectivities. (F) Summary of linearised Eyring plots. (G) Linearised Eyring plots for aminolysis. (H) Linearised Eyring plots for saponification. Uncertainties in k'_{Am} and k^{ψ}_{Hyd} conservatively estimated to be $\pm$ 10% on the basis of maximum expected integral errors of < 5 % (see previous sections); uncertainty in selectivities ($k^{\psi}_{Am}/(k^{\psi}_{Am} + k^{\psi}_{Hyd})$) calculated by propagation ($k^{\psi}_{Am} = k'_{Am}[^L\mathbf{S}]_T$).

Variance-weighted fitting: data

	H_2O (20 °C)	D_2O (10 °C)	D_2O (20 °C)	D_2O (30 °C)
pK_{aH}*(**E**$_m$) *(pK_{aH}^D)*	6.84(5) -	7.24(3) *(7.65(3))*	6.98(3) *(7.39(3))*	6.65(3) *(7.06(3))*
$k_{Am,+}$ / (M^{-1} s^{-1})	5.1(3) × 10^{-3}	3.0(1) × 10^{-3}	6.3(2) × 10^{-3}	1.2(1) × 10^{-2}
$k_{Am,0}$ / (M^{-1} s^{-1})	5.2(3) × 10^{-4}	2.6(1) × 10^{-4}	4.8(3) × 10^{-4}	8.2(4) × 10^{-4}
$k_{OH^-,+}$ / (M^{-1} s^{-1})	4.2(3) × 10^{3}	4.1(2) × 10^{3}	6.9(3) × 10^{3}	1.3(1) × 10^{4}
$k_{OH^-,0}$ / (M^{-1} s^{-1})	12(5)	12(3)	23(6)	38(8)
$(k_H/k_D)_{Am,+}$	0.82(6)	-	-	-
$(k_H/k_D)_{Am,0}$	1.08(9)	-	-	-
$(k_H/k_D)_{OH^-,+}$	0.61(6)	-	-	-
$(k_H/k_D)_{OH^-,0}$	0.5(2)	-	-	-

Table S6: Full summary of second-order rate-constants and pK_a* data for the reaction of MepA-L-PheF (**E**$_m$) with L-serinamide (^{L}**S**) in aqueous solution (L_2O; L = H or D; I = 2.0 M, KCl). All second-order rate constants and pK_{aH}*(**E**$_m$) parameters obtained vi*a global, variance-weighted, non-linear fitting of experimental pH*-k'_{Am} and pH*-k^{ψ}_{Hyd} profiles*, in turn obtained by *in situ* ^{19}F{^{1}H} NMR and analytical kinetic deconvolution, to the appropriate equations (see previous sections). pH* and pK_a* values are apparent/empirical and not true thermodynamic values. pK_{aH}*(^{L}**S**) and pK_w* determined independently (i.e., not by fitting pH*-k profiles); see **Table S4**.

k_i / (M^{-1} s^{-1})	$\Delta^{‡}H$ (kJ mol^{-1})	$\Delta^{‡}S$ (J K^{-1} mol^{-1})	-(T.$\Delta^{‡}S$)$_{293K}$ (kJ mol^{-1})	$\Delta^{‡}G_{293K}$ (kJ mol^{-1})
$k_{Am,+}$	47 ± 1	-128 ± 2	38 ± 1	84 ± 1
$k_{Am,0}$	38 ± 1	-178 ± 1	52 ± 1	90 ± 1
$k_{OD^-,+}$	40 ± 3	-35 ± 11	10 ± 3	50 ± 5
$k_{OD^-,0}$	40 ± 3	-84 ± 9	25 ± 3	64 ± 4

Table S7: Summary of activation parameters obtained by standard Eyring analysis, i.e., linear regression of ln($k_i$hc°/k$_B$$T$) vs 1000/T, with c° = 1 M, for the second-order rate constants in **Table S6**. Standard errors in $\Delta^{‡}H$ and $\Delta^{‡}S$ were determined by linear regression, and these were then propagated to estimate the uncertainties in $\Delta^{‡}G_{293K}$.

Variance-weighted fitting: plots

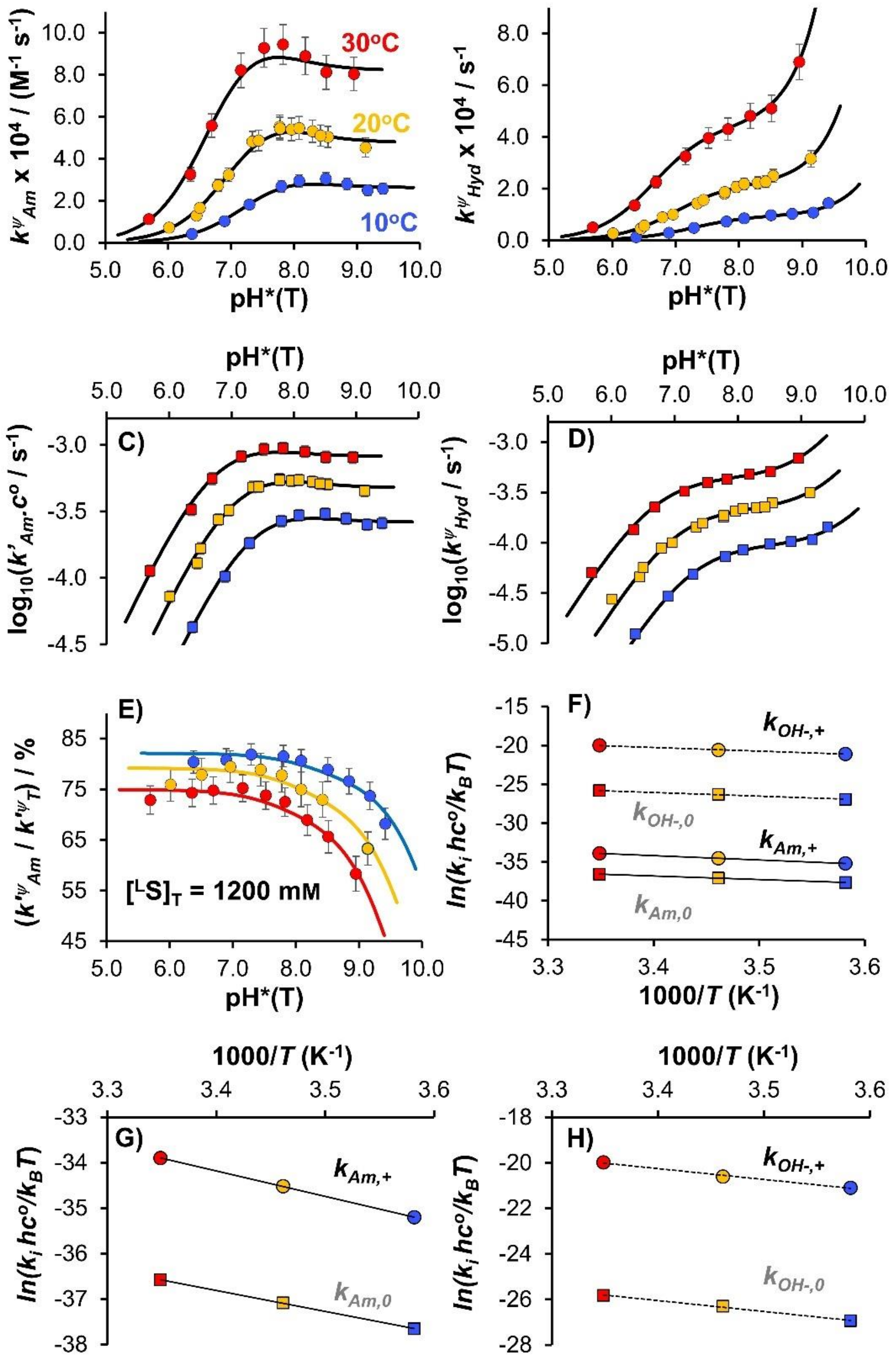

Figure S12: Key plots of experimental data and overlaid model fits, using parameters in **Table S6**, for the reaction of MepA-L-PheF (**E$_m$**) with L-serinamide (^{L}**S**) in aqueous solution (L_2O; L = H or D; I = 2.0 M, KCl; 10 – 30 °C). (A) pH*-k'_{Am} profiles; (B) pH*-k^{ψ}_{Hyd} profiles; (C) Logarithmic form of (A). (D) Logarithmic form of (C). (E) Empirical aminolysis selectivities. (F) Summary of linearised Eyring plots. (G) Linearised Eyring plots for aminolysis. (H) Linearised Eyring plots for saponification. Uncertainties in k'_{Am} and k^{ψ}_{Hyd} conservatively estimated to be $\pm$ 10% on the basis of maximum expected integral errors of < 5 % (see previous sections); uncertainty in selectivities ($k^{\psi}_{Am}/(k^{\psi}_{Am} + k^{\psi}_{Hyd})$) calculated by propagation ($k^{\psi}_{Am} = k'_{Am}$[^{L}**S**]$_T$).

MepA-L-PheF ($\mathbf{E_m}$) + L-serinamide ($^{\mathrm{L}}\mathbf{S}$); D_2O, 20 °C

Summary of pseudo first-order rate constants

pH*(20°C)	$[^{\mathrm{L}}S]_T$ / mM	$k^{\Psi}_{Am} \times 10^4$ / s^{-1}	$k'_{Am} \times 10^4$ / (M^{-1} s^{-1})	$k^{\Psi}_{Hyd} \times 10^4$ / s^{-1}
9.137	1200	5.44	4.53	3.16
8.540	600	3.03	5.04	2.49
8.295	600	3.19	5.31	2.23
7.960	600	3.24	5.40	2.07
7.770	600	3.32	5.53	1.80
7.339	600	2.89	4.82	1.43
6.792	600	1.64	2.74	0.89
6.449	600	0.78	1.30	0.46
8.421	1200	6.13	5.10	2.27
8.081	1200	6.56	5.46	2.19
7.777	1200	6.53	5.44	1.86
7.440	1200	5.84	4.87	1.56
6.960	1200	3.88	3.23	1.00
6.504	1200	2.00	1.66	0.57
6.012	1200	0.87	0.72	0.28

Table S8: Summary of raw data from the pH*-k'_{Am} and pH*-k^{Ψ}_{Hyd} profiles for the aminolysis/hydrolysis of MepA-L-PheF ($\mathbf{E_m}$) with L-serinamide (D_2O, 20 °C, I = 2.0 M, KCl).

Raw reaction profiles

pH*(20°C) = 9.137 ([^{L}**S**]$_T$ = 1200 mM)

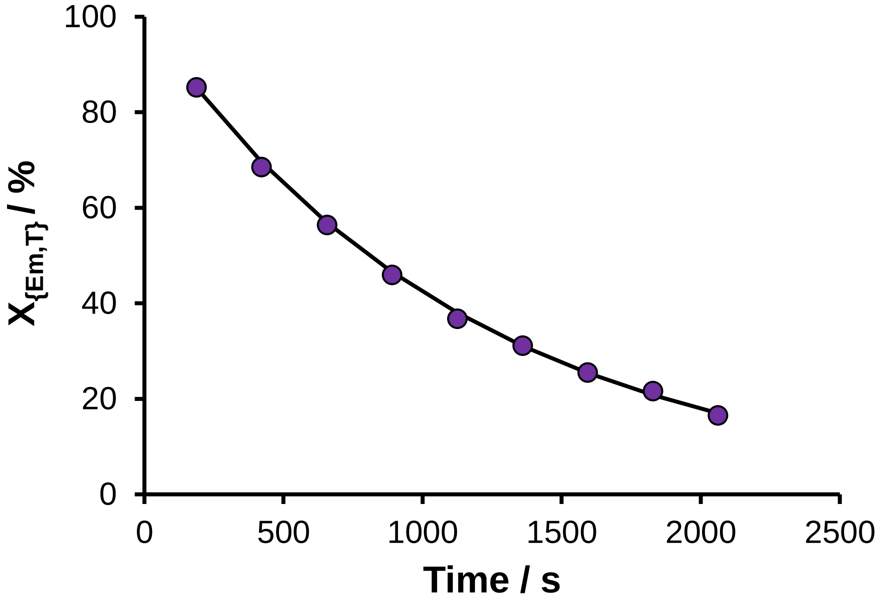

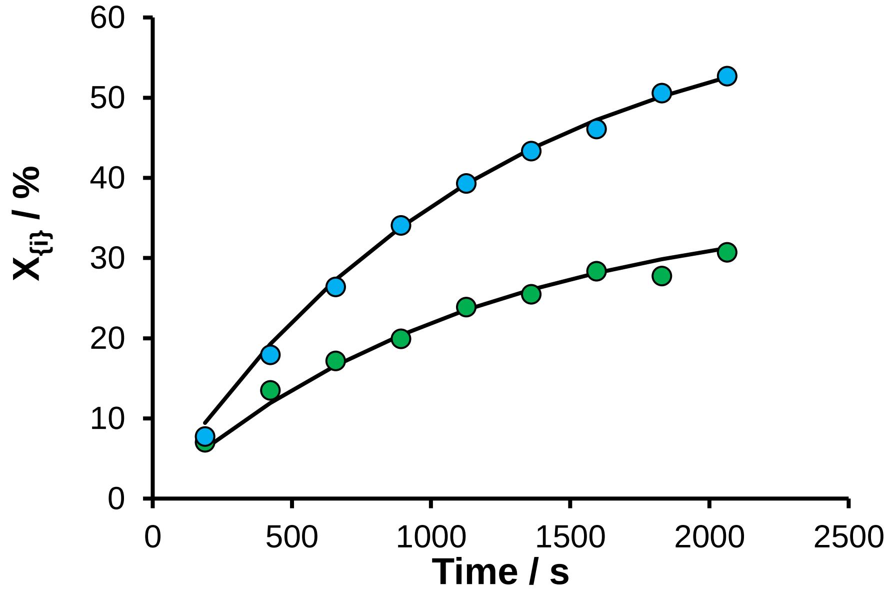

Figure S13: Reaction profiles, expressed in terms of mole fractions (X{i}), for the aminolysis and concurrent hydrolysis of **MepA-L-PheF** (**E**$_m$) in D_2O under the title conditions (20 °C; I = 2.0 M, KCl), as measured by in situ ^{19}F{^{1}H} NMR spectroscopy. The amide **P**$_{Am}$ is the major product; the hydrolysis product, **P**$_{aa}$, is the minor product. Fit to kinetic model 1 shown. ^{L}**S** = L-serinamide.

pH*(20°C) = 8.538 ([^{L}**S**]$_T$ = 600 mM)

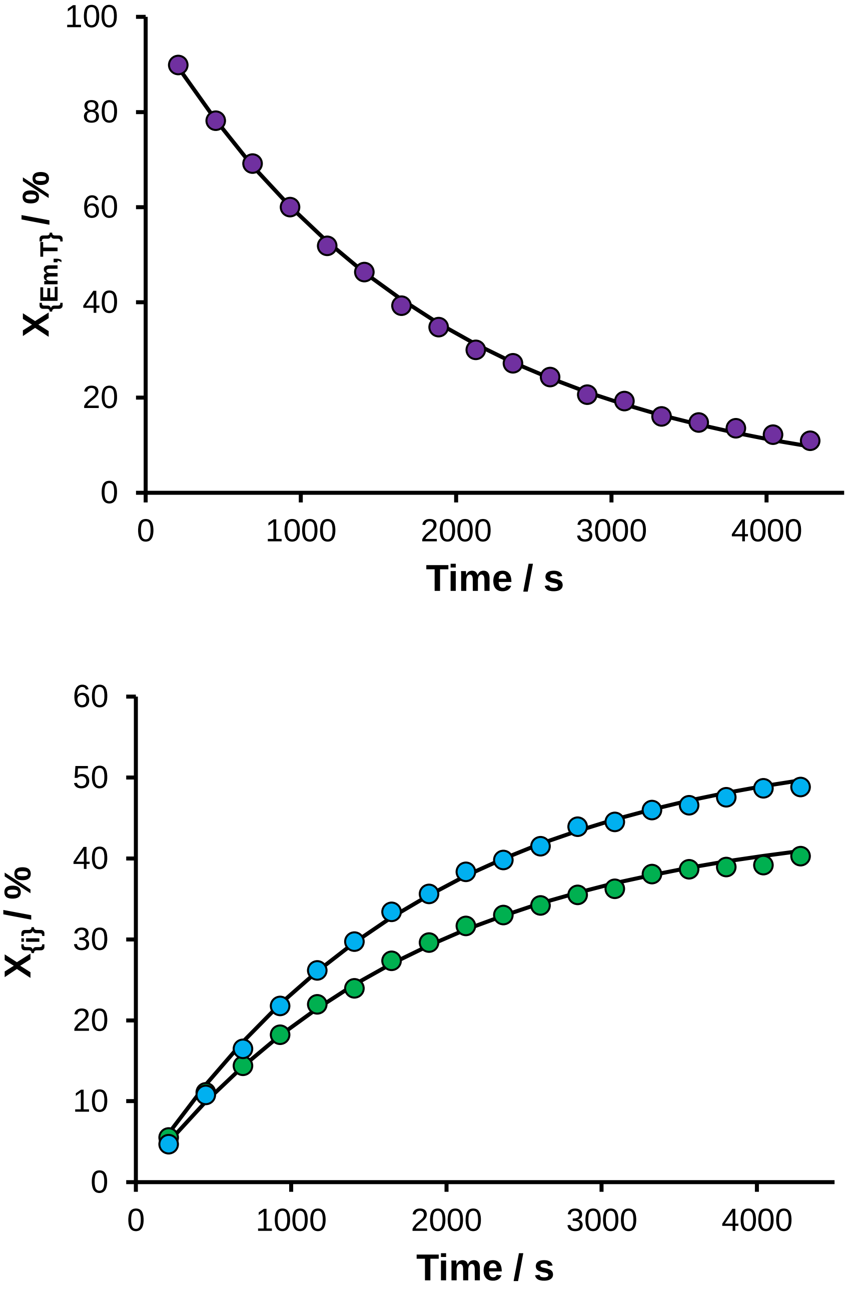

Figure S14: Reaction profiles, expressed in terms of mole fractions (X{i}), for the aminolysis and concurrent hydrolysis of **MepA-L-PheF** (**E**$_m$) in D_2O under the title conditions (20 °C; *I* = 2.0 M, KCl), as measured by in situ $^{19}F\{^{1}H\}$ NMR spectroscopy. The amide **P**$_{Am}$ is the major product; the hydrolysis product, **P**$_{aa,}$ is the minor product. Fit to kinetic model 1 shown. ^{L}**S** = L-serinamide.

pH*(20°C) = 8.295 ([^{L}**S**]$_T$ = 600 mM)

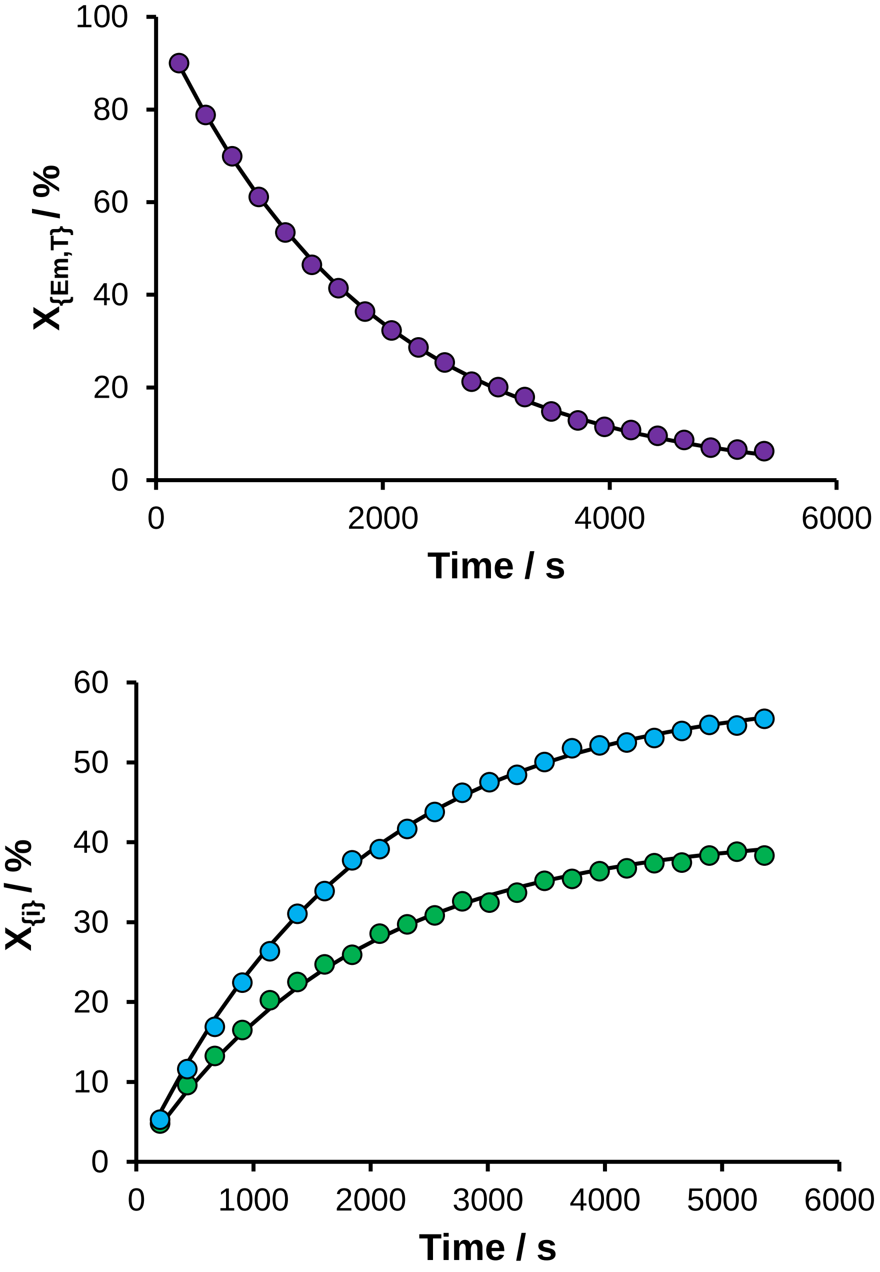

Figure S15: Reaction profiles, expressed in terms of mole fractions (X{i}), for the aminolysis and concurrent hydrolysis of **MepA-L-PheF** (**E**$_m$) in D_2O under the title conditions (20 °C; I = 2.0 M, KCl), as measured by in situ $^{19}F\{^1H\}$ NMR spectroscopy. The amide **P**$_{Am}$ is the major product; the hydrolysis product, **P**$_{aa}$, is the minor product. Fit to kinetic model 1 shown. ^{L}**S** = L-serinamide.

pH*(20°C) = 7.960 ([^{L}**S**]$_T$ = 600 mM)

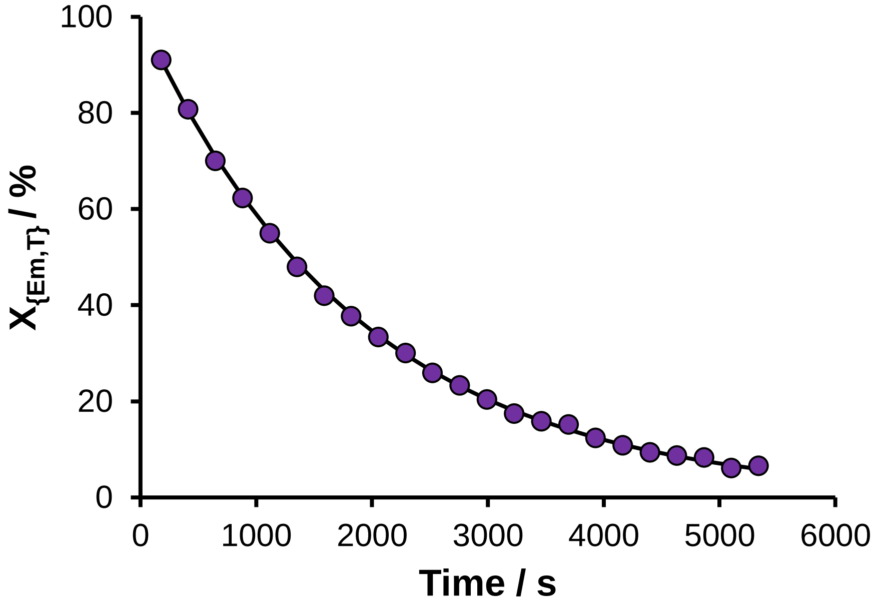

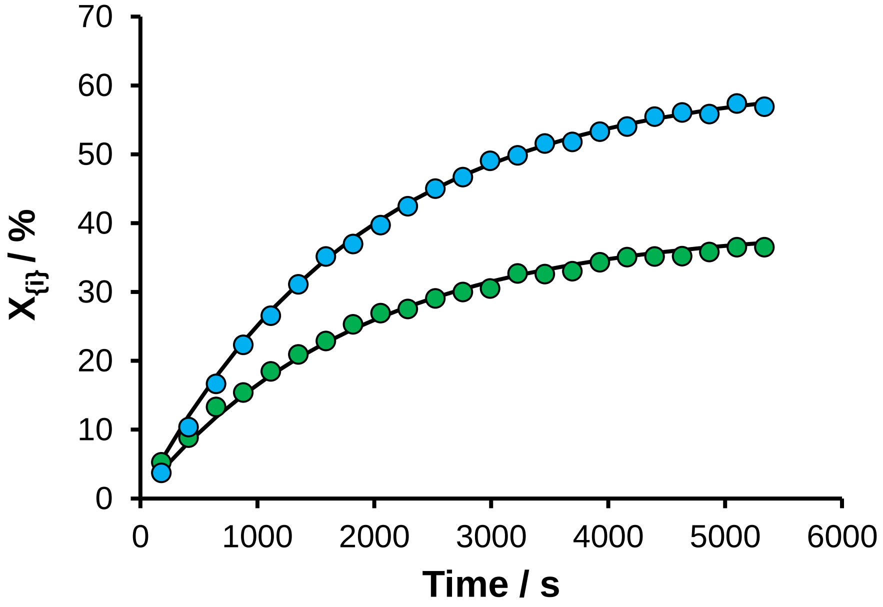

Figure S16: Reaction profiles, expressed in terms of mole fractions (X{i}), for the aminolysis and concurrent hydrolysis of **MepA-L-PheF** (**E$_m$**) in D_2O under the title conditions (20 °C; *I* = 2.0 M, KCl), as measured by in situ $^{19}F\{^1H\}$ NMR spectroscopy. The amide **P$_{Am}$** is the major product; the hydrolysis product, **P$_{aa}$,** is the minor product. Fit to kinetic model 1 shown. ^{L}**S** = L-serinamide.

pH*(20°C) = 7.770 ([^{L}**S**]$_T$ = 600 mM)

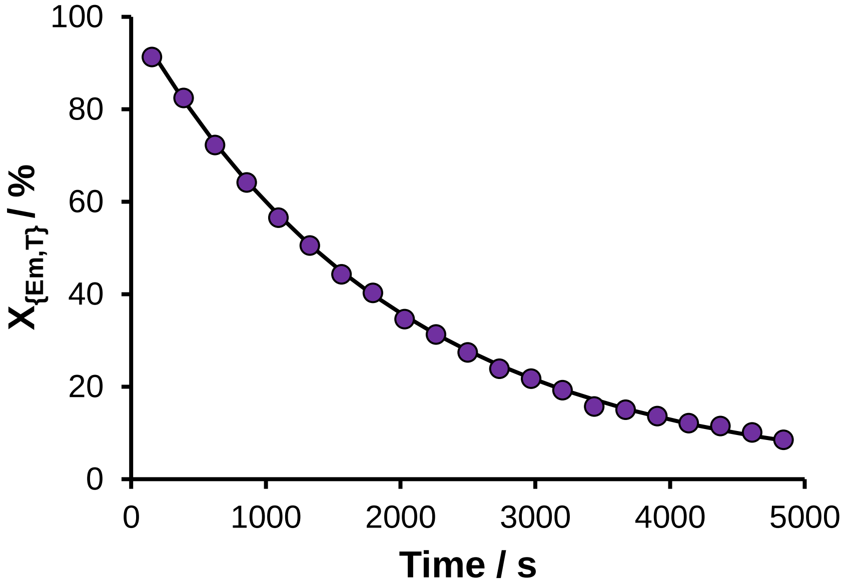

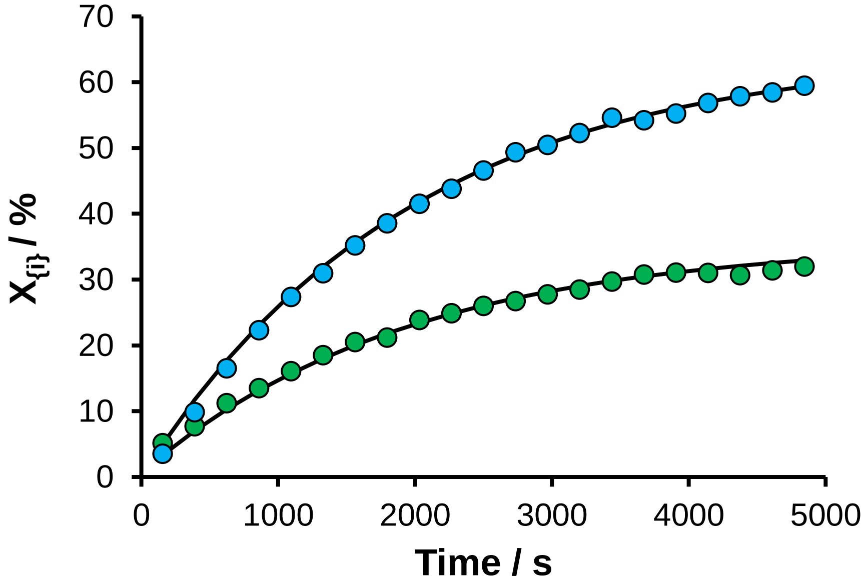

Figure S17: Reaction profiles, expressed in terms of mole fractions (X{i}), for the aminolysis and concurrent hydrolysis of **MepA-L-PheF** (**E**$_m$) in D_2O under the title conditions (20 °C; *I* = 2.0 M, KCl), as measured by in situ $^{19}F\{^1H\}$ NMR spectroscopy. The amide **P**$_{Am}$ is the major product; the hydrolysis product, **P**$_{aa}$, is the minor product. Fit to kinetic model 1 shown. ^{L}**S** = L-serinamide.

pH*(20°C) = 7.339 ([^{L}**S**]$_T$ = 600 mM)

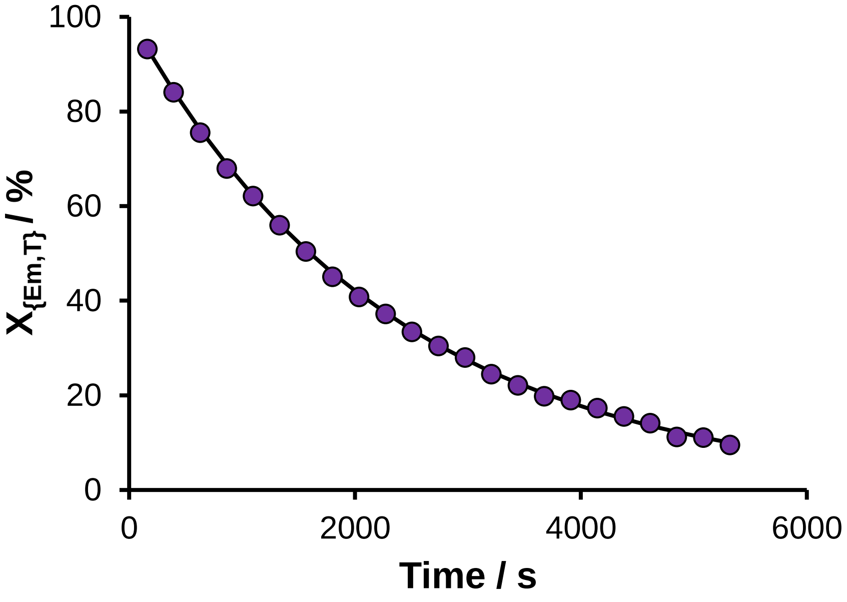

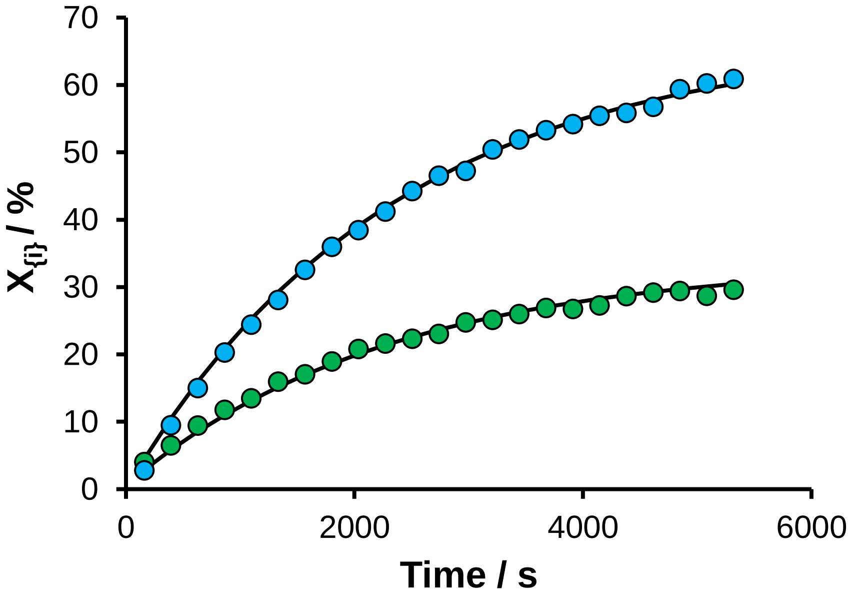

Figure S18: Reaction profiles, expressed in terms of mole fractions (X{i}), for the aminolysis and concurrent hydrolysis of **MepA-L-PheF** (**E**$_m$) in D_2O under the title conditions (20 °C; I = 2.0 M, KCl), as measured by in situ $^{19}F\{^1H\}$ NMR spectroscopy. The amide **P**$_{Am}$ is the major product; the hydrolysis product, **P**$_{aa}$, is the minor product. Fit to kinetic model 1 shown. ^{L}**S** = L-serinamide.

pH*(20°C) = 6.792 ([^{L}**S**]$_T$ = 600 mM)

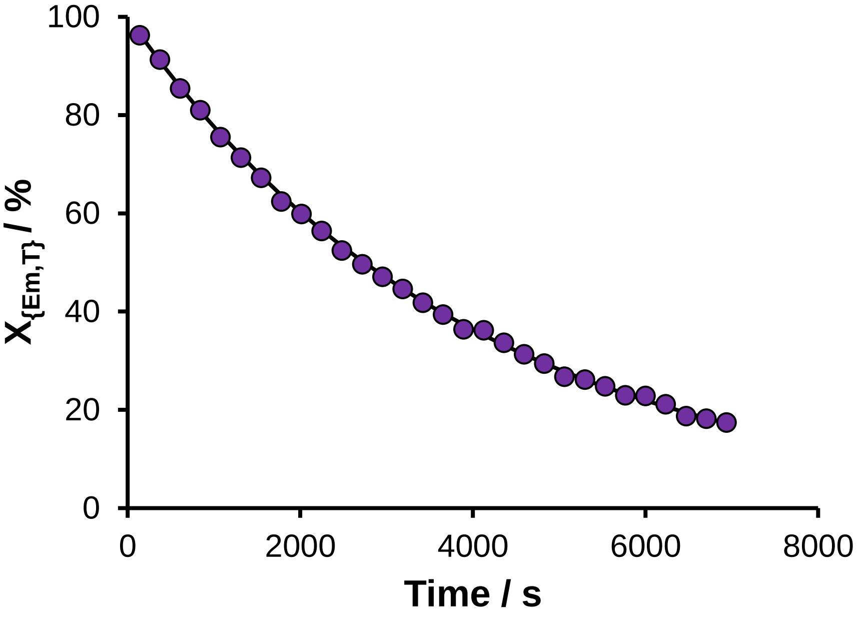

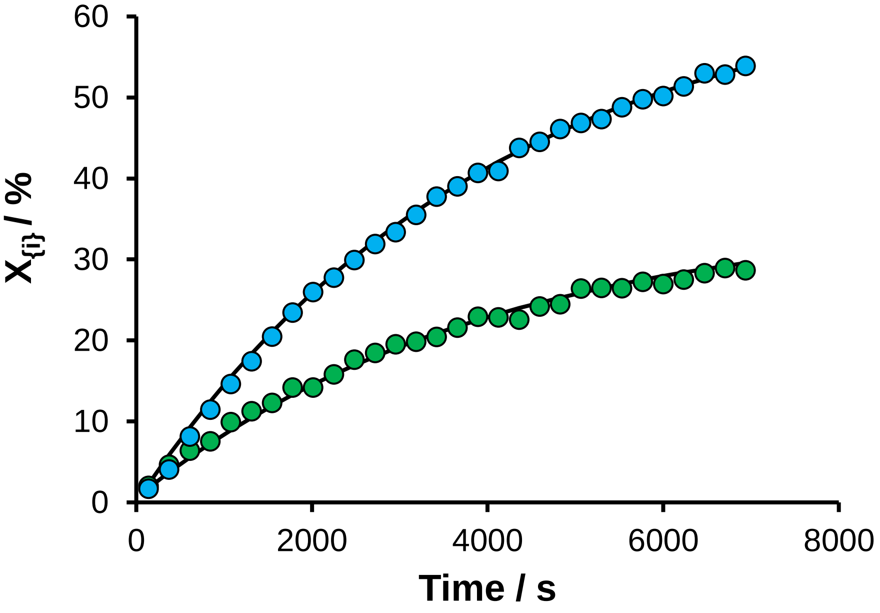

Figure S19: Reaction profiles, expressed in terms of mole fractions (X{i}), for the aminolysis and concurrent hydrolysis of **MepA-L-PheF** (**E$_m$**) in D_2O under the title conditions (20 °C; *I* = 2.0 M, KCl), as measured by in situ ^{19}F{^{1}H} NMR spectroscopy. The amide **P$_{Am}$** is the major product; the hydrolysis product, **P$_{aa}$**, is the minor product. Fit to kinetic model 1 shown. ^{L}**S** = L-serinamide.

pH*(20°C) = 6.449 ([^{L}**S**]$_T$ = 600 mM)

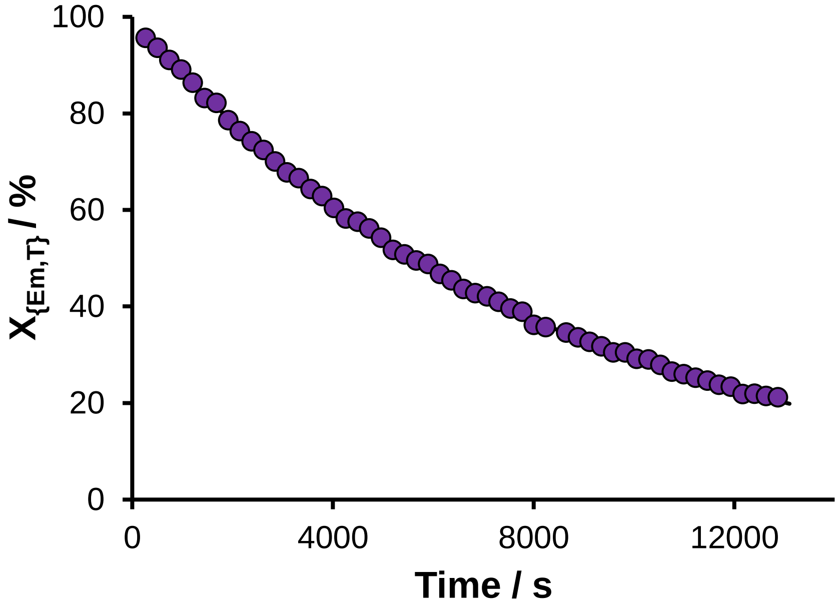

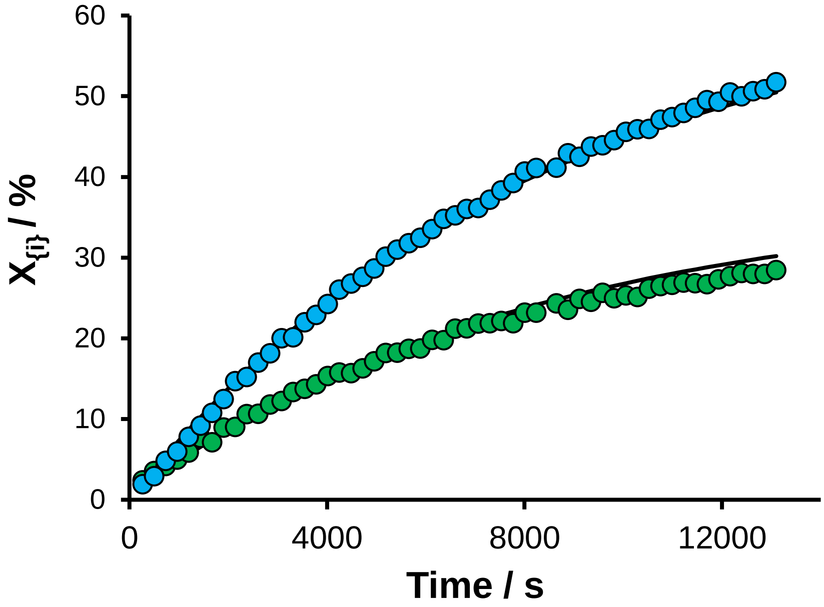

Figure S20: Reaction profiles, expressed in terms of mole fractions (X{i}), for the aminolysis and concurrent hydrolysis of **MepA-L-PheF** (**E**$_m$) in D_2O under the title conditions (20 °C; *I* = 2.0 M, KCl), as measured by in situ $^{19}F\{^1H\}$ NMR spectroscopy. The amide **P**$_{Am}$ is the major product; the hydrolysis product, **P**$_{aa}$, is the minor product. Fit to kinetic model 1 shown. ^{L}**S** = L-serinamide.

pH*(20°C) = 8.421 ([^{L}**S**]$_T$ = 1200 mM)

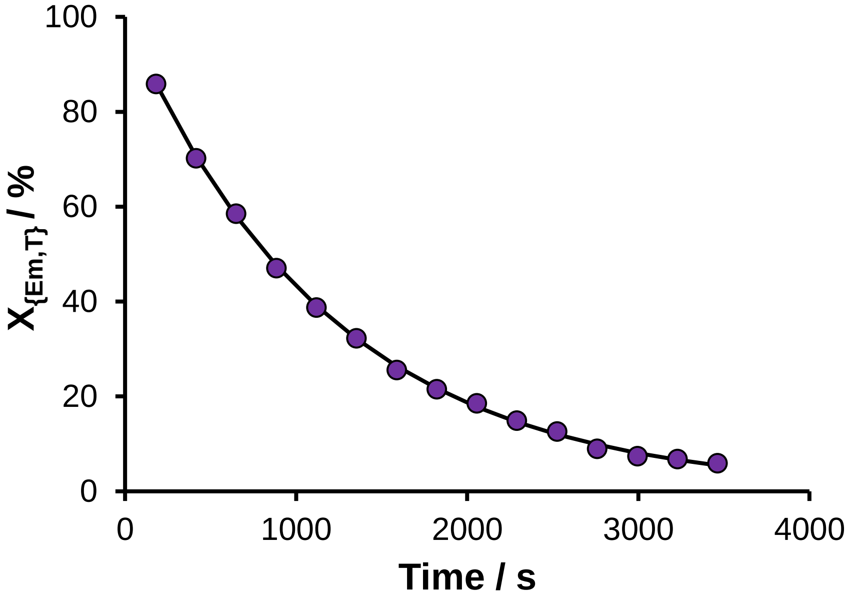

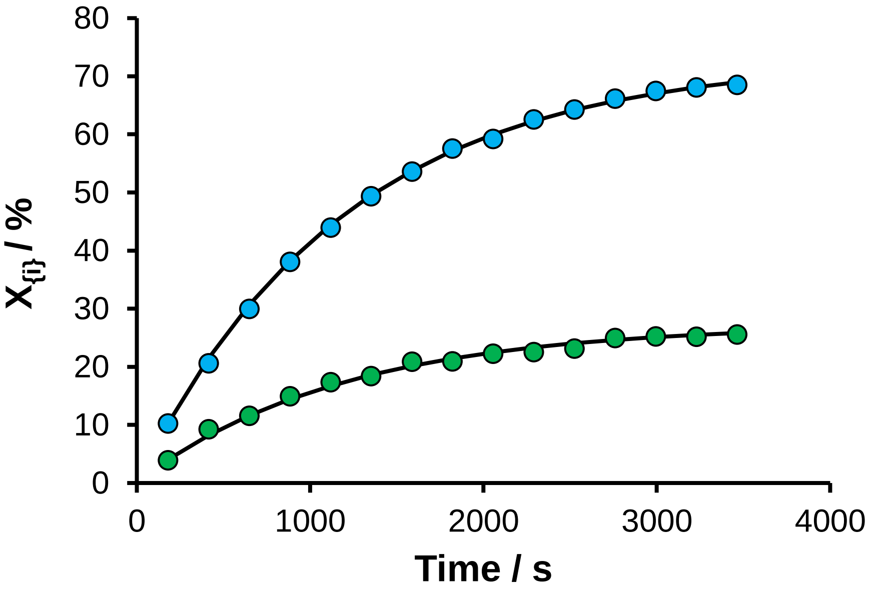

Figure S21: Reaction profiles, expressed in terms of mole fractions (X{i}), for the aminolysis and concurrent hydrolysis of **MepA-L-PheF** (**E$_m$**) in D_2O under the title conditions (20 °C; *I* = 2.0 M, KCl), as measured by in situ ^{19}F{^{1}H} NMR spectroscopy. The amide **P$_{Am}$** is the major product; the hydrolysis product, **P$_{aa}$,** is the minor product. Fit to kinetic model 1 shown. ^{L}**S** = L-serinamide.

pH*(20°C) = 8.081 ([^{L}**S**]$_T$ = 1200 mM)

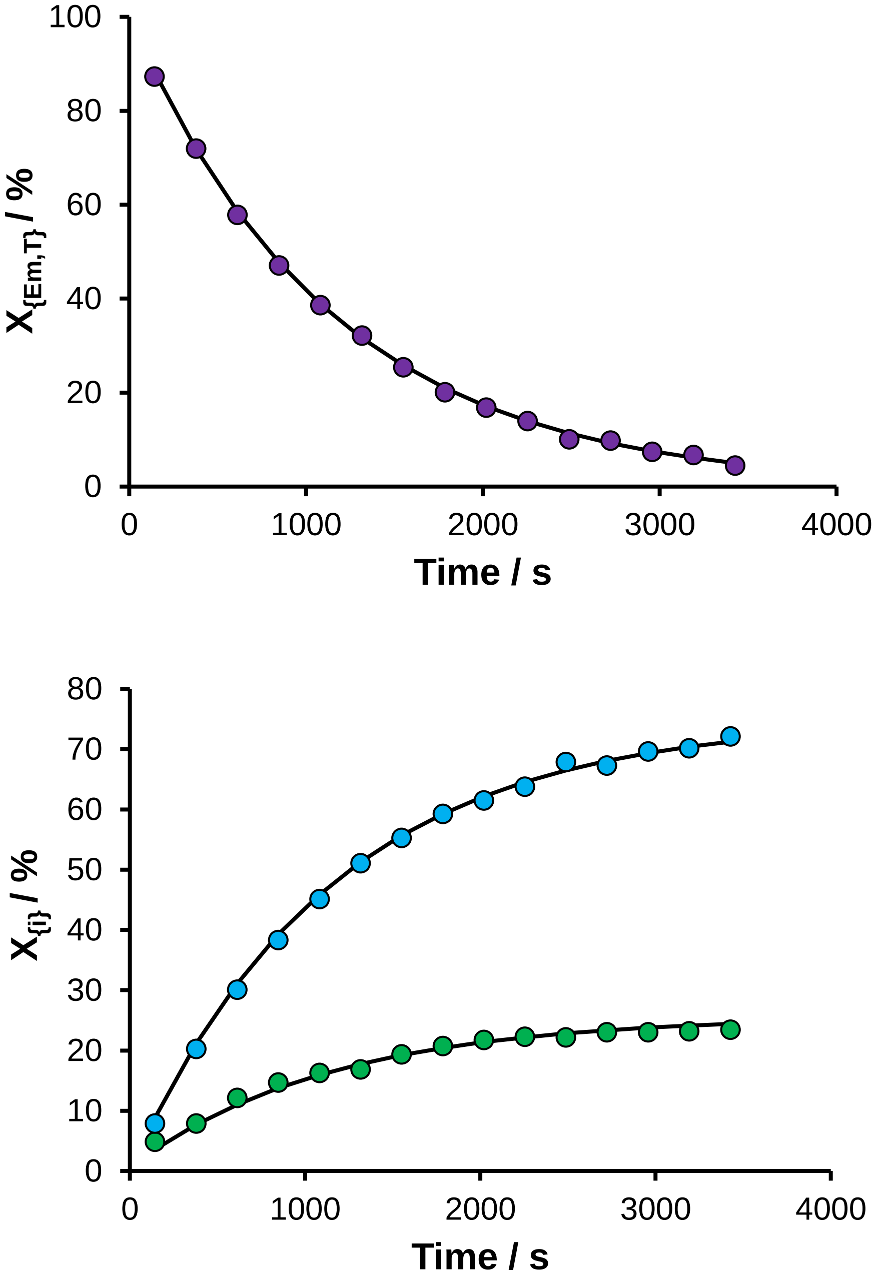

Figure S22: Reaction profiles, expressed in terms of mole fractions (X{i}), for the aminolysis and concurrent hydrolysis of **MepA-L-PheF** (**E**$_m$) in D_2O under the title conditions (20 °C; *I* = 2.0 M, KCl), as measured by in situ $^{19}F\{^1H\}$ NMR spectroscopy. The amide **P**$_{Am}$ is the major product; the hydrolysis product, **P**$_{aa}$, is the minor product. Fit to kinetic model 1 shown. ^{L}**S** = L-serinamide.

pH*(20°C) = 7.777 ([^{L}**S**]$_T$ = 1200 mM)

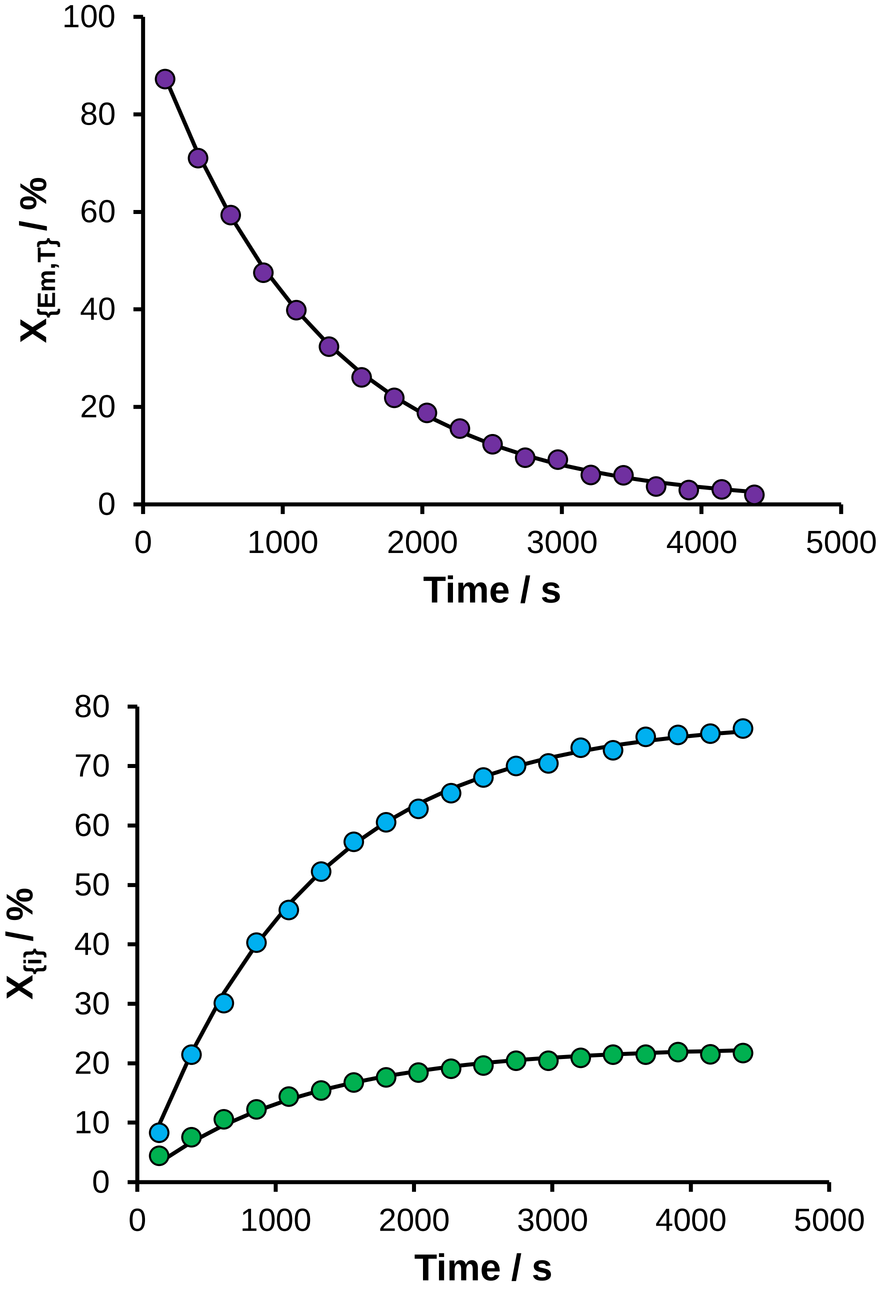

Figure S23: Reaction profiles, expressed in terms of mole fractions (X{i}), for the aminolysis and concurrent hydrolysis of **MepA-L-PheF** (**E$_m$**) in D_2O under the title conditions (20 °C; I = 2.0 M, KCl), as measured by in situ $^{19}F\{^1H\}$ NMR spectroscopy. The amide **P$_{Am}$** is the major product; the hydrolysis product, **P$_{aa}$**, is the minor product. Fit to kinetic model 1 shown. ^{L}**S** = L-serinamide.

pH*(20°C) = 7.440 ([^{L}**S**]$_T$ = 1200 mM)

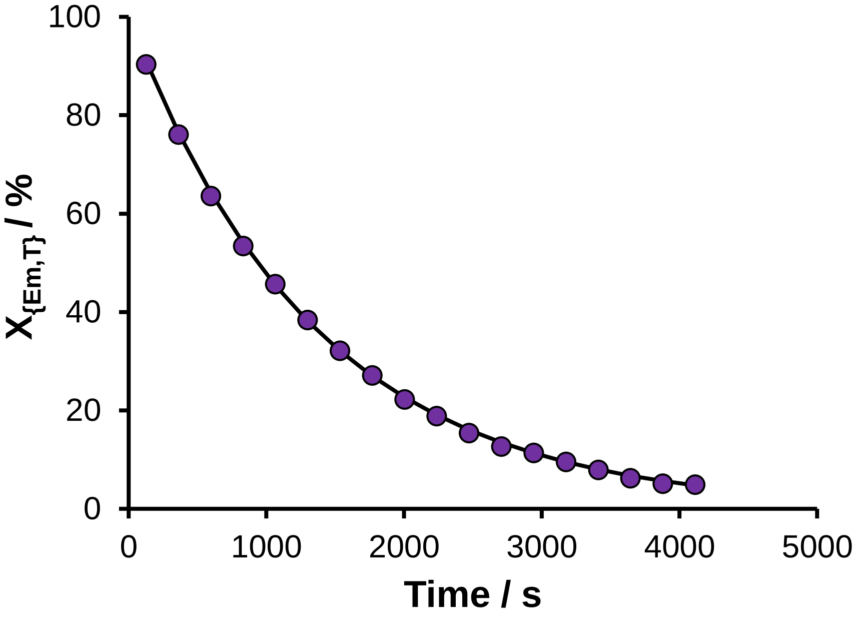

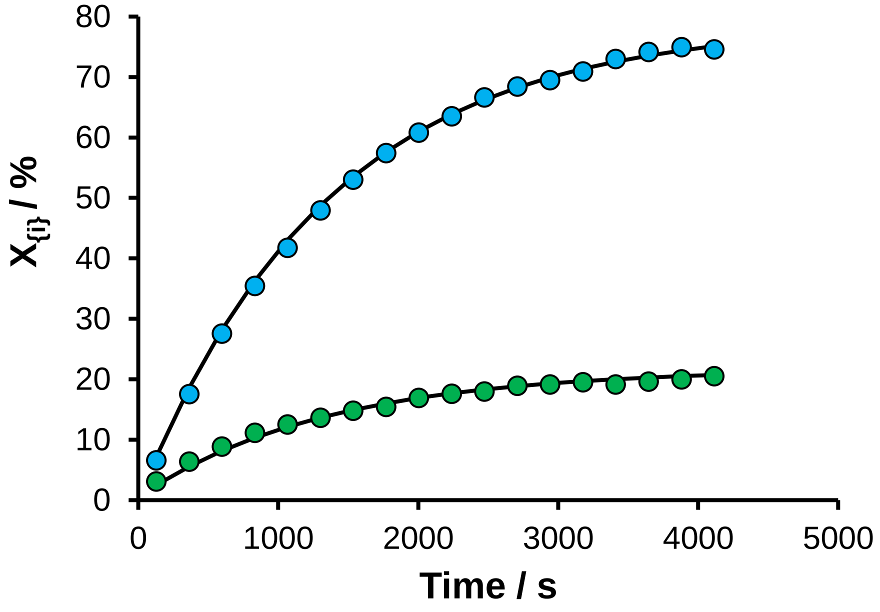

Figure S24: Reaction profiles, expressed in terms of mole fractions (X{i}), for the aminolysis and concurrent hydrolysis of **MepA-L-PheF** (**E**$_m$) in D_2O under the title conditions (20 °C; *I* = 2.0 M, KCl), as measured by in situ $^{19}F\{^1H\}$ NMR spectroscopy. The amide **P**$_{Am}$ is the major product; the hydrolysis product, **P**$_{aa}$, is the minor product. Fit to kinetic model 1 shown. ^{L}**S** = L-serinamide.

pH*(20°C) = 6.960 ([^{L}**S**]$_T$ = 1200 mM)

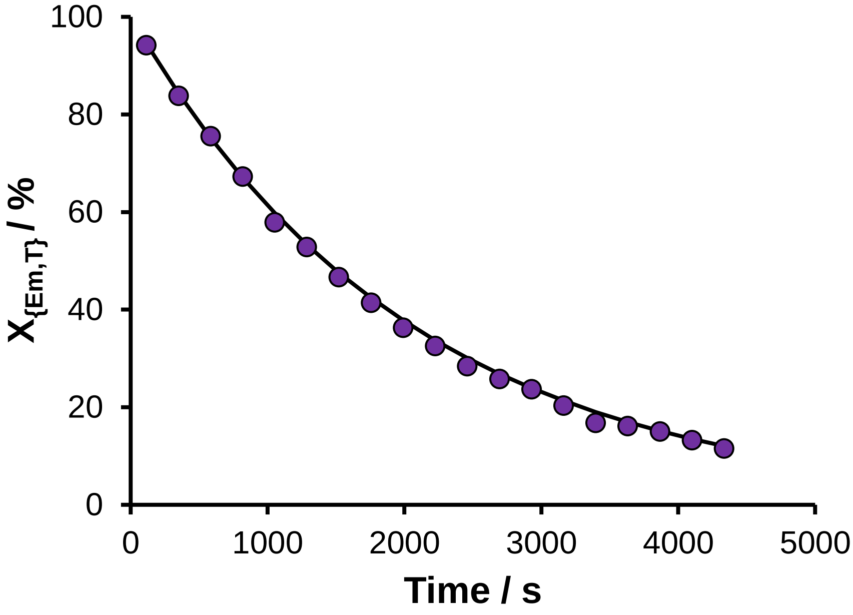

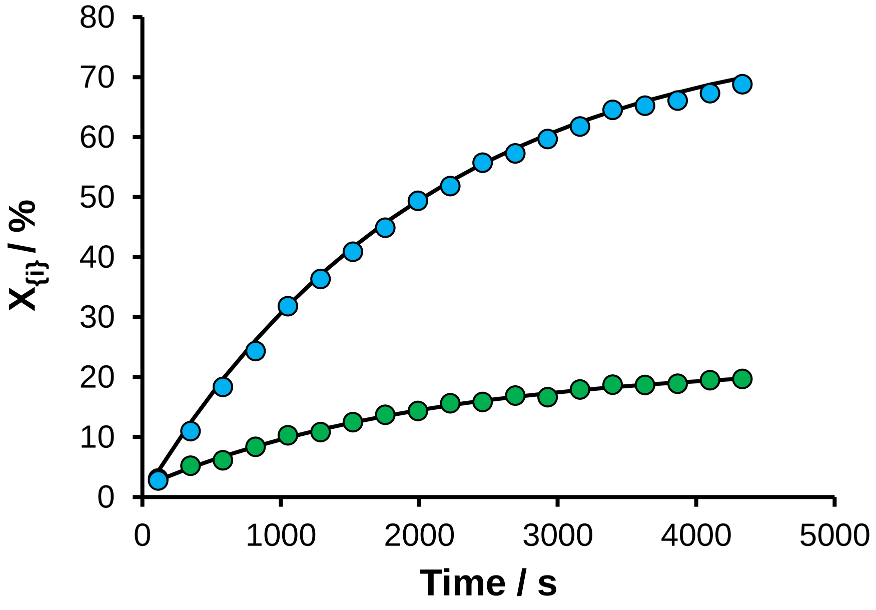

Figure S25: Reaction profiles, expressed in terms of mole fractions (X{i}), for the aminolysis and concurrent hydrolysis of **MepA-L-PheF** (**E**$_m$) in D_2O under the title conditions (20 °C; I = 2.0 M, KCl), as measured by in situ $^{19}F\{^{1}H\}$ NMR spectroscopy. The amide **P**$_{Am}$ is the major product; the hydrolysis product, **P**$_{aa}$, is the minor product. Fit to kinetic model 1 shown. ^{L}**S** = L-serinamide.

pH*(20°C) = 6.504 ([^{L}**S**]$_T$ = 1200 mM)

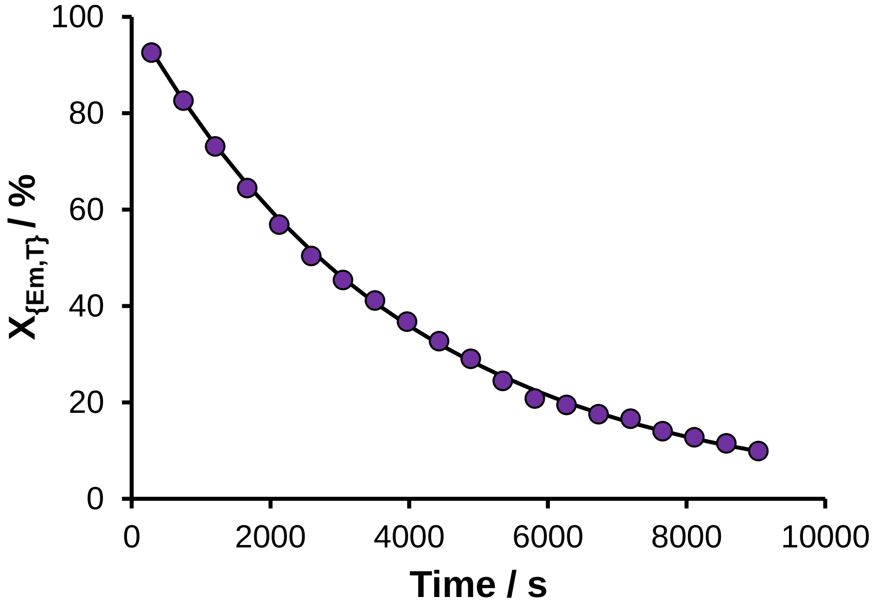

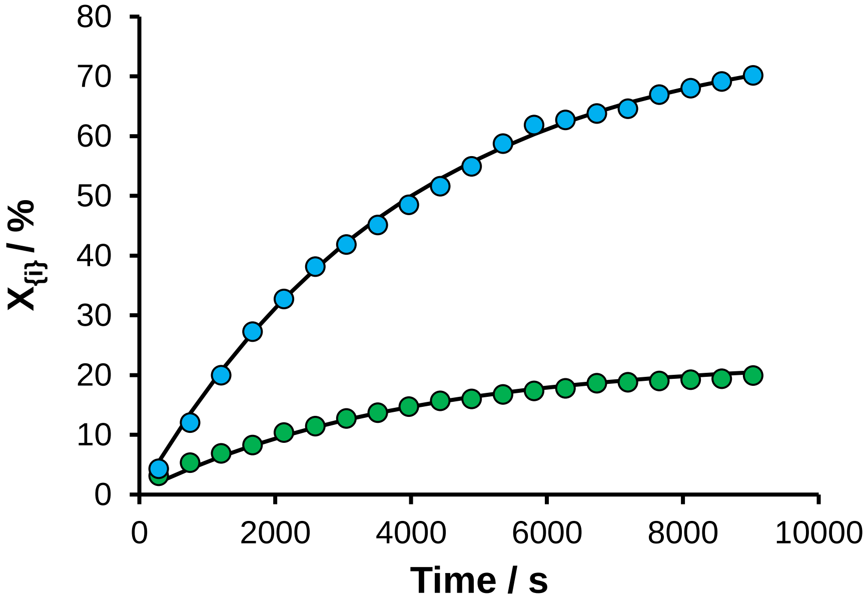

Figure S26: Reaction profiles, expressed in terms of mole fractions (X{i}), for the aminolysis and concurrent hydrolysis of **MepA-L-PheF** (**E$_m$**) in D_2O under the title conditions (20 °C; *I* = 2.0 M, KCl), as measured by in situ ^{19}F{^{1}H} NMR spectroscopy. The amide **P$_{Am}$** is the major product; the hydrolysis product, **P$_{aa}$**, is the minor product. Fit to kinetic model 1 shown. ^{L}**S** = L-serinamide.

pH*(20°C) = 6.012 ([^{L}**S**]$_{\mathrm{T}}$ = 1200 mM)

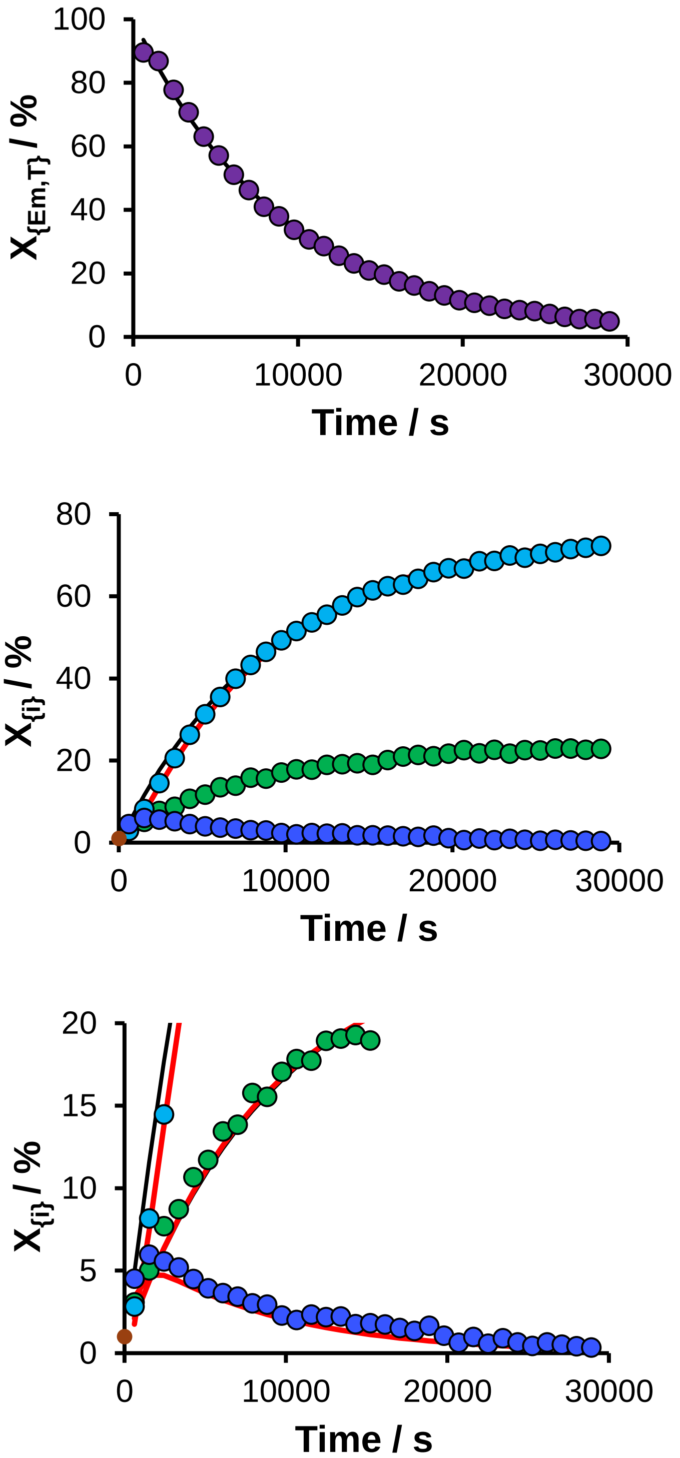

Figure S27: Reaction profiles, expressed in terms of mole fractions (X{i}), for the aminolysis and concurrent hydrolysis of **MepA-L-PheF** (**E**$_{\mathrm{m}}$) in D_2O under the title conditions (20 °C; *I* = 2.0 M, KCl), as measured by in situ ^{19}F{^{1}H} NMR spectroscopy. The amide **P**$_{\mathrm{Am}}$ is the major product; the hydrolysis product, **P**$_{\mathrm{aa}}$, is the minor product. The intermediate is assigned as **I**$_{\mathrm{Es}}$. Fit to kinetic model 2 shown. ^{L}**S** = L-serinamide.

MepA-L-PheF (**E$_m$**) + L-serinamide (**^{L}S**); H_2O, 20 °C

Summary of pseudo first-order rate constants

**pH*(20°C)**	**[^{L}S]$_T$ / mM**	$k^{\Psi}_{Am} \times 10^4$ / s^{-1}	$k'_{Am} \times 10^4$ / (M^{-1} s^{-1})	$k^{\Psi}_{Hyd} \times 10^4$ / s^{-1}
8.912	1200	6.21	5.18	3.97
8.708	1200	5.92	4.93	3.29
8.376	1200	6.31	5.25	2.95
7.979	1200	6.77	5.64	2.85
7.644	1200	6.87	5.73	2.57
7.342	1200	6.14	5.11	2.19
6.85	1200	4.07	3.39	1.47
6.518	1200	2.18	1.81	0.82
5.908	1200	0.95	0.79	0.36

Table S9: Summary of raw data from the pH*-k'_{Am} and pH*-k^{Ψ}_{Hyd} profiles for the aminolysis/hydrolysis of MepA-L-PheF (**E$_m$**) with L-serinamide (H_2O, 20 °C, I = 2.0 M, KCl).

Raw reaction profiles

pH*(20°C) = 8.912 ([^{L}**S**]$_T$ = 1200 mM)

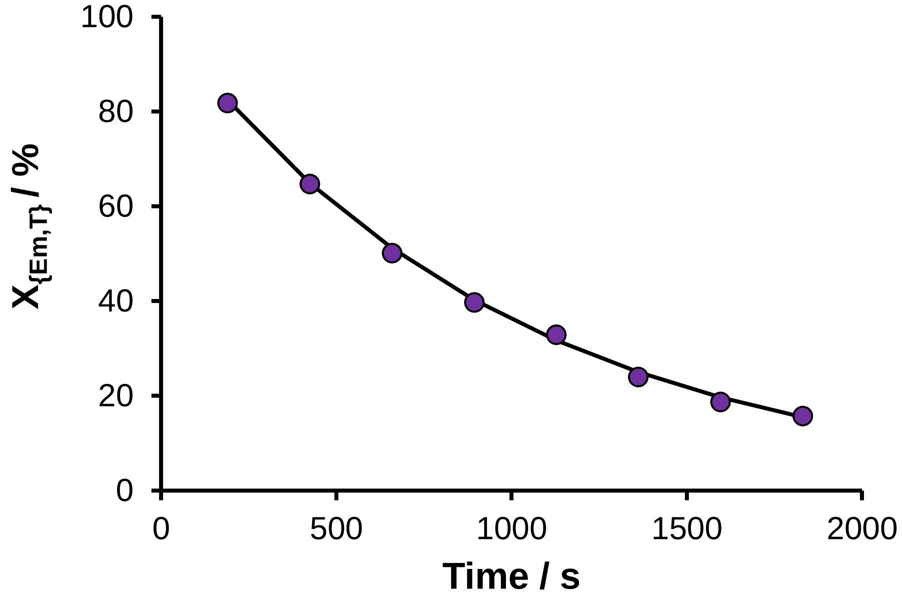

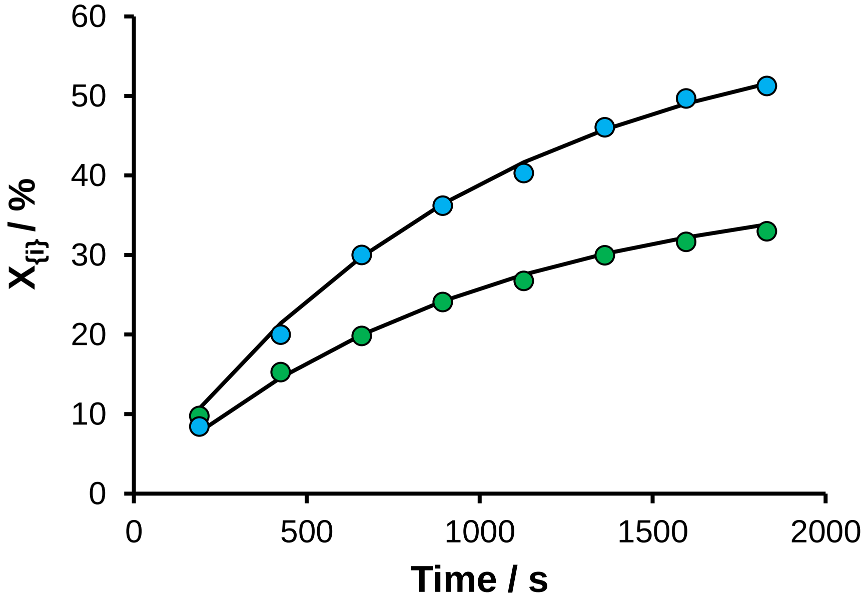

Figure S28: Reaction profiles, expressed in terms of mole fractions (X{i}), for the aminolysis and concurrent hydrolysis of **MepA-L-PheF** (**E$_m$**) in H_2O under the title conditions (20 °C; *I* = 2.0 M, KCl), as measured by in situ $^{19}F\{^1H\}$ NMR spectroscopy. The amide **P$_{Am}$** is the major product; the hydrolysis product, **P$_{aa}$**, is the minor product. Fit to kinetic model 1 shown. ^{L}**S** = L-serinamide.

pH*(20°C) = 8.708 ([^{L}**S**]$_T$ = 1200 mM)

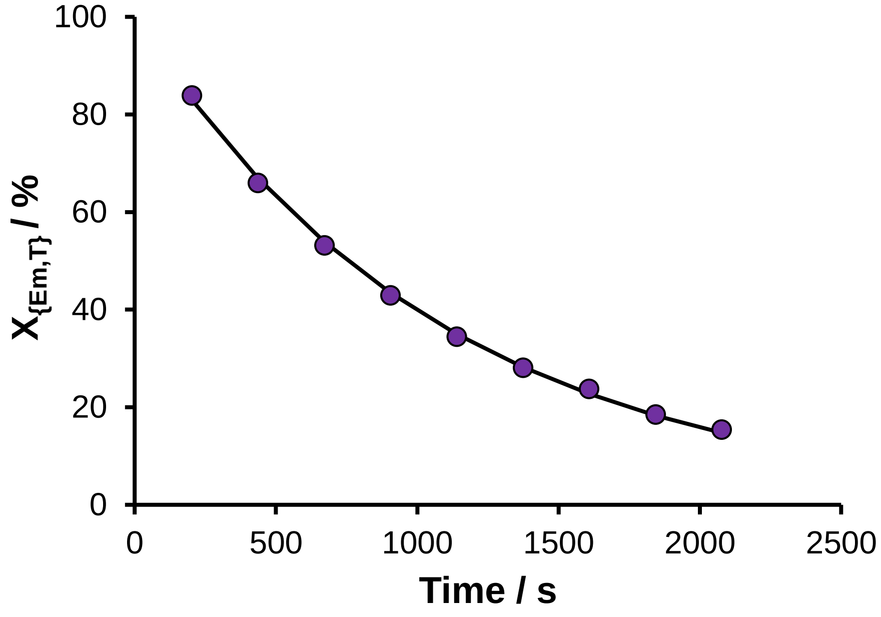

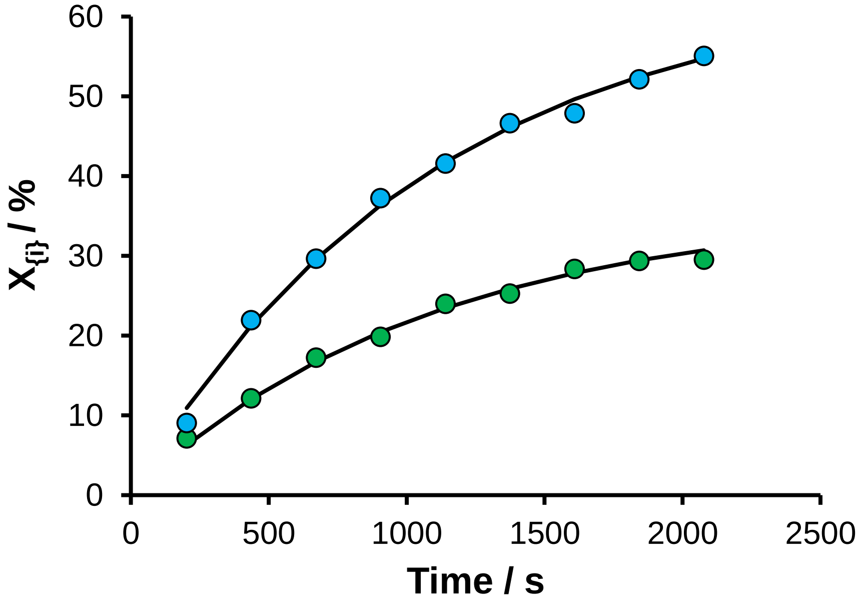

Figure S29: Reaction profiles, expressed in terms of mole fractions (X{i}), for the aminolysis and concurrent hydrolysis of **MepA-L-PheF** (**E$_m$**) in H_2O under the title conditions (20 °C; *I* = 2.0 M, KCl), as measured by in situ $^{19}F\{^1H\}$ NMR spectroscopy. The amide **P$_{Am}$** is the major product; the hydrolysis product, **P$_{aa}$**, is the minor product. Fit to kinetic model 1 shown. ^{L}**S** = L-serinamide.

pH*(20°C) = 8.376 ([ᴸ**S**]$_T$ = 1200 mM)

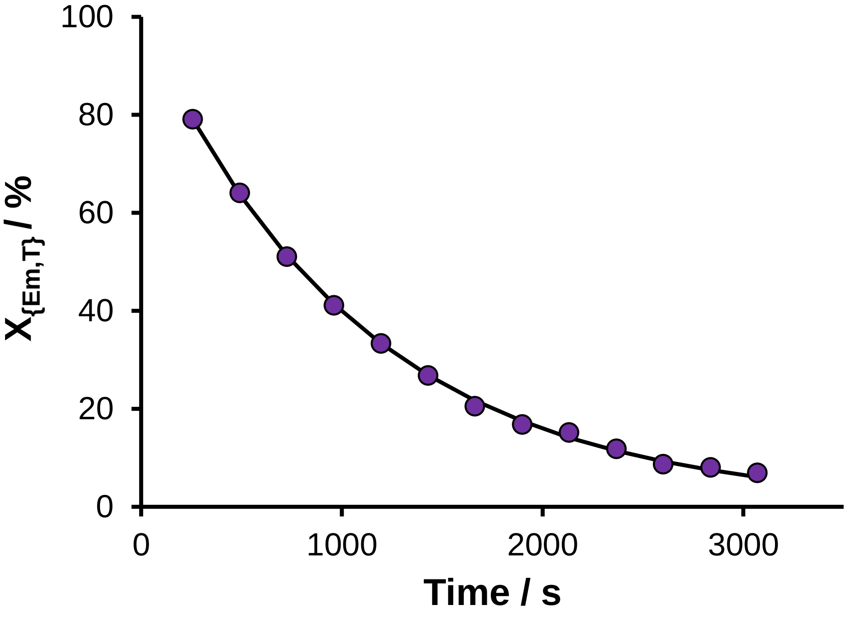

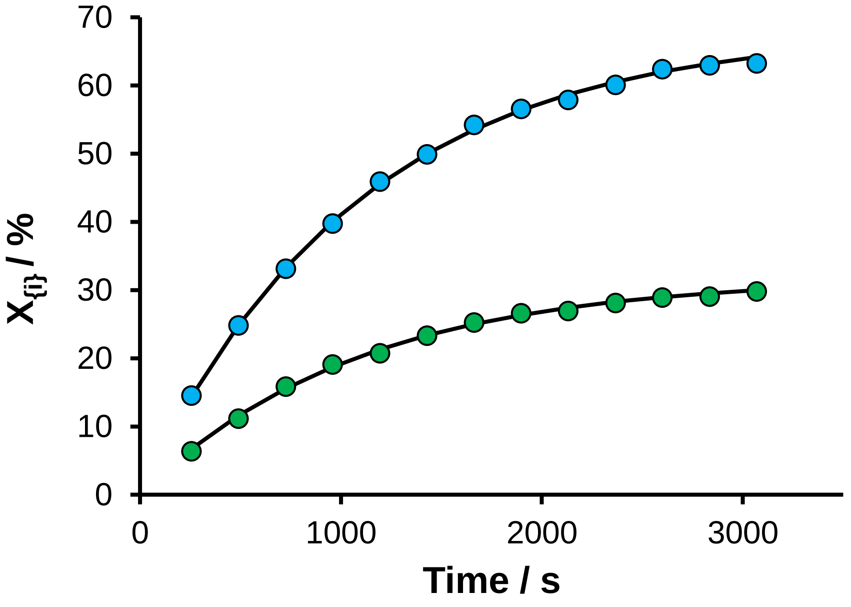

Figure S30: Reaction profiles, expressed in terms of mole fractions (X{i}), for the aminolysis and concurrent hydrolysis of **MepA-L-PheF** (**E**$_m$) in H_2O under the title conditions (20 °C; *I* = 2.0 M, KCl), as measured by in situ $^{19}F\{^1H\}$ NMR spectroscopy. The amide $\mathbf{P}_{Am}$ is the major product; the hydrolysis product, $\mathbf{P}_{aa}$, is the minor product. Fit to kinetic model 1 shown. ᴸ**S** = L-serinamide.

pH*(20°C) = 7.979 ([^{L}**S**]$_T$ = 1200 mM)

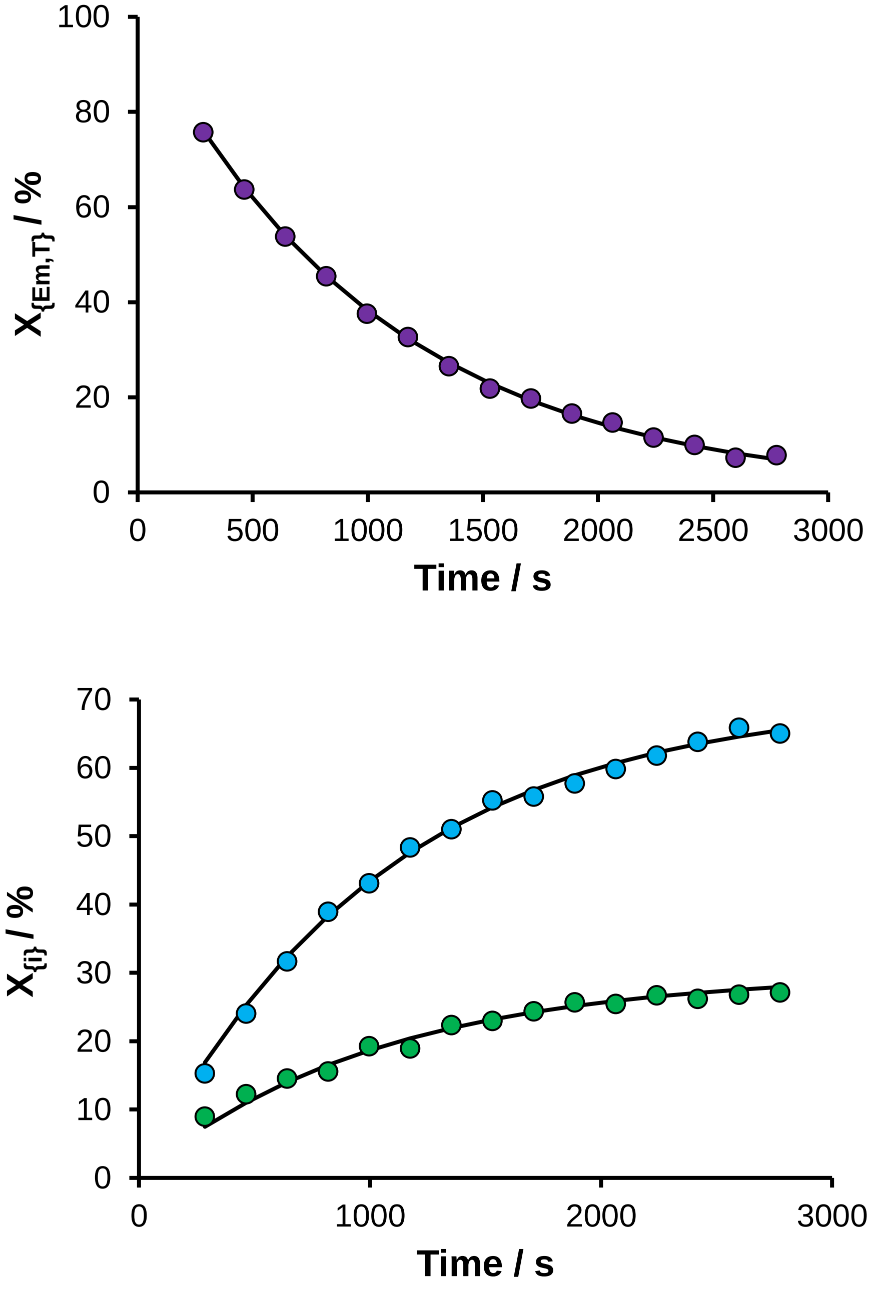

Figure S31: Reaction profiles, expressed in terms of mole fractions (X{i}), for the aminolysis and concurrent hydrolysis of **MepA-L-PheF** (**E**$_m$) in H_2O under the title conditions (20 °C; *I* = 2.0 M, KCl), as measured by in situ $^{19}F\{^1H\}$ NMR spectroscopy. The amide **P**$_{Am}$ is the major product; the hydrolysis product, **P**$_{aa}$, is the minor product. Fit to kinetic model 1 shown. ^{L}**S** = L-serinamide.

pH*(20°C) = 7.644 ([ᴸS]$_T$ = 1200 mM)

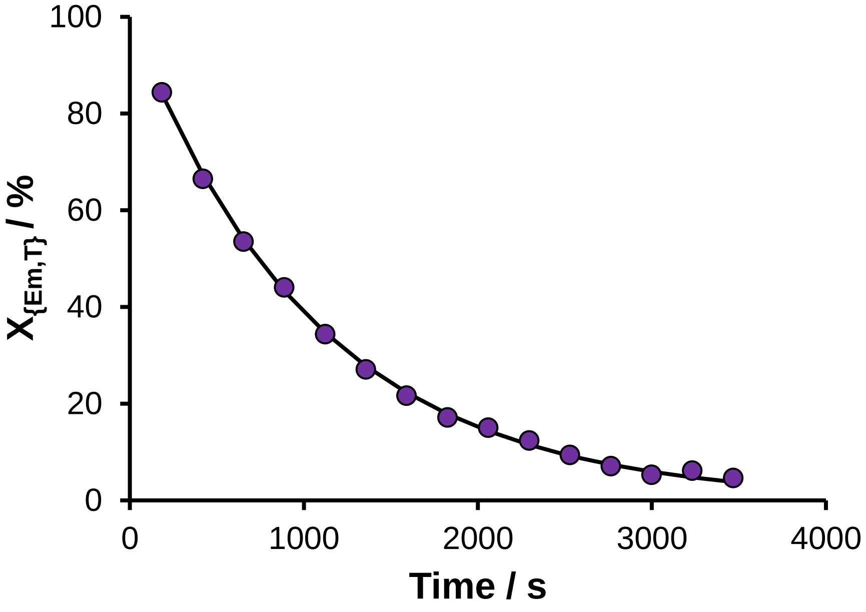

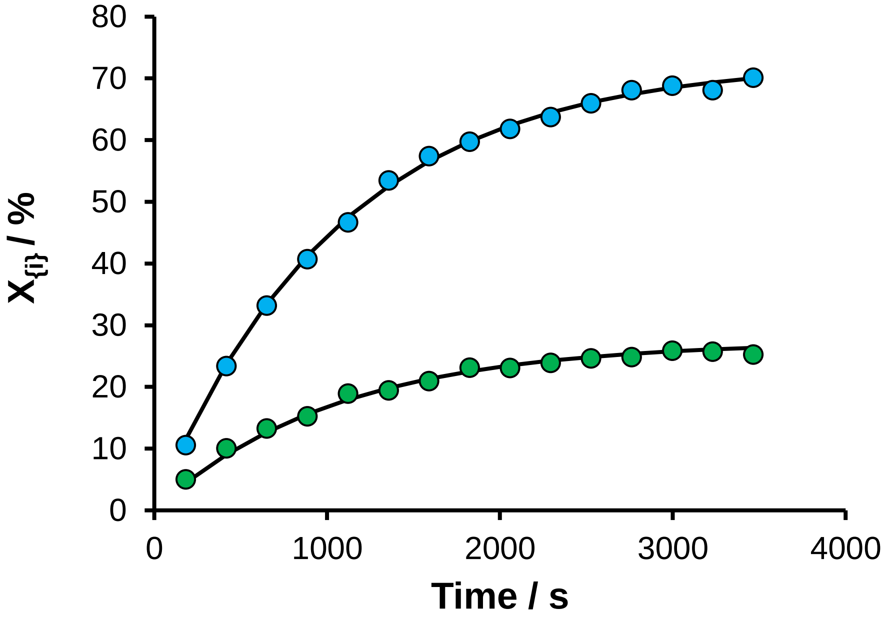

Figure S32: Reaction profiles, expressed in terms of mole fractions (X{i}), for the aminolysis and concurrent hydrolysis of **MepA-L-PheF** (**E$_m$**) in H_2O under the title conditions (20 °C; I = 2.0 M, KCl), as measured by in situ $^{19}F\{^1H\}$ NMR spectroscopy. The amide **P$_{Am}$** is the major product; the hydrolysis product, **P$_{aa}$**, is the minor product. Fit to kinetic model 1 shown. **ᴸS** = L-serinamide.

pH*(20°C) = 7.342 ([^{L}**S**]$_T$ = 1200 mM)

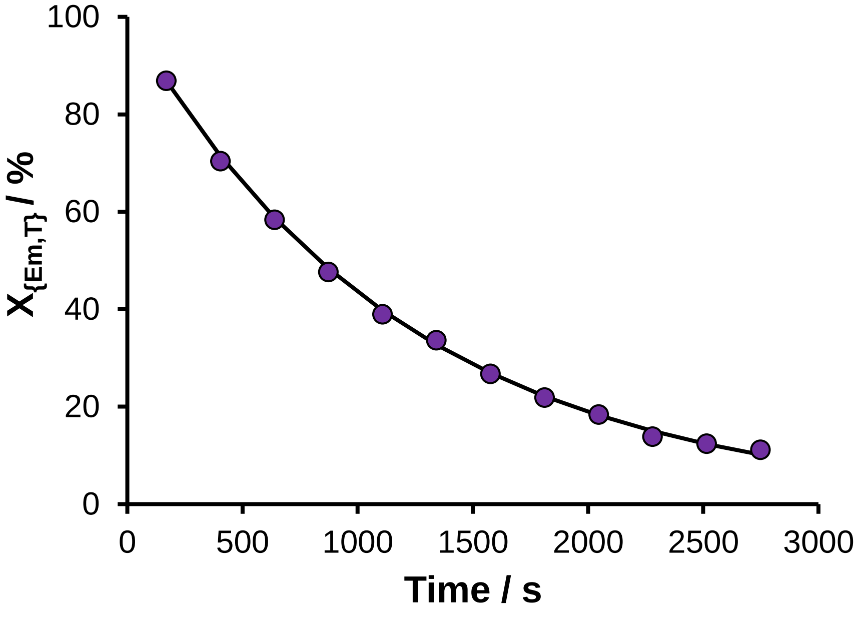

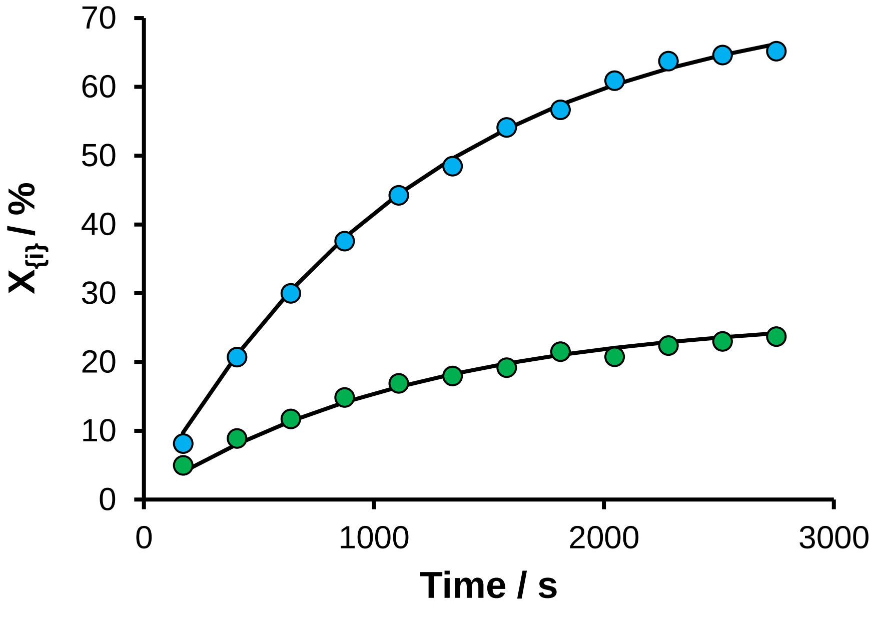

Figure S33: Reaction profiles, expressed in terms of mole fractions (X{i}), for the aminolysis and concurrent hydrolysis of **MepA-L-PheF** (**E$_m$**) in H_2O under the title conditions (20 °C; *I* = 2.0 M, KCl), as measured by in situ $^{19}F\{^1H\}$ NMR spectroscopy. The amide **P$_{Am}$** is the major product; the hydrolysis product, **P$_{aa}$**, is the minor product. Fit to kinetic model 1 shown. ^{L}**S** = L-serinamide.

pH*(20°C) = 6.850 ([^{L}**S**]$_T$ = 1200 mM)

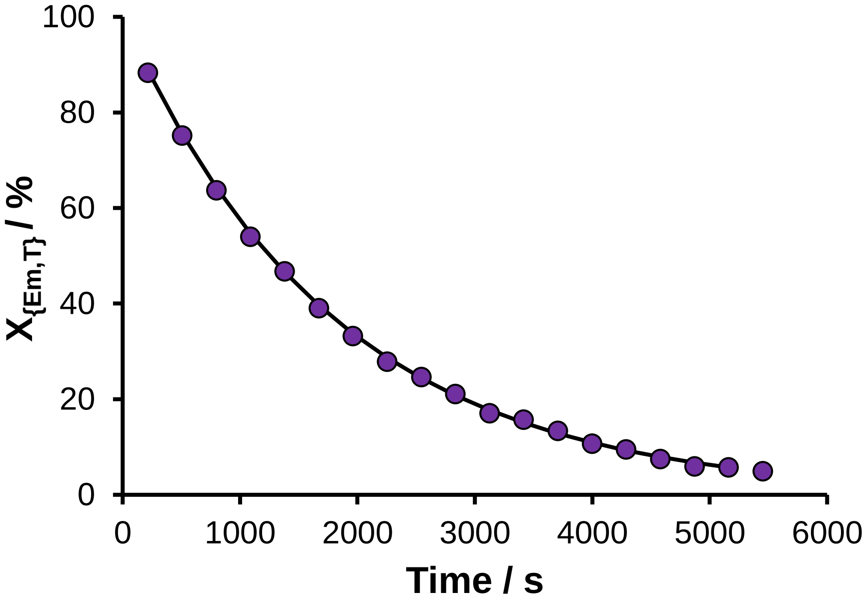

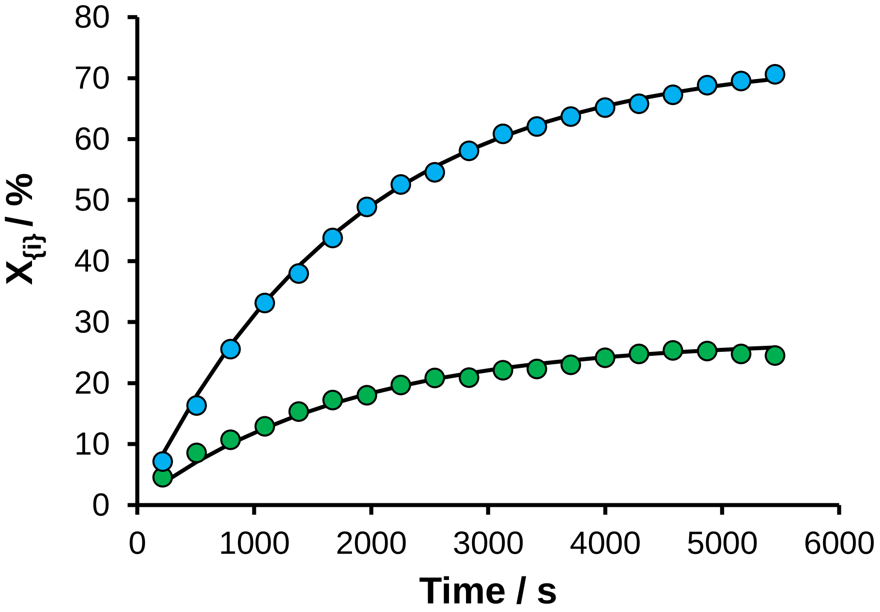

Figure S34: Reaction profiles, expressed in terms of mole fractions (X{i}), for the aminolysis and concurrent hydrolysis of **MepA-L-PheF** (**E**$_m$) in H_2O under the title conditions (20 °C; I = 2.0 M, KCl), as measured by in situ $^{19}F\{^1H\}$ NMR spectroscopy. The amide $\mathbf{P_{Am}}$ is the major product; the hydrolysis product, $\mathbf{P_{aa}}$, is the minor product. Fit to kinetic model 1 shown. ^{L}**S** = L-serinamide.

pH*(20°C) = 6.518 ([^{L}**S**]$_T$ = 1200 mM)

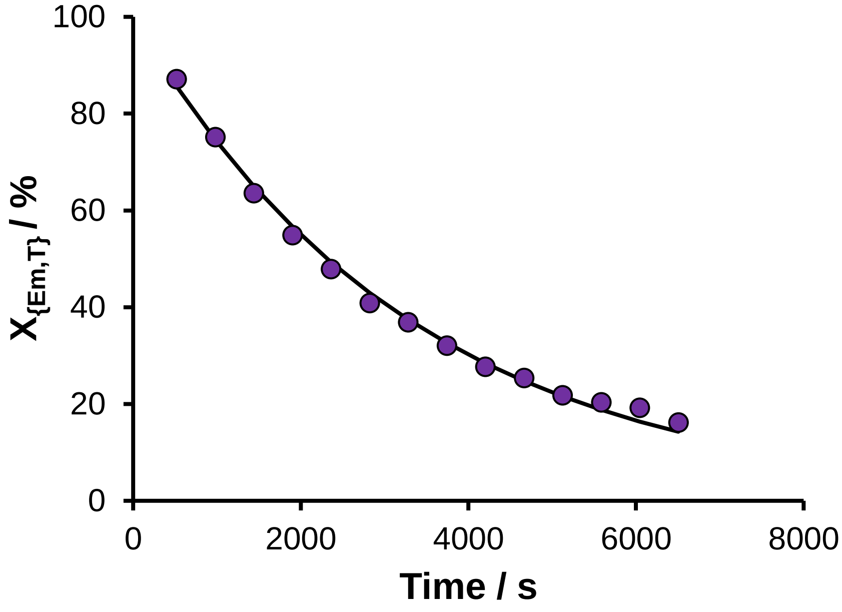

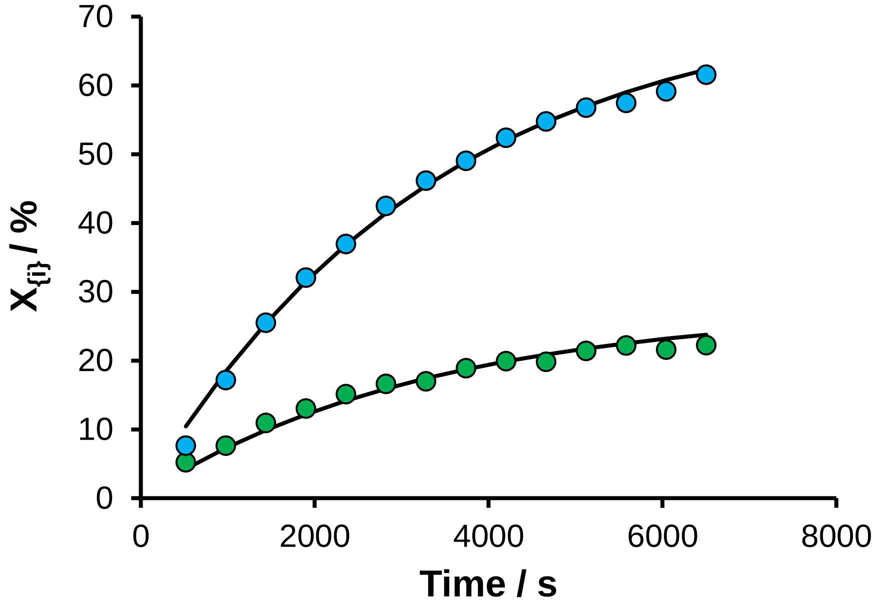

Figure S35: Reaction profiles, expressed in terms of mole fractions (X{i}), for the aminolysis and concurrent hydrolysis of **MepA-L-PheF** (**E**$_m$) in H_2O under the title conditions (20 °C; *I* = 2.0 M, KCl), as measured by in situ $^{19}F\{^1H\}$ NMR spectroscopy. The amide **P**$_{Am}$ is the major product; the hydrolysis product, **P**$_{aa}$, is the minor product. Fit to kinetic model 1 shown. ^{L}**S** = L-serinamide.

pH*(20°C) = 5.908 ([^{L}**S**]$_T$ = 1200 mM)

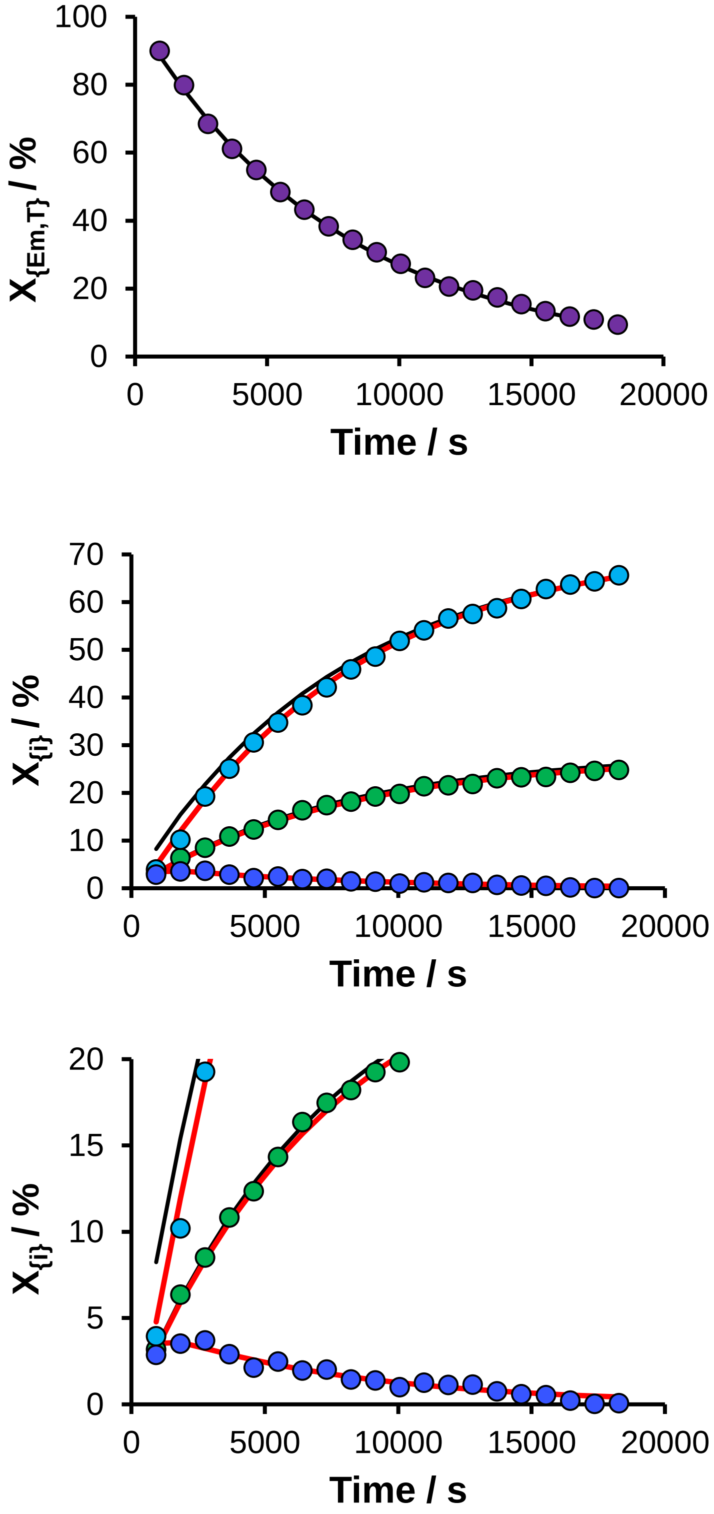

Figure S36: Reaction profiles, expressed in terms of mole fractions (X{i}), for the aminolysis and concurrent hydrolysis of **MepA-L-PheF** (**E$_m$**) in H_2O under the title conditions (20 °C; *I* = 2.0 M, KCl), as measured by in situ $^{19}F\{^1H\}$ NMR spectroscopy. The amide **P$_{Am}$** is the major product; the hydrolysis product, **P$_{aa}$**, is the minor product. The intermediate is assigned as **I$_{Es}$**. Fit to kinetic model 2 shown. ^{L}**S** = L-serinamide.

MepA-L-PheF (**E$_m$**) + L-serinamide (**^{L}S**); D_2O, 10 °C

Summary of pseudo first-order rate constants

pH*(10°C)	[^{L}S]$_T$ / mM	$k^{\Psi}_{Am} \times 10^4$ / s^{-1}	$k'_{Am} \times 10^4$ / (M^{-1} s^{-1})	$k^{\Psi}_{Hyd} \times 10^4$ / s^{-1}
9.415	1200	3.09	2.58	1.44
9.173	1200	3.02	2.51	1.08
8.841	1200	3.36	2.80	1.03
8.506	1200	3.65	3.04	0.98
8.082	1200	3.54	2.95	0.85
7.801	1200	3.22	2.69	0.73
7.29	1200	2.19	1.82	0.48
6.895	1200	1.23	1.03	0.29
6.377	1200	0.51	0.43	0.12

Table S10: Summary of raw data from the pH*-k'_{Am} and pH*-k^{Ψ}_{Hyd} profiles for the aminolysis/hydrolysis of MepA-L-PheF (**E$_m$**) with L-serinamide (D_2O, 10 °C, I = 2.0 M, KCl).

Raw reaction profiles

pH*(10°C) = 9.415 ([^{L}**S**]$_T$ = 1200 mM)

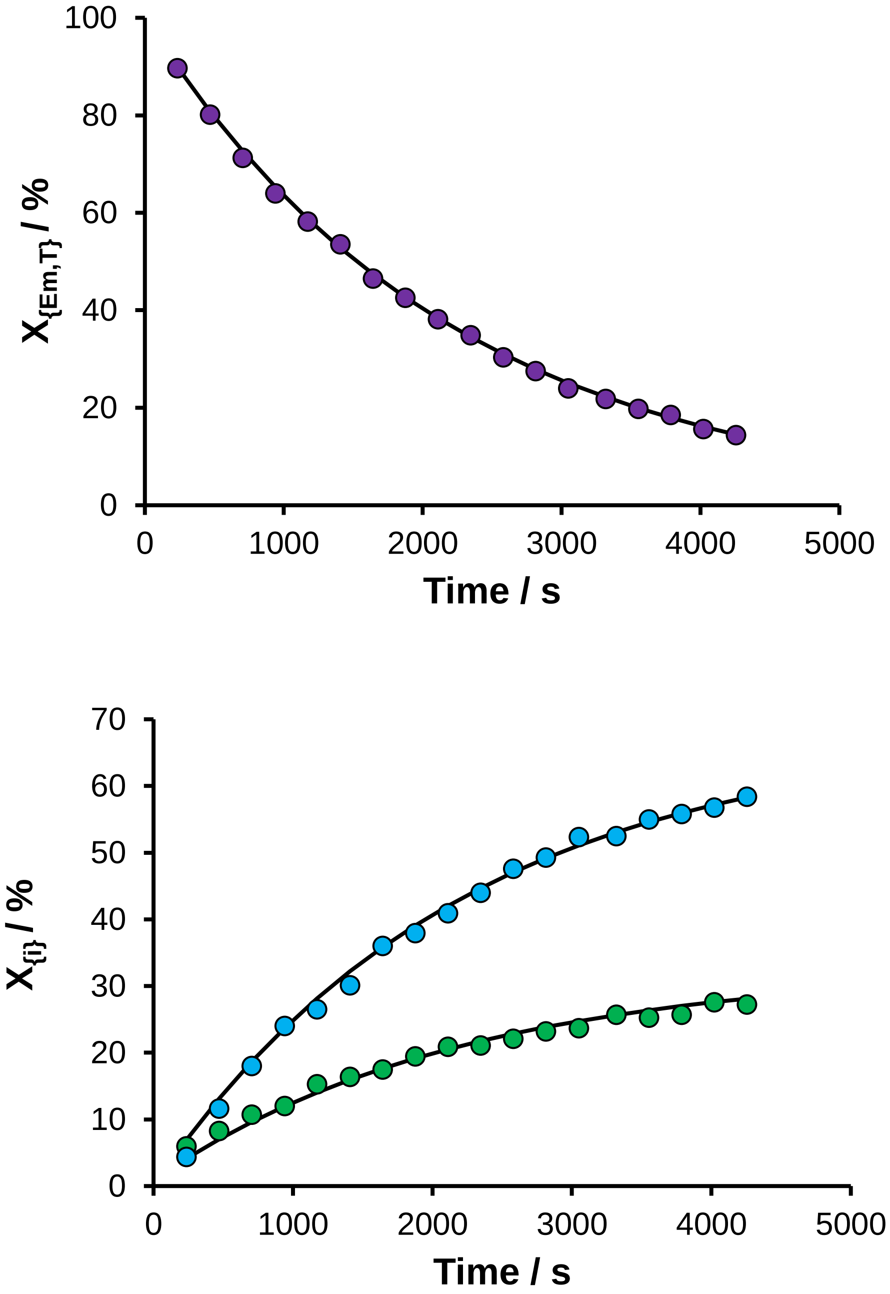

Figure S37: Reaction profiles, expressed in terms of mole fractions (X{i}), for the aminolysis and concurrent hydrolysis of **MepA-L-PheF** (**E$_m$**) in D_2O under the title conditions (10 °C; *I* = 2.0 M, KCl), as measured by in situ $^{19}F\{^{1}H\}$ NMR spectroscopy. The amide **P$_{Am}$** is the major product; the hydrolysis product, **P$_{aa}$**, is the minor product. Fit to kinetic model 1 shown. ^{L}**S** = L-serinamide.

pH*(10°C) = 9.173 ([^{L}**S**]$_T$ = 1200 mM)

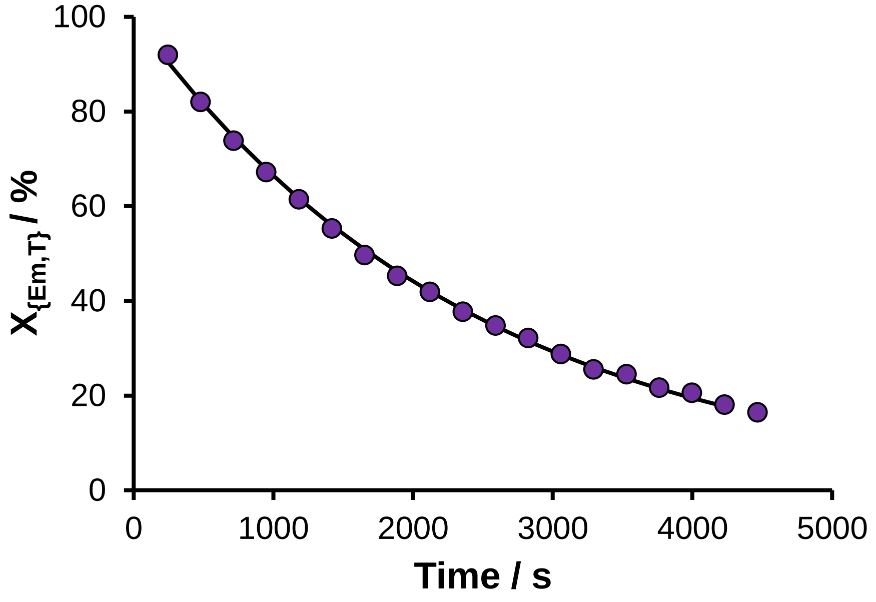

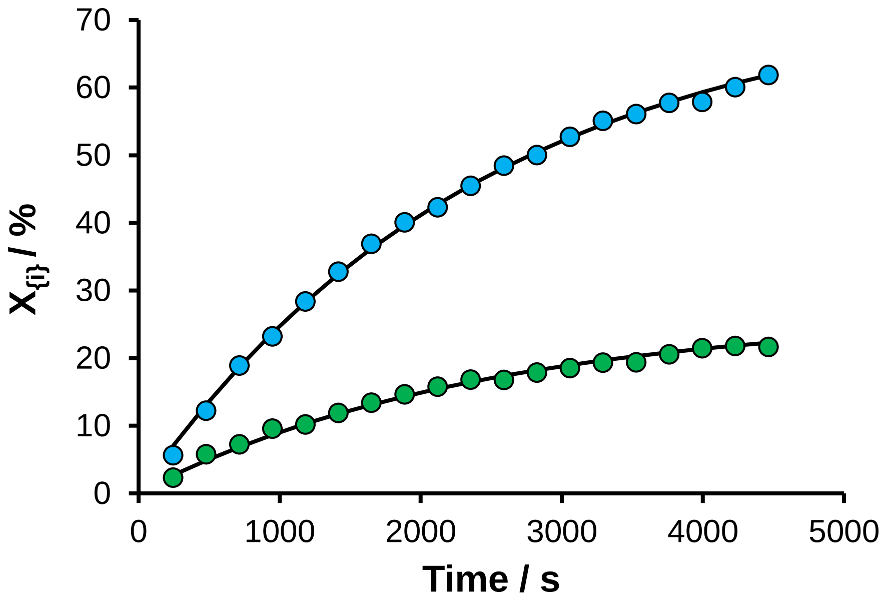

Figure S38: Reaction profiles, expressed in terms of mole fractions (X{i}), for the aminolysis and concurrent hydrolysis of **MepA-L-PheF** (**E$_m$**) in D_2O under the title conditions (10 °C; *I* = 2.0 M, KCl), as measured by in situ $^{19}F\{^1H\}$ NMR spectroscopy. The amide **P$_{Am}$** is the major product; the hydrolysis product, **P$_{aa}$**, is the minor product. Fit to kinetic model 1 shown. ^{L}**S** = L-serinamide.

pH*(10°C) = 8.841 ([^{L}**S**]$_T$ = 1200 mM)

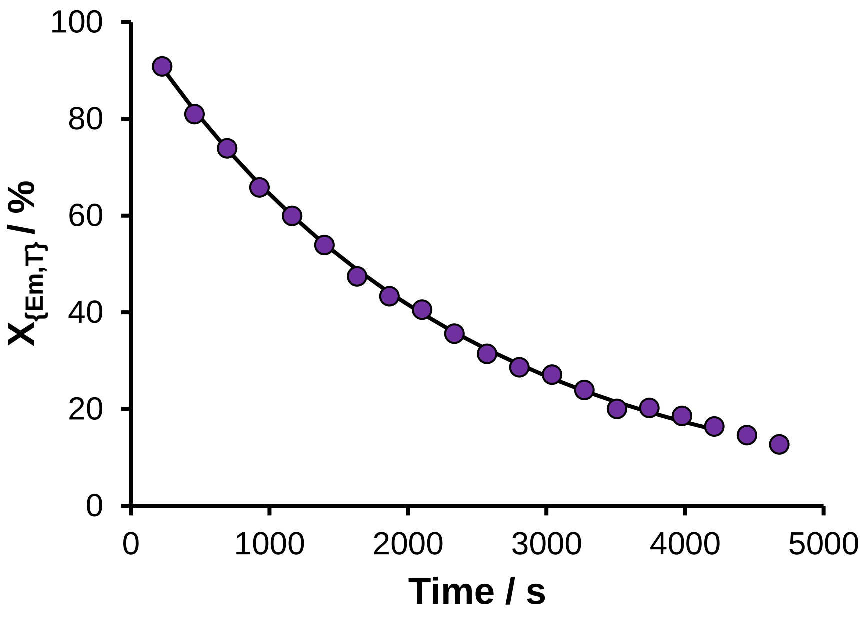

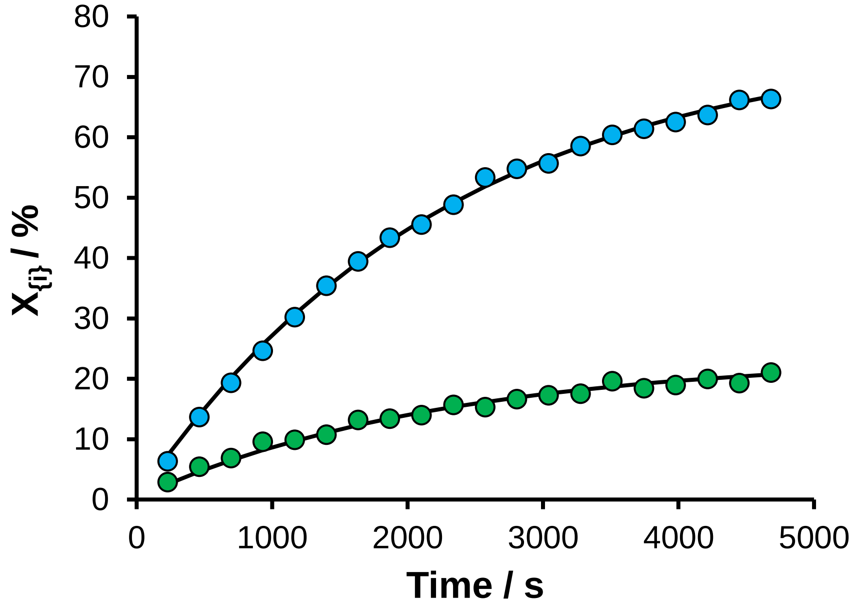

Figure S39: Reaction profiles, expressed in terms of mole fractions (X{i}), for the aminolysis and concurrent hydrolysis of **MepA-L-PheF** (**E**$_m$) in D_2O under the title conditions (10 °C; *I* = 2.0 M, KCl), as measured by in situ $^{19}F\{^1H\}$ NMR spectroscopy. The amide **P**$_{Am}$ is the major product; the hydrolysis product, **P**$_{aa}$, is the minor product. Fit to kinetic model 1 shown. ^{L}**S** = L-serinamide.

pH*(10°C) = 8.506 ([^{L}**S**]$_{T}$ = 1200 mM)

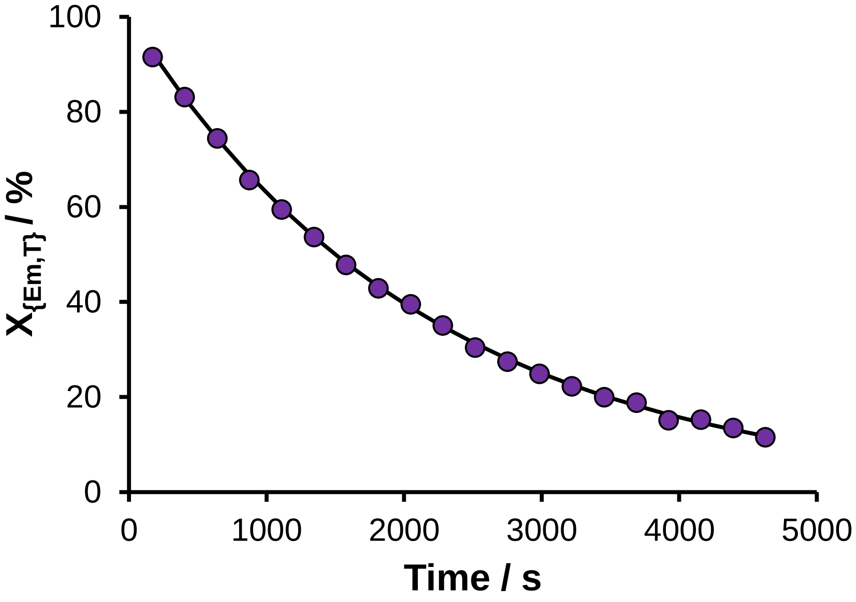

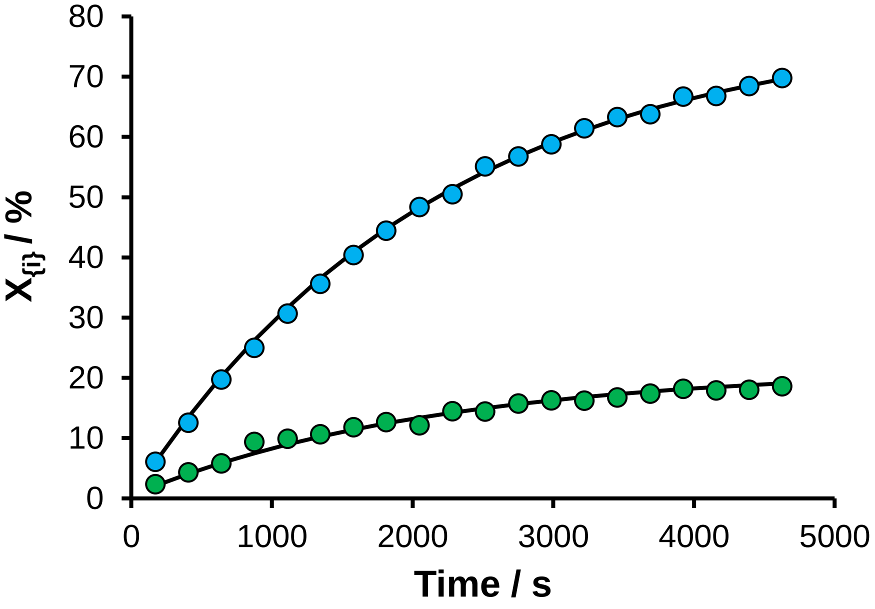

Figure S40: Reaction profiles, expressed in terms of mole fractions (X{i}), for the aminolysis and concurrent hydrolysis of **MepA-L-PheF** (**E$_m$**) in D_2O under the title conditions (10 °C; *I* = 2.0 M, KCl), as measured by in situ $^{19}F\{^{1}H\}$ NMR spectroscopy. The amide **P$_{Am}$** is the major product; the hydrolysis product, **P$_{aa}$**, is the minor product. Fit to kinetic model 1 shown. ^{L}**S** = L-serinamide.

pH*(10°C) = 8.082 ([ᴸ**S**]$_T$ = 1200 mM)

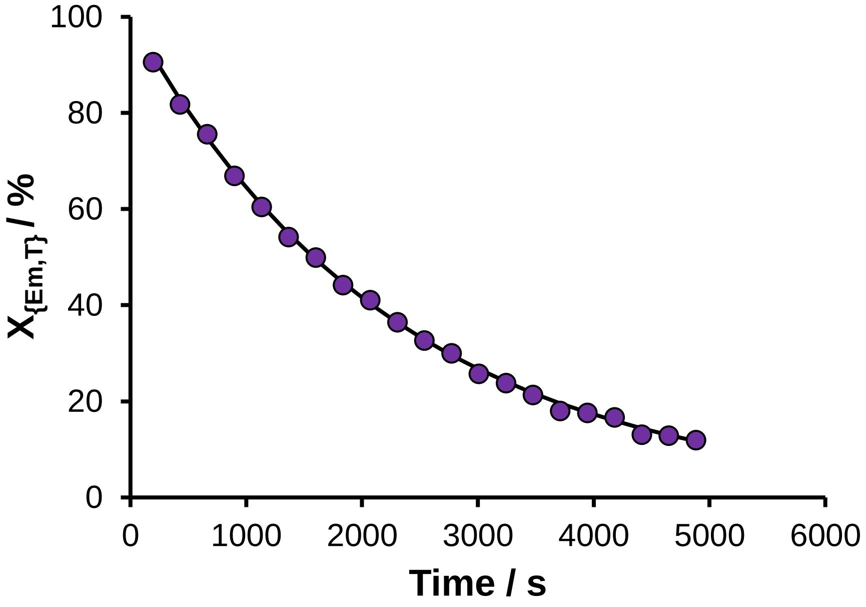

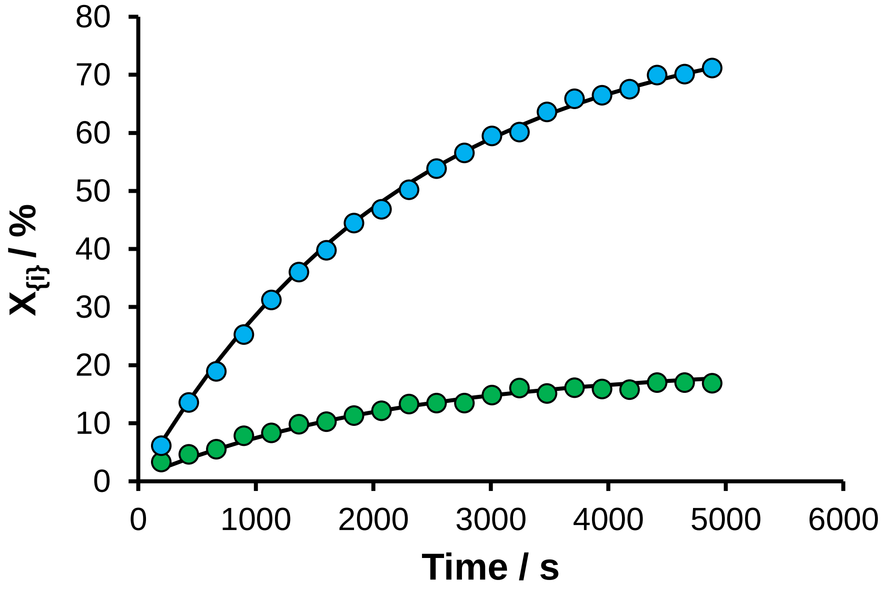

Figure S41: Reaction profiles, expressed in terms of mole fractions (X{i}), for the aminolysis and concurrent hydrolysis of **MepA-L-PheF** (**E**$_m$) in D_2O under the title conditions (10 °C; *I* = 2.0 M, KCl), as measured by in situ $^{19}F\{^1H\}$ NMR spectroscopy. The amide **P**$_{Am}$ is the major product; the hydrolysis product, **P**$_{aa}$, is the minor product. Fit to kinetic model 1 shown. ᴸ**S** = L-serinamide.

pH*(10°C) = 7.801 ([^{L}**S**]$_T$ = 1200 mM)

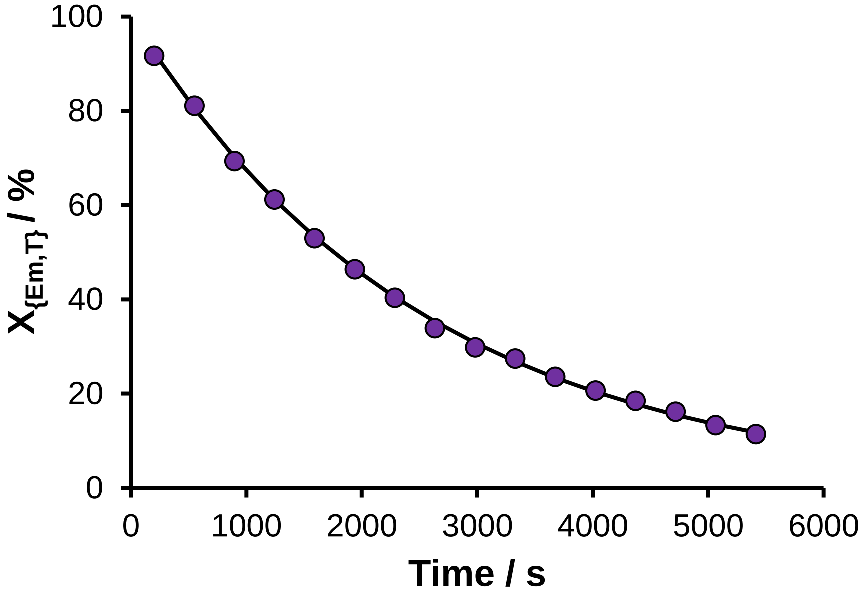

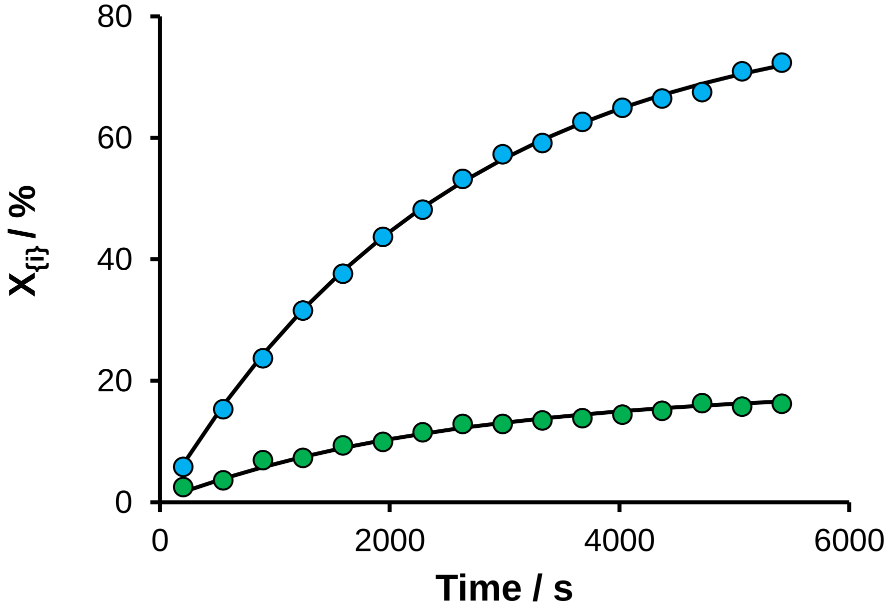

Figure S42: Reaction profiles, expressed in terms of mole fractions (X{i}), for the aminolysis and concurrent hydrolysis of **MepA-L-PheF** (**E$_m$**) in D_2O under the title conditions (10 °C; *I* = 2.0 M, KCl), as measured by in situ $^{19}F\{^{1}H\}$ NMR spectroscopy. The amide **P$_{Am}$** is the major product; the hydrolysis product, **P$_{aa}$,** is the minor product. Fit to kinetic model 1 shown. ^{L}**S** = L-serinamide.

pH*(10°C) = 7.290 ([^{L}**S**]$_T$ = 1200 mM)

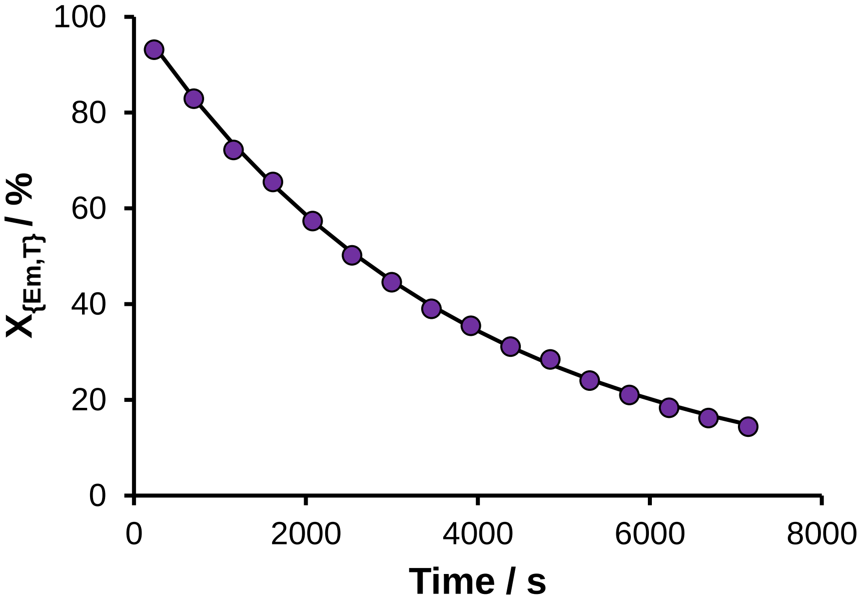

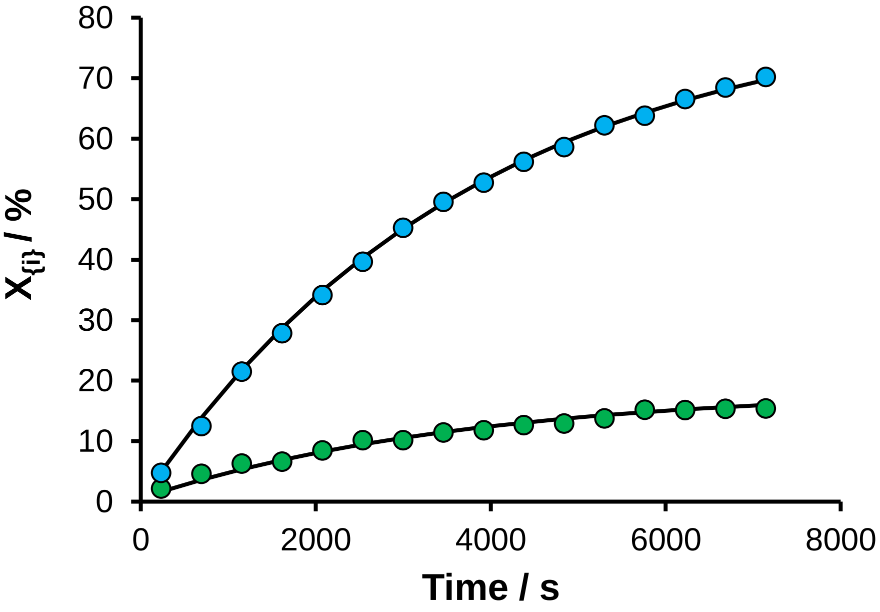

Figure S43: Reaction profiles, expressed in terms of mole fractions (X{i}), for the aminolysis and concurrent hydrolysis of **MepA-L-PheF** (**E$_m$**) in D_2O under the title conditions (10 °C; *I* = 2.0 M, KCl), as measured by in situ $^{19}F\{^{1}H\}$ NMR spectroscopy. The amide **P$_{Am}$** is the major product; the hydrolysis product, **P$_{aa}$**, is the minor product. Fit to kinetic model 1 shown. ^{L}**S** = L-serinamide.

pH*(10°C) = 6.895 ([^{L}**S**]$_T$ = 1200 mM)

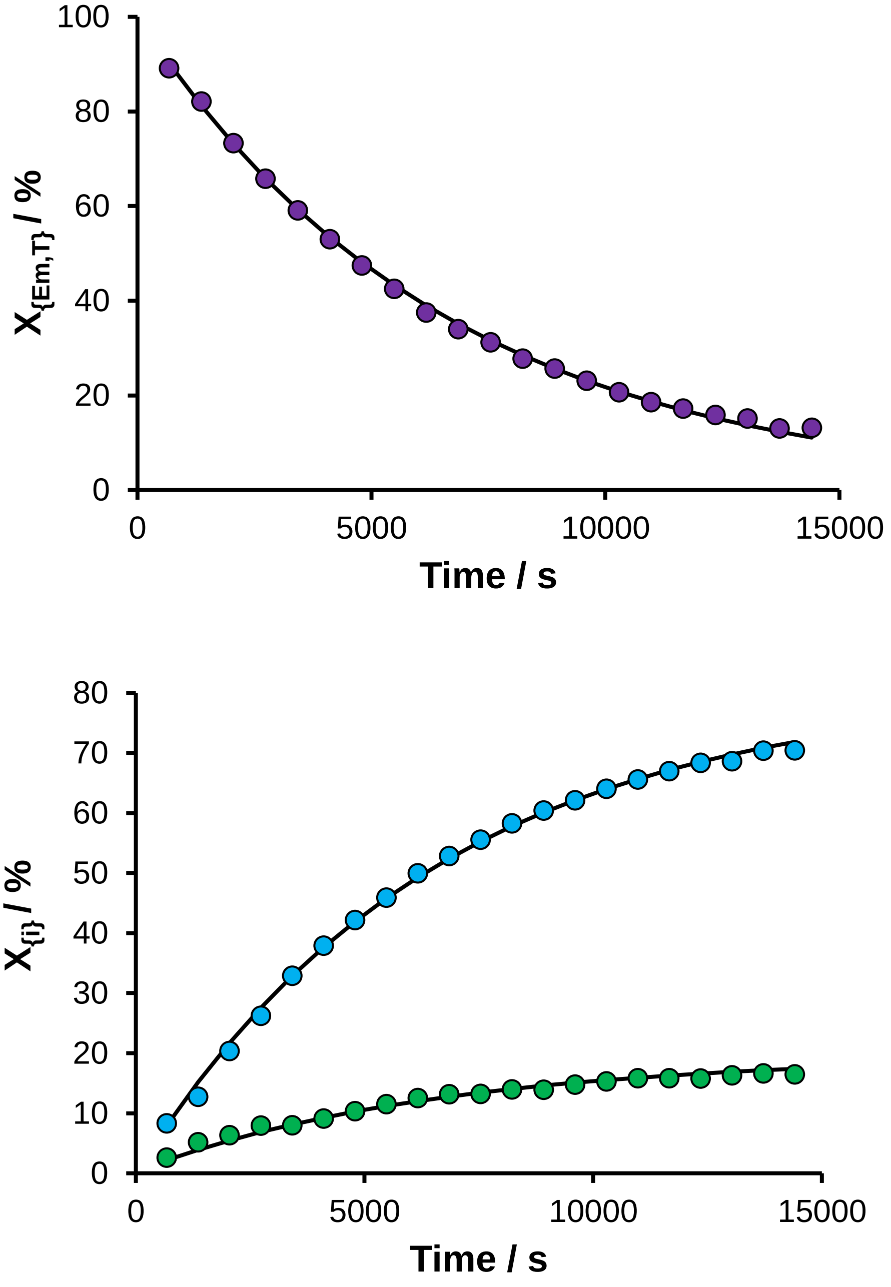

Figure S44: Reaction profiles, expressed in terms of mole fractions (X{i}), for the aminolysis and concurrent hydrolysis of **MepA-L-PheF** (**E**$_m$) in D_2O under the title conditions (10 °C; *I* = 2.0 M, KCl), as measured by in situ $^{19}F\{^{1}H\}$ NMR spectroscopy. The amide **P**$_{Am}$ is the major product; the hydrolysis product, **P**$_{aa}$, is the minor product. Fit to kinetic model 1 shown. ^{L}**S** = L-serinamide.

pH*(10°C) = 6.377 ([^{L}**S**]$_T$ = 1200 mM)

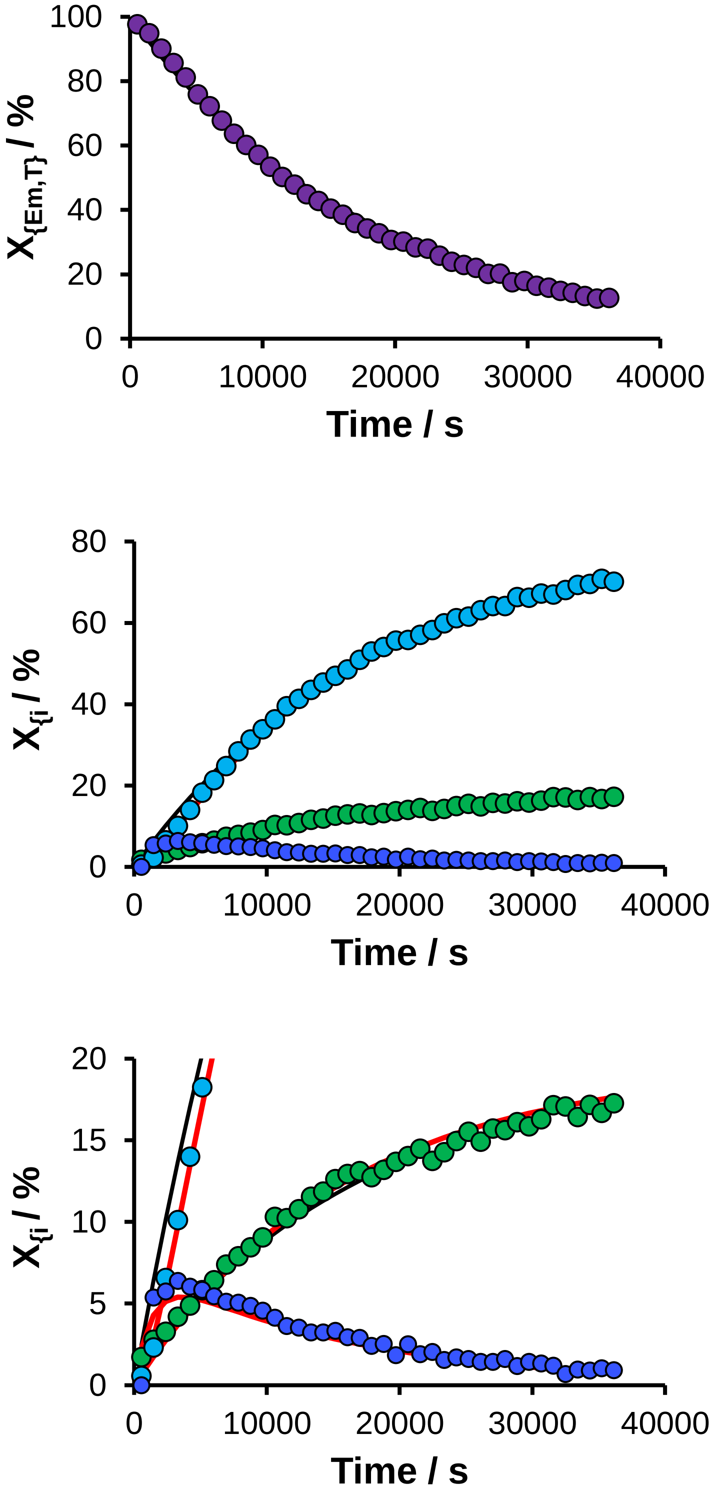

Figure S45: Reaction profiles, expressed in terms of mole fractions (X{i}), for the aminolysis and concurrent hydrolysis of **MepA-L-PheF** (**E$_m$**) in D_2O under the title conditions (10 °C; I = 2.0 M, KCl), as measured by in situ ^{19}F{^{1}H} NMR spectroscopy. The amide **P$_{Am}$** is the major product; the hydrolysis product, **P$_{aa}$**, is the minor product. The intermediate is assigned as **I$_{Es}$**. Fit to kinetic model 2 shown. ^{L}**S** = L-serinamide.

MepA-L-PheF ($\mathbf{E_m}$) + L-serinamide ($^{\mathrm{L}}\mathbf{S}$); D_2O, 30 °C

Summary of pseudo first-order rate constants

pH*(30°C)	$[^{\mathrm{L}}S]_T$ / mM	$k^{\Psi}_{Am} \times 10^4$ / s^{-1}	$k'_{Am} \times 10^4$ / (M^{-1} s^{-1})	$k^{\Psi}_{Hyd} \times 10^4$ / s^{-1}
8.951	1200	9.64	8.04	6.90
8.513	1200	9.74	8.12	5.11
8.179	1200	10.68	8.90	4.82
7.826	1200	11.33	9.44	4.30
7.522	1200	11.14	9.28	3.96
7.157	1200	9.85	8.21	3.25
6.69	1200	6.69	5.58	2.26
6.353	1200	3.91	3.26	1.35
5.691	1200	1.35	1.13	0.50

Table S11: Summary of raw data from the pH*-k'_{Am} and pH*-k^{Ψ}_{Hyd} profiles for the aminolysis/hydrolysis of MepA-L-PheF ($\mathbf{E_m}$) with L-serinamide (D_2O, 30 °C, I = 2.0 M, KCl).

Raw reaction profiles

pH*(30°C) = 8.951 ([^{L}**S**]$_T$ = 1200 mM)

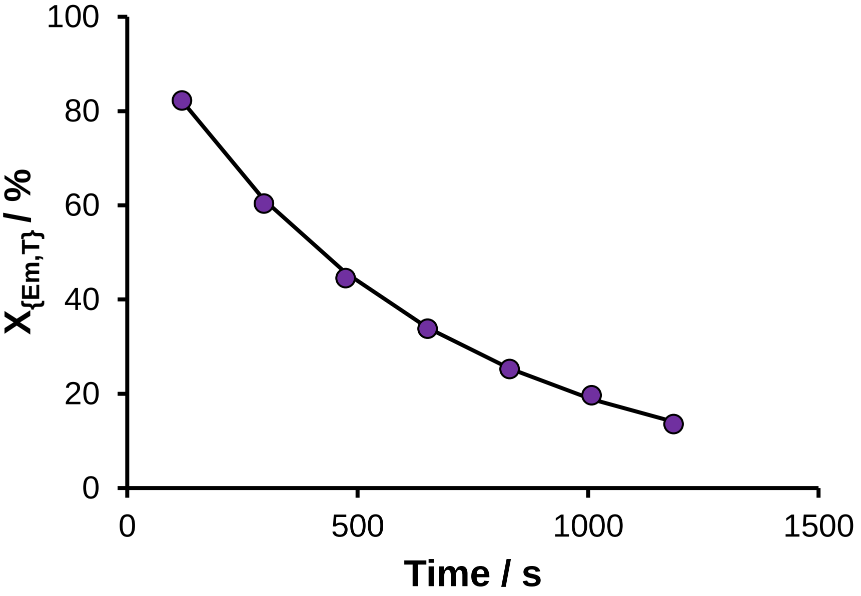

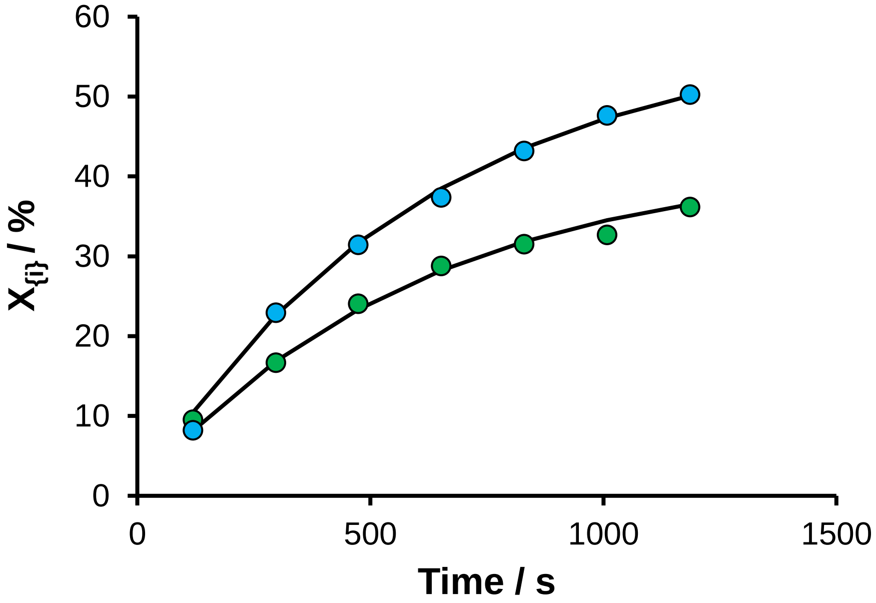

Figure S46: Reaction profiles, expressed in terms of mole fractions (X{i}), for the aminolysis and concurrent hydrolysis of **MepA-L-PheF** (**E**$_m$) in D_2O under the title conditions (30 °C; *I* = 2.0 M, KCl), as measured by in situ $^{19}F\{^1H\}$ NMR spectroscopy. The amide **P**$_{Am}$ is the major product; the hydrolysis product, **P**$_{aa}$, is the minor product. Fit to kinetic model 1 shown. ^{L}**S** = L-serinamide.

pH*(30°C) = 8.513 ([^{L}**S**]$_T$ = 1200 mM)

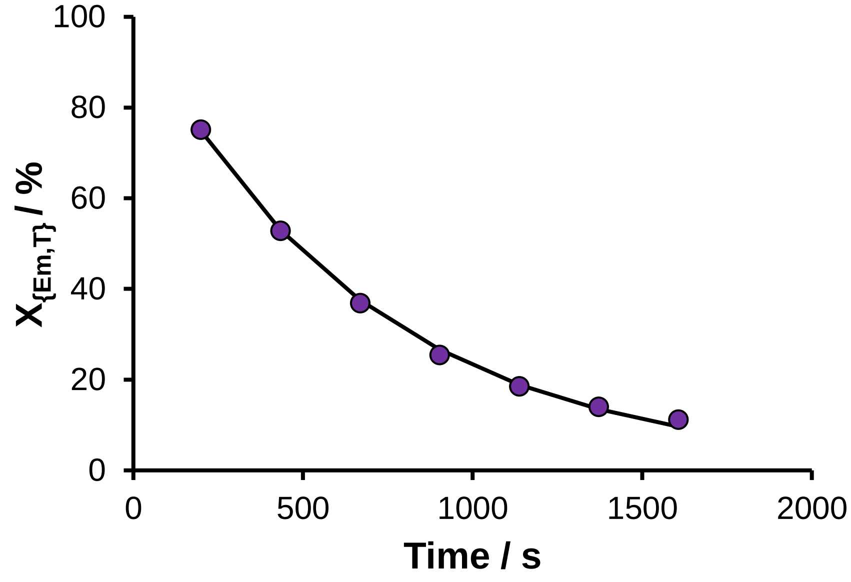

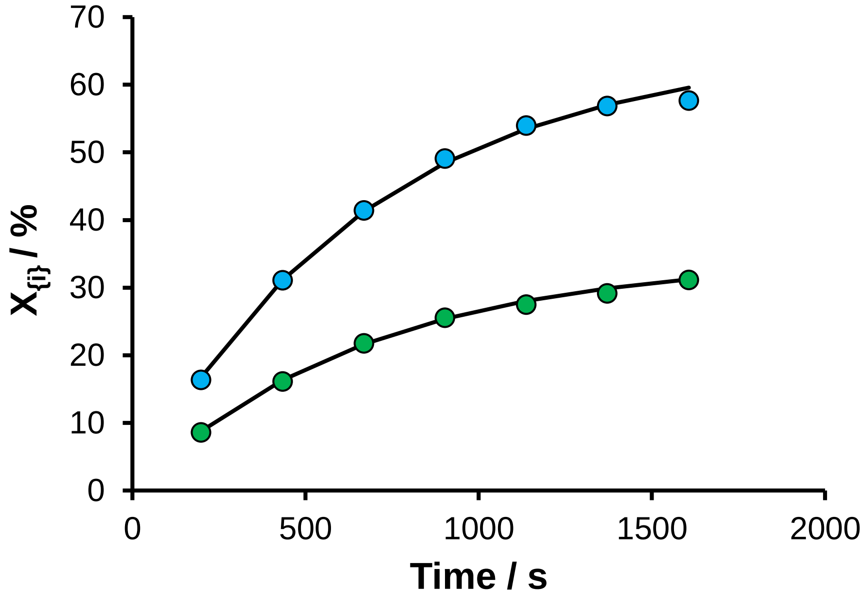

Figure S47: Reaction profiles, expressed in terms of mole fractions (X{i}), for the aminolysis and concurrent hydrolysis of **MepA-L-PheF** (**E**$_m$) in D_2O under the title conditions (30 °C; *I* = 2.0 M, KCl), as measured by in situ $^{19}F\{^1H\}$ NMR spectroscopy. The amide **P**$_{Am}$ is the major product; the hydrolysis product, **P**$_{aa}$, is the minor product. Fit to kinetic model 1 shown. ^{L}**S** = L-serinamide.

pH*(30°C) = 8.179 ([^{L}**S**]$_T$ = 1200 mM)

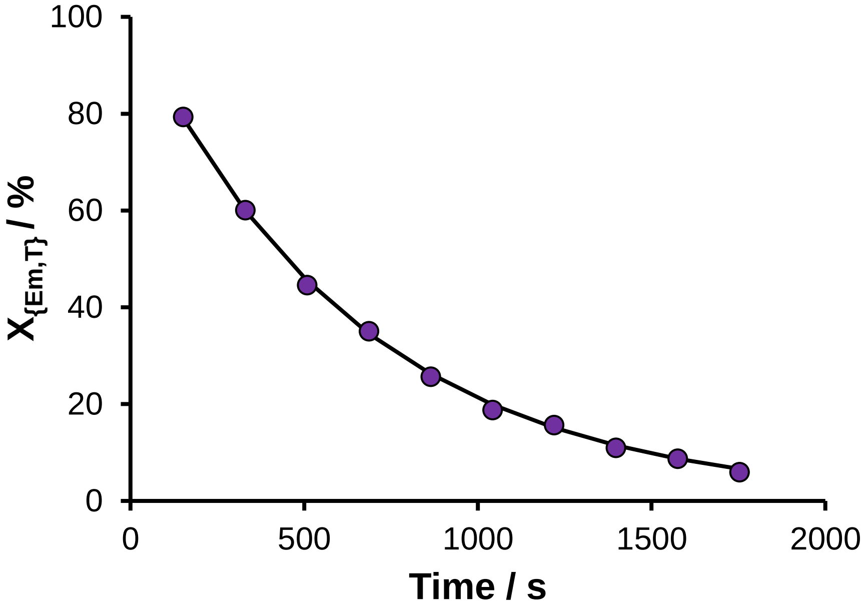

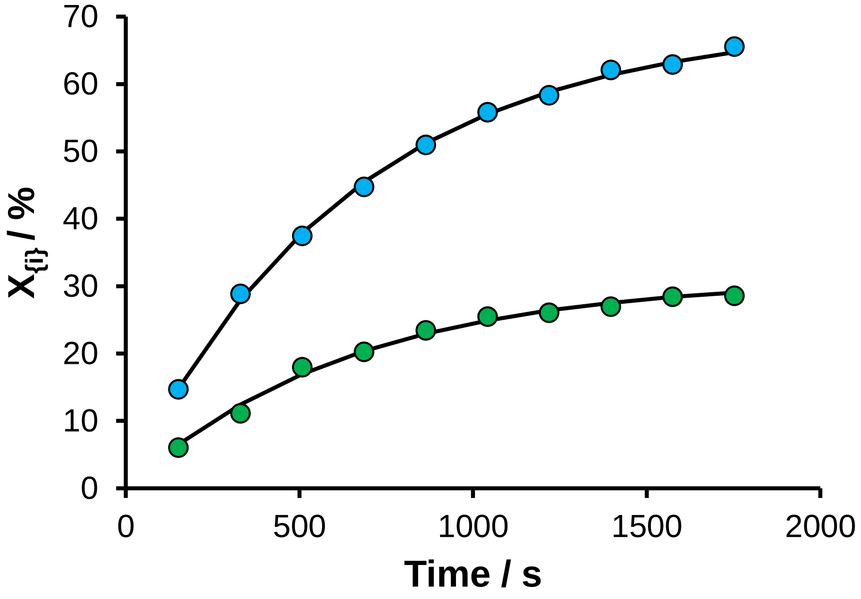

Figure S48: Reaction profiles, expressed in terms of mole fractions (X{i}), for the aminolysis and concurrent hydrolysis of **MepA-L-PheF** (**E$_m$**) in D_2O under the title conditions (30 °C; *I* = 2.0 M, KCl), as measured by in situ ^{19}F{^{1}H} NMR spectroscopy. The amide **P$_{Am}$** is the major product; the hydrolysis product, **P$_{aa}$,** is the minor product. Fit to kinetic model 1 shown. ^{L}**S** = L-serinamide.

pH*(30°C) = 7.826 ([^{L}**S**]$_T$ = 1200 mM)

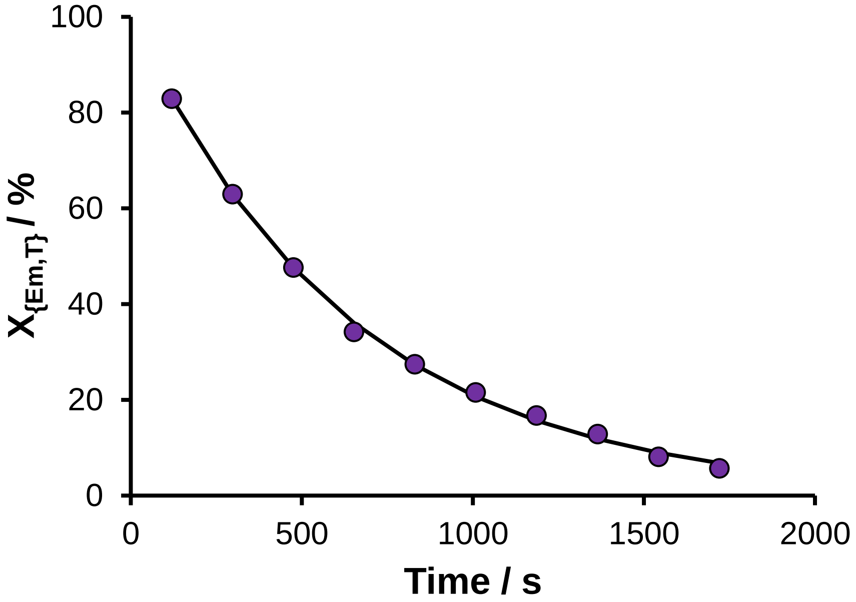

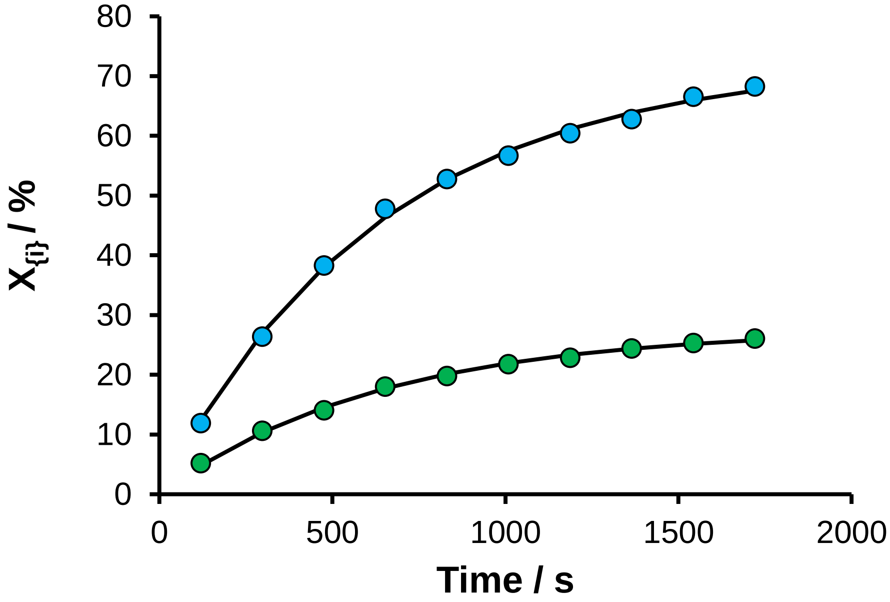

Figure S49: Reaction profiles, expressed in terms of mole fractions (X{i}), for the aminolysis and concurrent hydrolysis of **MepA-L-PheF** (**E$_m$**) in D_2O under the title conditions (30 °C; *I* = 2.0 M, KCl), as measured by in situ $^{19}F\{^{1}H\}$ NMR spectroscopy. The amide **P$_{Am}$** is the major product; the hydrolysis product, **P$_{aa}$**, is the minor product. Fit to kinetic model 1 shown. **^{L}S** = L-serinamide.

pH*(30°C) = 7.522 ([^{L}**S**]$_T$ = 1200 mM)

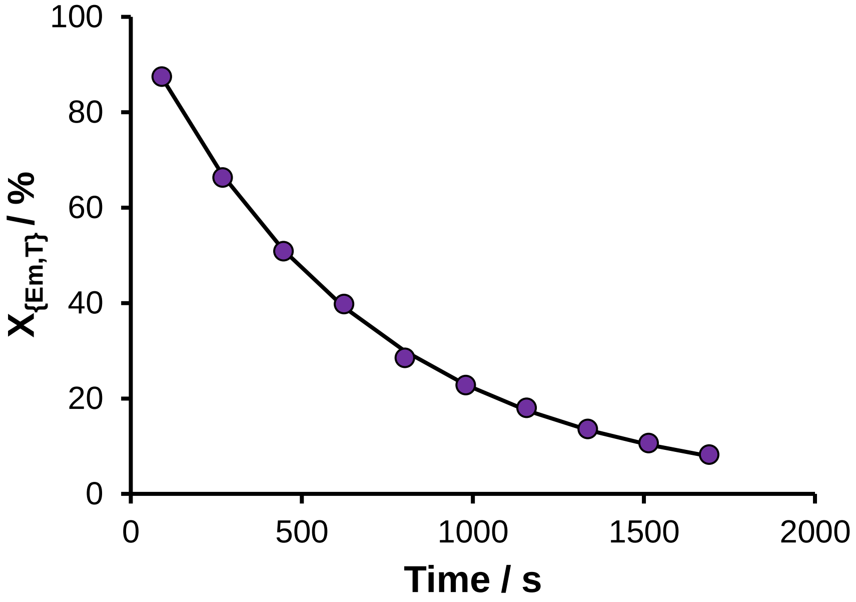

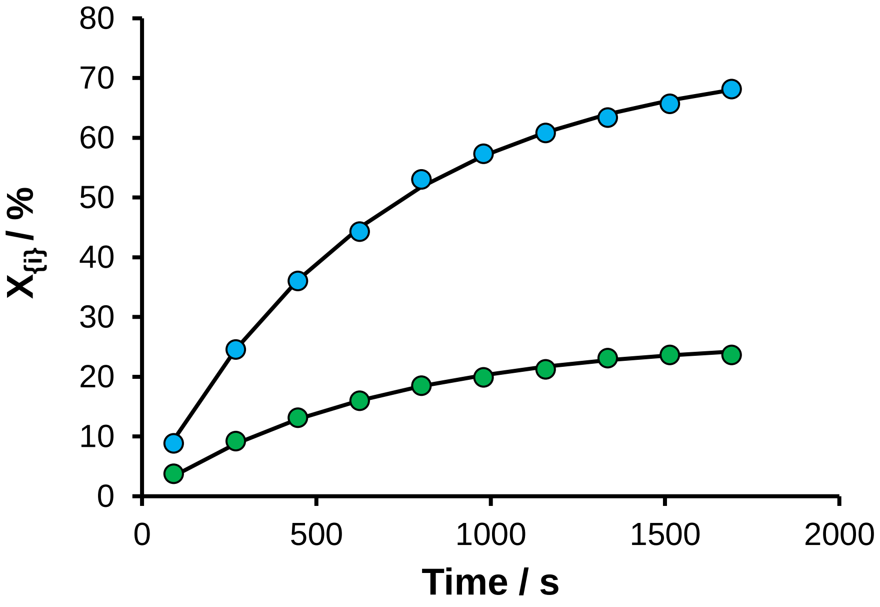

Figure S50: Reaction profiles, expressed in terms of mole fractions (X{i}), for the aminolysis and concurrent hydrolysis of **MepA-L-PheF** (**E**$_m$) in D_2O under the title conditions (30 °C; *I* = 2.0 M, KCl), as measured by in situ $^{19}F\{^{1}H\}$ NMR spectroscopy. The amide **P**$_{Am}$ is the major product; the hydrolysis product, **P**$_{aa}$, is the minor product. Fit to kinetic model 1 shown. ^{L}**S** = L-serinamide.

pH*(30°C) = 7.157 ([^{L}**S**]$_T$ = 1200 mM)

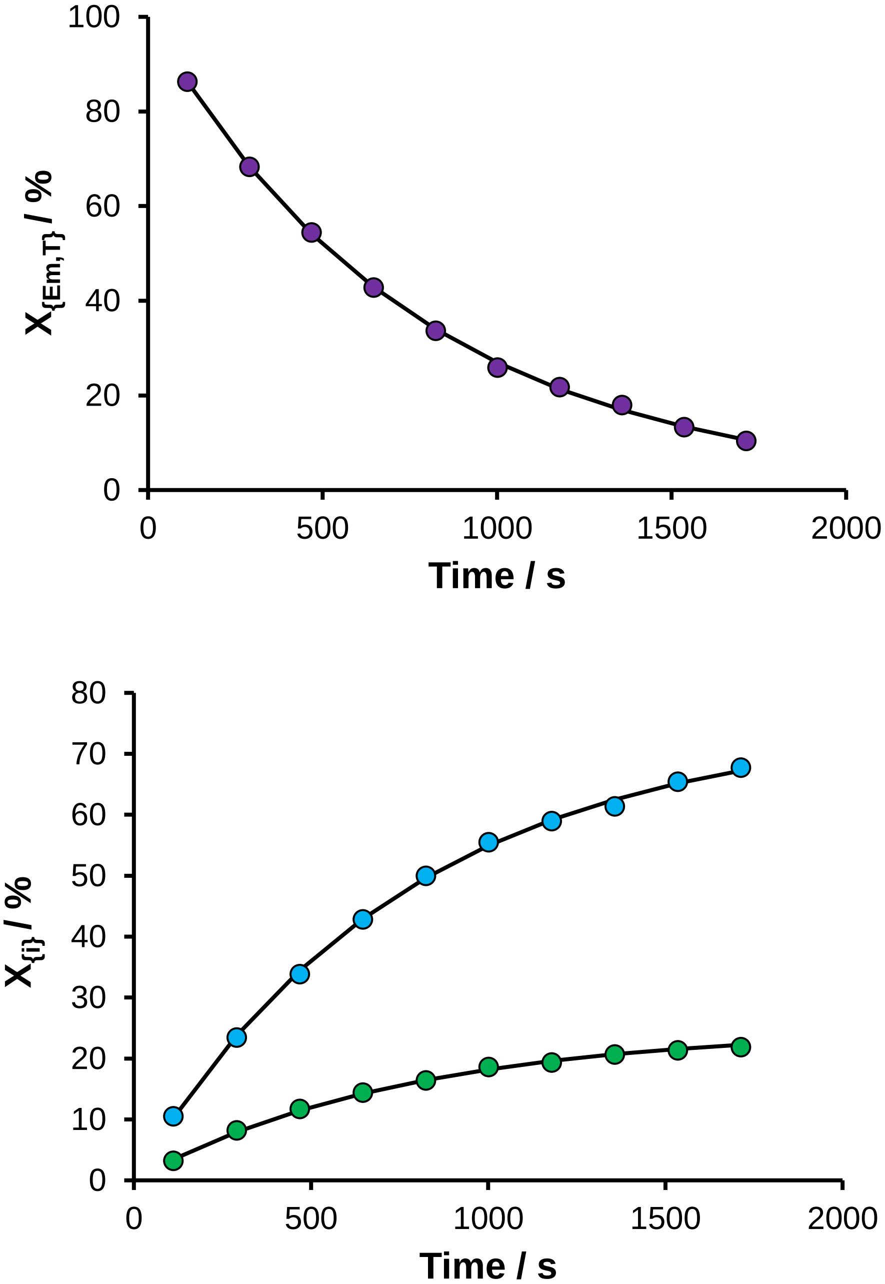

Figure S51: Reaction profiles, expressed in terms of mole fractions (X{i}), for the aminolysis and concurrent hydrolysis of **MepA-L-PheF** (**E**$_m$) in D_2O under the title conditions (30 °C; *I* = 2.0 M, KCl), as measured by in situ $^{19}F\{^1H\}$ NMR spectroscopy. The amide **P**$_{Am}$ is the major product; the hydrolysis product, **P**$_{aa}$, is the minor product. Fit to kinetic model 1 shown. ^{L}**S** = L-serinamide.

pH*(30°C) = 6.690 ([^{L}**S**]$_T$ = 1200 mM)

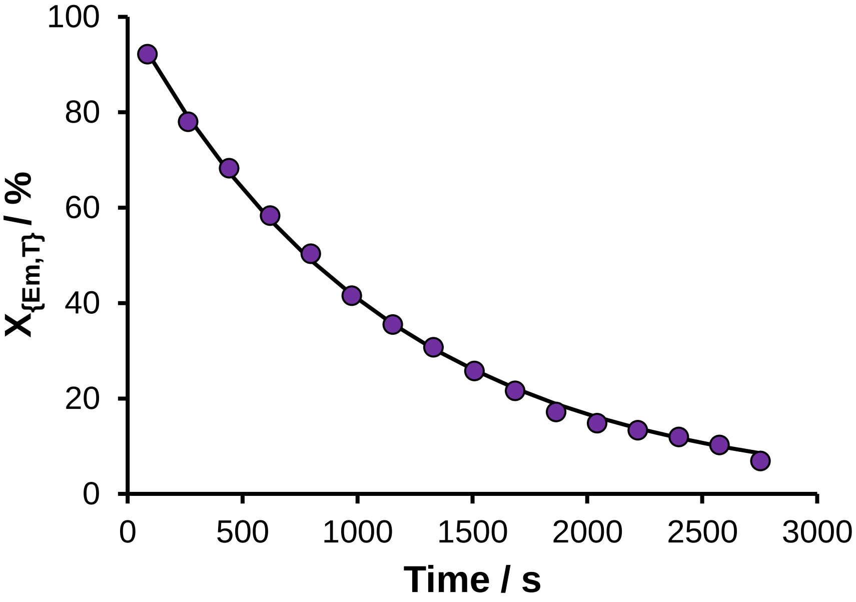

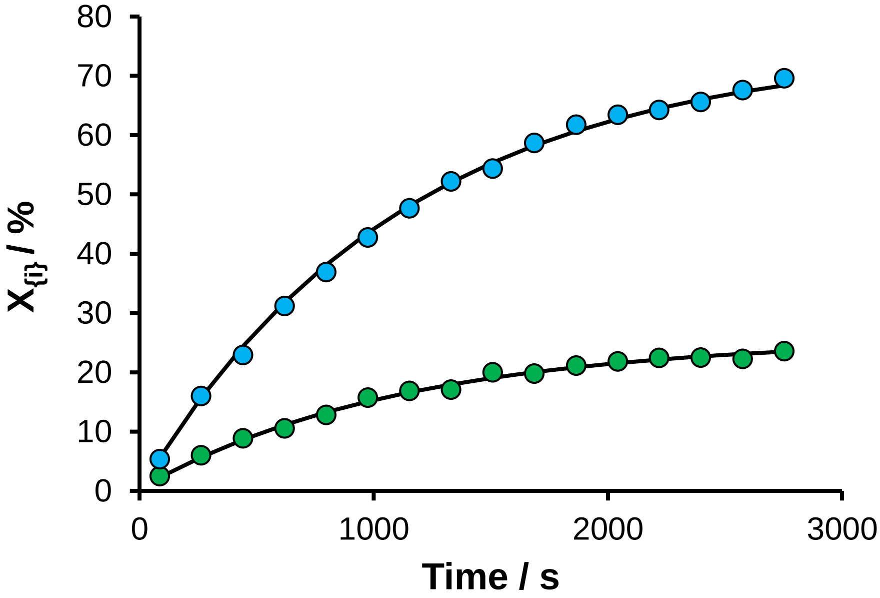

Figure S52: Reaction profiles, expressed in terms of mole fractions (X{i}), for the aminolysis and concurrent hydrolysis of **MepA-L-PheF** (**E**$_m$) in D_2O under the title conditions (30 °C; *I* = 2.0 M, KCl), as measured by in situ $^{19}F\{^1H\}$ NMR spectroscopy. The amide **P**$_{Am}$ is the major product; the hydrolysis product, **P**$_{aa}$, is the minor product. Fit to kinetic model 1 shown. ^{L}**S** = L-serinamide.

pH*(30°C) = 6.353 ([^{L}**S**]$_T$ = 1200 mM)

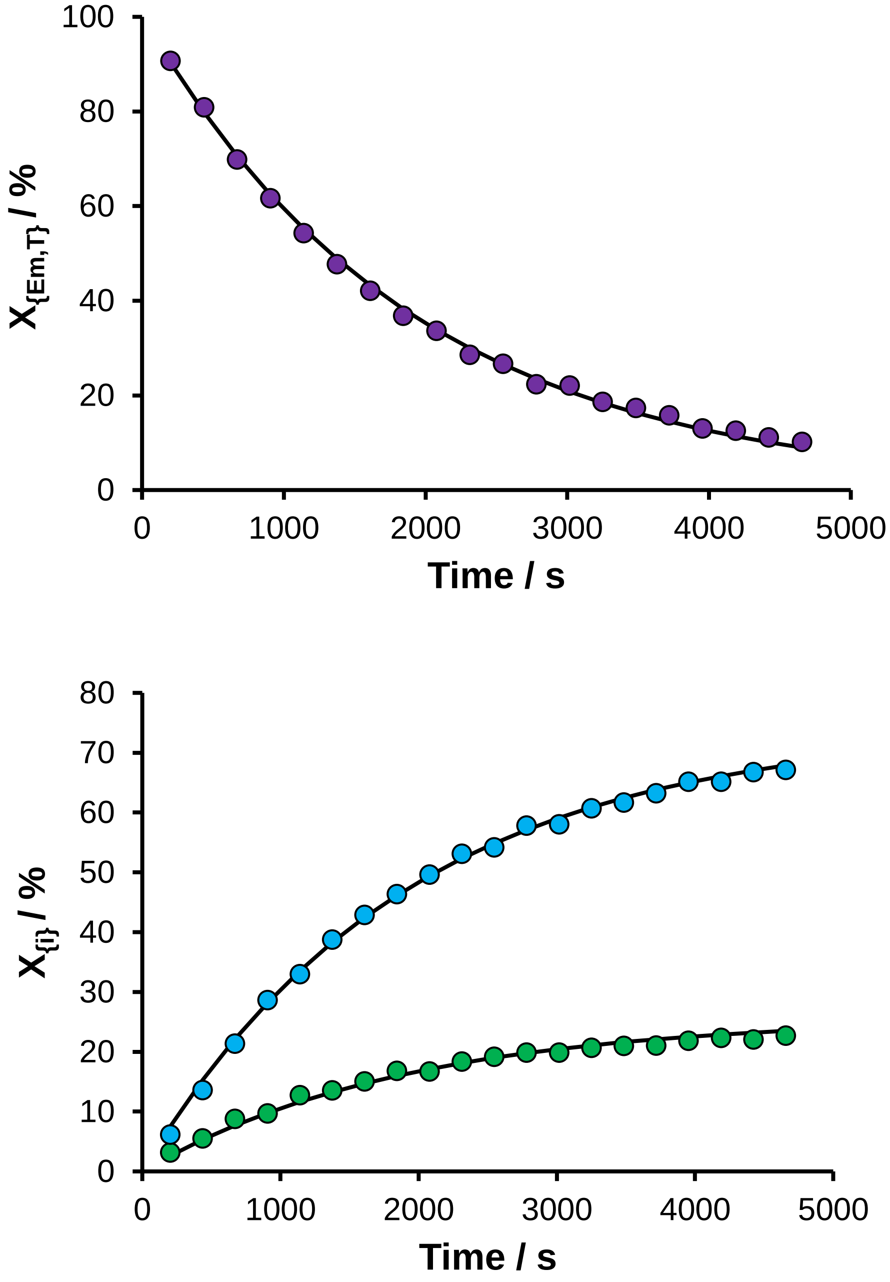

Figure S53: Reaction profiles, expressed in terms of mole fractions (X{i}), for the aminolysis and concurrent hydrolysis of **MepA-L-PheF** (**E**$_m$) in D_2O under the title conditions (30 °C; *I* = 2.0 M, KCl), as measured by in situ $^{19}F\{^1H\}$ NMR spectroscopy. The amide **P**$_{Am}$ is the major product; the hydrolysis product, **P**$_{aa}$, is the minor product. Fit to kinetic model 1 shown. ^{L}**S** = L-serinamide.

pH*(30°C) = 5.691 ($[^{L}\mathbf{S}]_T$ = 1200 mM)

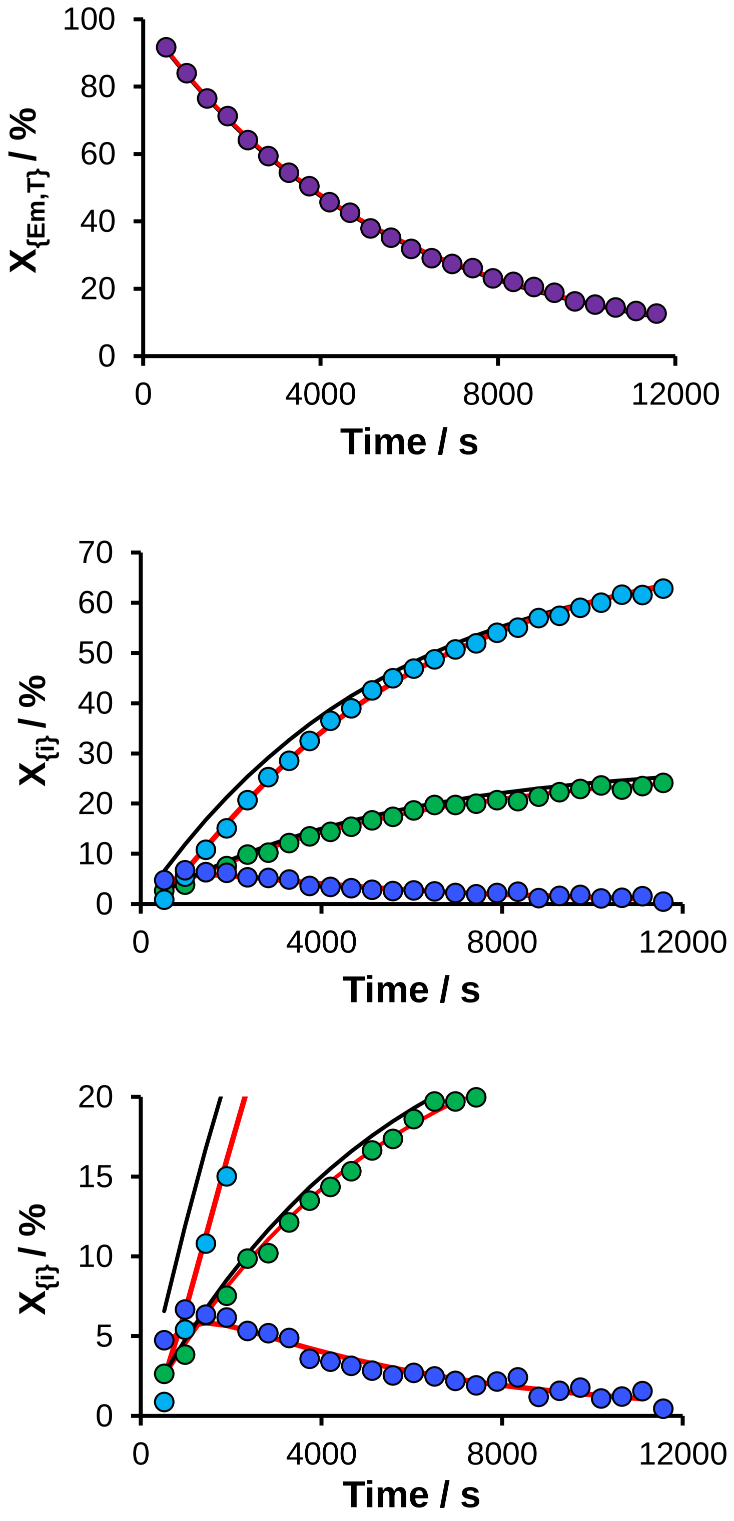

Figure S54: Reaction profiles, expressed in terms of mole fractions (X{i}), for the aminolysis and concurrent hydrolysis of **MepA-L-PheF** (**E_m**) in D_2O under the title conditions (30 °C; *I* = 2.0 M, KCl), as measured by in situ $^{19}F\{^{1}H\}$ NMR spectroscopy. The amide **P_{Am}** is the major product; the hydrolysis product, **P_{aa}**, is the minor product. The intermediate is assigned as **I_{Es}**. Fit to kinetic model 2 shown. **^{L}S** = L-serinamide.

MepA-N-f-PheF (**E_m^f**) + L-serinamide (**^LS**); H_2O/D_2O, 20 °C

Summary of rate constants

	**D_2O**			**H_2O**
	***T* / °C**			***T* / °C**
	20.0	30.0	40.0	20.0
**$pK_a^*(^LS^+; T)$**	7.94	7.69	7.44	7.72
**$pK_w^*(T)$**	14.64	14.29	13.97	14.16
**$pH^*(T)$**	7.807	7.529	7.260	7.603
**$[^LS]_T$ / mM**	1200	1200	1200	1200
**$[^LS]$ / mM**	510	490	480	520
**$k^{\Psi}_{Am} \times 10^4$ / s^{-1}**	1.69	2.86	4.35	2.10
**$k'_{Am} \times 10^4$ / (M^{-1} s^{-1})**	1.40	2.39	3.63	1.75
**$k_{Am,f} \times 10^4$ / (M^{-1} s^{-1})**	3.31	5.84	9.13	4.05
**$k^{\Psi}_{Hyd} \times 10^4$ / s^{-1}**	0.49	1.04	1.91	0.75
**$k_{OH^-,f}$ / (M^{-1} s^{-1})**	231	415	683	191

Table S12: Summary of k'^{Ψ}_{Am}, k^{Ψ}_{Am} and k'^{Ψ}_{Hyd} for the aminolysis/hydrolysis of MepA-N-f-L-PheF (**E_m^f**) with L-serinamide (H_2O/D_2O, 20 – 40 °C, I = 2.0 M, KCl), alongside corresponding second-order rate-constants ($k_{Am,f}$; $k_{OH^-,f}$). Value for pK_w^* calculated from literature data as described in the text.

Raw reaction profiles

pH*(20°C) = 7.807 ([^{L}**S**]$_T$ = 1200 mM) ; D_2O

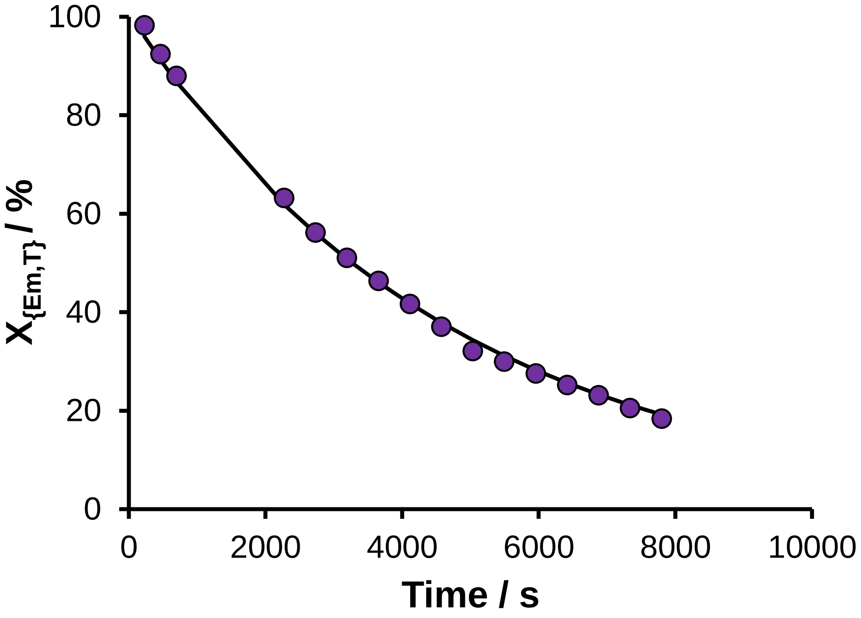

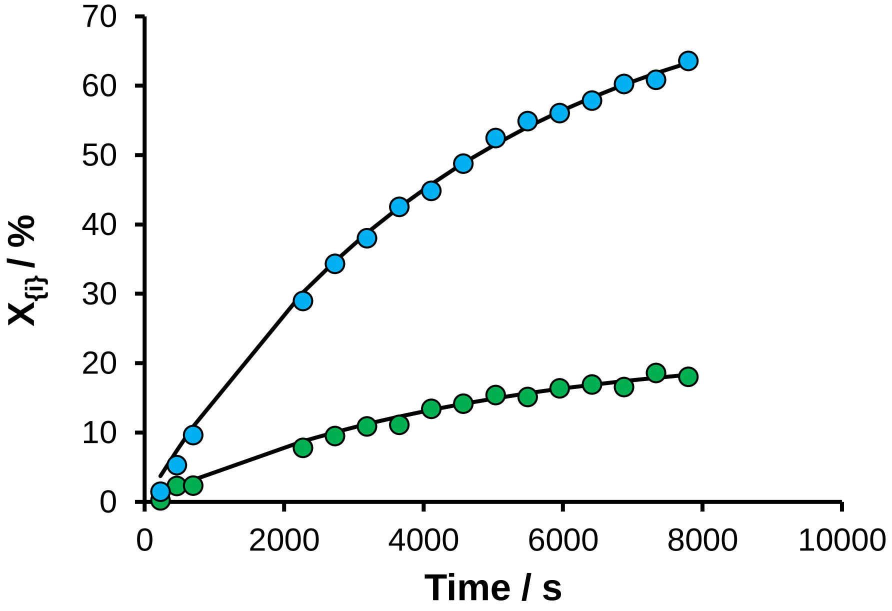

Figure S55: Reaction profiles, expressed in terms of mole fractions (X{i}), for the aminolysis and concurrent hydrolysis of **MepA-N-f-PheF** (**E**$_{\mathbf{m}}^{\mathbf{f}}$) in D_2O under the title conditions (20 °C; *I* = 2.0 M, KCl), as measured by in situ $^{19}F\{^{1}H\}$ NMR spectroscopy. The amide **P**$_{\mathbf{Am}}^{\mathbf{f}}$ is the major product; the hydrolysis product, **P**$_{\mathbf{aa}}^{\mathbf{f}}$, is the minor product. Fit to kinetic model 1 shown. ^{L}**S** = L-serinamide.

pH*(30°C) = 7.529 ([^{L}**S**]$_T$ = 1200 mM); D_2O

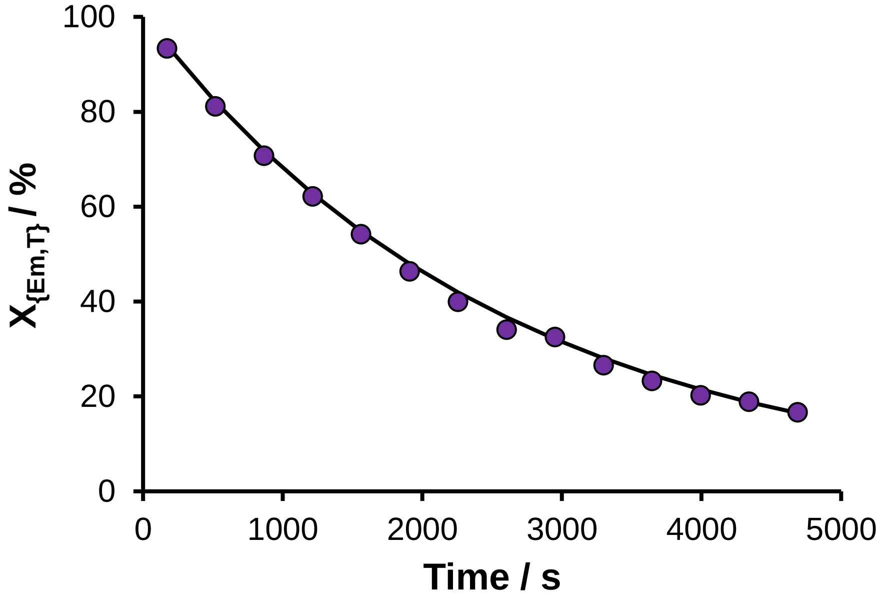

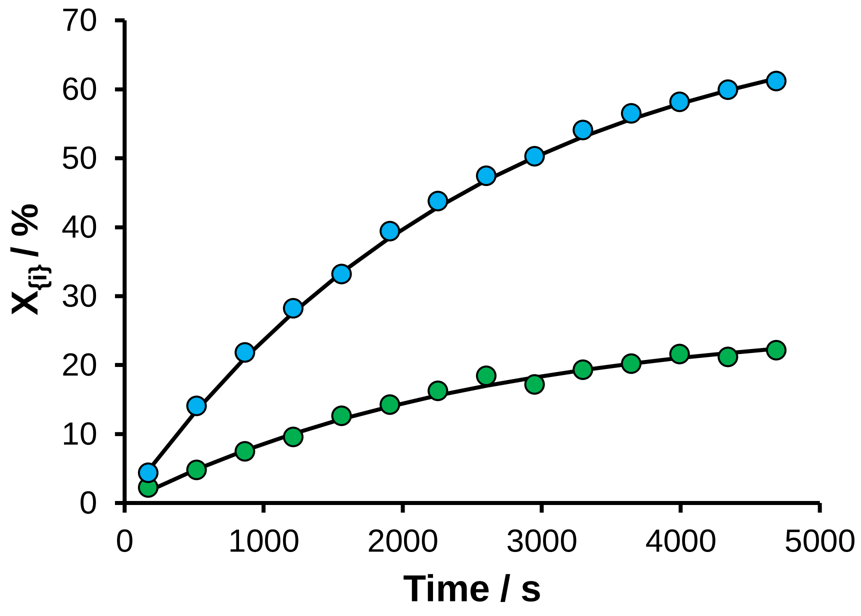

Figure S56: Reaction profiles, expressed in terms of mole fractions (X{i}), for the aminolysis and concurrent hydrolysis of **MepA-N-f-PheF** ($\mathbf{E_m^f}$) in D_2O under the title conditions (30 °C; I = 2.0 M, KCl), as measured by in situ ^{19}F{^{1}H} NMR spectroscopy. The amide $\mathbf{P_{Am}^f}$ is the major product; the hydrolysis product, $\mathbf{P_{aa}^f}$, is the minor product. Fit to kinetic model 1 shown. ^{L}**S** = L-serinamide.

pH*(40°C) = 7.260 ([^{L}**S**]$_T$ = 1200 mM); D_2O

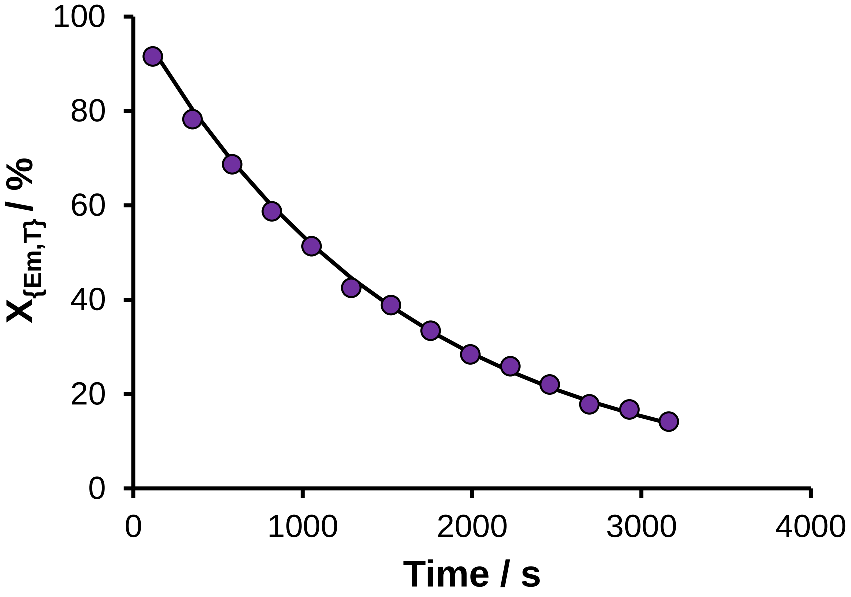

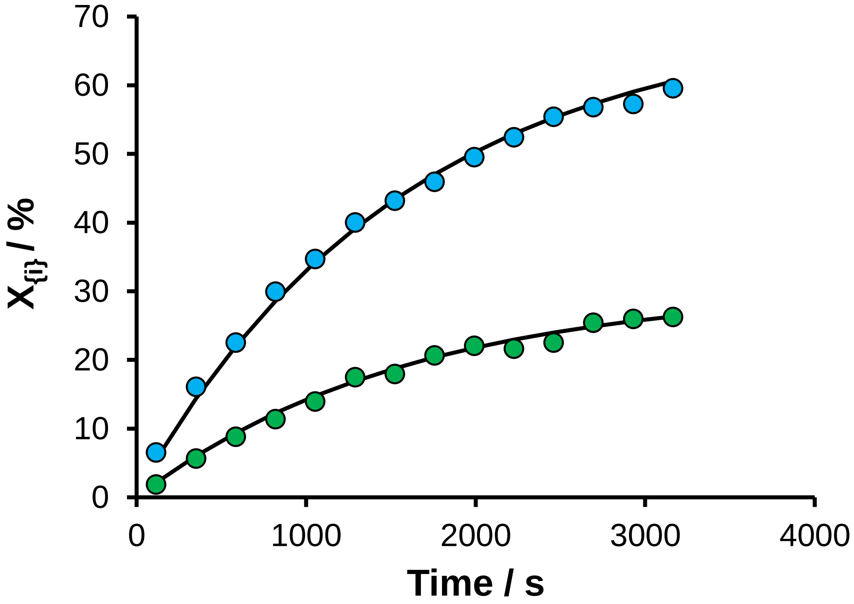

Figure S57: Reaction profiles, expressed in terms of mole fractions (X{i}), for the aminolysis and concurrent hydrolysis of **MepA-N-f-PheF** (**E$_m$^{f}**) in D_2O under the title conditions (40 °C; *I* = 2.0 M, KCl), as measured by in situ $^{19}F\{^{1}H\}$ NMR spectroscopy. The amide **P$_{Am}$^{f}** is the major product; the hydrolysis product, **P$_{aa}$^{f}**, is the minor product. Fit to kinetic model 1 shown. ^{L}**S** = L-serinamide.

pH*(20°C) = 7.603 ([^{L}**S**]$_T$ = 1200 mM); H_2O

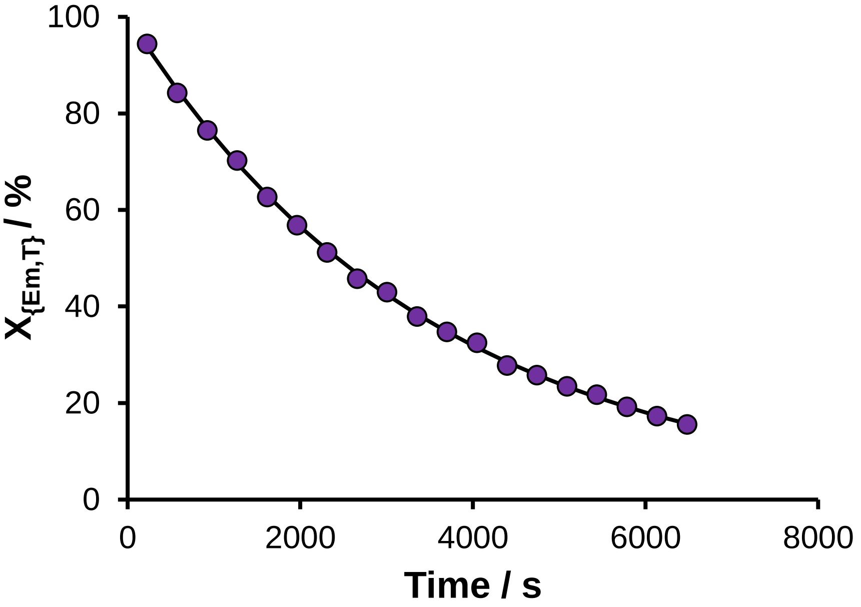

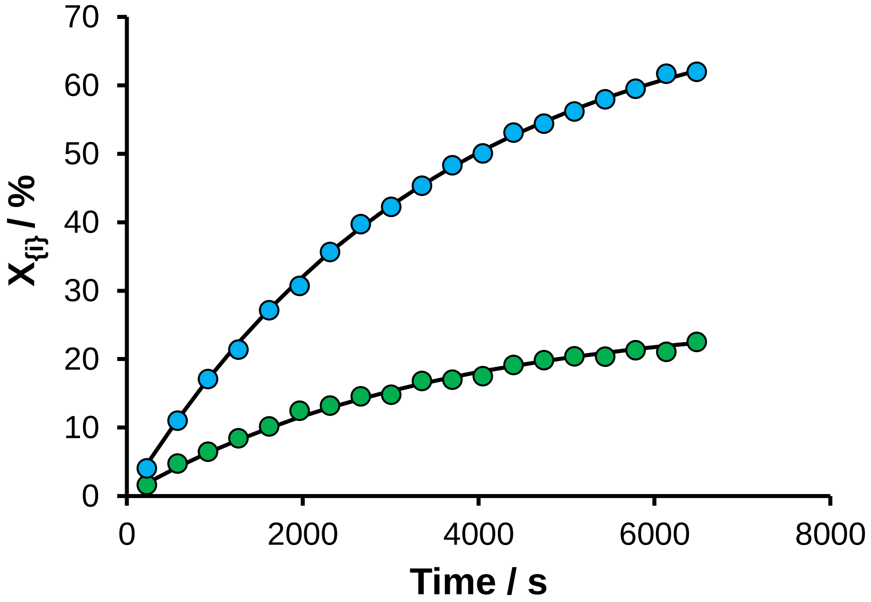

Figure S58: Reaction profiles, expressed in terms of mole fractions (X{i}), for the aminolysis and concurrent hydrolysis of **MepA-N-f-PheF** (**E$_m$^{f}**) in H_2O under the title conditions (20 °C; *I* = 2.0 M, KCl), as measured by in situ $^{19}F\{^{1}H\}$ NMR spectroscopy. The amide **P$_{Am}$^{f}** is the major product; the hydrolysis product, **P$_{aa}$^{f}**, is the minor product. Fit to kinetic model 1 shown. ^{L}**S** = L-serinamide.

MepA-(PheF)$_2$ (**E$_{Bis}$**) + L-serinamide (**^{L}S**); D_2O, 20 °C

Summary of pseudo first-order rate constants

pH*(20°C)	[^{L}S]$_T$ / mM	$k_{\Psi Am,Bis} \times 10^4$ / s^{-1}	$k'_{Am,Bis} \times 10^4$ / (M^{-1} s^{-1})	$k_{\Psi Hyd,Bis} \times 10^4$ / s^{-1}
8.623	600	2.28	3.80	0.79
8.357	600	2.55	4.25	0.96
8.016	600	3.18	5.29	0.91
7.781	600	3.51	5.85	1.02
7.469	600	3.83	6.38	1.16
6.965	600	3.07	5.12	1.12
6.482	600	2.14	3.57	0.83
6.006	1200	2.63	2.19	0.58
5.613	2000	2.66	1.33	0.53

pH*(20°C)	[^{L}S]$_T$ / mM	$k_{\Psi Am} \times 10^4$ / s^{-1}	$k'_{Am} \times 10^4$ / (M^{-1} s^{-1})	$k_{\Psi Hyd} \times 10^4$ / s^{-1}
8.623	600	3.59	5.98	2.17
8.357	600	3.98	6.63	1.93
8.016	600	3.49	5.81	1.88
7.781	600	3.64	6.06	1.37
7.469	600	2.95	4.92	1.20
6.965	600	1.89	3.15	0.79
6.482	600	0.86	1.43	0.42
6.006	1200	0.84	0.70	0.25
5.613	2000	0.81	0.40	0.16

Table S13: Summary of raw data from the pH*-k'_{Am}, pH*-$k'_{Am,Bis}$, pH*-$k_{\Psi Hyd}$ and pH*-$k_{\Psi Hyd,Bis}$ profiles for the aminolysis/hydrolysis of MepA-(L-PheF)$_2$ (**E$_{bis}$**) with L-serinamide (D_2O, 20 °C, I = 2.0 M, KCl).

Raw reaction profiles

pH*(20°C) = 8.623 ([^{L}**S**]$_T$ = 600 mM) ; D_2O

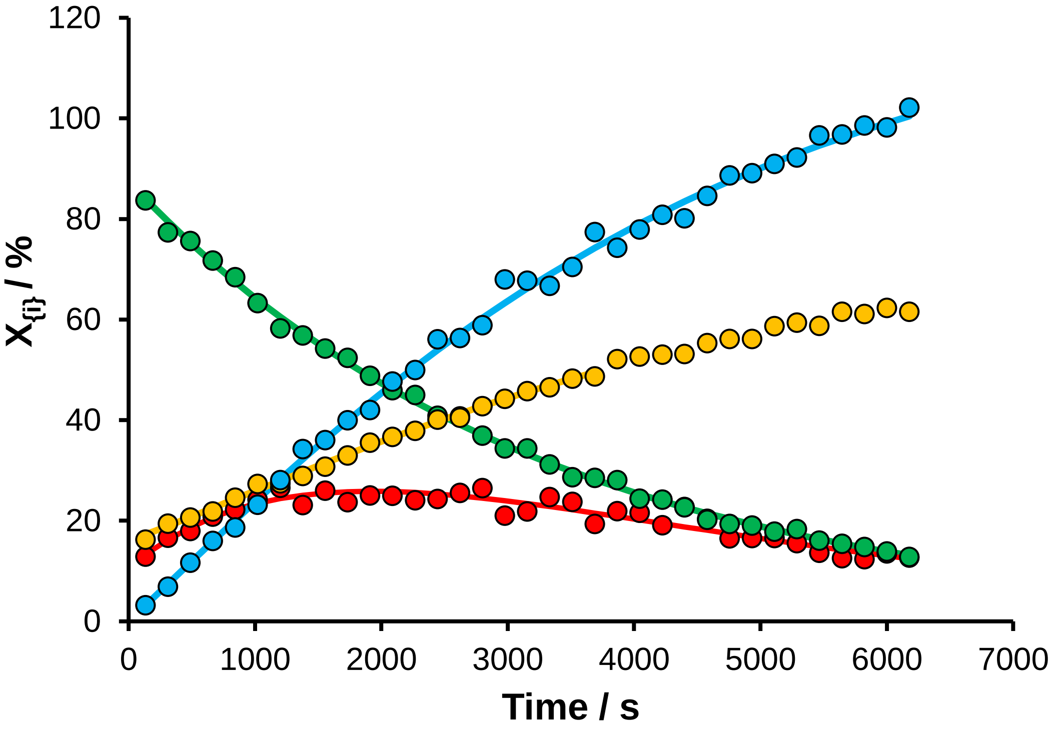

Figure S59: Reaction profiles, expressed in terms of mole fractions (X{i}), for the aminolysis and concurrent hydrolysis of **MepA-(PheF)**$_2$ (**E**$_{Bis}$) in D_2O under the title conditions (20 °C; *I* = 2.0 M, KCl), as measured by in situ $^{19}F\{^1H\}$ NMR spectroscopy. The amide **P**$_{Am}$ is the major product; the hydrolysis product, **P**$_{aa}$, is the minor product. The intermediate is **E**$_{m,T}$. Non-zero y-intercepts of **X**$_{\{Paa\}}$ and **X**$_{\{Em,T\}}$ are the result of pre-reaction hydrolysis in the stock solution of **E**$_{Bis}$. Initial mole fraction at the first time point i.e., (**X**$_{\{Bis\}}$ + **X**$_{\{Paa\}}$ + **X**$_{\{Em,T\}}$)$_{t=t1}$, normalised arbitrarily to 100%. Fit to kinetic model 3 shown. ^{L}**S** = L-serinamide.

pH*(20°C) = 8.357 ([^{L}**S**]$_T$ = 600 mM) ; D_2O

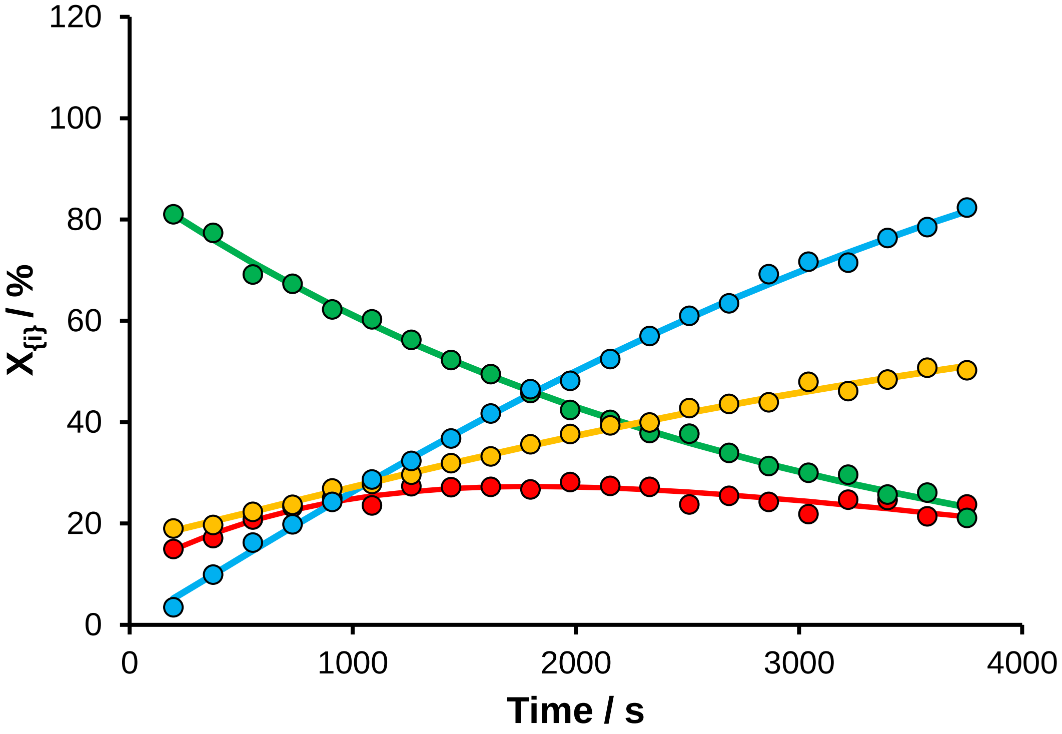

Figure S60: Reaction profiles, expressed in terms of mole fractions (X{i}), for the aminolysis and concurrent hydrolysis of **MepA-(PheF)$_2$** (**E$_{Bis}$**) in D_2O under the title conditions (20 °C; *I* = 2.0 M, KCl), as measured by in situ ^{19}F{^{1}H} NMR spectroscopy. The amide **P$_{Am}$** is the major product; the hydrolysis product, **P$_{aa}$**, is the minor product. The intermediate is **E$_{m,T}$**. Non-zero y-intercepts of **X$_{\{Paa\}}$** and **X$_{\{Em,T\}}$** are the result of pre-reaction hydrolysis in the stock solution of **E$_{Bis}$**. Initial mole fraction at the first time point i.e., (**X$_{\{Bis\}}$ + X$_{\{Paa\}}$ + X$_{\{Em,T\}}$**)$_{t=t1}$, normalised arbitrarily to 100%. Fit to kinetic model 3 shown. ^{L}**S** = L-serinamide.

pH*(20°C) = 8.016 ([^{L}**S**]$_T$ = 600 mM) ; D_2O

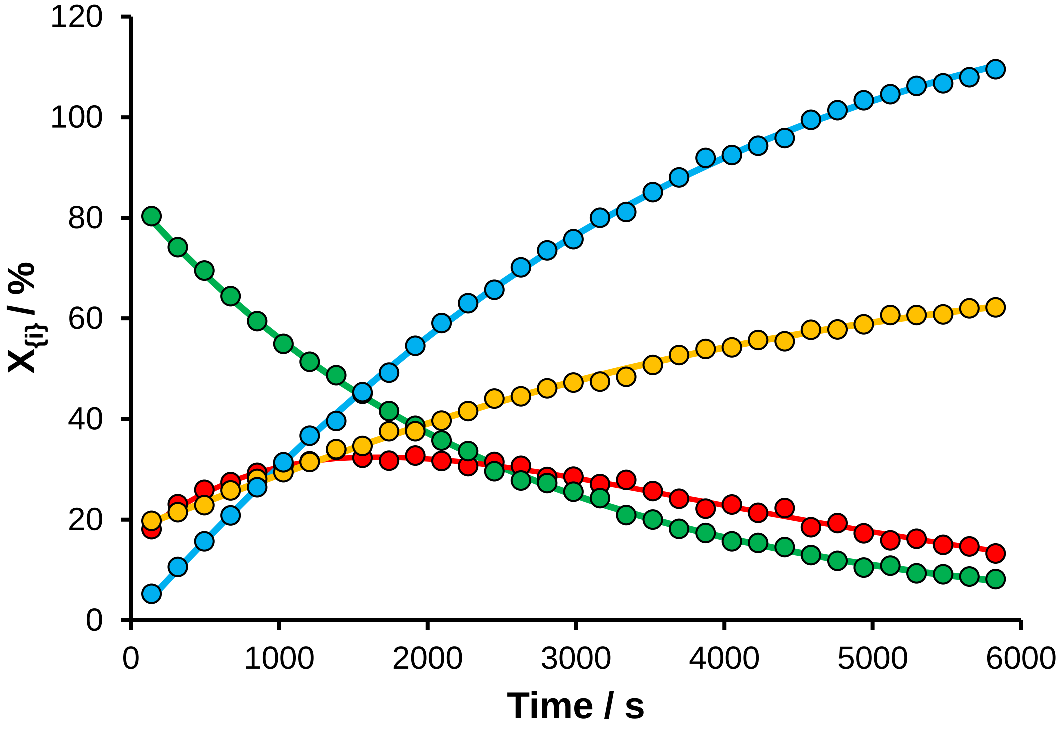

Figure S61: Reaction profiles, expressed in terms of mole fractions (X{i}), for the aminolysis and concurrent hydrolysis of **MepA-(PheF)₂** (**E$_{Bis}$**) in D_2O under the title conditions (20 °C; *I* = 2.0 M, KCl), as measured by in situ ^{19}F{^{1}H} NMR spectroscopy. The amide **P$_{Am}$** is the major product; the hydrolysis product, **P$_{aa}$**, is the minor product. The intermediate is **E$_{m,T}$**. Non-zero y-intercepts of **X$_{\{Paa\}}$** and **X$_{\{Em,T\}}$** are the result of pre-reaction hydrolysis in the stock solution of **E$_{Bis}$**. Initial mole fraction at the first time point i.e., (**X$_{\{Bis\}}$** + **X$_{\{Paa\}}$** + **X$_{\{Em,T\}}$**)$_{t=t1}$, normalised arbitrarily to 100%. Fit to kinetic model 3 shown. ^{L}**S** = L-serinamide.

pH*(20°C) = 7.781 ([^{L}**S**]$_T$ = 600 mM) ; D_2O

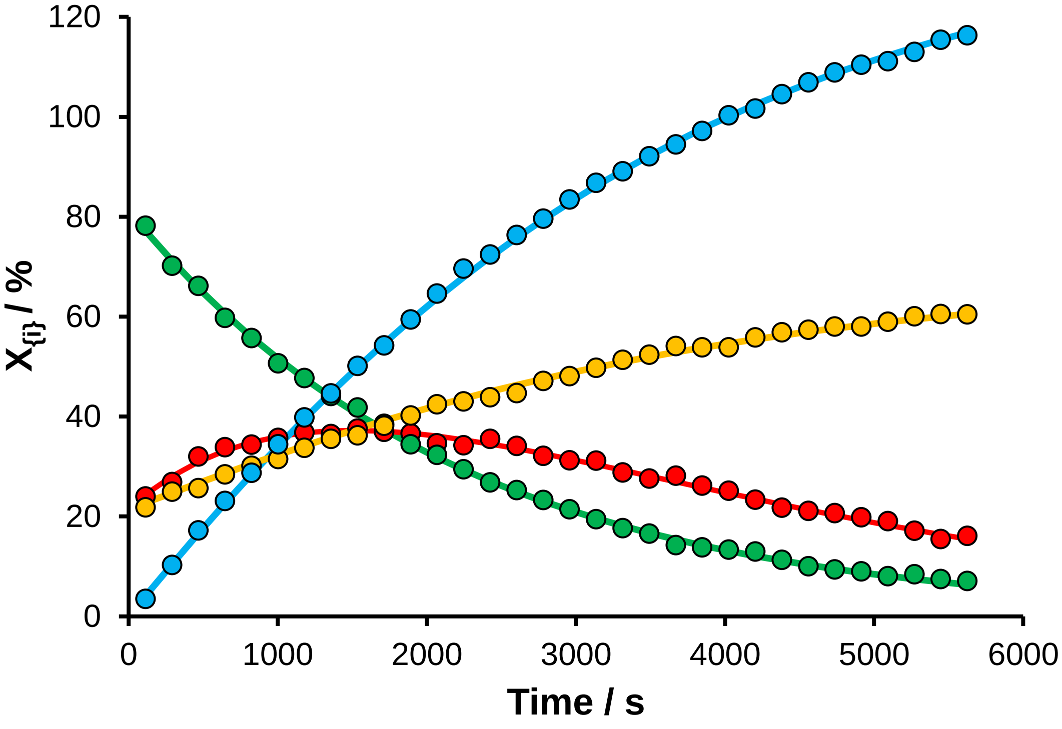

Figure S62: Reaction profiles, expressed in terms of mole fractions (X{i}), for the aminolysis and concurrent hydrolysis of **MepA-(PheF)₂** (**E**$_{Bis}$) in D_2O under the title conditions (20 °C; *I* = 2.0 M, KCl), as measured by in situ $^{19}F\{^1H\}$ NMR spectroscopy. The amide **P**$_{Am}$ is the major product; the hydrolysis product, **P**$_{aa}$, is the minor product. The intermediate is **E**$_{m,T}$. Non-zero y-intercepts of **X**$_{\{Paa\}}$ and **X**$_{\{Em,T\}}$ are the result of pre-reaction hydrolysis in the stock solution of **E**$_{Bis}$. Initial mole fraction at the first time point i.e., (**X**$_{\{Bis\}}$ + **X**$_{\{Paa\}}$ + **X**$_{\{Em,T\}}$)$_{t=t1}$, normalised arbitrarily to 100%. Fit to kinetic model 3 shown. ^{L}**S** = L-serinamide.

pH*(20°C) = 7.469 ([ᴸ**S**]$_T$ = 600 mM) ; D_2O

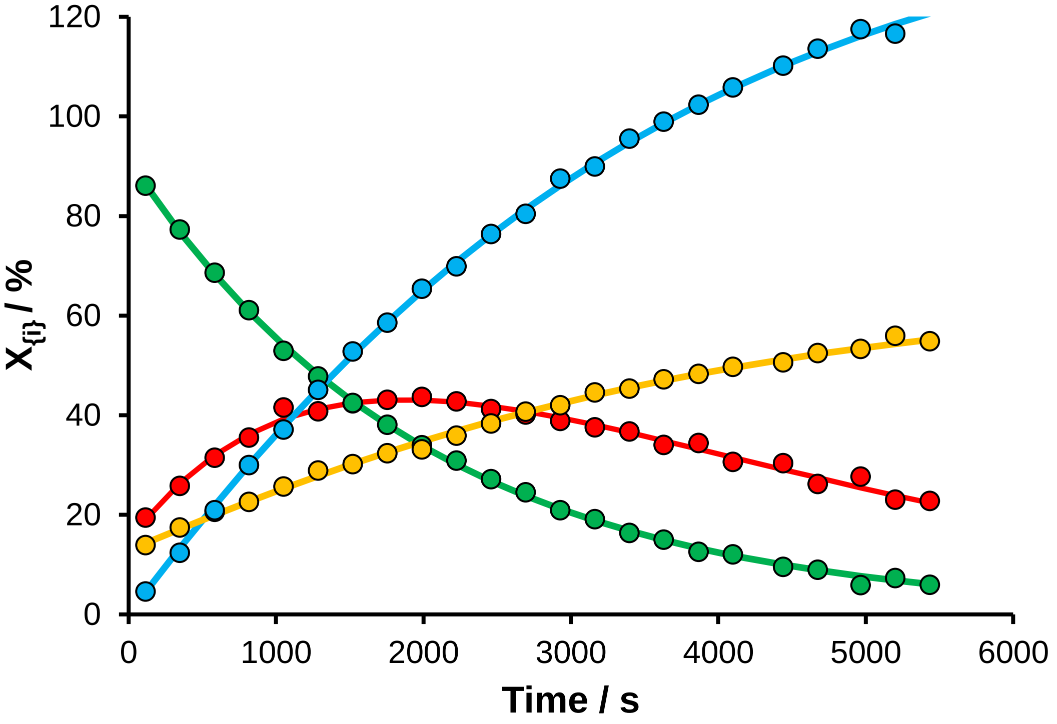

Figure S63: Reaction profiles, expressed in terms of mole fractions (X{i}), for the aminolysis and concurrent hydrolysis of **MepA-(PheF)$_2$** (**E_{Bis}**) in D_2O under the title conditions (20 °C; *I* = 2.0 M, KCl), as measured by in situ $^{19}F\{^1H\}$ NMR spectroscopy. The amide **P_{Am}** is the major product; the hydrolysis product, **P_{aa}**, is the minor product. The intermediate is **$E_{m,T}$**. Non-zero y-intercepts of **$X_{\{Paa\}}$** and **$X_{\{Em,T\}}$** are the result of pre-reaction hydrolysis in the stock solution of **E_{Bis}**. Initial mole fraction at the first time point i.e., (**$X_{\{Bis\}}$ + $X_{\{Paa\}}$ + $X_{\{Em,T\}}$**)$_{t=t1}$, normalised arbitrarily to 100%. Fit to kinetic model 3 shown. ᴸ**S** = L-serinamide.

pH*(20°C) = 6.965 ([^{L}**S**]$_T$ = 600 mM) ; D_2O

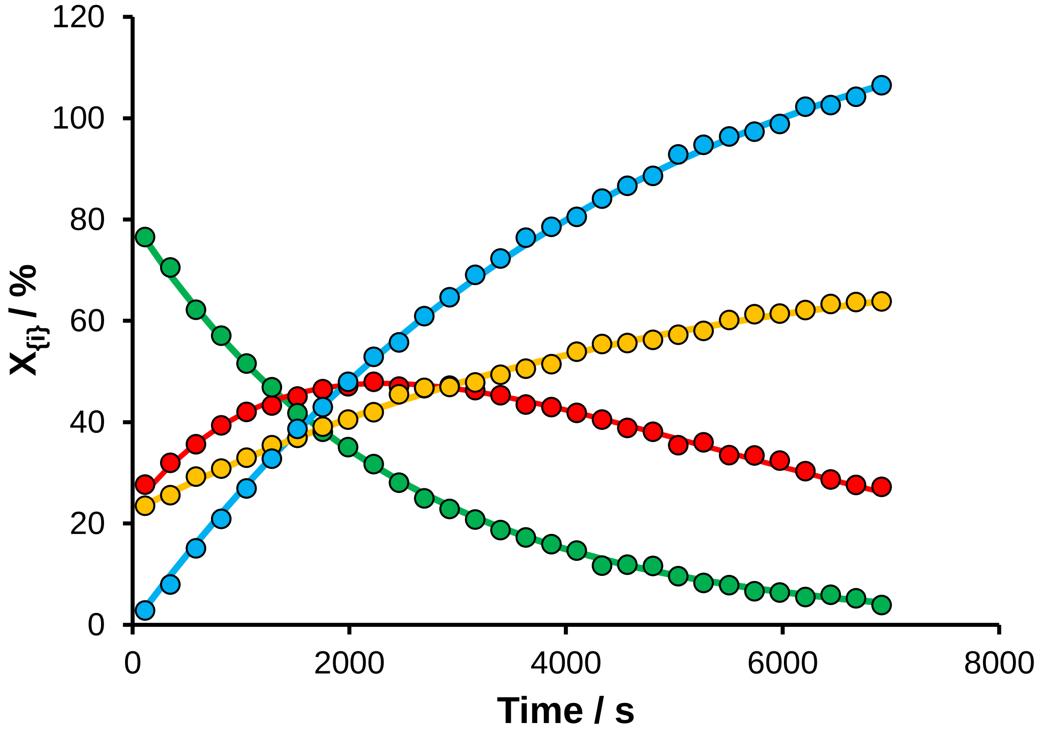

Figure S64: Reaction profiles, expressed in terms of mole fractions (X{i}), for the aminolysis and concurrent hydrolysis of **MepA-(PheF)$_2$** (**E$_{Bis}$**) in D_2O under the title conditions (20 °C; *I* = 2.0 M, KCl), as measured by in situ ^{19}F{^{1}H} NMR spectroscopy. The amide **P$_{Am}$** is the major product; the hydrolysis product, **P$_{aa}$**, is the minor product. The intermediate is **E$_{m,T}$**. Non-zero y-intercepts of **X$_{\{Paa\}}$** and **X$_{\{Em,T\}}$** are the result of pre-reaction hydrolysis in the stock solution of **E$_{Bis}$**. Initial mole fraction at the first time point i.e., (**X$_{\{Bis\}}$ + X$_{\{Paa\}}$ + X$_{\{Em,T\}}$**)$_{t=t1}$, normalised arbitrarily to 100%. Fit to kinetic model 3 shown. ^{L}**S** = L-serinamide.

pH*(20°C) = 6.482 ([^{L}**S**]$_T$ = 600 mM) ; D_2O

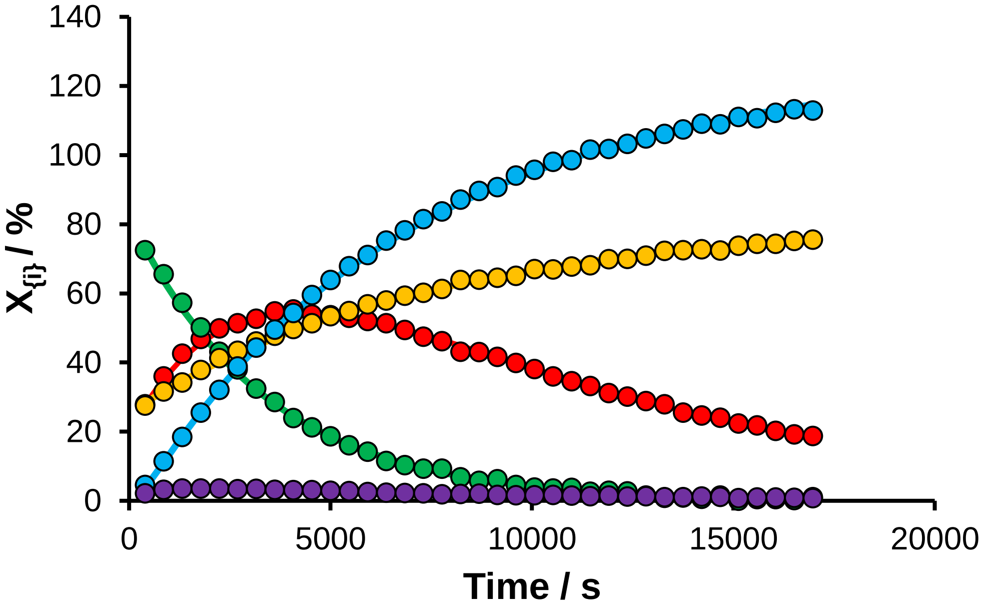

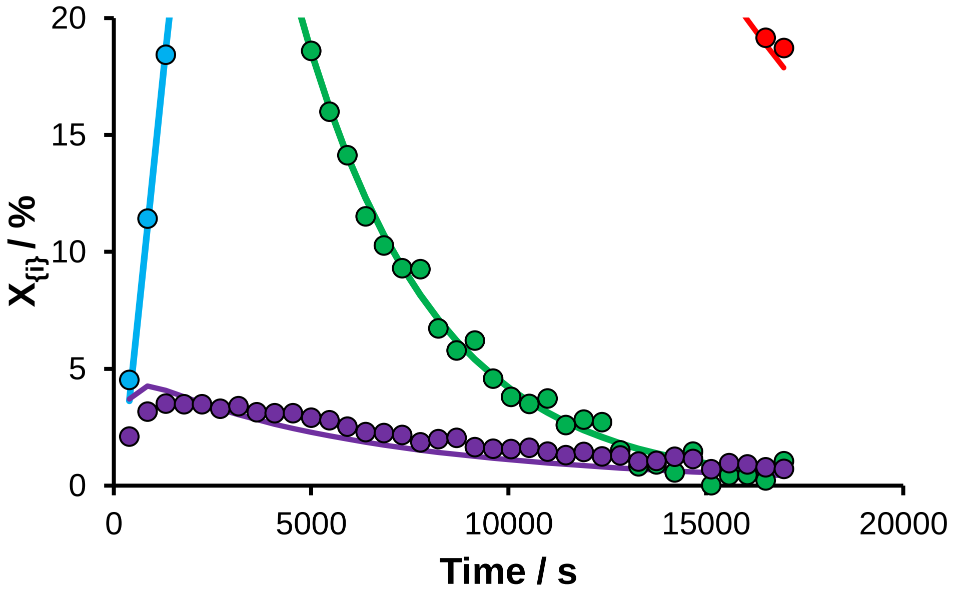

Figure S65: Reaction profiles, expressed in terms of mole fractions (X{i}), for the aminolysis and concurrent hydrolysis of **MepA-(PheF)$_2$** (**E$_{Bis}$**) in D_2O under the title conditions (20 °C; *I* = 2.0 M, KCl), as measured by in situ ^{19}F{^{1}H} NMR spectroscopy. The amide **P$_{Am}$** is the major product; the hydrolysis product, **P$_{aa}$**, is the minor product. The major intermediate is **E$_{m,T}$**; the minor intermediate is assigned as **I$_{Es}$**. Non-zero y-intercepts of **X$_{\{Paa\}}$** and **X$_{\{Em,T\}}$** are the result of pre-reaction hydrolysis in the stock solution of **E$_{Bis}$**. Initial mole fraction at the first time point i.e., (**X$_{\{Bis\}}$** + **X$_{\{Paa\}}$** + **X$_{\{Em,T\}}$** + **X$_{\{IEs\}}$**)$_{t=t1}$, normalised arbitrarily to 100%. Fit to kinetic model 4 shown. ^{L}**S** = L-serinamide.

pH*(20°C) = 6.006 ([^{L}**S**]$_T$ = 1200 mM) ; D_2O

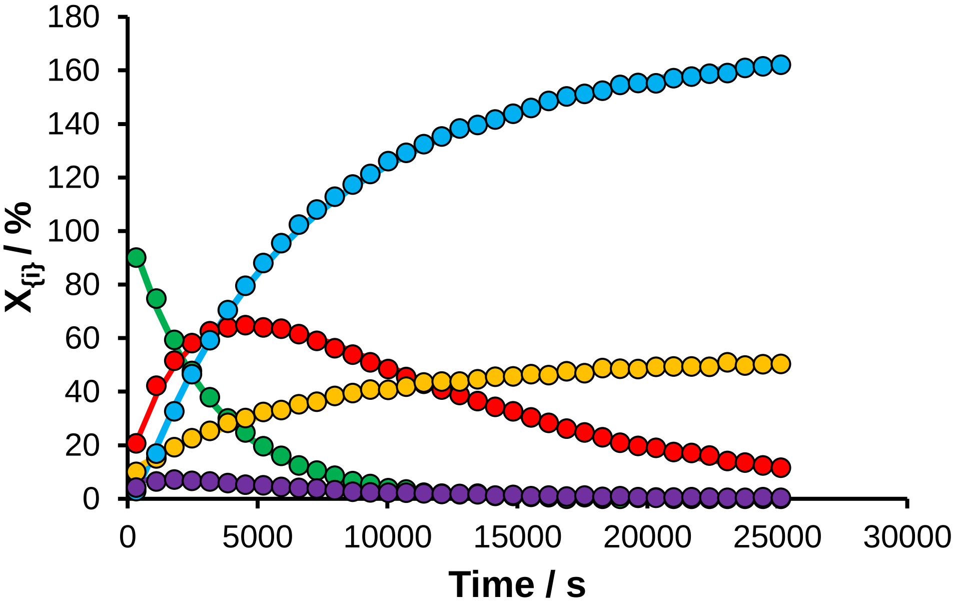

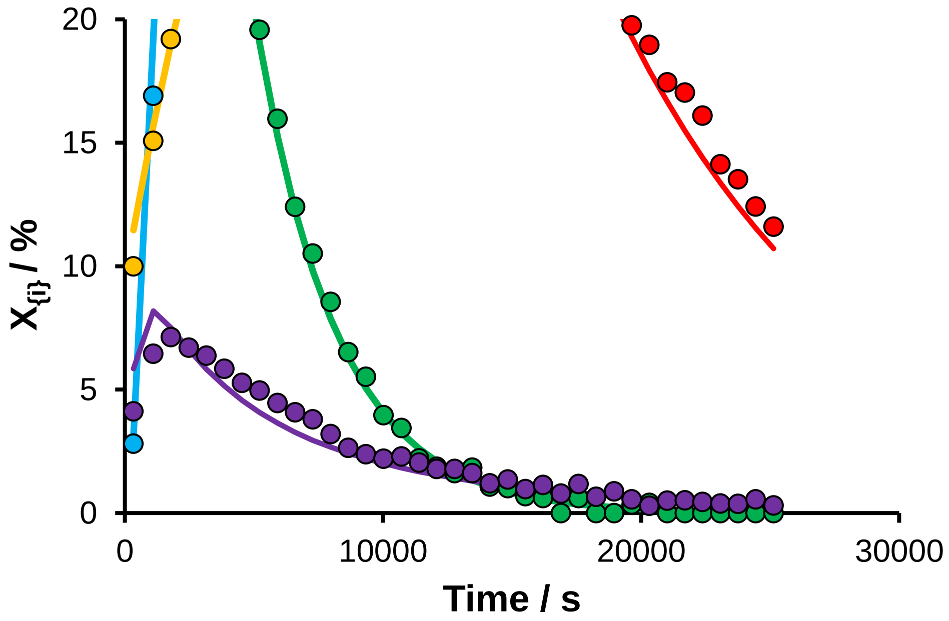

Figure S66: Reaction profiles, expressed in terms of mole fractions (X{i}), for the aminolysis and concurrent hydrolysis of **MepA-(PheF)**$_2$ (**E**$_{Bis}$) in D_2O under the title conditions (20 °C; *I* = 2.0 M, KCl), as measured by in situ ^{19}F{^{1}H} NMR spectroscopy. The amide **P**$_{Am}$ is the major product; the hydrolysis product, **P**$_{aa}$, is the minor product. The major intermediate is **E**$_{m,T}$; the minor intermediate is assigned as **I**$_{Es}$. Non-zero y-intercepts of **X**$_{\{Paa\}}$ and **X**$_{\{Em,T\}}$ are the result of pre-reaction hydrolysis in the stock solution of **E**$_{Bis}$. Initial mole fraction at the first time point i.e., (**X**$_{\{Bis\}}$ + **X**$_{\{Paa\}}$ + **X**$_{\{Em,T\}}$ + **X**$_{\{IEs\}}$)$_{t=t1}$, normalised arbitrarily to 100%. Fit to kinetic model 4 shown. ^{L}**S** = L-serinamide.

pH*(20°C) = 5.613 ([^{L}**S**]$_T$ = 2000 mM) ; D_2O

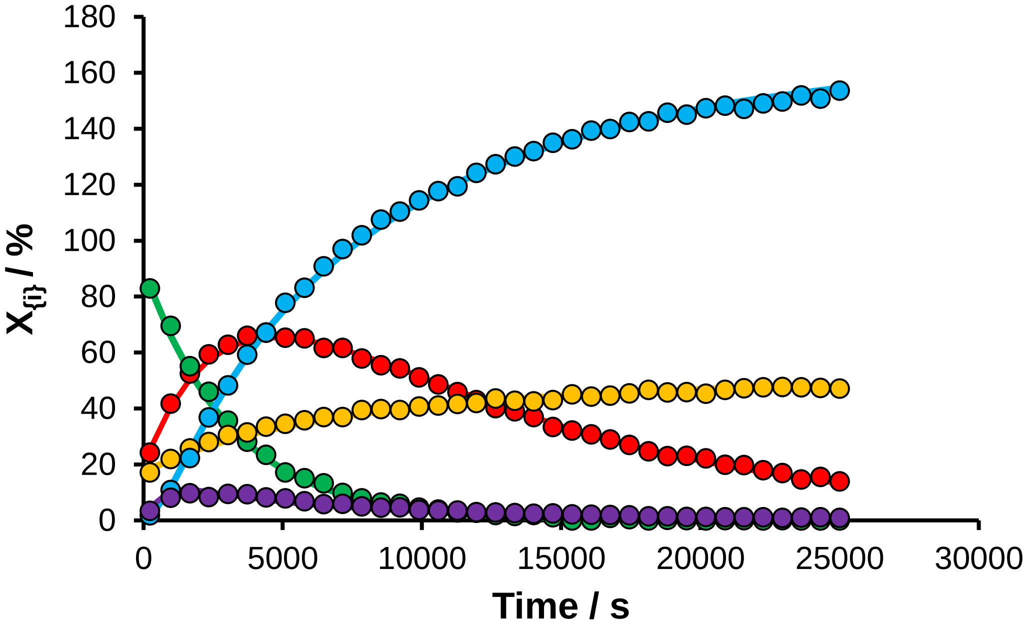

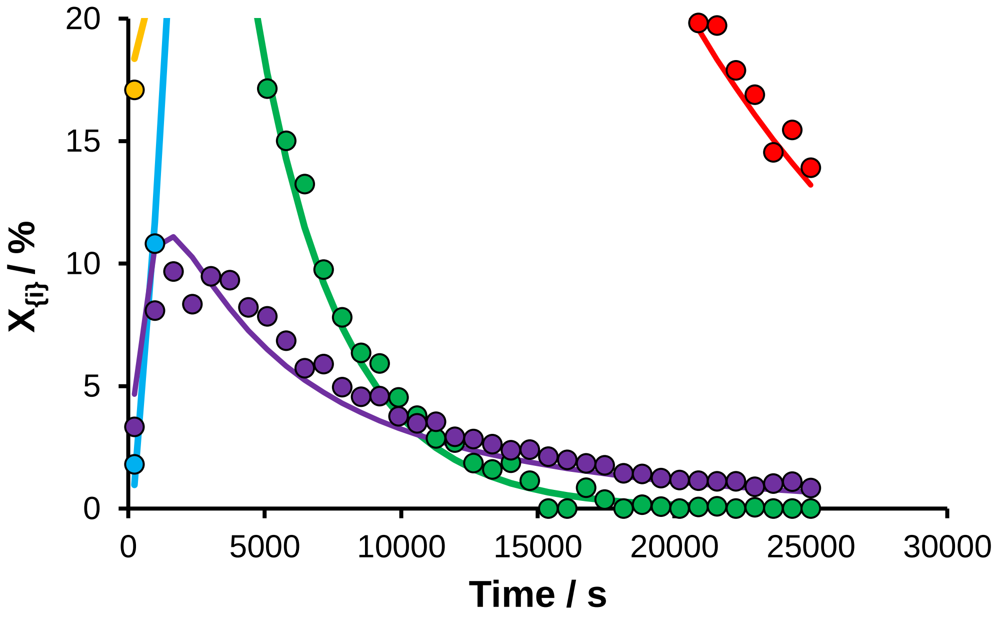

Figure S67: Reaction profiles, expressed in terms of mole fractions (X{i}), for the aminolysis and concurrent hydrolysis of **MepA-(PheF)**$_2$ (**E**$_{Bis}$) in D_2O under the title conditions (20 °C; *I* = 2.0 M, KCl), as measured by in situ $^{19}F\{^1H\}$ NMR spectroscopy. The amide **P**$_{Am}$ is the major product; the hydrolysis product, **P**$_{aa}$, is the minor product. The major intermediate is **E**$_{m,T}$; the minor intermediate is assigned as **I**$_{Es}$. Non-zero y-intercepts of **X**$_{\{Paa\}}$ and **X**$_{\{Em,T\}}$ are the result of pre-reaction hydrolysis in the stock solution of **E**$_{Bis}$. Initial mole fraction at the first time point i.e., (**X**$_{\{Bis\}}$ + **X**$_{\{Paa\}}$ + **X**$_{\{Em,T\}}$ + **X**$_{\{IEs\}}$)$_{t=t1}$, normalised arbitrarily to 100%. Fit to kinetic model 4 shown. ^{L}**S** = L-serinamide.

Aminoacyl ester synthesis

*Adenosine-5‘-O-methylphosphate (**MepA**)*

Adenosine-5’-monophosphate monohydrate (acid; 1.0 g, 2.7 mmol) and N,N’-dicyclohexylcarbodiimide (2.82 g, 13.7 mmol, 5.0 equiv.) were weighed into a round-bottomed flask (100 mL) and suspended in methanol (27 mL) under a nitrogen atmosphere. N,N-diisopropylethylamine (0.96 mL, 5.5 mmol, 2.0 equiv.) was then added over the course of 30 seconds to afford a colourless solution, which was then stirred at ambient temperature under N_2; after 24 h, concentrated sodium hydroxide (1.5 mL, 4.0 M, 2.2 equiv.) was added and the solution stirred vigorously for 30 min. The solution was then evaporated to dryness in vacuo (50 °C), resuspended in deionised water, filtered, and lyophilised; the lyophilizate was resuspended in water again and this process repeated to afford a white solid (MepA, 1.05 g, quant.) that was used without further purification.

^{1}H NMR (400 MHz, D_2O): δ_H / ppm = 8.39 (1H, s, *H*2), 8.19 (1H, s, *H*8), 5.98 (1H, d, $^3J_{H1'-H2'}$ = 5.4 Hz, *H*1’), 4.71 (1H, app t., $^3J_{H1'-H2'}$ = $^3J_{H2'-H3'}$ = 5.4 Hz, *H*2’), 4.36 (1H, dd, $^3J_{H2'-H3'}$ = 5.4 Hz, $^3J_{H3'-H4'}$ = 4.0 Hz, *H*3’), 4.30 (1H, m, H4’), 4.10 (2H, m, H5’), 3.51 (3H, d, $^3J_{CH3-P}$ = 10.8 Hz, C*H*$_3$).

^{13}C{^{1}H} NMR (101 MHz, D_2O): δ_C / ppm = 155.4 (C6), 152.7 (C8), 149.0 (C4), 139.8 (C2), 118.5 (C5), 88.0 (C1’), 84.1 (d, $^3J_{C4'-P}$ = 8.6 Hz, C4’), 75.3 (C2’), 71.2 (C3’), 65.2 (d, $^2J_{C5'-P}$ = 5.3 Hz, C5’), 52.9 (d, $^2J_{CH3-P}$ = 5.9 Hz, *C*H$_3$)

^{31}P{^{1}H} NMR (162 MHz, D_2O): δ_P / ppm = 1.60 (s)

ESI-MS(M+H): 362.0 (calc. 362.1)

*2'(3')-O-(4-fluoro-L-phenylalanyl)-adenosine-5'-(O-methylphosphate) (**E$_m$**)*

N-Boc-4-fluoro-L-phenylalanine (86 mg, 0.31 mmol, 1.1 equiv.) and carbonyldiimidazole (50 mg, 0.31 mmol, 0.1 equiv.) were dissolved in MeCN (0.10 M) and the resulting solution was stirred under ambient conditions for 20 min. An equal volume of an aqueous solution of adenosine-5‘-O-methylphosphate (MepA) (100 mg, 0.28 mmol, 1.0 equiv.) was then added, with rapid mixing, and the combined solution was stirred for a further 30 min, after which the reaction was quenched with concentrated HCl (ca. 180 µL, 4.0 M) to achieve an apparent pH of 3 – 4. The acidified solution was evaporated to dryness *in vacuo*, redissolved by addition of 10 mL of a 70:30 (v:v) mixture of aqueous formic acid (20 mM) and MeCN, respectively, and subjected to purification by preparative HPLC (20 – 60% B; A = aqueous formic acid (20 mM, pH 2.3); B = acetonitrile). The 2’- and 3’-isomers of O-(N-Boc-4-fluoro-L-phenylalanyl)-adenosine-5'-(O-methylphosphate), and the bis-aminoacylated product, were isolated as separate compounds and their identities assigned by ESI(+)-MS, after which the fractions containing the 2’- and 3’-isomers were combined.

Solutions containing, separately, the mono- and bis-aminoacylated products were then lyophilised, and the corresponding lyophilisates in turn dissolved in neat trifluoroacetic acid (2 mL) and stirred under an atmosphere of N_2 for 15 min. In each case the trifluoroacetic acid was evaporated under reduced pressure (40 °C), with the residue suspended in ethyl acetate (10 mL) and then evaporated a total of three times. The final residue in each case was redissolved by addition (10 mL) of a 80:20 (v:v) mixture of aqueous formic acid (20 mM) and MeCN, respectively, and subjected to purification by preparative HPLC (10 – 50% B; A = aqueous formic acid (20 mM, pH 2.3); B = acetonitrile). The 2'- and 3'-isomers of O-(4-fluoro-L-phenylalanyl)-adenosine-5'-(O-methylphosphate) were isolated together, the combined solution lyophilised, and the corresponding lyophilizate dissolved in pure D_2O or H_2O to afford an aqueous stock solution (pH* = 5, typically 50 – 150 mM) of the trifluoroacetate salt of the title compound as a mixture of the 2'- and 3'-isomers (equilibration was exceptionally rapid). The isolated yield was determined by measurement of the absorbance of this solution at 260 nm, assuming an extinction coefficient of $\varepsilon_{260} = 1.5 \times 10^4$ M^{-1} cm^{-1}, and was generally found to be 25 – 30%. Aqueous stock solutions were generally stable over a period of weeks when stored at -28 °C.

^{1}H NMR (400 MHz, D_2O): δ_H / ppm **=** 8.53 (1H, s, H2; 3'), 8.50 (1H, s, H2; 2'), 8.37 (1H, s, H8; 3'), 8.31 (1H, s, H8; 2'), 7.42 (2H, m, Ar-*H*; 3'), 7.24 (2H, m, Ar-*H*; 2'+3'), 6.98 (2H, m, Ar-*H*; 2'), 6.29 (1H, d, $^3J_{H1'-H2'}$ = 5.2 Hz, H1'; 2'), 6.01 (1H, d, $^3J_{H1'-H2'}$ = 7.4 Hz, H1'; 3'), 5.78 (1H, app. t, $^3J_{H1'-H2'}$ = $^3J_{H2'-H3'}$ = 5.1 Hz, H2'; 2'), 5.62 (1H, dd, $^3J_{H3'-H4'}$ = 2.0 Hz, $^3J_{H2'-H3'}$ = 5.3 Hz, H3'; 3'), 5.08 (1H, dd, $^3J_{H1'-H2'}$ = 7.4 Hz , $^3J_{H2'-H3'}$ = 5.3 Hz, H2'; 3'), 4.86 (1H, app. t, $^3J_{H2'-H3'}$ = $^3J_{H3'-H4'}$ = 5.1 Hz, H3'; 2'), 4.66 (1H, app. t, $^3J_{H\alpha-H\beta}$ = $^3J_{H\alpha-H\beta*}$ = 7.4 Hz, Hα; 3'), 4.62 (1H, app. t, $^3J_{H\alpha-H\beta}$ = $^3J_{H\alpha-H\beta*}$ = 6.5 Hz, Hα; 2'), 4.42 (1H, m, H4'; 2'), 4.34 (1H, m, H4'; 3'), 4.18 (2H, m, H5'+H5'*; 2'+3'), 3.63 (3H, d, $^3J_{CH3-P}$ = 10.7 Hz, CH_3; 2'), 3.62 (3H, d, $^3J_{CH3-P}$ = 10.7 Hz, CH_3; 3'), 3.42 (2H, d, $^3J_{H\alpha-H\beta}$ = $^3J_{H\alpha-H\beta*}$ = 7.4 Hz, Hβ + Hβ*; 3'), 3.35 (2H, m, Hβ + Hβ*; 2'). (3' = 73%; 2' = 27%)

^{13}C{^{1}H} NMR (101 MHz, D_2O): δ_C / ppm (3' only) **=** 168.6 (*C*O), 162.2 (d, $^1J_{C-F}$ = 240 Hz, ipso-C; approx. from ^{13}C-^{1}H HMBC), 153.6 (C6, HMBC), 150.1 (C8), 149.2 (C4), 140.5 (C2), 131.1 (d, $^3J_{C-F}$ = 8.7 Hz, meta-C), 129.8 (d, $^4J_{C-F}$ = 2.9 Hz, para-C), 118.6 (C5), 116.0 (d, $^2J_{C-F}$ = 21.9 Hz, ortho-C), 86.3 (C1'), 81.9 (C4'), 75.5 (C3'), 72.6 (C2'), 64.7 (C5'), 54.0 (Cα), 53.1 (*C*H_3), 35.5 (Cβ)

^{31}P{^{1}H} NMR (162 MHz, D_2O): δ_P / ppm **=** 1.52 (s; 2'-isomer, ca. 27%), 1.41 (s; 3'-isomer, ca. 73%)

^{19}F{^{1}H} NMR (377 MHz, D_2O): δ_F / ppm **=** -114.50 (s; 2'isomer, ca. 27%), -114.89 (s; 3'-isomer, ca. 73%).

ESI-MS(M+H): 527.0 (calc. 527.1)

*2',3'-bis-O-(4-fluoro-L-phenylalanyl)-adenosine-5'-(O-methylphosphate) (**E_{Bis}**)*

Following synthesis and purification by preparative HPLC alongside **MepA-L-PheF** (**E_m**), *vide supra*, the lyophilizate of the title compound was dissolved in pure D_2O or H_2O to afford an aqueous stock solution of its trifluoroacetate salt (pH* = 5). The isolated yield was determined by measurement of the absorbance of this solution at 260 nm, assuming an extinction coefficient of $\varepsilon_{260} = 1.5 \times 10^4$ M^{-1} cm^{-1}, and was generally found to be 10 – 15%. Aqueous stock solutions were generally stable over a period of days when stored at -28 °C; the title compound was much more susceptible to hydrolysis at relative acidic pH than the corresponding mono-aminoacyl species (*vide supra*).

^{1}H NMR (400 MHz, D_2O): δ_H / ppm **=** 8.35 (1H, s, H2), 8.18 (1H, s, H8), 7.28 (2H, m, Ar-*H*; 3'), 7.08 (2H, m, Ar-*H*; 3'), 7.04 (2H, m, Ar-H; 2'), 6.89 (2H, m, Ar-H; 2'), 6.01 (1H, d, $^3J_{H1'-H2'}$ = 6.4 Hz, H1'), 5.84 (1H, dd, $^3J_{H1'-H2'}$ = 6.4 Hz, $^3J_{H2'-H3'}$ = 5.3 Hz, H2'), 5.75 (1H, dd, $^3J_{H2'-H3'}$ = 5.3 Hz, $^3J_{H3'-H4'}$ = 2.9 Hz, H3'), 4.44 (1H, t, $^3J_{H\alpha-H\beta}$ = 7.6 Hz, Hα; 3'), 4.35 (1H, t, $^3J_{H\alpha-H\beta}$ = 6.8 Hz, Hα; 2'), 4.26 (1H, app. p, J = 2.6 Hz, H4'), 4.11 (2H, m, H5'), 3.54 (3H, d, $^3J_{CH3-P}$ = 10.8 Hz, C*H*$_3$), 3.27 (2H, m, Hβ+Hβ*; 3'), 3.06 (2H, m, Hβ+Hβ*; 2').

^{13}C{^{1}H} NMR (101 MHz, D_2O): δ_C / ppm = 168.7 (CO; 3'), 167.8 (CO, 2'), 162.2 (d, $^1J_{C-F}$ = 244 Hz, ipso-C; 3'), 162.0 (d, $^1J_{C-F}$ = 244 Hz, ipso-C; 2'), 155.1 (C6; HMBC), 152.5 (C8), 148.8 (C4), 139.7 (C2), 131.1 (d, $^3J_{C-F}$ = 8.5 Hz, meta-C; 3'), 130.8 (d, $^3J_{C-F}$ = 8.4 Hz, meta-C; 2'), 129.9 (d, $^4J_{C-F}$ = 3.0 Hz, ipso-C; 3'), 129.2 (d, $^4J_{C-F}$ = 3.0 Hz, ipso-C; 2'), 118.4 (C5), 116.0 (d, $^2J_{C-F}$ = 22.0 Hz, ortho-C; 3'), 115.7 (d, $^2J_{C-F}$ = 21.8 Hz, ortho-C; 2'), 84.2 (C1'), 82.0 (d, $^3J_{C4'-P}$ = 8.8 Hz, C4'), 75.0 (C2'), 73.3 (C3'), 64.3 (d, $^2J_{C5'-P}$ = 5.0 Hz, C5'), 53.8 (Cα; 3'), 53.6 (Cα, 2'), 53.0 (d, $^2J_{CH3-P}$ = 5.8 Hz, *C*H$_3$), 35.2 (Cβ; 3'), 34.9 (Cβ; 2').

^{31}P{^{1}H} NMR (162 MHz, D_2O): δ_P / ppm **=** 1.31

^{19}F{^{1}H} NMR (377 MHz, D_2O): δ_F / ppm **=** -114.43 (2'), -114.69 (3') (+ MepA-L-PheF + L-Phe-OH)

ESI-MS(M+H): 692.2 (calc. 692.0)

2'(3')-O-(4-fluoro-D-phenylalanyl)-adenosine-5'-(O-methylphosphate) ($\mathbf{E_m^D}$)

Synthesised and purified in an identical manner to the diastereomer 2'(3')-O-(4-fluoro-L-phenylalanyl)-adenosine-5'-(O-methylphosphate) (**MepA-L-PheF**; $\mathbf{E_m}$) (isolated yield 25%).

^{1}H NMR (400 MHz, D_2O): δ_H / ppm = 8.55 (1H, s, H2; 3'), 8.46 (1H, s, H2; 2'), 8.35 (1H, s, H8; 3'), 8.29 (1H, s, H8; 2'), 7.41 (2H, m, Ar-H; 3'), 7.20 (2H, m, Ar-H*; 3'), 7.07 (2H, m, Ar-H; 2'), 6.74 (2H, m, Ar-H*; 2'), 6.16 (1H, d, $^3J_{H1'-H2'}$ = 7.0 Hz, H1'; 3'), 6.15 (1H, d, $^3J_{H1'-H2'}$ = 6.4 Hz, H1'; 2'), 5.68 (1H, dd, $^3J_{H2'-H3'}$ = 5.4 Hz, $^3J_{H3'-H4'}$ = 2.4 Hz, H3'; 3'), 5.61 (1H, dd, $^3J_{H1'-H2'}$ = 6.3 Hz, $^3J_{H2'-H3'}$ = 5.4 Hz, H2'; 2'), 5.13 (1H, dd, $^3J_{H1'-H2'}$ = 7.0 Hz, $^3J_{H2'-H3'}$ = 5.4 Hz, H2'; 3'), 4.84 (1H, dd, $^3J_{H2'-H3'}$ = 5.4 Hz, $^3J_{H3'-H4'}$ = 3.2 Hz, H3'; 2'), 4.66 (1H, dd, $^3J_{H\alpha-H\beta}$ = 7.9 Hz, $^3J_{H\alpha-H\beta*}$ = 6.0 Hz, Hα; 3'), 4.58 (1H, dd, $^3J_{H\alpha-H\beta}$ = 9.3 Hz, $^3J_{H\alpha-H\beta*}$ = 6.0 Hz; Hα; 2'), 4.57 (1H, m, H4'; 3'), 4.40 (1H, app pent., J = 2.8 Hz, H4'; 2'), 4.17 (2H, m, H5'; 2'+3'), 3.62 (3H, d, $^3J_{CH3-P}$ = 10.8 Hz, C*H*$_3$; 2'), 3.61 (3H, d, $^3J_{CH3-P}$ = 10.8 Hz, C*H*$_3$; 3'), 3.53 (1H, dd, $^2J_{H\beta-H\beta*}$ = 14.9 Hz, $^3J_{H\alpha-H\beta*}$ = 6.0 Hz; Hβ/Hβ*; 2'), 3.33 (1H, m, Hβ/Hβ*; 2'+3'), 3.14 (1H, dd, $^2J_{H\beta-H\beta*}$ = 14.9 Hz, $^3J_{H\alpha-H\beta*}$ = 9.3 Hz, Hβ/Hβ*; 2'). (3' = 54%; 2' = 46%)

^{13}C{^{1}H} NMR (101 MHz, D_2O): δ_C / ppm = 168.8 (CO; 2'), 168.6 (CO; 3'), 162.2 (d, $^1J_{C-F}$ = 240 Hz (HMBC), ipso-C; 3'), 161.6 (d, $^1J_{C-F}$ = 240 Hz (HMBC), ipso-C; 2'), 154.6 (C6; 2'), 153.8 (C6; 3'), 151.2 (C8; 2'), 149.1 (C8; 3'), 140.5 (C2; 3'), 140.0 (C2; 2'), 131.2 (d, $^3J_{C-F}$ = 8.6 Hz, meta-C; 3'), 130.5 (d, $^3J_{C-F}$ = 8.6 Hz, meta-C; 2'), 129.5 (d, $^4J_{C-F}$ = 3.0 Hz, para-C; 3'), 129.2 (d, $^4J_{C-F}$ = 3.0 Hz, para-C; 2'), 118.6 (C5; 3'), 118.5 (C5; 2'), 116.0 (d, $^2J_{C-F}$ = 21.6 Hz, ortho-C; 3'), 115.2 (d, $^2J_{C-F}$ = 21.7 Hz, ortho-C; 2'), 86.6 (C1'; 3'), 84.7 (d, $^3J_{C4'-P}$ = 8.6 Hz, C4'; 2'), 84.3 (C1'; 2'), 81.7 (d, $^3J_{C4'-P}$ = 7.9 Hz, C4'; 3'), 77.3 (C2'; 2'), 75.3 (C3'; 3'), 72.6 (C2'; 3'), 69.0 (C3'; 2'), 64.7 (C5'; 2'+3'), 54.0 (C4'; 3'), 53.6 (C4'; 2'), 52.9 (d, $^2J_{CH3-P}$ = 5.8 Hz, *C*H$_3$), 35.2 (Cβ; 2'/3'), 34.9 (Cβ, 2'/3').

^{31}P{^{1}H} NMR (162 MHz, D_2O): δ_P / ppm **=** 1.49 (2'), 1.47 (3')

^{19}F{^{1}H} NMR (377 MHz, D_2O): δ_F / ppm **=** -114.4 (2'), -114.9 (3')

ESI-MS(M+H): 526.9 (calc. 527.1)

N-formyl-4-fluorophenylalanine (f-PheF-OH)

4-Fluoro-L-phenylalanine (L-PheF-OH; 300 mg, 1.64 mmol) was dissolved in formic acid (2.4 mL) and the resulting solution was cooled on ice and placed under an N_2 atmosphere. Acetic anhydride (1.4 mL, 8.0 equiv.) was added dropwise over 10 min, after which the fully assembled reaction was stirred for a further 20 min on ice and then overnight at room temperature. Deionised water (5 mL) was added to quench residual acetic anhydride, after which the solution stirred for 15 min and then evaporated to dryness *in vacuo.* The crude solid was recrystallised from water, with the saturated solution refluxed for 10 min before cooling to room temperature, to afford a white solid (201 mg, 58 %).

^{1}H NMR (400 MHz, CD_3CN): δ_H / ppm **=** 8.04 (1H, s, C*H*O; trans, 92%), 7.68 (1H, d, $^3J_{NH\text{-}CHO}$ = 11.5 Hz, C*H*O; cis, 8%), 7.24 (2H, m, Ar-H; cis+trans), 7.04 (2H, m, Ar-H; cis+trans), 6.78 (1H, s, N*H*; br s; trans), 6.42 (1H, t, $^3J_{NH\text{-}CHO}$ = 11.5 Hz, N*H*; cis), 4.70 (1H, tdd, $^3J_{H\alpha\text{-}H\beta}$ = 8.0 Hz, $^3J_{H\alpha\text{-}NH}$ = 5.4 Hz, $^4J_{H\alpha\text{-}CHO}$ = 0.8 Hz, Hα; trans), 4.34 (1H, td, $^3J_{H\alpha\text{-}H\beta}$ = 9.7 Hz, $^3J_{H\alpha\text{-}NH}$ = 4.8 Hz, Hα; cis), 3.07 (2H, m, Hβ; cis+trans).

^{13}C{^{1}H} NMR (101 MHz, CD_3CN): δ_C / ppm = 172.3 (CO; cis), 172.1 (CO; trans), 164.7 (CHO; cis), 162.4 (d, $^1J_{C\text{-}F}$ = 242 Hz, ipso-C; trans), 161.7 (CHO; trans), 133.4 (d, $^4J_{C\text{-}F}$ = 3.1 Hz, para-C; trans), 131.9 (d, $^3J_{C\text{-}F}$ = 7.8 Hz, meta-C; cis), 131.7 (d, $^3J_{C\text{-}F}$ = 7.8 Hz, meta-C; trans), 115.8 (d, $^2J_{C\text{-}F}$ = 21.0 Hz, ortho-C; cis), 115.5 (d, $^2J_{C\text{-}F}$ = 21.0 Hz, ortho-C; trans), 56.4 (Cα; cis), 52.5 (Cα; trans), 38.3 (Cβ; cis), 36.7 (Cβ; trans).

^{19}F{^{1}H} NMR (377 MHz, CD_3CN): δ_F / ppm **=** -117.76 (cis), -117.96 (trans)

ESI-MS(M+H): 212.1 (calc. 212.1)

*2'(3')-O-(N-formyl-4-fluoro-L/D-phenylalanyl)-adenosine-5'-(O-methylphosphate) (**E$_m$f**)*

N-formyl-4-fluoro-L-phenylalanine (31 mg, 0.15 mmol, 1.1 equiv.) and carbonyldiimidazole (24 mg, 0.31 mmol, 1.1 equiv.) were dissolved in MeCN (0.10 M) and the resulting solution was stirred under ambient conditions for 20 min. An equal volume of an aqueous solution of adenosine-5'-O-methylphosphate (MepA) (50 mg, 0.14 mmol, 1.0 equiv.) was then added, with rapid mixing, and the combined solution was stirred for a further 30 min, after which the reaction was quenched with concentrated HCl (ca. 90 µL, 4.0 M) to achieve an apparent pH of 3 – 4. The acidified solution was evaporated to dryness *in vacuo*, redissolved by addition of 10 mL of a 80:20 (v:v) mixture of aqueous formic acid (20 mM) and MeCN, respectively, and subjected to purification by preparative HPLC (10 – 50% B; A = aqueous formic acid (20 mM, pH 2.3); B = acetonitrile).

Four peaks with the same mass (ESI(+)-MS), corresponding to the title compound, were observed during HPLC purification, apparently the result of racemisation of the α-stereocentre in the aminoacyl fragment during CDI activation. A single isomer of the title compound was isolated cleanly, the combined fractions lyophilised, and the lyophilizate dissolved in pure D_2O or H_2O to

afford an aqueous stock solution (pH* = 5) of the title compound. The isolated yield (7%) was determined by measurement of the absorbance of this solution at 260 nm, assuming an extinction coefficient of ε_{260} = 1.5 × 10^4 M^{-1} cm^{-1}.

2'/3'-equilibration of the title compound at moderately acidic pH* was slower than the free aminoacyl ester, but still apparently faster than hydrolysis, and the NMR characterisation data below corresponds to a non-equilibrium mixture of 2'- and 3'-isomers. The preponderance of the 2'-regioisomer in the NMR data is in contrast to the preponderance of the 3' isomer at equilibrium – observed during kinetics experiments at higher pH* - suggesting it was a single diastereomer of *the 2'-regioisomer* that was initially isolated during HPLC purification (though this is not kinetically significant, given the rapid 2'/3'-equilibration at higher pH*).

The absolute stereochemistry of this diastereomer was not confirmed explicitly, but *in situ* monitoring of its aminolysis/hydrolysis in aqueous solution by $^{19}F\{^1H\}$ NMR showed that the *equilibrium* 2'/3' ratio, rapidly achieved at higher pH* (7 – 8), was essentially identical to that of the free (i.e., non-formylated) aminoacyl ester 2'(3')-O-(4-fluoro-**L**-phenylalanyl)-adenosine-5'-(O-methylphosphate. The diastereomer 2'(3')-O-(4-fluoro-**D**-phenylalanyl)-adenosine-5'-O-methylphosphate was found to exhibit a materially different equilibrium distribution of the 2' and 3'-regioisomers in aqueous solution.

^{1}H NMR (400 MHz, D_2O): δ_H / ppm **=** 8.67 (1H, s, H2; 3', cis), 8.65 (1H, s, H2; 3', trans), 8.64 (1H, s, H2; 2', cis), 8.63 (1H, s, H2; 2', trans), 8.52 (1H, s, H8; 3', cis+trans), 8.47 (1H, s, H8; 2', cis+trans), 8.16 (1H, br s, C*H*O; 3', trans), 8.10 (1H, br s, CHO; 2', trans), 7.86 (1H, s, C*H*O; 3', cis), 7.84 (1H, s, C*H*O; 2' cis), 7.41 (2H, m, Ar-H; 3', cis+trans), 7.24 (2H, m, Ar-H; 2', cis+trans), 7.20 (2H, m, Ar-H*; 3', cis+trans), 7.00 (2H, m, Ar-H*; 2', cis+trans), 6.40 (1H, d, $^3J_{H1'-H2'}$ = 4.9 Hz, H1'; 2', cis), 6.34 (1H, d, $^3J_{H1'-H2'}$ = 5.1 Hz, H1'; 2', trans), 6.16 (1H, d, $^3J_{H1'-H2'}$ = 7.0 Hz, H1'; 3', cis), 6.07 (1H, d, $^3J_{H1'-H2'}$ = 7.0 Hz, H1'; 3', trans), 5.73 (1H, t , $^3J_{H1'-H2'}$ = $^3J_{H2'-H3'}$ = 5.2 Hz, H2'; 2', cis), 5.71 (1H, t, $^3J_{H1'-H2'}$ = $^3J_{H2'-H3'}$ = 5.2 Hz, H2'; 2', trans), 5.59 (1H, dd, $^3J_{H2'-H3'}$ = 5.2 Hz, $^3J_{H3'-H4'}$ = 2.3 Hz, H3'; 3', cis), 5.50 (1H, dd, $^3J_{H2'-H3'}$ = 5.3 Hz, $^3J_{H3'-H4'}$ = 2.4 Hz, H3'; 3', trans), 5.11 (1H, dd, $^3J_{H1'-H2'}$ = 7.0 Hz, $^3J_{H2'-H3'}$ = 5.2 Hz, H2'; 3' cis), 5.07 (1H, dd, $^3J_{H1'-H2'}$ = 7.0 Hz, $^3J_{H2'-H3'}$ = 5.4 Hz, H2'; 3', trans), 5.00 (1H, m, Hα; 2'+3', cis+trans), 4.87 (1H, t, $^3J_{H2'-H3'}$ = $^3J_{H3'-H4'}$ = 5.2 Hz, H3'; 2', cis), 4.83 (1H, t, $^3J_{H2'-H3'}$ = $^3J_{H3'-H4'}$ = 4.9 Hz, H3'; 2', trans), 4.46 (1H, m, H4'; 2', cis+trans), 4.28 (1H, m H4'; 3', cis+trans), 4.21 (2H, m, H5'; 2', cis+trans), 4.13 (2H, m, H5'; 3', cis+trans), 3.63 (3H, d, $^3J_{CH3-P}$ = 10.7 Hz, C*H*$_3$; 2', cis), 3.63 (3H, d, $^3J_{CH3-P}$ = 10.7 Hz, C*H*$_3$; 2', trans), 3.62 (3H, d, $^3J_{CH3-P}$ = 10.8 Hz, C*H*$_3$; 3', cis), 3.61 (3H, d, $^3J_{CH3-P}$ = 10.8 Hz, C*H*$_3$; 3', trans), 3.21 (2H, m, Hβ+Hβ*; 2'+3', cis+trans). (2', trans = 61%; 3', trans = 31%; 2', cis = 5%; 3', cis = 3%).

$^{31}P\{^1H\}$ NMR (162 MHz, D_2O): δ_P / ppm **=** 1.54 (2', cis), 1.52 (2', trans), 1.43 (3', cis), 1.39 (3', trans)

$^{19}F\{^1H\}$ NMR (377 MHz, D_2O): δ_F / ppm **=** -115.91 (2', cis), -115.92 (2', trans), -116.03 (3', trans), -116.07 (3', trans)

ESI-MS(M+H): 554.9 (calc. 555.1)

Raw characterisation data: NMR

MepA

^{1}H (400 MHz, D_2O)

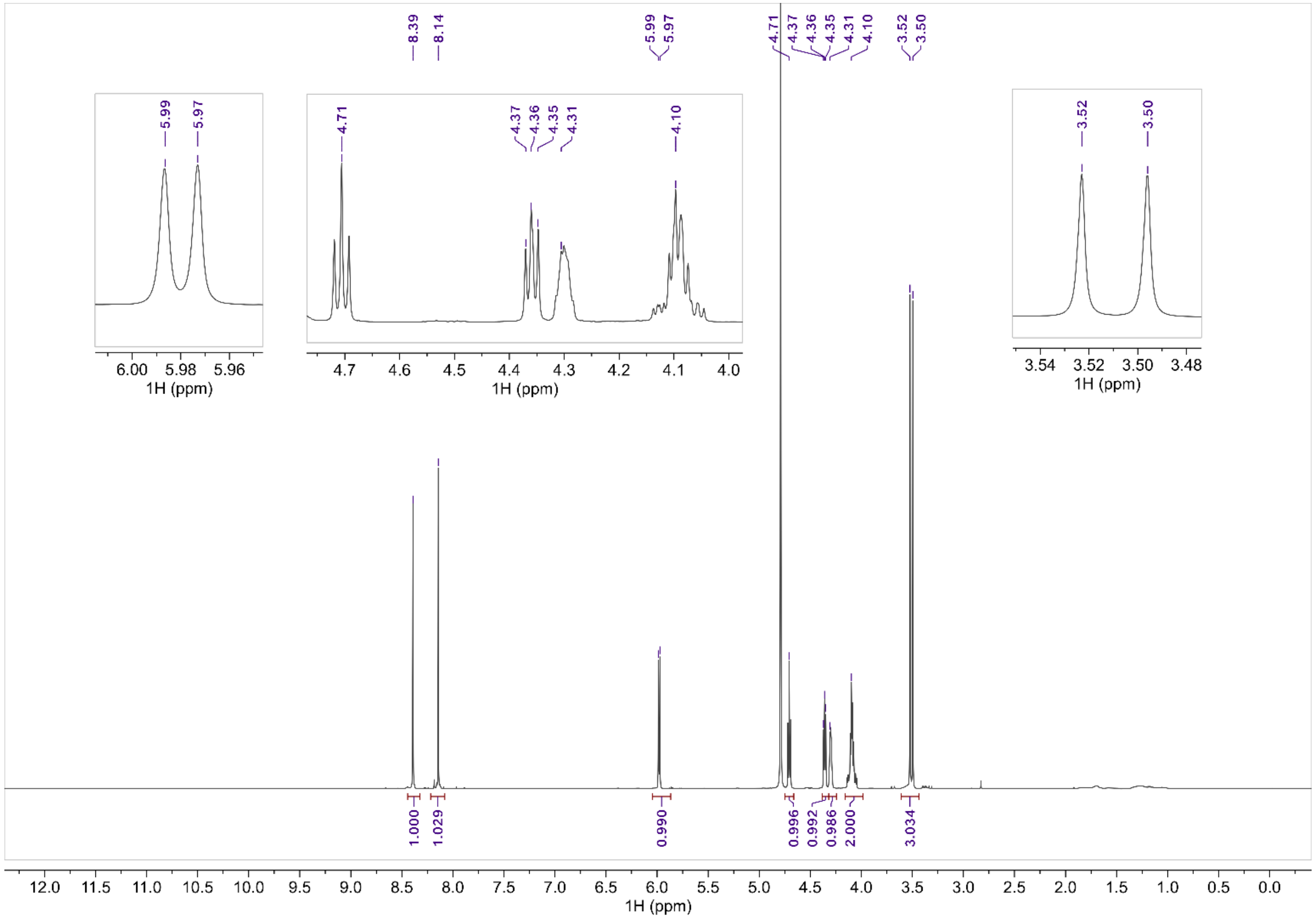

^{13}C (101 MHz, D_2O)

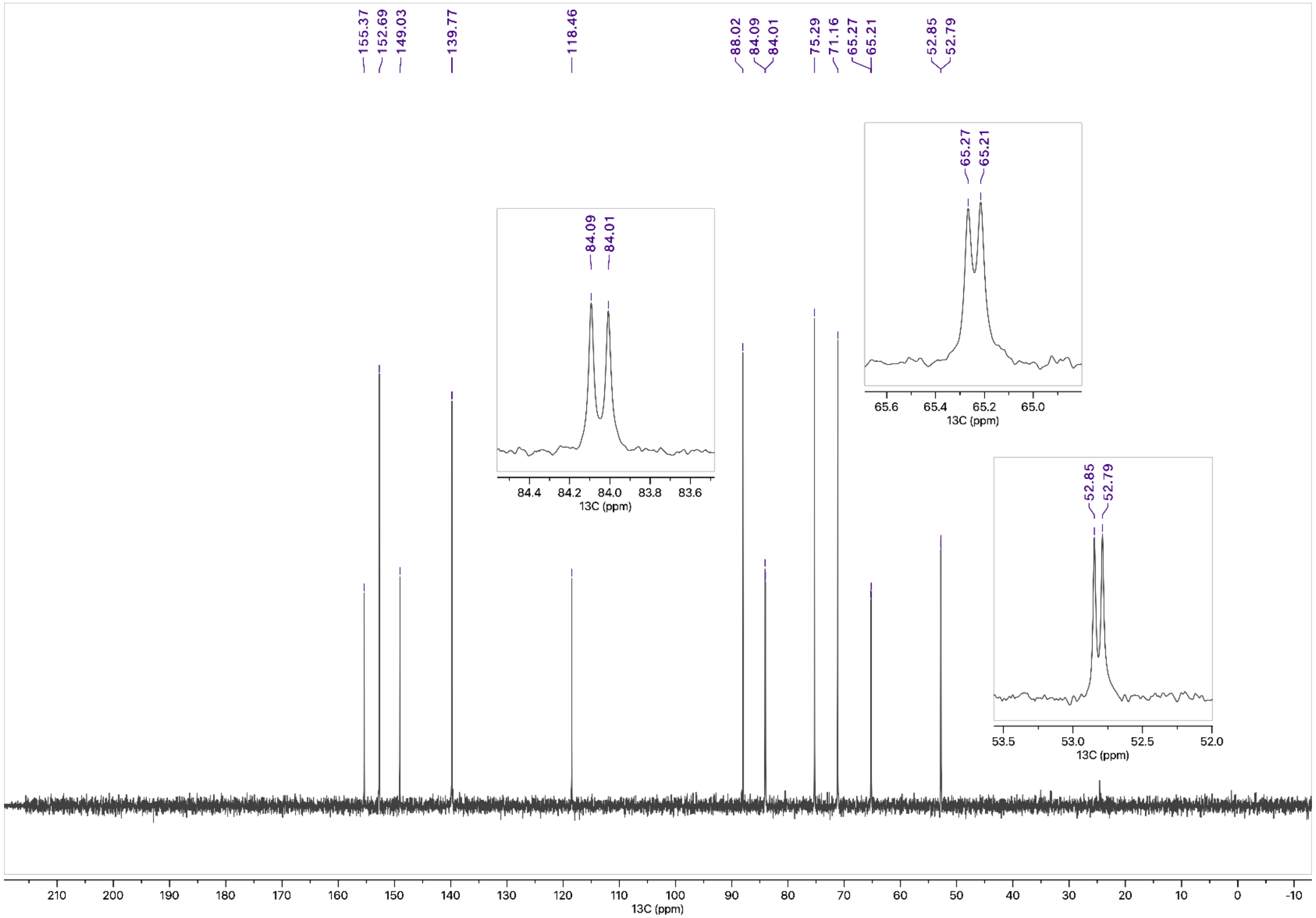

^{31}P (162 MHz, D_2O)

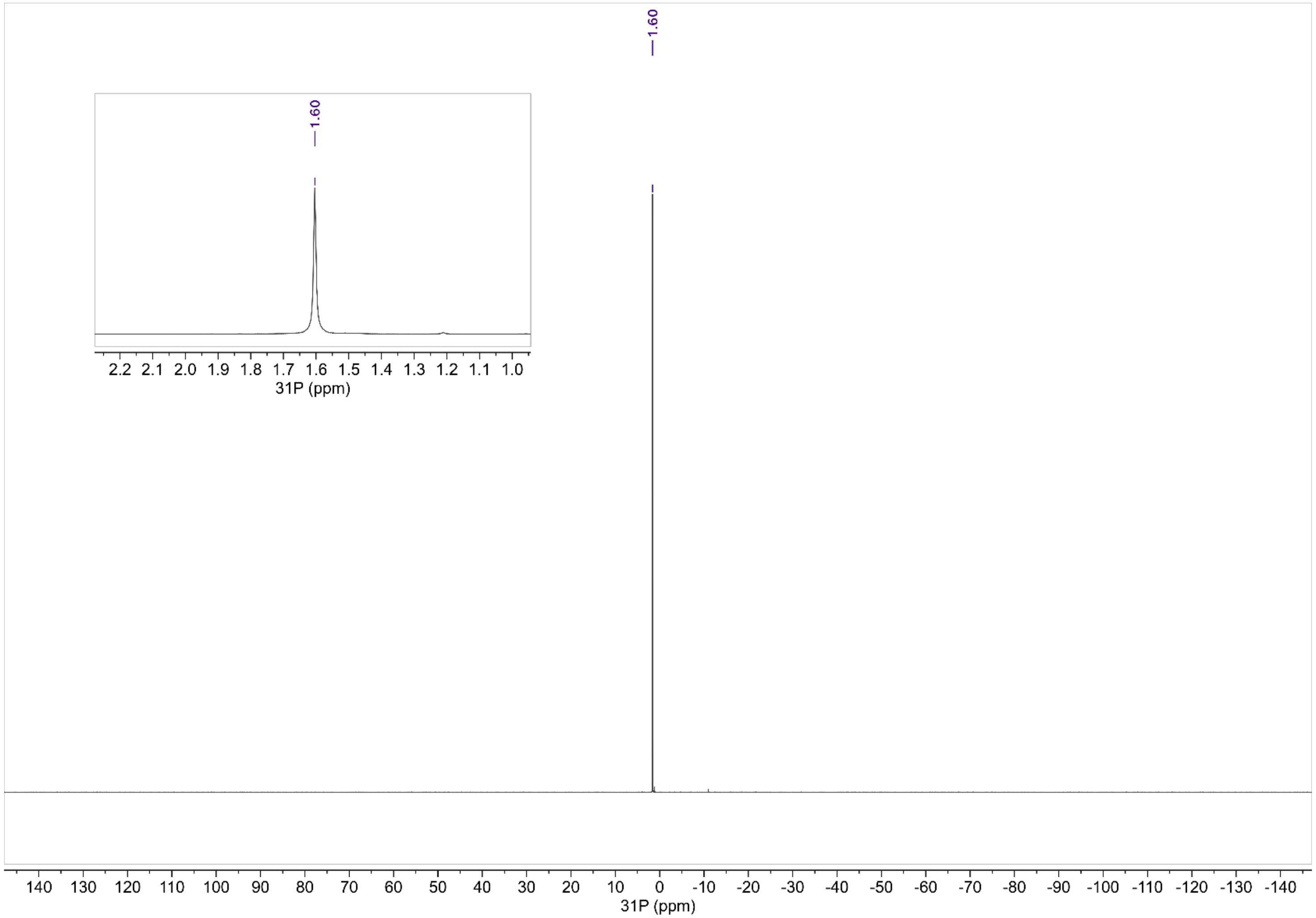

MepA-L-PheF (E_m)

^{1}H (400 MHz, D_2O)

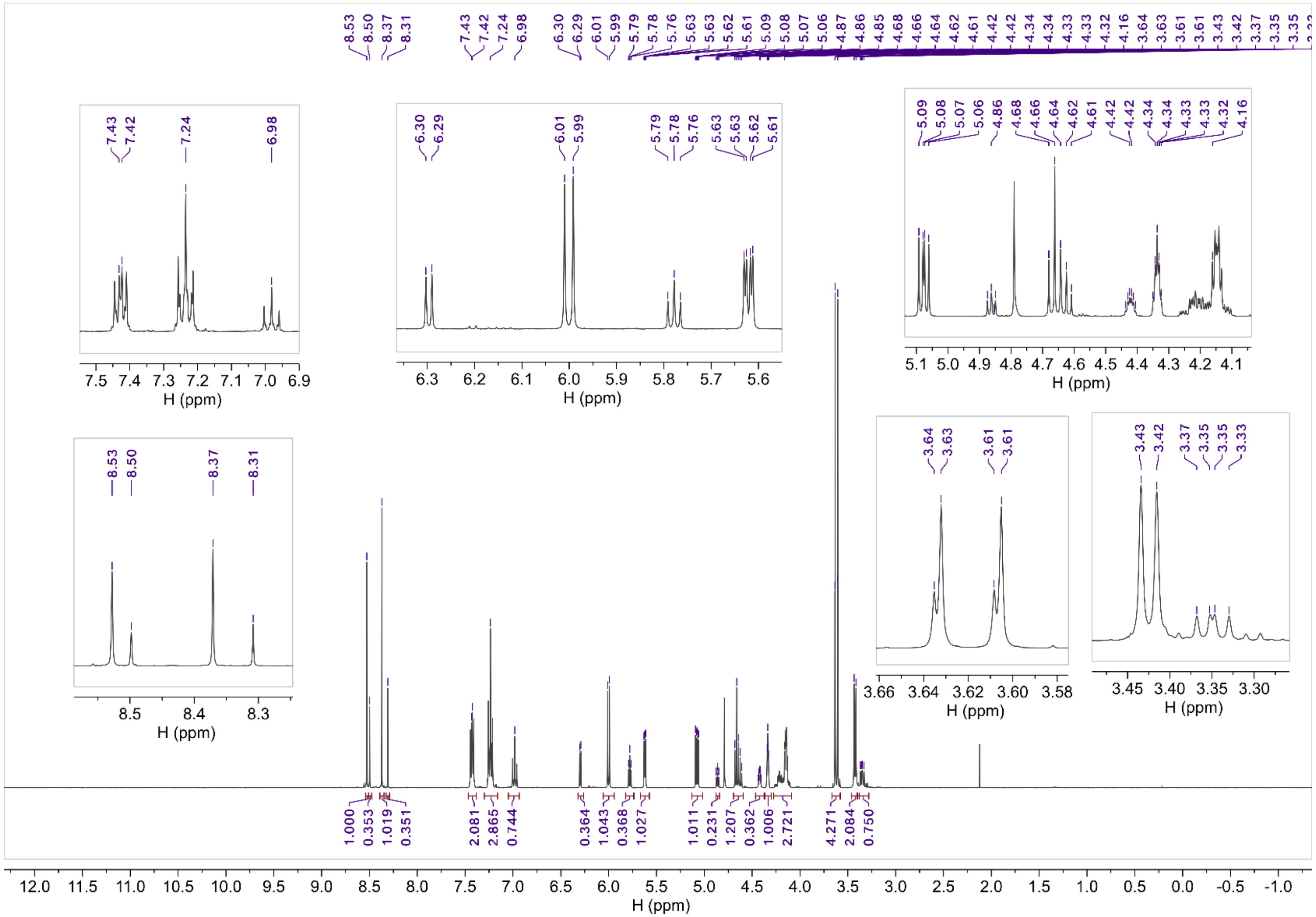

^{13}C (101 MHz, D_2O)

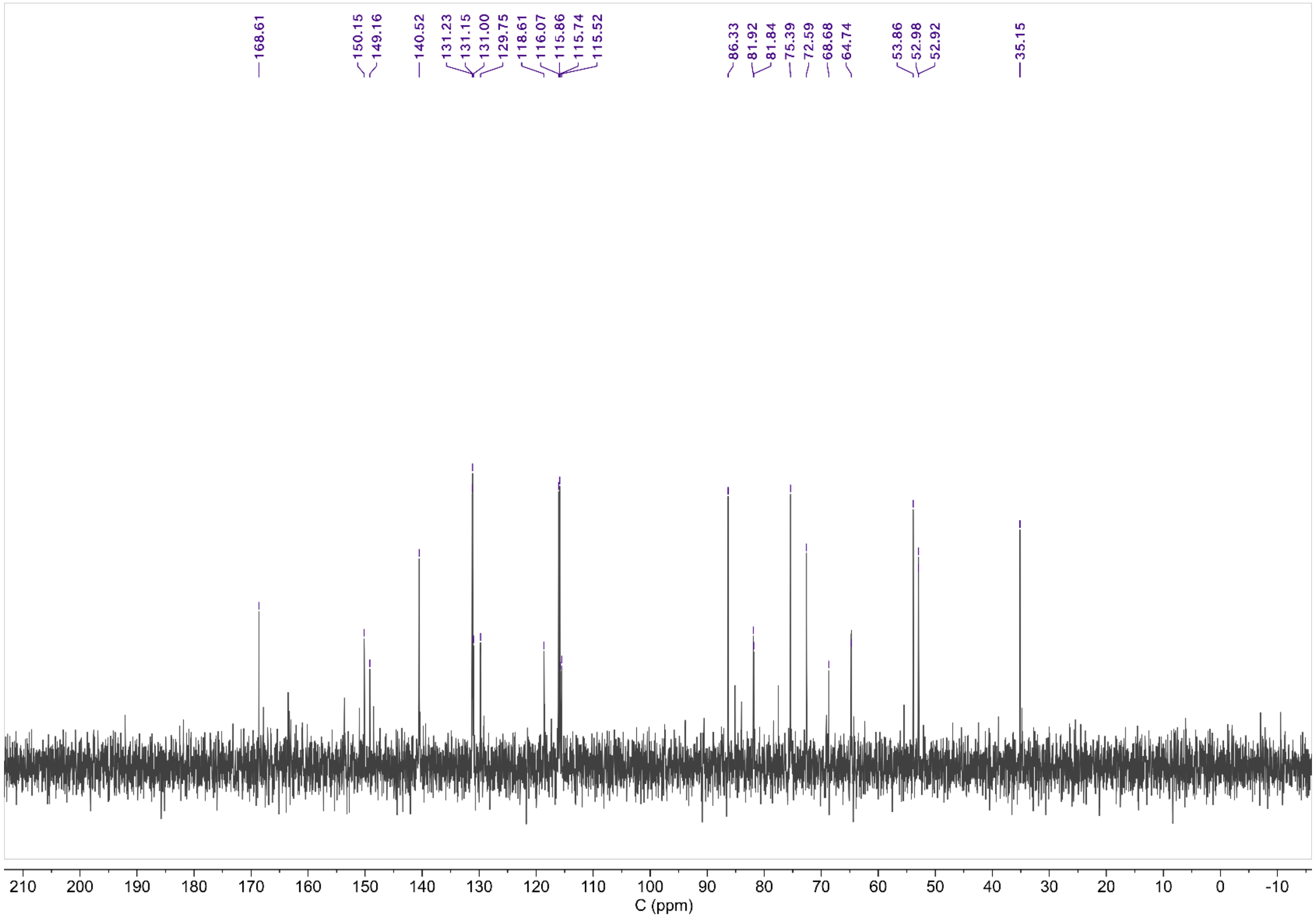

^{31}P (162 MHz, D_2O)

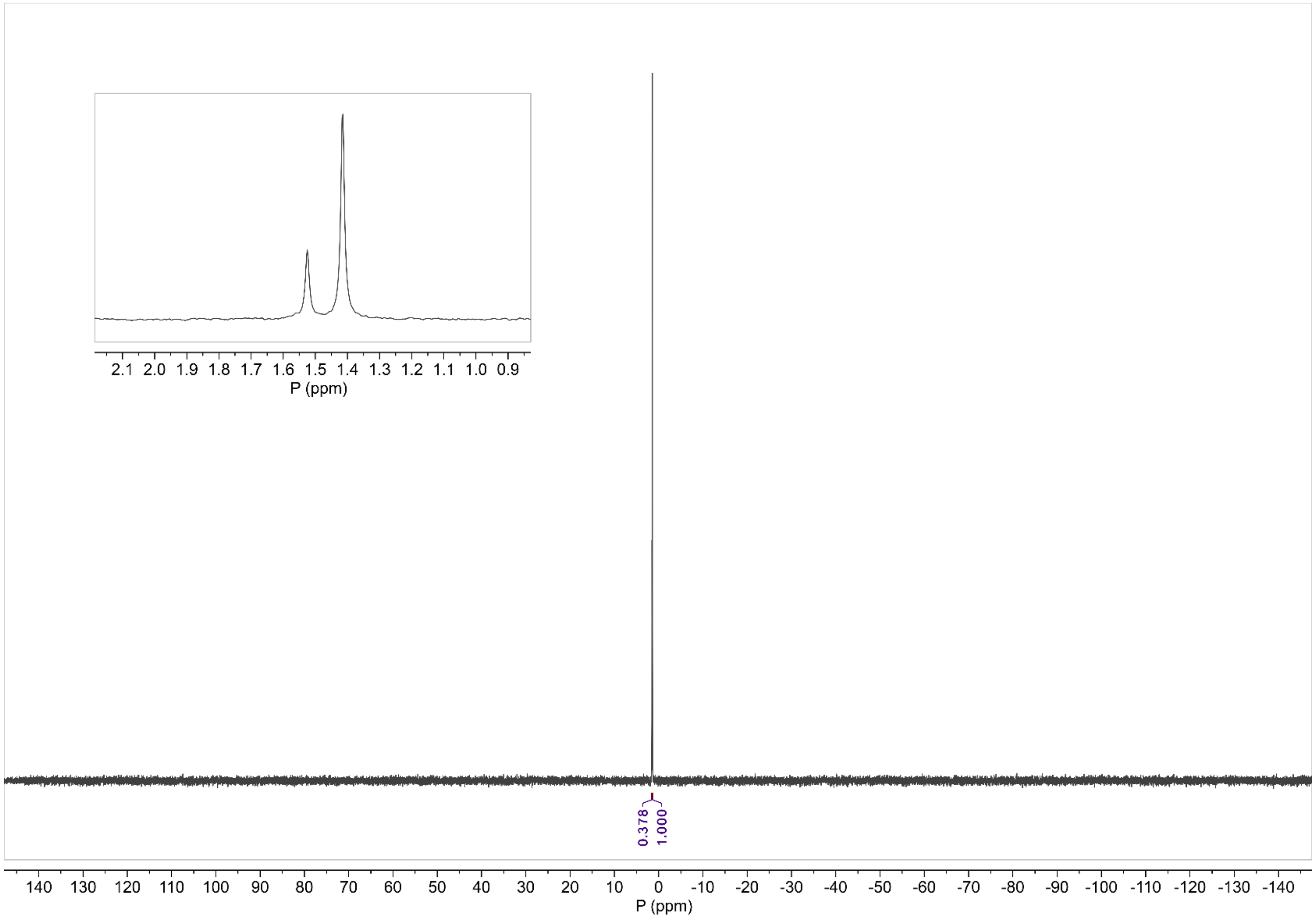

^{19}F (377 MHz, D_2O)

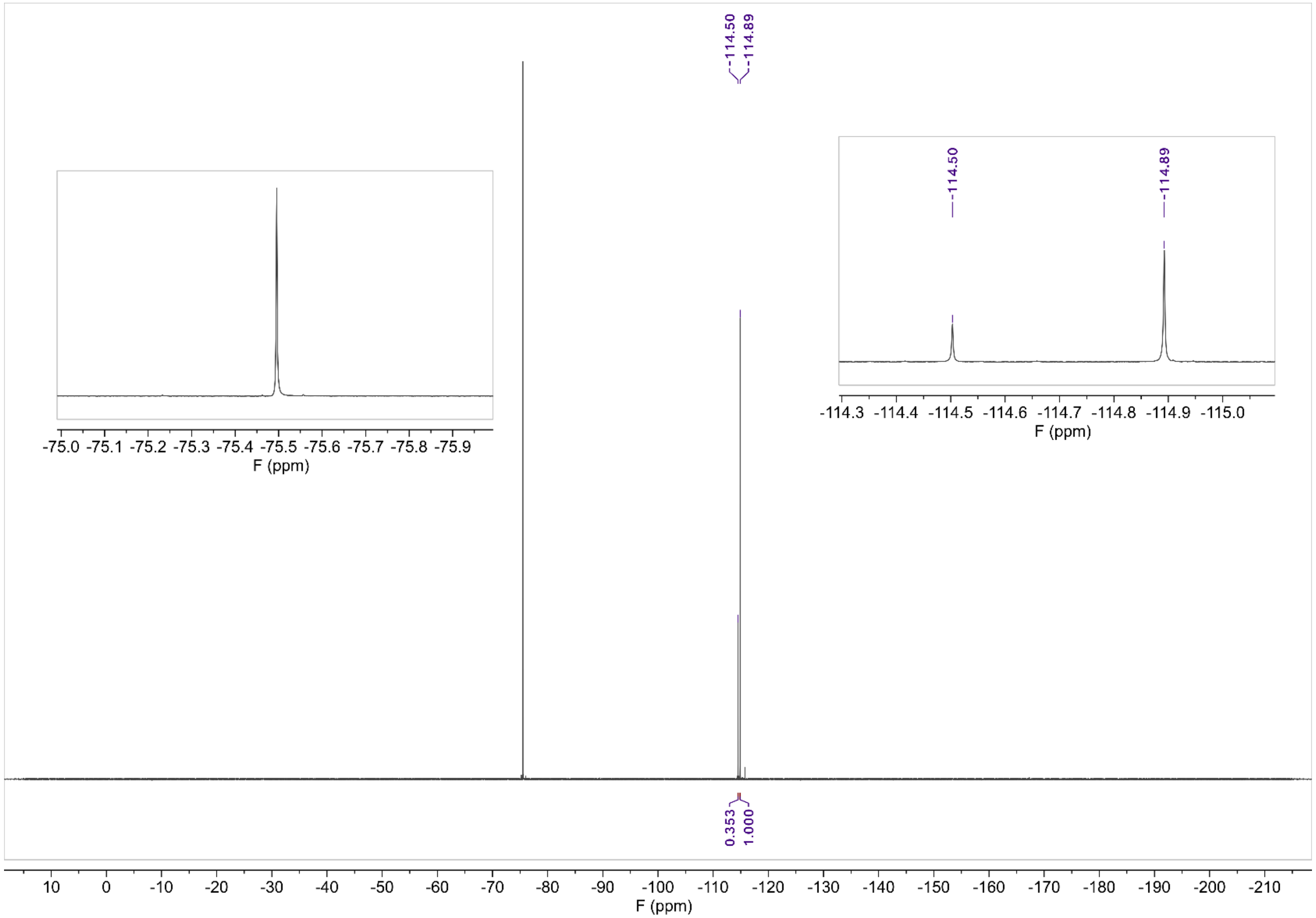

MepA-(L-PheF)$_2$ (E_{bis})

^{1}H (400 MHz, D_2O)

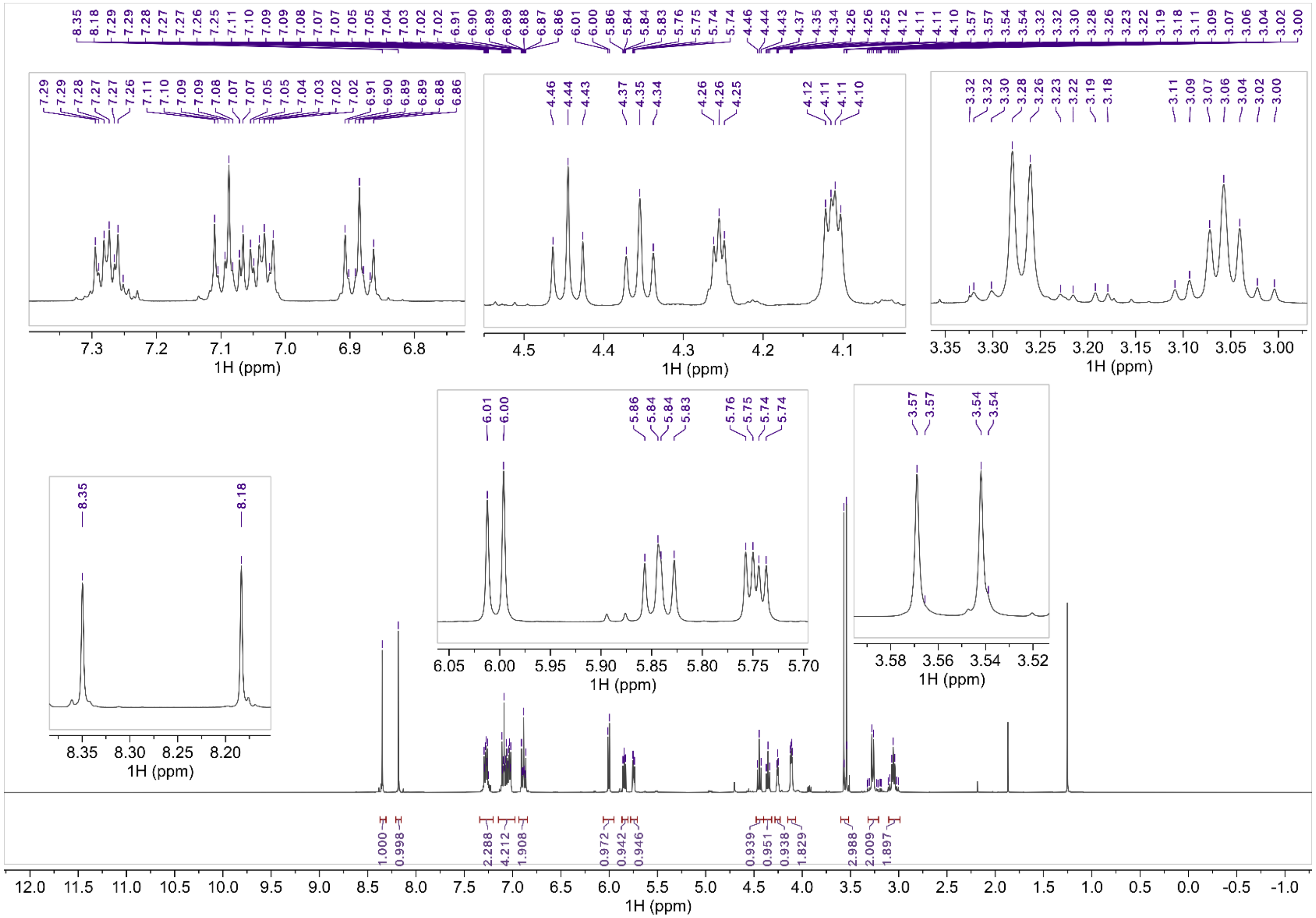

^{13}C (101 MHz, D_2O)

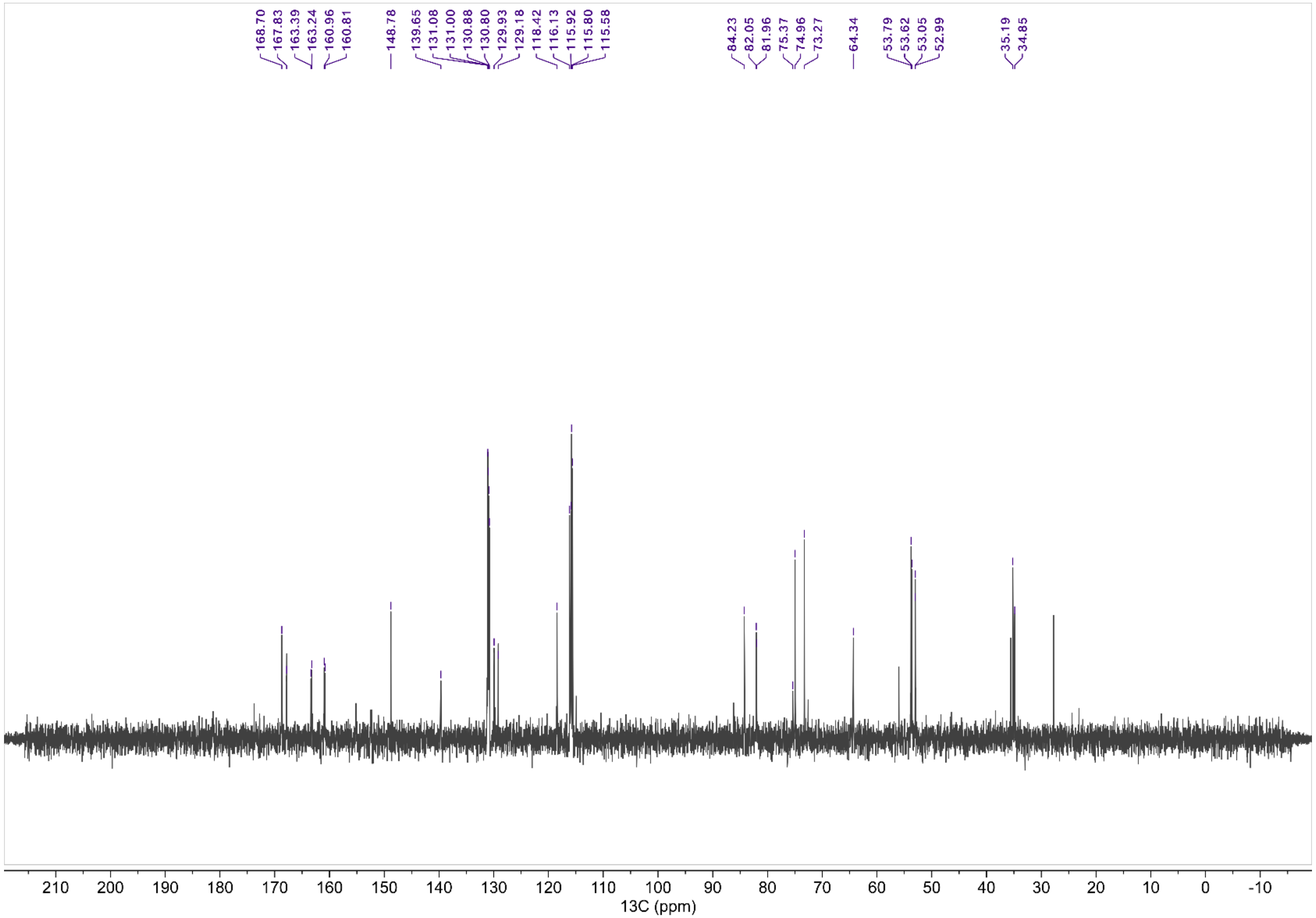

^{31}P (162 MHz, D_2O)

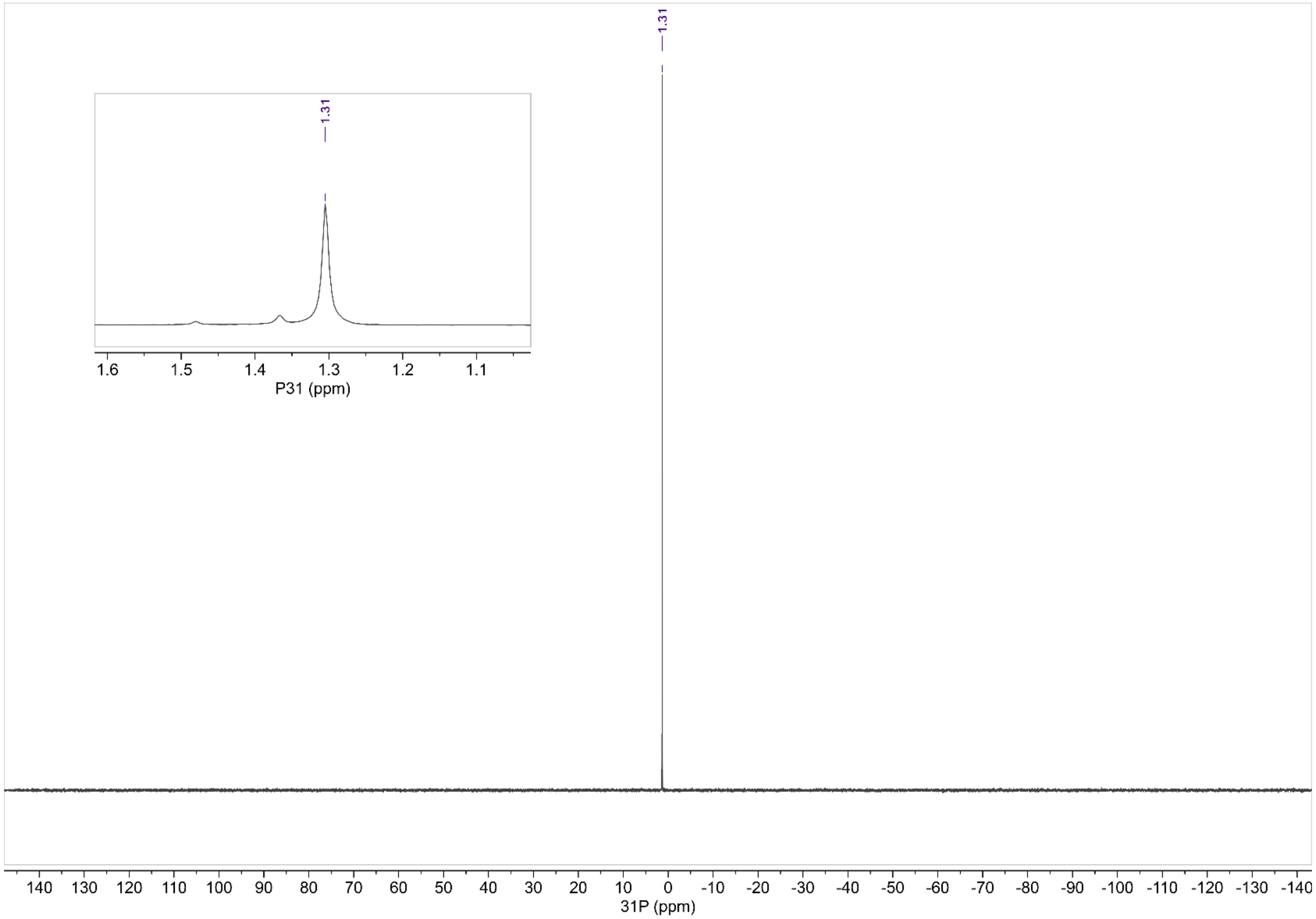

^{19}F (377 MHz, D_2O)

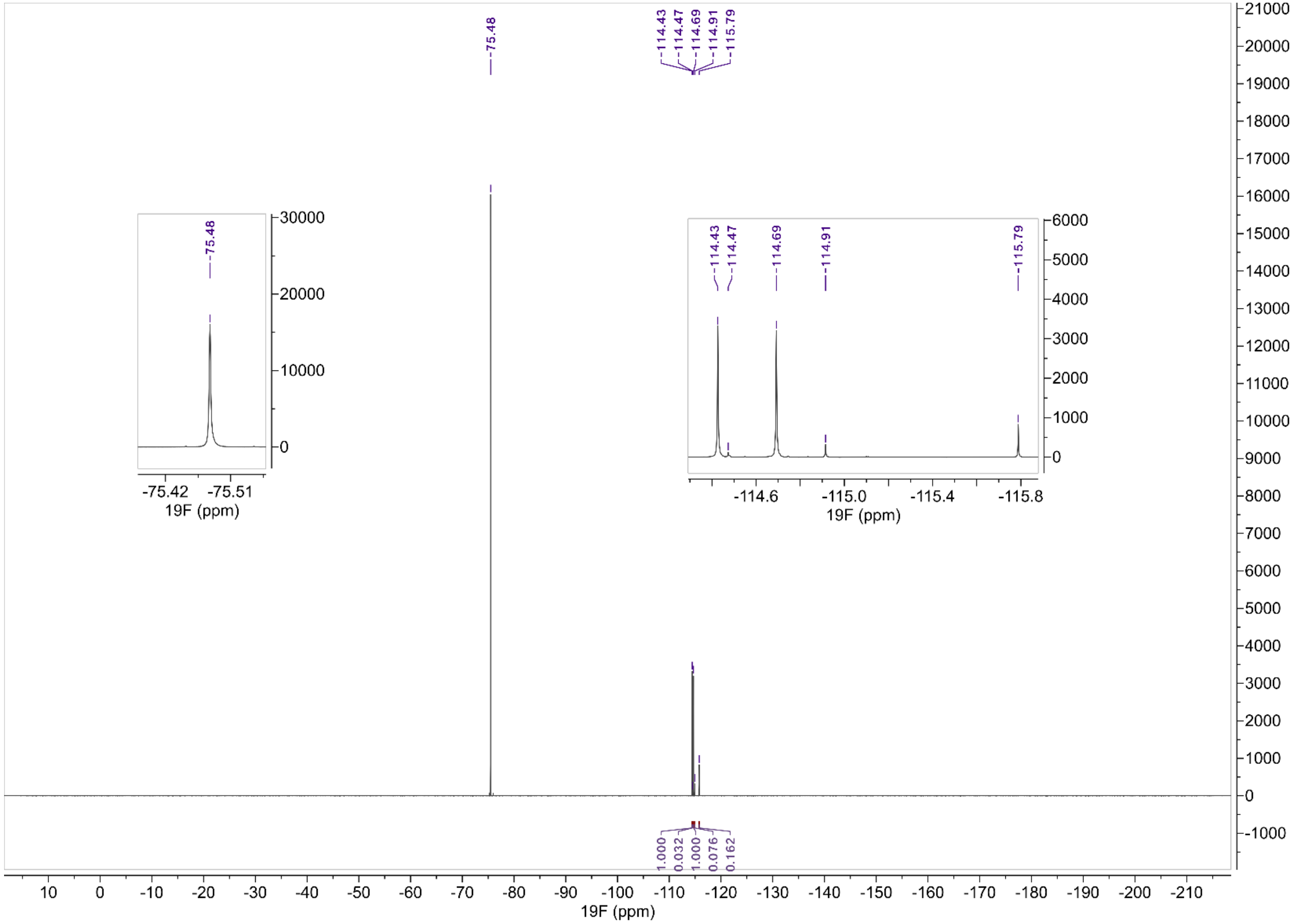

MepA-D-PheF (E_m^D)

^{1}H (400 MHz, D_2O)

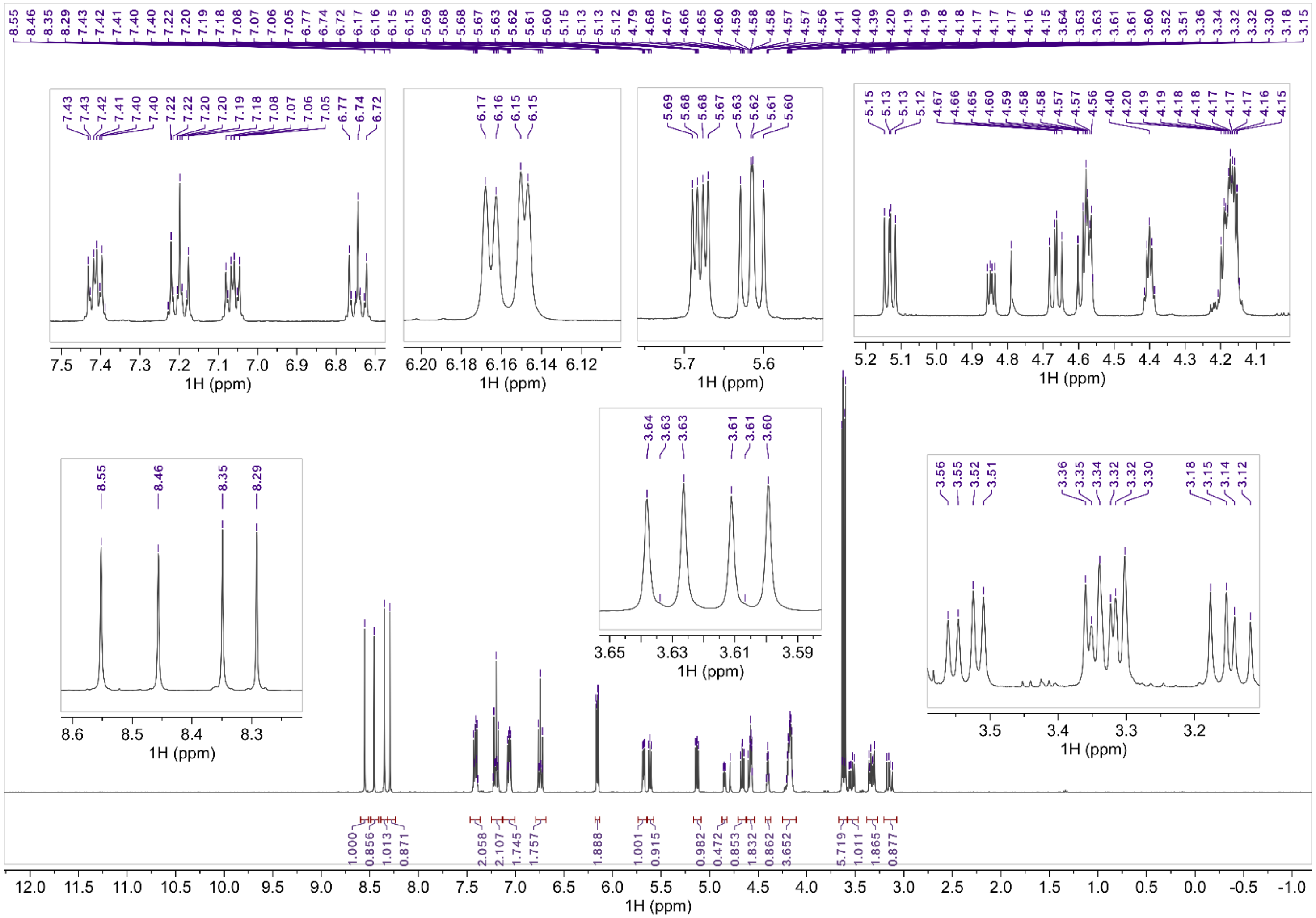

^{13}C (101 MHz, D_2O)

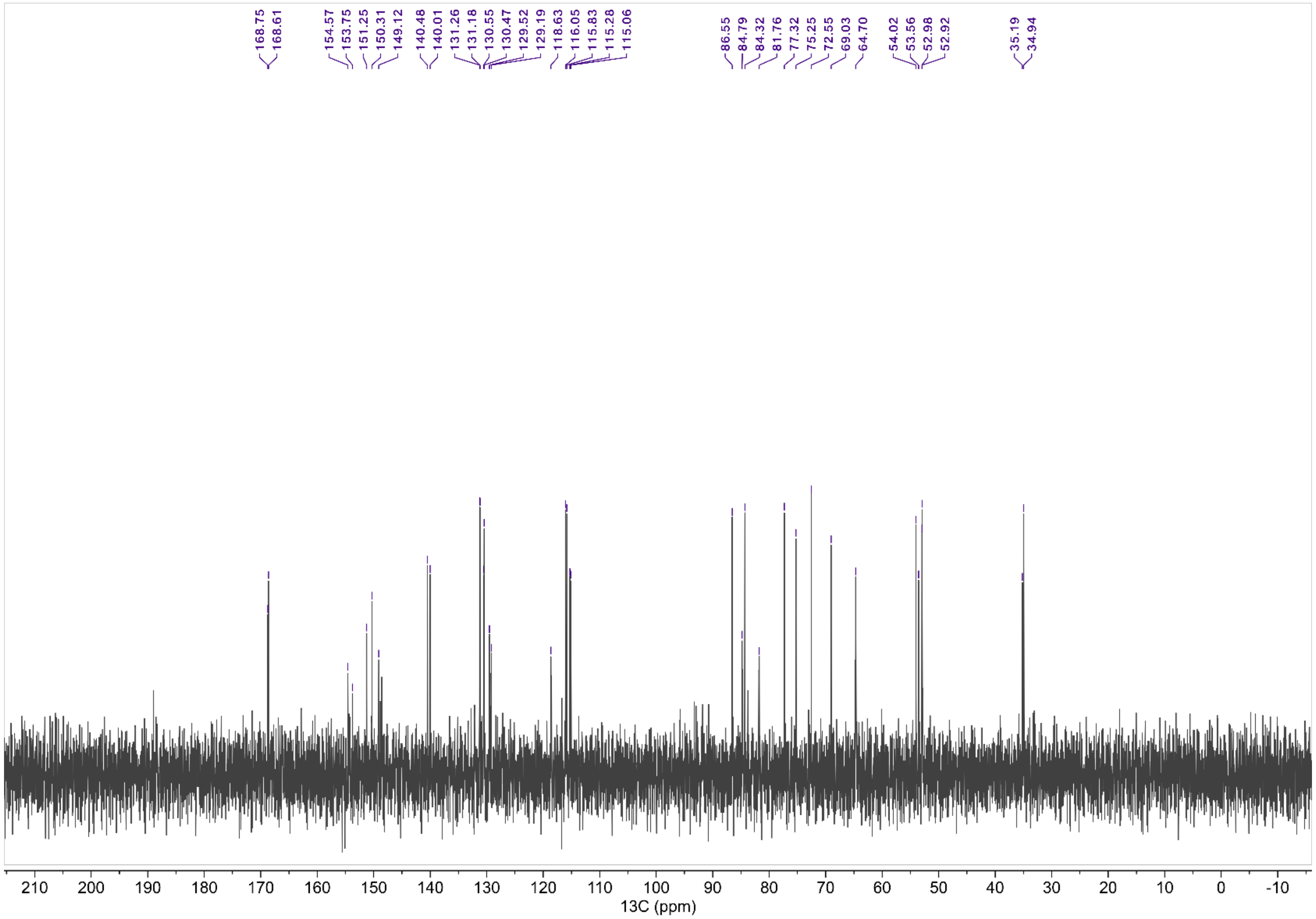

^{31}P (162 MHz, D_2O)

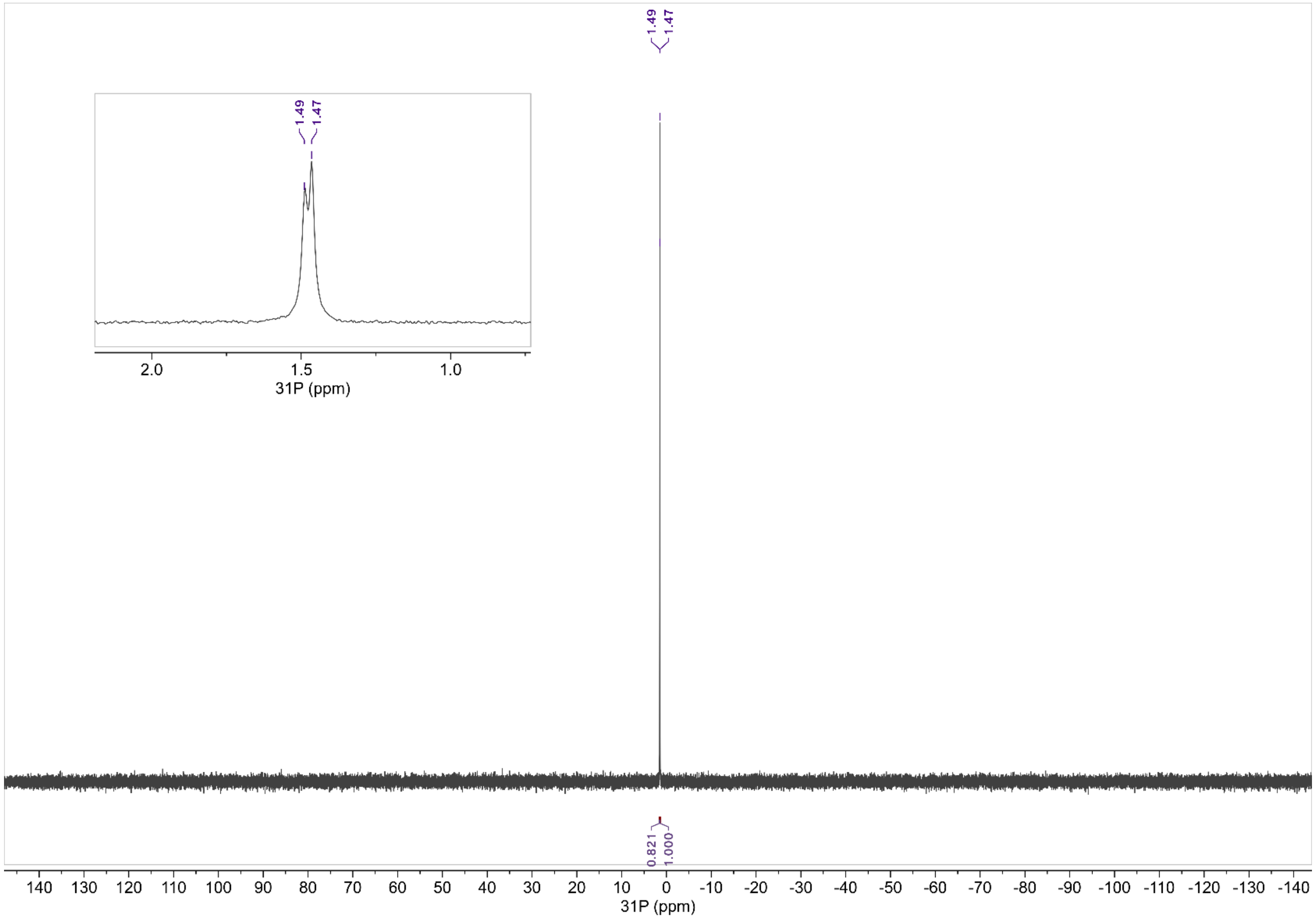

^{19}F (377 MHz, D_2O)

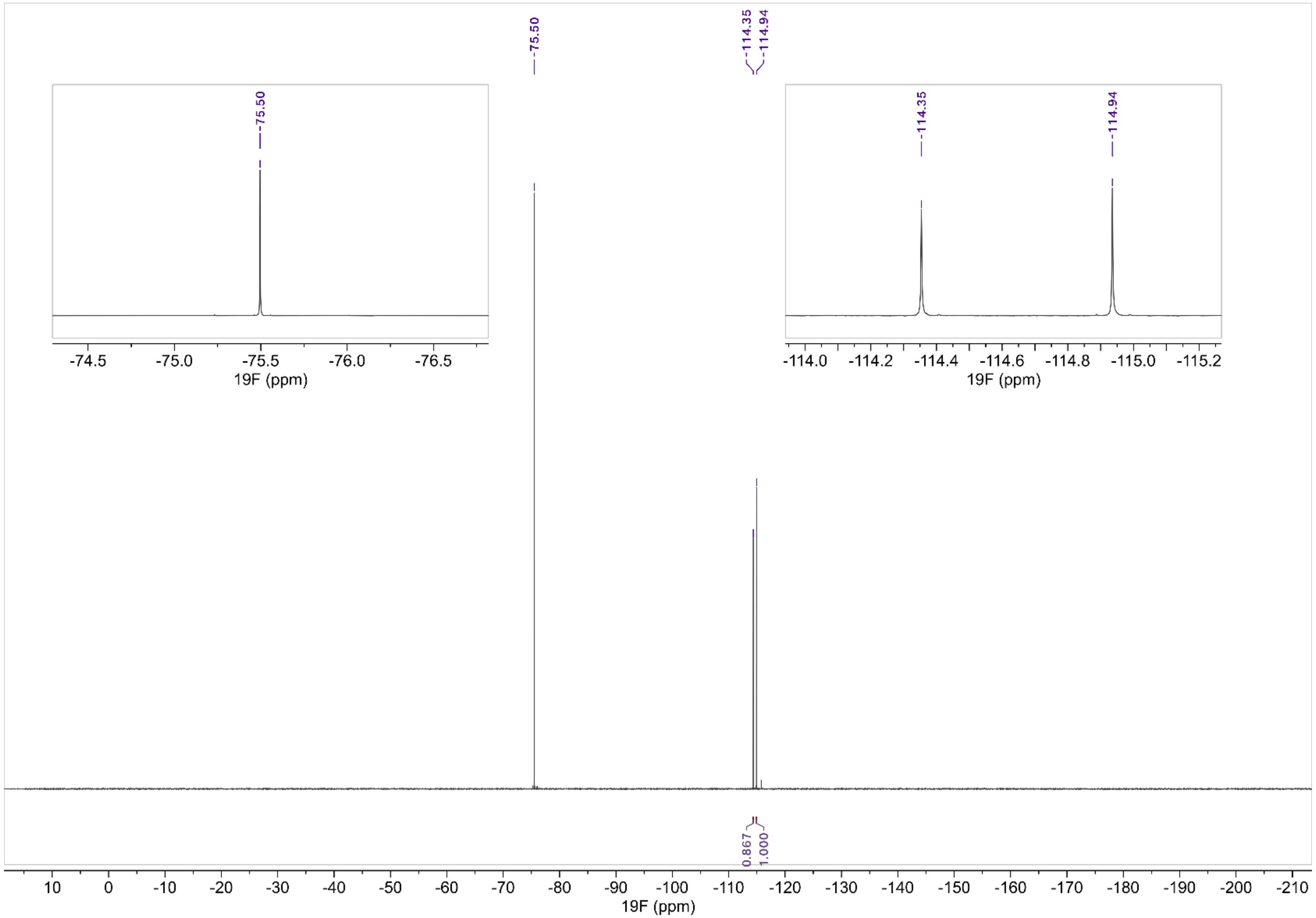

MepA-N-f-L-PheF ($E_m{}^f$)

^1H (400 MHz, D_2O)

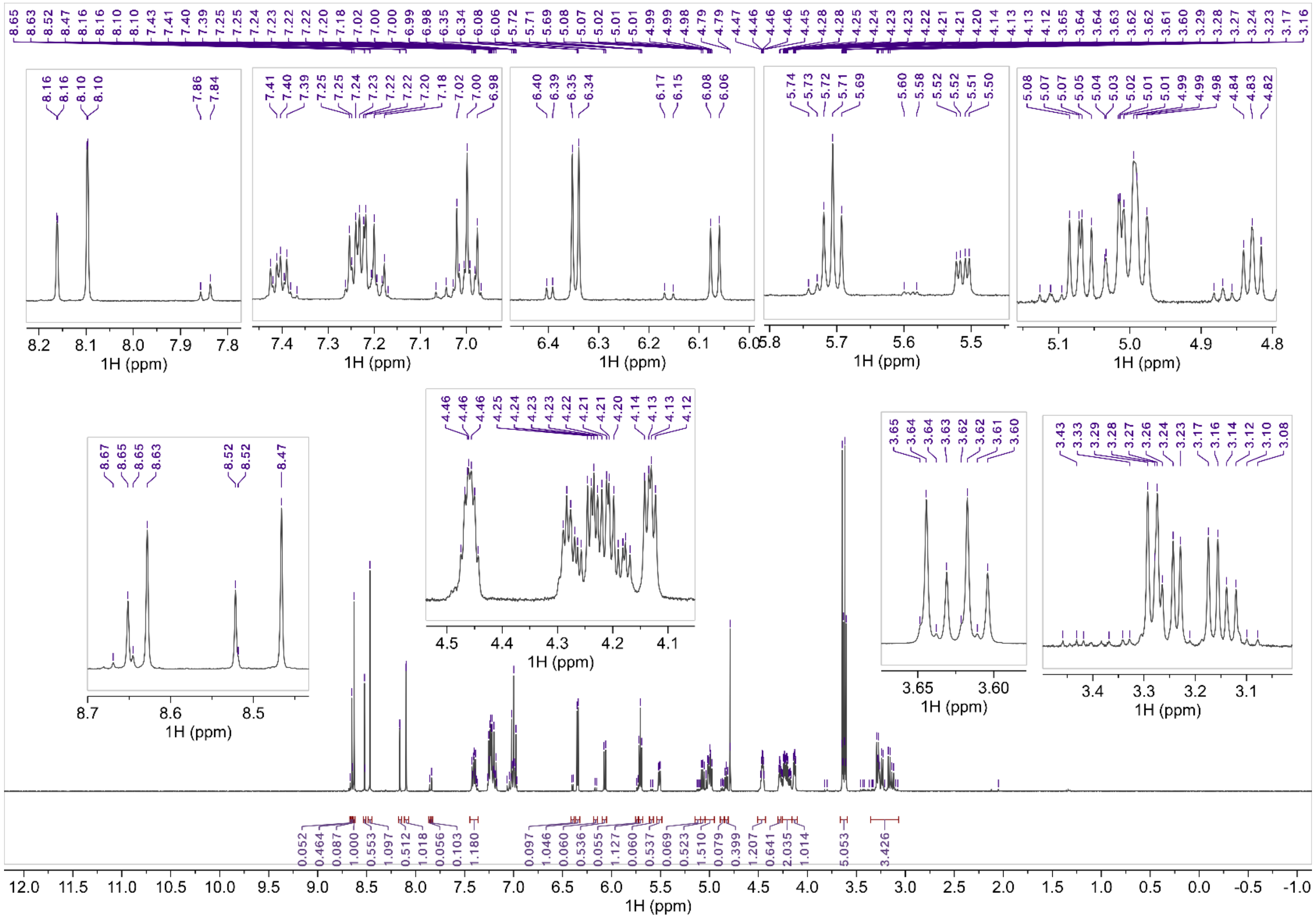

^{31}P (162 MHz, D_2O)

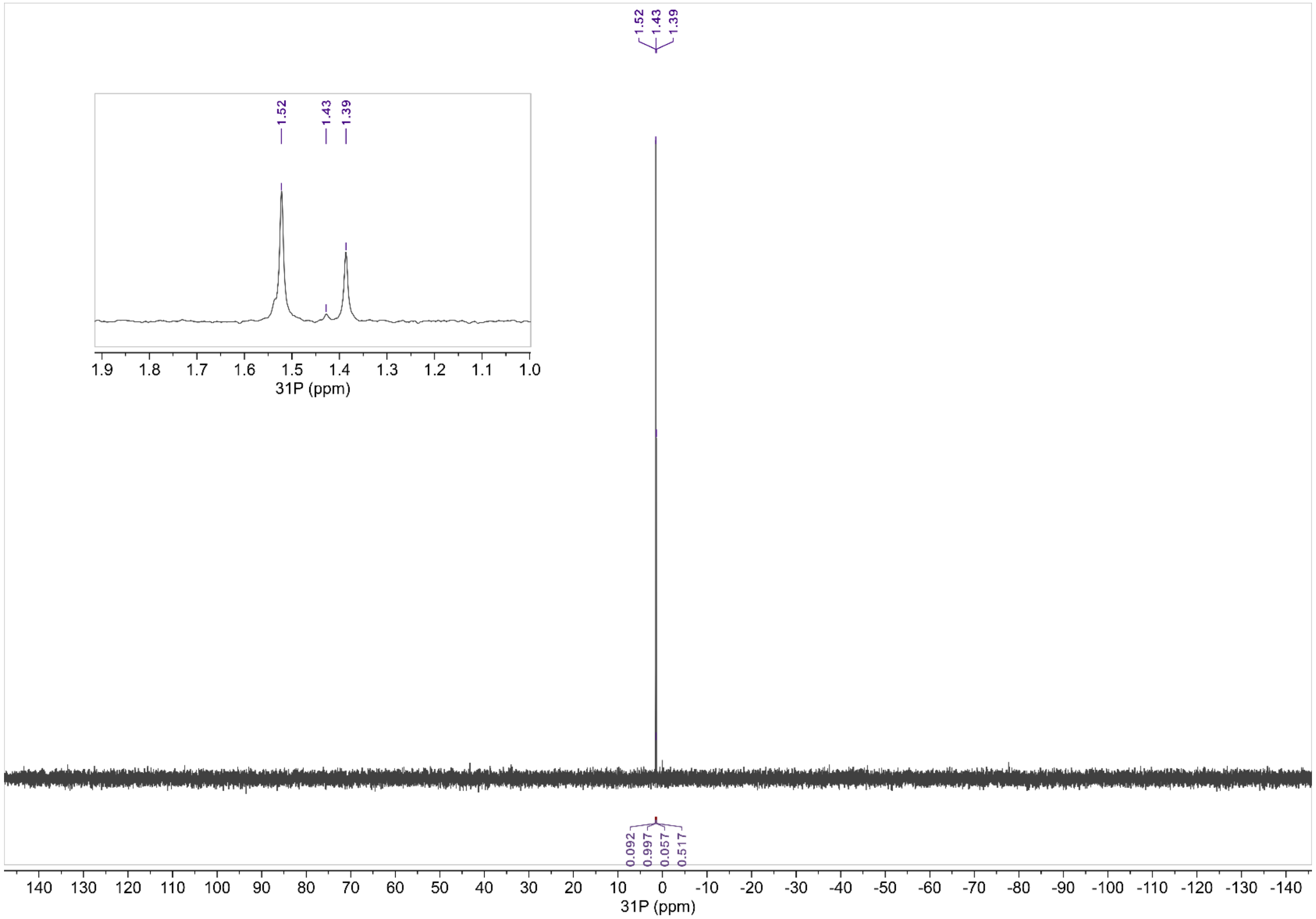

^{19}F (377 MHz, D_2O)

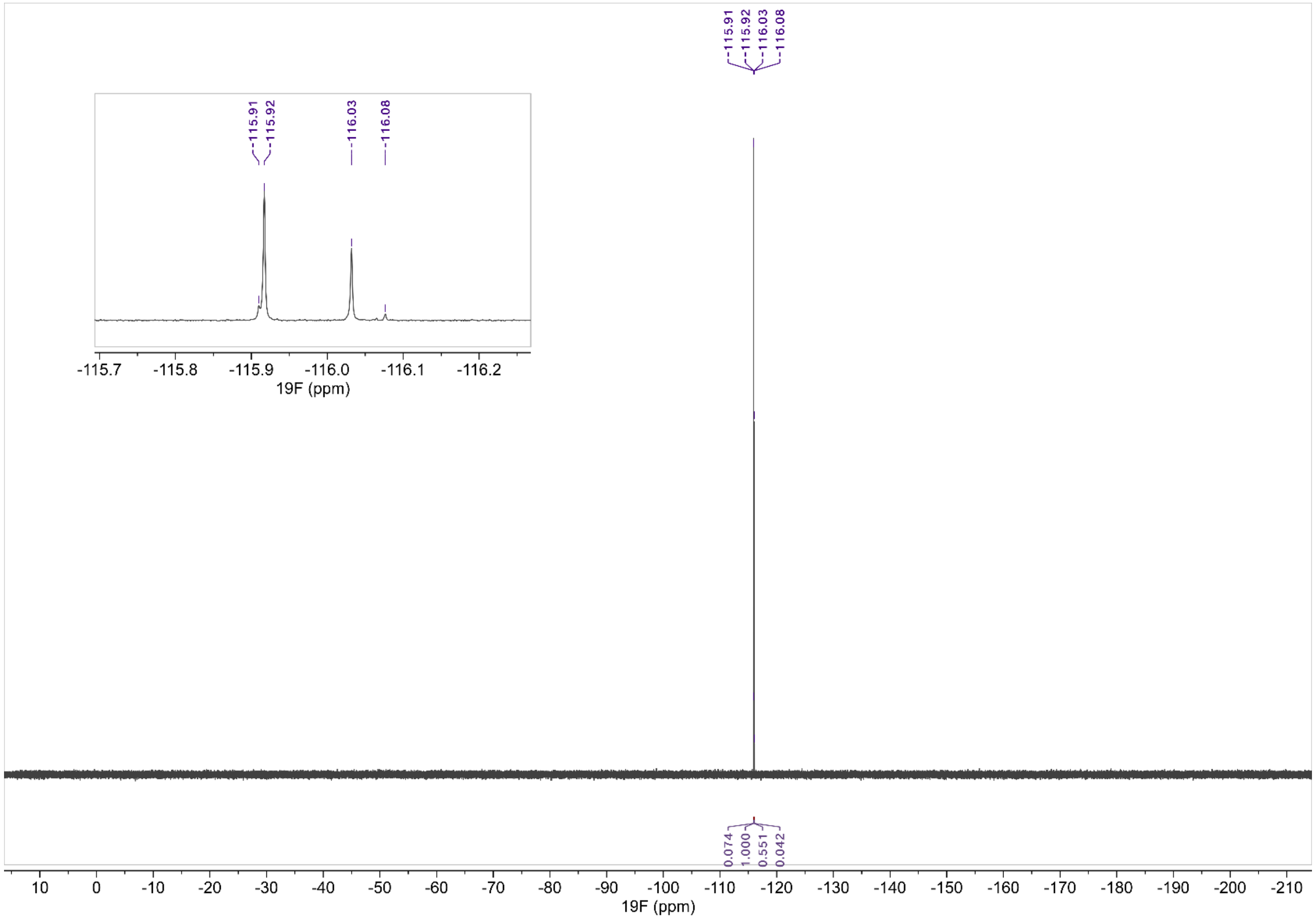

N-formyl-4-fluoro-L-phenylalanine (N-f-L-PheF-OH)

^{1}H (400 MHz, CD_3CN)

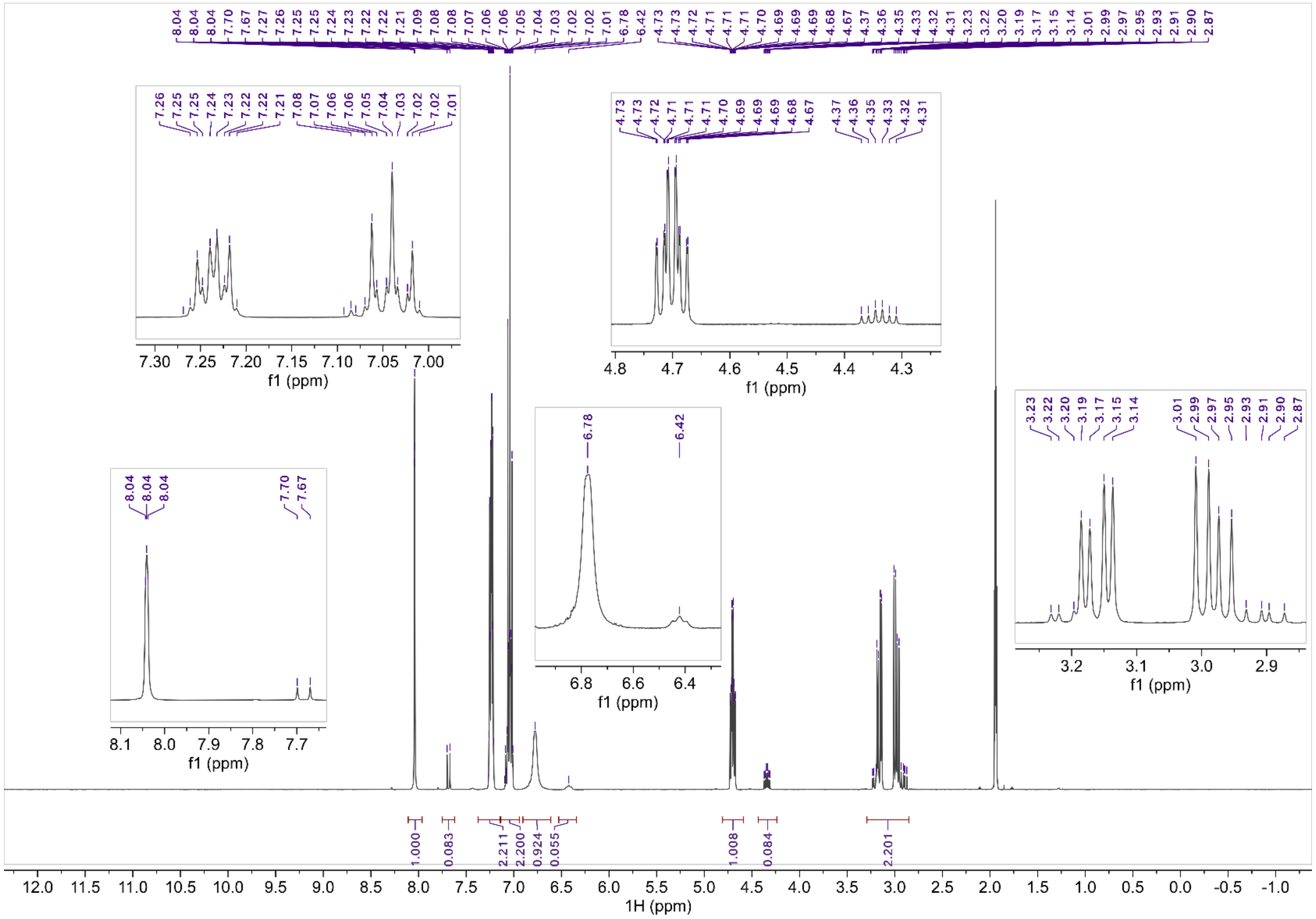

^{13}C (101 MHz, CD_3CN)

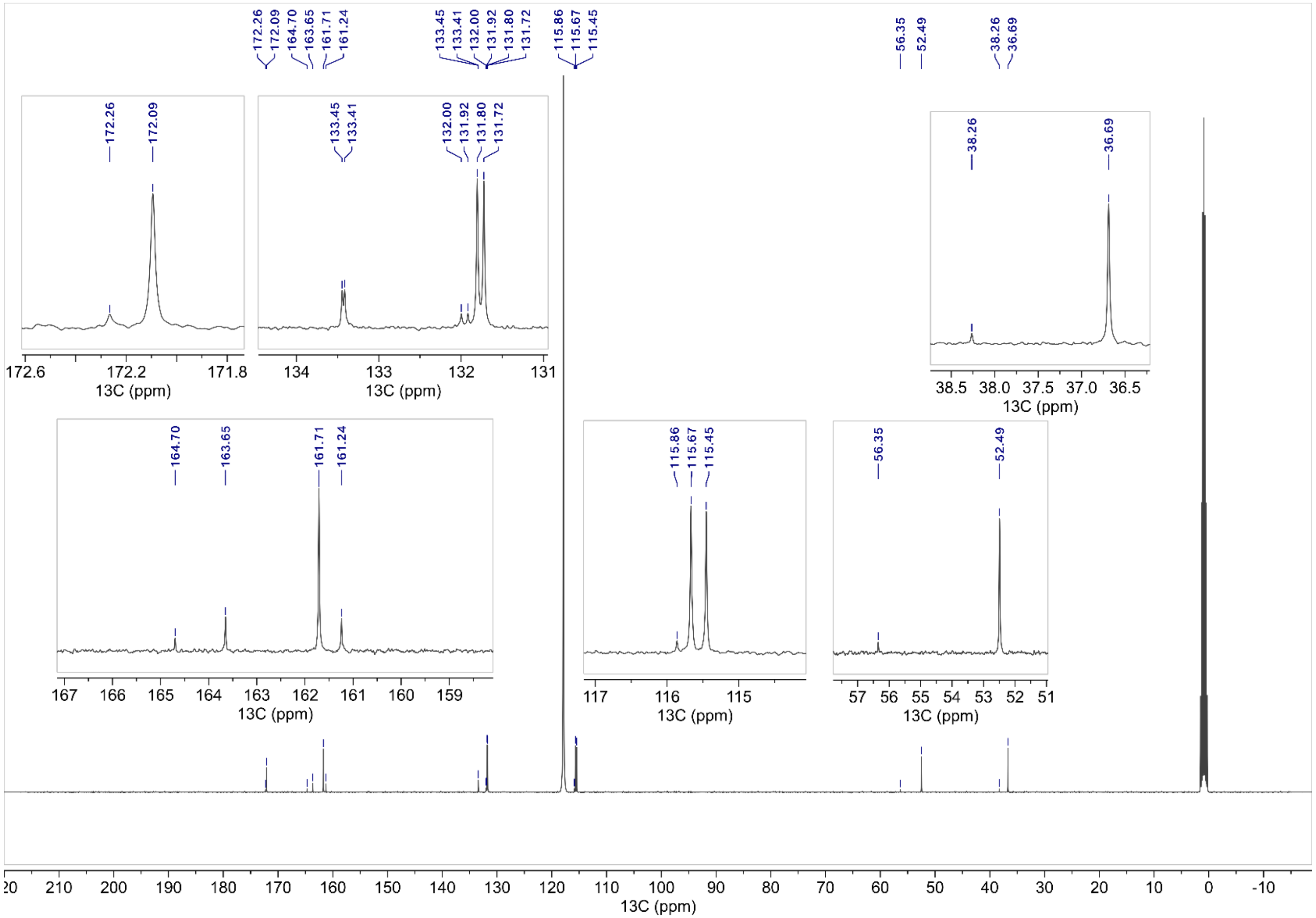

^{19}F (377 MHz, CD_3CN)

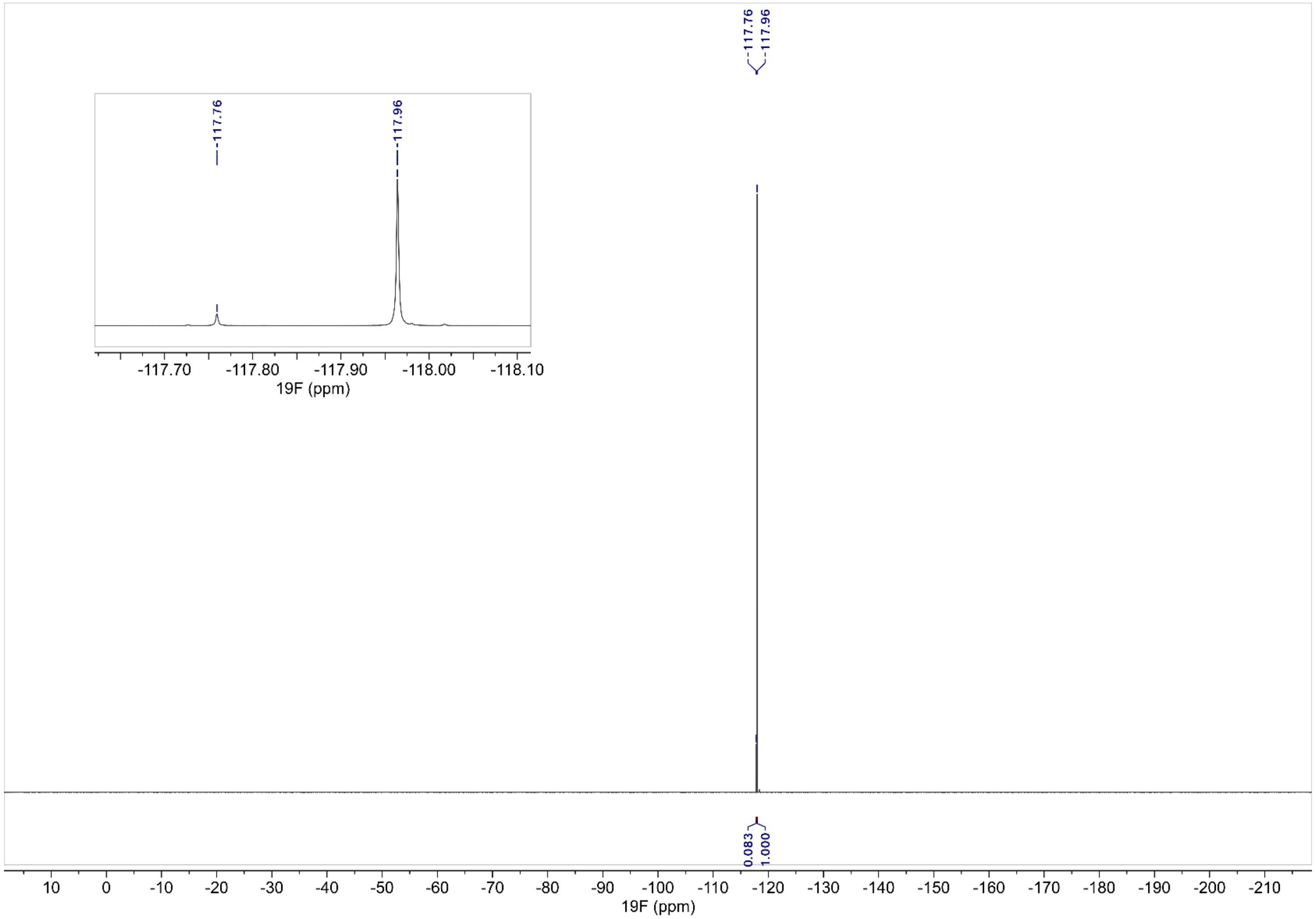

Raw characterisation data: ESI(+)-MS

MepA

Print of window 80: MS Spectrum

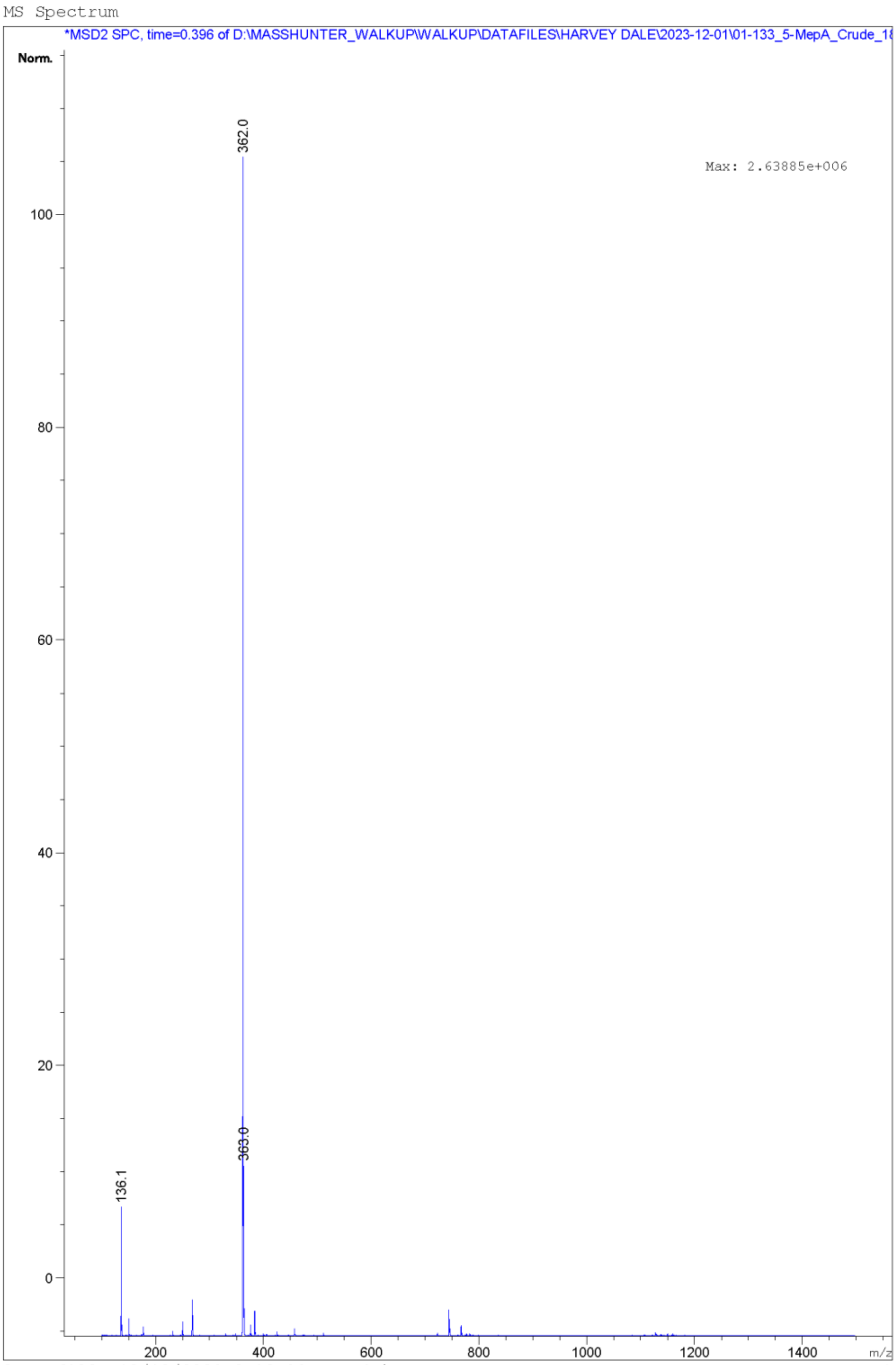

LMB_Agilent_6130B 12/10/2023 2:10:39 PM admin Page 1 of 1

MepA-L-PheF (E_m)

Print of window 80: MS Spectrum

MS Spectrum

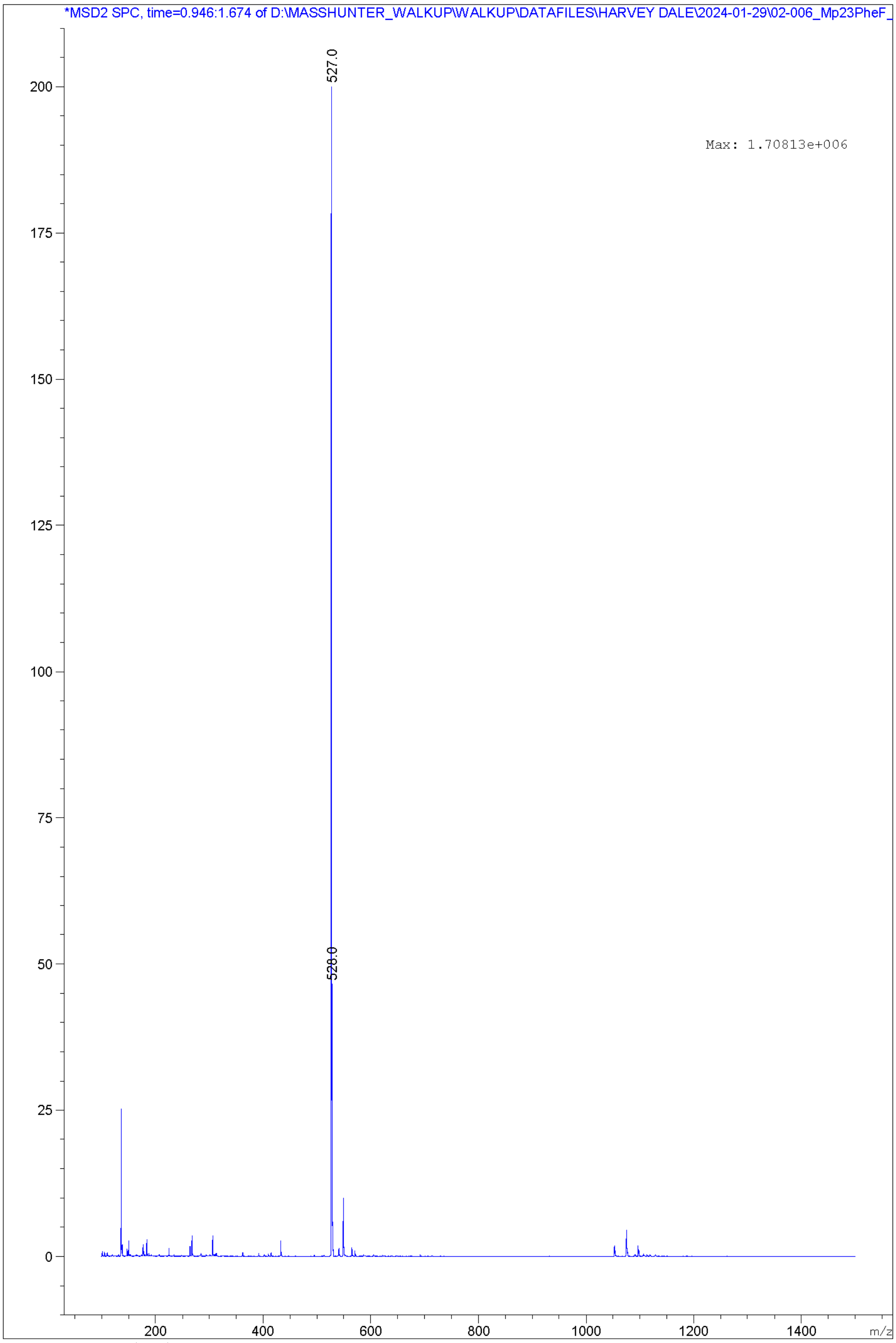

LMB_Agilent_6130B 1/31/2024 10:25:36 AM admin Page 1 of 1

MepA-(L-PheF)$_2$ (E_{bis})

Print of window 80: MS Spectrum

MS Spectrum

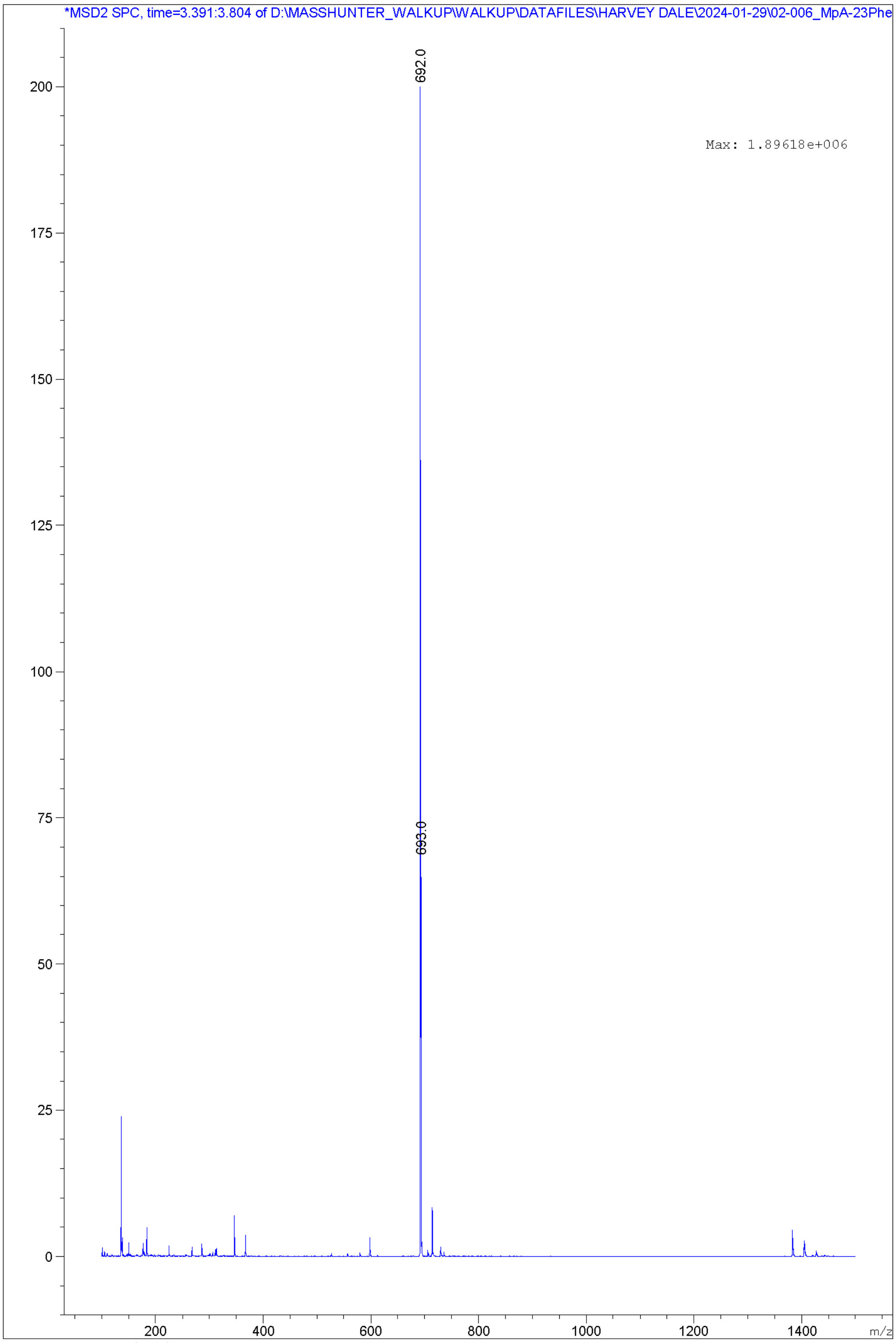

LMB_Agilent_6130B 1/31/2024 10:26:15 AM admin Page 1 of 1

MepA-D-PheF (E_m^D)

Print of window 80: MS Spectrum

MS Spectrum

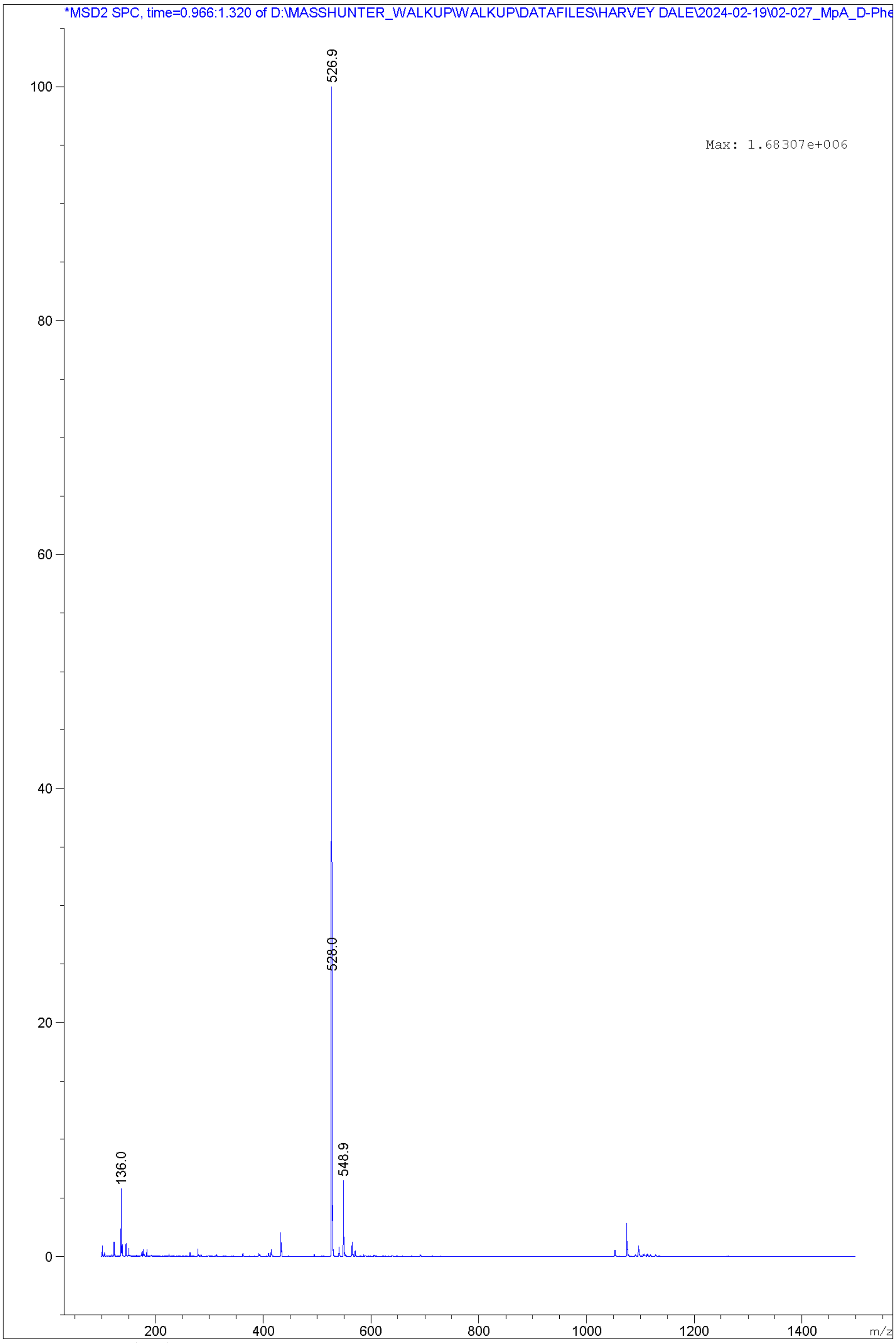

LMB_Agilent_6130B 2/21/2024 2:08:00 PM admin Page 1 of 1

MepA-N-f-L-PheF ($E_m f$)

Print of window 80: MS Spectrum

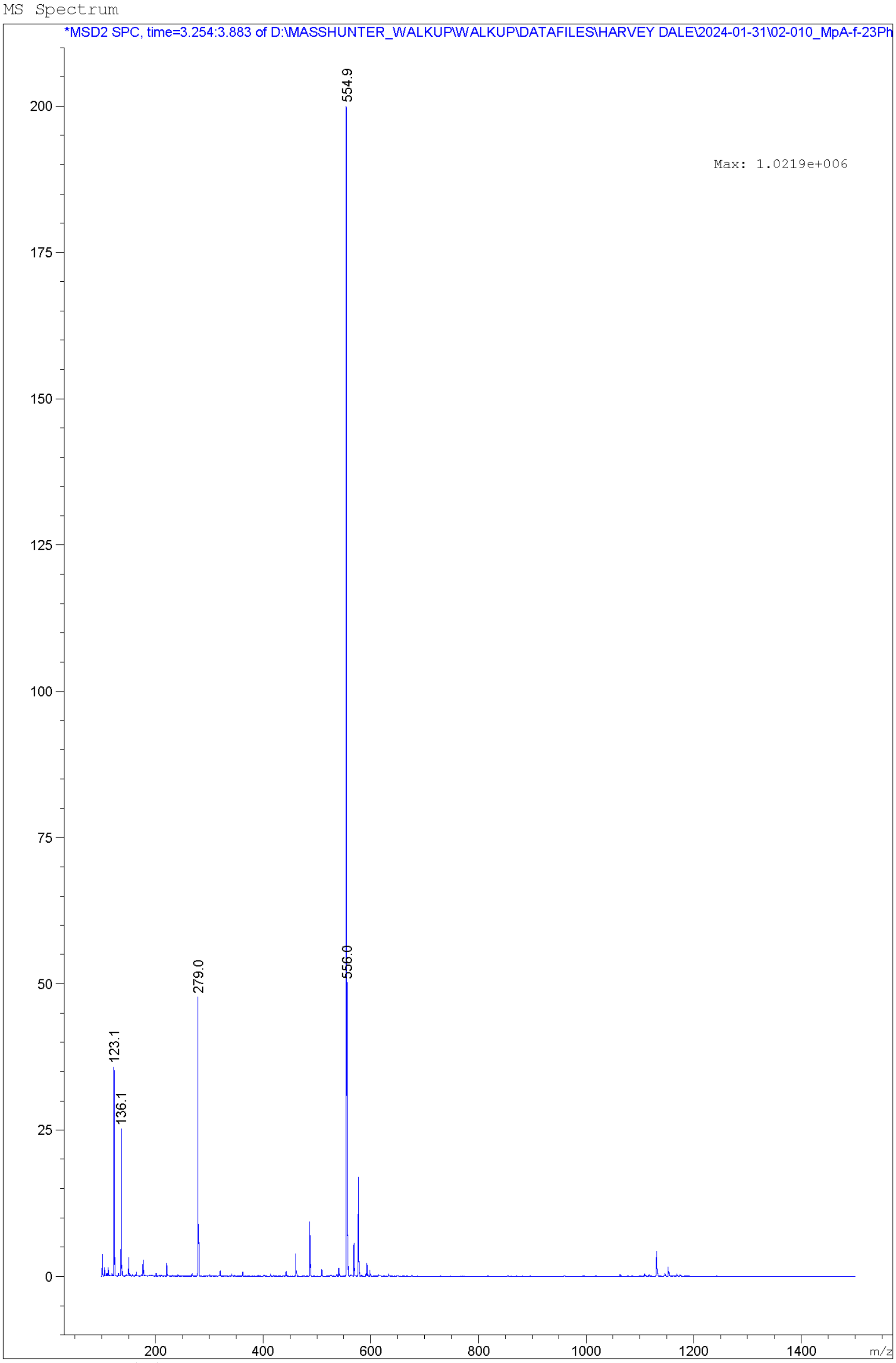

LMB_Agilent_6130B 2/1/2024 11:53:40 AM admin Page 1 of 1

N-formyl-4-fluoro-L-phenylalanine (N-f-L-PheF-OH)

Print of window 80: MS Spectrum

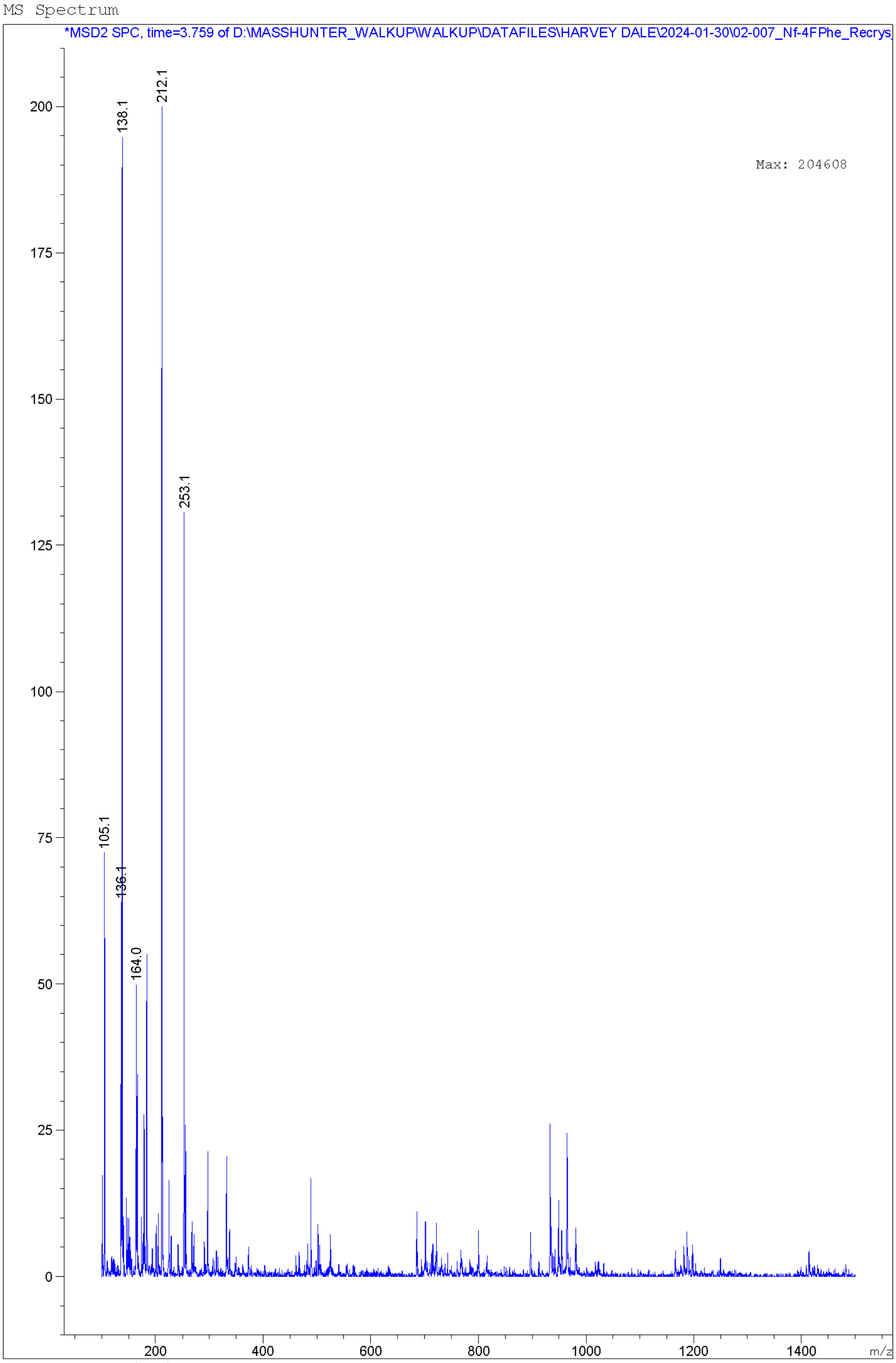

LMB_Agilent_6130B 1/31/2024 10:27:06 AM admin Page 1 of 1

References

(1) Covington, A. K.; Robinson, R. A.; Bates, R. G. The Ionization Constant of Deuterium Oxide from 5 to 50°. *J. Phys. Chem.* **1966**, *70*, 3820-3824
(2) (a) Covington, A. K.; Paabo, M.; Robinson, R. A.; Bates, R. G. Use of the glass electrode in deuterium oxide and the relation between the standardized pD (paD) scale and the operational pH in heavy water. *Anal. Chem.* **1968**, *40*, 700-706. (b) Beringer, M.; Rodnina, M. V. The Ribosomal Peptidyl Transferase. *Mol. Cell* **2007**, *26*, 311-321.
(3) Harned, H. S. The Activity Coefficient of Potassium Hydroxide in Potassium Chloride Solutions. *J. Am. Chem. Soc.* **1925**, *47*, 689-692.
(4) Zhu, J.; Moreno, I.; Quinn, P.; Yufit, D. S.; Song, L.; Young, C. M.; Duan, Z.; Tyler, A. R.; Waddell, P. G.; Hall, M. J.; et al. The Role of the Fused Ring in Bicyclic Triazolium Organocatalysts: Kinetic, X-ray, and DFT Insights. *J. Org. Chem.* **2022**, *87*, 4241-4253.
(5) de Jersey, J.; Fihelly, A. K.; Zerner, B. On the mechanism of the reaction of tris(hydroxymethyl)aminomethane with activated carbonyl compounds: A model for the serine proteinases. *Bioorg. Chem.* **1980**, *9*, 153-162.
(6) (a) Bannwarth, C.; Ehlert, S.; Grimme, S. GFN2-xTB—An Accurate and Broadly Parametrized Self-Consistent Tight-Binding Quantum Chemical Method with Multipole Electrostatics and Density-Dependent Dispersion Contributions. *J. Chem. Theory Comput.* **2019**, *15*, 1652-1671. (b) Spicher, S.; Grimme, S. Robust Atomistic Modeling of Materials, Organometallic, and Biochemical Systems. *Angew. Chem. Int. Ed.* **2020**, *59*, 15665-15673. (c) Bannwarth, C.; Caldeweyher, E.; Ehlert, S.; Hansen, A.; Pracht, P.; Seibert, J.; Spicher, S.; Grimme, S. Extended tight-binding quantum chemistry methods. *Wiley Interdiscip. Rev. Comput. Mol. Sci.* **2021**, *11*, e1493.
(7) Pracht, P.; Bohle, F.; Grimme, S.; Automated exploration of the low-energy chemical space with fast quantum chemical methods, *Phys. Chem. Chem. Phys.*, **2020**, *22*, 7169-7192.
(8) Spicher, S.; Plett, C.; Pracht, P.; Hansen, A.; Grimme, S. Automated Molecular Cluster Growing for Explicit Solvation by Efficient Force Field and Tight Binding Methods. *J. Chem. Theory Comput.* **2022**, *18*, 3174-3189.
(9) Grimme, S.; Bohle, F.; Hansen, A.; Pracht, P.; Spicher, S.; Stahn, M. Efficient Quantum Chemical Calculation of Structure Ensembles and Free Energies for Nonrigid Molecules. *J. Phys. Chem. A* **2021**, *125*, 4039-4054.
(10) Neese, F. Software update: The ORCA program system—Version 5.0. *Wiley Interdiscip. Rev. Comput. Mol. Sci.* **2022**, *12*, e1606.
(11) Śmiechowski, M. Theoretical pKa prediction of O-phosphoserine in aqueous solution. *Chem. Phys. Lett.* **2010**, *501*, 123-129.
